# Supplementary material for: Allylic C–H oxygenation of unactivated internal olefins by the Cu/azodiformate catalyst system
Source: Nat Commun. 2025 Jan 20;16:870. doi: 10.1038/s41467-025-56230-0 (PMC11756401; doi:10.1038/s41467-025-56230-0)
Supplement: Supplementary file 1 — Supplementary Information [file 41467_2025_56230_MOESM1_ESM.pdf]

Supplementary Information

Allylic C−H Oxygenation of Unactivated Internal Olefins by the Cu/Azodiformate Catalyst System

Le Wang, Yuan She, Jie Xiao, Zi-Hao Li, Shen-Yuan Zhang, Peng-Fei Lian, Tong-Mei Ding and Shu-Yu Zhang*

Shanghai Key Laboratory for Molecular Engineering of Chiral Drugs/School of Chemistry and Chemical Engineering, Shanghai Jiao Tong University, Shanghai 200240, P. R. China

E-mail: zhangsy16@sjtu.edu.cn

1. General information S2
2. General procedure for synthesis of 3-alkenamide substrates S2
3. Optimization of the Reaction Conditions S9
4. General procedure for Cu-catalyzed allylic C−H oxygenation reactions S13
5. Synthetic utilities S43
6. Primary kinetic isotope effects S48
7. General procedure for KIE experiments S51
8. Crystal structure information for compound **28** and **70** S52
9. Computational details S54
10. NMR spectra S56
11. References S140

1. General information

Reagents: All commercial materials were used as received from Energy Chemical or Adamas-beta, Alfa Aesar, TCI and Acros unless otherwise noted.

Chromatography: Thin layer chromatography (TLC) was carried out on silica gel 60 F254 pre-coated glass plates. Visualization was detected by irradiation with UV light (254 nm), or by treatment with a solution of phosphomolybdic acid in ethanol followed by heating. Flash chromatography was carried out on 200 – 300 mesh silica gel, eluting with a mixture of petroleum ether (b.p. 60 – 90 °C) and ethyl acetate.

NMR Spectroscope: ^{1}H NMR and ^{13}C NMR spectra were recorded on a Bruker AVANCE III HD 400 or 500 spectrometer, operating at 400 (or 500) MHz and 100 (or 125) MHz respectively. Chemical shifts (δ) were given in parts per million (ppm), and referenced relative to residual solvent $CHCl_3$ (7.26 ppm) in $CDCl_3$, or tetramethylsilane (0.00 ppm) as an internal standard for ^{1}H NMR spectra and deuterated solvent $CDCl_3$ (77.0 ppm) for ^{13}C NMR spectra. Coupling constants (*J*) were reported in hertz (Hz). The following abbreviations are used to indicate the multiplicity of the signals: s = singlet, d = doublet, t = triplet, q = quartet, m = multiplet, and associated combinations, e.g. dd = doublet of doublets.

Mass Spectrometry: High-resolution mass spectra (HRMS) were obtained on a Bruker Buker impact II using the electrospray ionization (ESI) technique.

2. Synthesis of 3-Alkenamide Substrates

2.1 Most of the alkenamide substrates are known compounds and were synthesized according to the literature reports[1-3].

2.2 General procedure for the preparation alkenamide substrates.

Preparation of AQ-enamide substrate-1 (intramolecular)

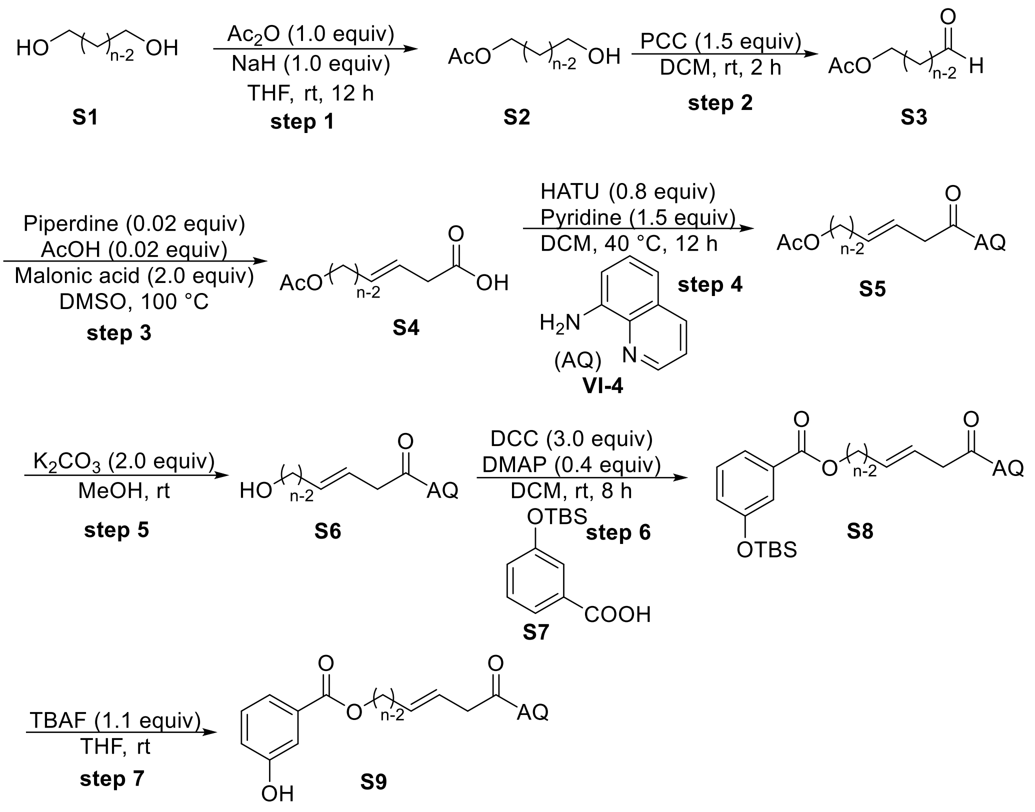

S2: NaH (1200 mg, 50.0 mmol) was added to a solution of 1,5 pentanediol **S1** (5210 mg, 50.0 mmol) in dry THF (300 mL). The suspension was refluxed and stirred under argon for 6 h, a solution of acetic anhydride (5100 mg, 50.0 mmol) in dry hexane (50 mL) was added dropwise and the mixture was stirred for 6 h, followed by carefully addition of water and extraction with EA (500 mL). The organic layer was washed with 5% Na_2CO_3 (2 × 250 mL) and brine (2 × 250 mL), dried ($MgSO_4$) and the solvent evaporated. The residue was purified by flash chromatography (PE/EA, 3:1) to afford the monoacetate **S2** as a colorless liquid.

S3: PCC (10.44 g, 48.0 mmol) was added to a solution of **S2** (4720 mg, 32.0 mmol) in DCM (250 mL) and the mixture was stirred for 4 h. After disappearance of starting material, the reaction mixture was diluted with Et_2O (100 mL), filtered off through a pad of celite and silica. After washing with Et_2O, the filtrate was concentrated under reduced pressure. The residue was purified by flash chromatography on silica gel (PE/EA = 5:1) to give **S3** as colorless liquid.

S4: To a solution of **S3** (2.82 g, 19.5 mmol) in DMSO (16 mL, 1.2 M) was added malonic acid (4.06 g, 39.0 mmol), piperidine (19.3 μL, 0.19 mmol) and AcOH (11.2

μL, 0.19 mmol) under argon atmosphere. The reaction mixture was stirred at room temperature for 20 min. After 20 min at room temperature, reaction temperature was elevated to 100 °C and stirred for 12 h. The reaction mixture was monitored by TLC using PE/EA = 1:1 as the mobile phase. After disappearance of starting material, the reaction mixture was quenched with distilled water and extracted with Et_2O (3 × 50 mL). The combined organic layer was dried over $MgSO_4$. After the removal of solvent, the residue was purified by flash chromatography on silica gel (PE/EA = 3:1 to 1:1) to give **S4** as pale yellow oil.

S5: To a solution of **S4** (3.36 g, 15.7 mmol) in DCM (45 mL, 0.35 M) was added 8-aminoquinoline (1.90 g, 13.0 mmol), pyridine (2.10 mL, 26.1 mmol) and HATU (5.96 g, 15.7 mmol). The reaction mixture was stirred at 40 °C for 12 h. The reaction mixture was monitored by TLC using PE/EA = 3:1 as the mobile phase. After disappearance of starting material, the reaction mixture was concentrated under reduced pressure. The residue was diluted with EA (100 mL) and washed with aqueous $NaHCO_3$ (3 × 100 mL) and brine (1 × 100 mL). The combined organic layer was dried over $MgSO_4$. After the removal of solvent, the residue was purified by flash chromatography on silica gel (PE/EA = 3:1) to give **S5** as colorless oil.

S6: To a solution of K_2CO_3 (2.76 g, 20.0 mmol, 2.0 equiv) in MeOH (20.0 mL) at room temperature was added compound **S5** (3.12 g, 10.0 mmol, 1.0 equiv). Then the mixture was stirred for 5 h at the same temperature. Once completion, the mixture was concentrated *in vacuo*. And the residue was purified by column chromatography using eluents (PE/EA = 1:1) to afford the product **S6** as yellow oil.

S8: To a solution of **S6** (2.7 g, 10.0 mmol) in DCM (50 mL, 0.5 M) was added DCC (6.18 g, 30.0 mmol), DMAP (488 mg, 4.0 mmol) and **S7** (5.04 g, 20.0 mmol). The reaction mixture was stirred at room temperature for 8 h. The reaction mixture was monitored by TLC using PE/EA = 4:1 as the mobile phase. After disappearance of starting material, the reaction mixture was quenched with distilled water and extracted with EA (3 × 50 mL). The combined organic layer was dried over $MgSO_4$. After the removal of solvent, the residue was purified by flash chromatography on silica gel (PE/EA = 6:1 to 4:1) to give **S8** as pale yellow oil.

S9: To a solution of TBAF (2.88 g, 11.0 mmol, 1.1 equiv) in THF (30.0 mL) at room temperature was added compound **S8** (5.05 g, 10.0 mmol, 1.0 equiv). Then the mixture was stirred for 6 h at the same temperature. Once completion, the mixture was concentrated *in vacuo*. And the residue was purified by column chromatography using eluents (PE/EA = 2:1) to afford the product **S9** as colorless oil.

Preparation of AQ-enamide substrate-2 (intramolecular)

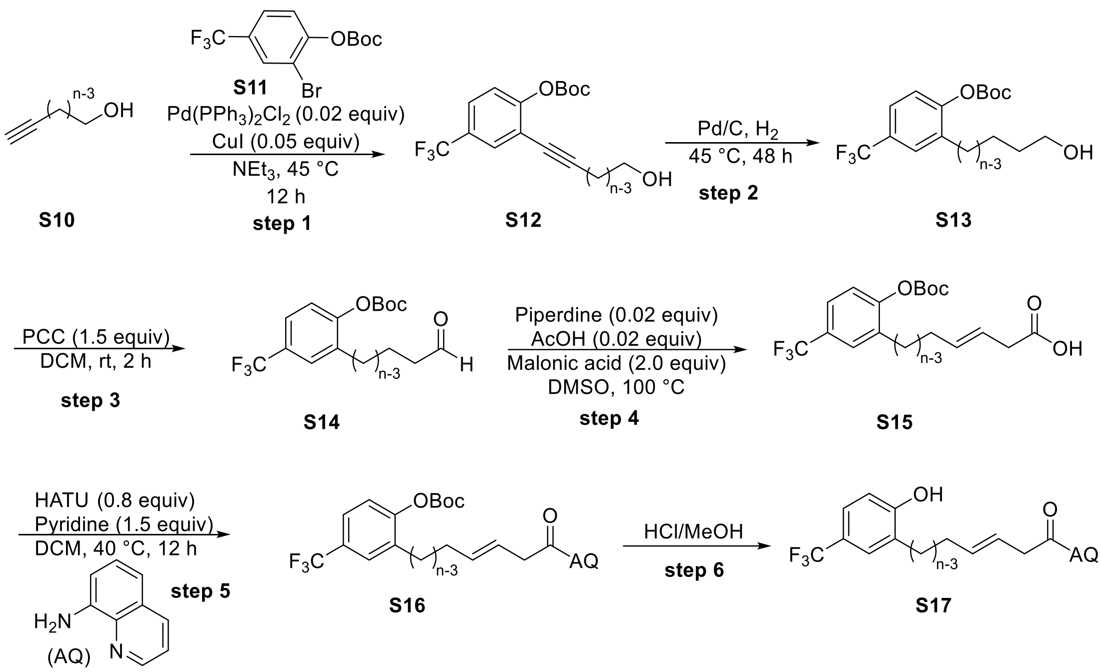

S12: A solution of aryl bromide **S11** (3.40 g, 10 mmol) and 4-pentyn-1-ol **S10**(8328 mg, 9.9 mmol) in Et_3N (20.0 mL) was degassed with argon via bubbling for 15 min. CuI (95.2 mg, 0.5 mmol) was added, followed by $PdCl_2(PPh_3)_2$ (141 mg, 0.2 mmol), and the mixture was stirred at 45 °C overnight. After 12 h, the reaction mixture filtered through a chromatographic column packed with silica gel, washing with EA (50 mL). The filtrate was concentrated via rotary evaporation, and the resulting residue was purified by flash chromatography on silica gel (PE/EA = 4:1 to 2:1) to give **S12** as colorless oil.

S13: A solution of **S12** (3.44 g, 10 mmol) in MeOH (20 ml) was stirred in the presence of 10%-Pd/C (344 mg) in hydrogen atmosphere at 45 °C for 48 h. After the reaction was complete, the mixture was filtered through celite and the filtrate was concentrated *in vacuo*. The resulting residue was purified by flash chromatography on

silica gel (PE/EA = 3:1 to 2:1) to give **S13** as yellow oil.

The synthetic procedures for **S14**, **S15**, and **S16** can be referenced from the aforementioned synthetic methods of **S3**, **S4**, and **S5**.

S17: To a solution of HCl (2 M in CH_3OH, 2.0 mL) in MeOH (10.0 mL) at room temperature was added compound **S16** (2.57 g, 5.0 mmol). Then the mixture was stirred for 5 h at the same temperature. Once completion, the mixture was concentrated *in vacuo*. And the residue was purified by column chromatography using eluents (PE/EA = 3:1) to afford the product **S17** as colorless oil.

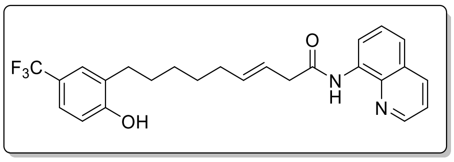

(*E*)-9-(2-hydroxy-5-(trifluoromethyl)phenyl)-*N*-(quinolin-8-yl)non-3-enamide :

^{1}H NMR (400 MHz, Chloroform-*d*) δ 10.1 (s, 1H), 8.9 – 8.7 (m, 2H), 8.2 (dd, *J* = 8.3, 1.7 Hz, 1H), 7.6 – 7.5 (m, 2H), 7.4 (dd, *J* = 8.3, 4.3 Hz, 1H), 7.3 (d, *J* = 2.3 Hz, 1H), 7.3 (dd, *J* = 8.3, 2.3 Hz, 1H), 7.1 (s, 1H), 6.9 (d, *J* = 8.3 Hz, 1H), 5.9 – 5.6 (m, 2H), 3.3 (d, *J* = 6.6 Hz, 2H), 2.6 (t, *J* = 7.6 Hz, 2H), 2.1 (q, *J* = 6.9 Hz, 2H), 1.7 (p, *J* = 7.5 Hz, 2H), 1.6 – 1.5 (m, 2H), 1.5 – 1.4 (m, 2H). **^{13}C NMR** (101 MHz, Chloroform-*d*) δ 171.0, 157.1, 148.3, 138.5, 137.1, 136.5, 134.1, 129.5, 128.0, 127.4, 127.3 (d, *J* = 3.6 Hz), 124.3 (d, *J* = 3.9 Hz), 122.0, 122.0, 121.7, 116.7, 115.2, 42.0, 32.5, 29.7, 29.4, 28.7, 28.5. **^{19}F NMR** (377 MHz, Chloroform-*d*) δ -61.2. **HRMS**: calculated for $C_{25}H_{26}F_3N_2O_2$ [M+H$^+$] 443.1941, found 443.1943.

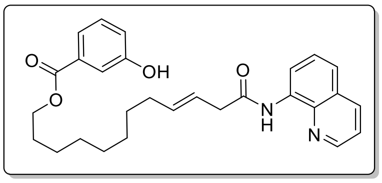

(*E*)-12-oxo-12-(quinolin-8-ylamino)dodec-9-en-1-yl 3-hydroxybenzoate :

^{1}H NMR (400 MHz, Chloroform-*d*) δ 10.1 (s, 1H), 8.8 – 8.7 (m, 2H), 8.2 (dd, *J* = 8.3, 1.7 Hz, 1H), 7.6 – 7.5 (m, 4H), 7.5 (dd, *J* = 8.3, 4.3 Hz, 1H), 7.3 (t, *J* = 7.9 Hz, 1H), 7.1 (ddd, *J* = 8.1, 2.7, 1.1 Hz, 1H), 6.6 (s, 1H), 5.8 (qt, *J* = 15.4, 6.6 Hz, 2H), 4.3 (t, *J*

= 6.5 Hz, 2H), 3.3 (d, *J* = 6.7 Hz, 2H), 2.1 (q, *J* = 6.9 Hz, 2H), 1.8 – 1.7 (m, 2H), 1.5 – 1.4 (m, 2H), 1.3 (dd, *J* = 19.6, 6.1 Hz, 8H). **^{13}C NMR** (101 MHz, Chloroform-*d*) δ 170.7, 166.6, 156.2, 148.2, 137.2, 136.5, 134.3, 131.9, 129.6, 128.0, 127.5, 122.1, 121.8, 121.7, 121.7, 120.2, 116.7, 116.3, 65.2, 42.2, 32.6, 29.2, 29.0, 28.9, 28.8, 28.6, 25.9. **HRMS**: calculated for $C_{28}H_{33}N_2O_4$ [M+H$^+$] 461.2435, found 461.2431.

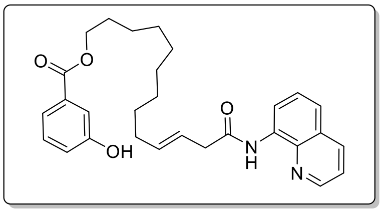

(*E*)-13-oxo-13-(quinolin-8-ylamino)tridec-10-en-1-yl 3-hydroxybenzoate:

^{1}H NMR (400 MHz, Chloroform-*d*) δ 10.1 (s, 1H), 8.8 – 8.7 (m, 2H), 8.1 (dd, *J* = 8.4, 1.7 Hz, 1H), 7.7 – 7.5 (m, 4H), 7.4 (dd, *J* = 8.2, 4.2 Hz, 1H), 7.3 – 7.2 (m, 1H), 7.1 (dd, *J* = 8.2, 2.5 Hz, 1H), 5.9 – 5.6 (m, 2H), 4.3 (t, *J* = 6.6 Hz, 2H), 3.3 (d, *J* = 6.8 Hz, 2H), 2.1 (q, *J* = 7.2 Hz, 2H), 1.7 (p, *J* = 6.7 Hz, 2H), 1.5 (q, *J* = 7.4 Hz, 2H), 1.4 – 1.2 (m, 10H). **^{13}C NMR** (101 MHz, Chloroform-*d*) δ 170.9, 166.9, 156.6, 148.2, 138.5, 137.6, 136.4, 134.1, 131.7, 129.5, 127.9, 127.4, 121.9, 121.8, 121.6, 121.3, 120.3, 116.6, 116.4, 65.2, 42.1, 32.7, 29.3, 29.1, 29.1, 29.1, 28.5, 25.9. **HRMS**: calculated for $C_{29}H_{35}N_2O_4$ [M+H$^+$] 475.2591, found 475.2596.

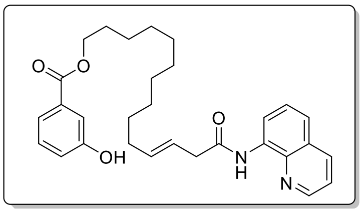

(*E*)-14-oxo-14-(quinolin-8-ylamino)tetradec-11-en-1-yl 3-hydroxybenzoate :

^{1}H NMR (400 MHz, Chloroform-*d*) δ 10.1 (s, 1H), 8.8 (ddt, *J* = 5.7, 3.9, 2.1 Hz, 2H), 8.2 (dd, *J* = 8.3, 1.6 Hz, 1H), 7.6 – 7.5 (m, 4H), 7.5 (dd, *J* = 8.2, 4.2 Hz, 1H), 7.3 (td, *J* = 7.9, 1.6 Hz, 1H), 7.1 – 7.0 (m, 1H), 5.9 – 5.6 (m, 2H), 4.3 (td, *J* = 6.6, 3.2 Hz, 2H), 3.3 (dd, *J* = 31.8, 7.0 Hz, 2H), 2.2 (dq, *J* = 14.3, 7.1 Hz, 2H), 1.8 – 1.7 (m, 2H), 1.4 (t, *J* = 6.3 Hz, 2H), 1.4 – 1.2 (m, 12H). **^{13}C NMR** (101 MHz, Chloroform-*d*) δ 166.6, 156.2, 148.3, 148.2, 137.5, 136.2, 131.9, 129.6, 128.0, 127.5, 122.0, 121.9, 121.8,

121.7, 121.7, 120.2, 116.4, 65.2, 42.2, 36.8, 32.7, 29.2, 29.1, 28.9, 28.6, 27.5, 26.0, 25.9. **HRMS**: calculated for $C_{30}H_{37}N_2O_4$ [M+H$^+$] 489.2748, found 489.2750.

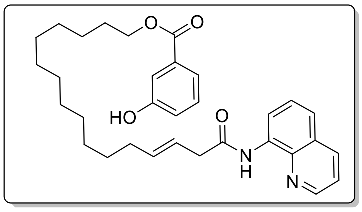

(*E*)-12-hydroxy-16-oxo-16-(quinolin-8-ylamino)hexadec-13-en-1-yl benzoate :

^{1}H NMR (400 MHz, Chloroform-*d*) δ 10.2 (s, 1H), 8.8 – 8.7 (m, 2H), 8.1 (dd, *J* = 8.3, 1.7 Hz, 1H), 7.6 (t, *J* = 2.1 Hz, 1H), 7.6 (d, *J* = 7.9 Hz, 1H), 7.5 – 7.5 (m, 2H), 7.4 (dd, *J* = 8.3, 4.2 Hz, 1H), 7.3 – 7.2 (m, 1H), 7.1 (dd, *J* = 8.2, 2.6 Hz, 1H), 5.9 – 5.6 (m, 2H), 4.3 (t, *J* = 6.6 Hz, 2H), 3.3 (d, *J* = 6.9 Hz, 2H), 2.1 (q, *J* = 7.1 Hz, 2H), 1.7 (p, *J* = 6.8 Hz, 2H), 1.5 (q, *J* = 7.6 Hz, 2H), 1.4 – 1.2 (m, 16H). **^{13}C NMR** (101 MHz, Chloroform-*d*) δ 170.9, 166.9, 156.7, 148.2, 138.5, 137.8, 136.3, 134.0, 131.6, 129.5, 127.9, 127.3, 121.9, 121.7, 121.6, 121.2, 120.3, 116.6, 116.4, 65.2, 42.0, 32.7, 29.5, 29.5, 29.5, 29.4, 29.2, 29.2, 28.6, 25.9. **HRMS**: calculated for $C_{32}H_{41}N_2O_4$ [M+H$^+$] 517.3061, found 517.3055.

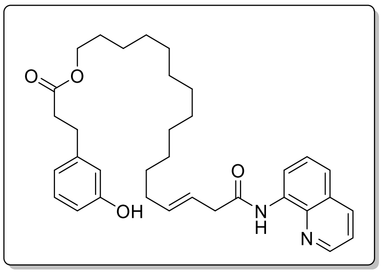

(*E*)-4-(16-oxo-2,15-dioxa-1(1,3)-benzenacyclooctadecaphane-3-yl)-*N*-(quinolin-8-yl)but-3-enamide:

^{1}H NMR (400 MHz, Chloroform-*d*) δ 10.1 (s, 1H), 8.9 – 8.6 (m, 2H), 8.2 (dd, *J* = 8.3, 1.7 Hz, 1H), 7.7 – 7.4 (m, 3H), 7.1 (td, *J* = 7.4, 1.4 Hz, 1H), 6.9 – 6.6 (m, 3H), 6.2 (s, 1H), 5.9 – 5.6 (m, 2H), 4.1 (t, *J* = 6.7 Hz, 2H), 3.3 (d, *J* = 6.8 Hz, 2H), 2.9 (t, *J* = 7.8 Hz, 2H), 2.7 – 2.4 (m, 2H), 2.1 (q, *J* = 7.1 Hz, 2H), 1.6 (t, *J* = 6.9 Hz, 2H), 1.5 (q, *J* = 7.6 Hz, 2H), 1.4 – 1.2 (m, 16H). **^{13}C NMR** (101 MHz, Chloroform-*d*) δ 173.2, 170.6, 156.2, 148.2, 142.3, 138.6, 137.5, 136.4, 134.3, 129.6, 128.0, 127.4, 122.0, 121.7,

121.6, 120.3, 116.6, 115.3, 113.3, 64.8, 42.1, 35.8, 32.7, 30.9, 29.6, 29.6, 29.5, 29.5, 29.4, 29.2, 29.2, 28.6, 25.9. **HRMS**: calculated for $C_{34}H_{45}N_2O_4$ $[M+H^+]$ 545.3374, found 545.3370.

3. Optimization of the Reaction Conditions

Table S1. Screening of the nucleophile substrate range[a,b]

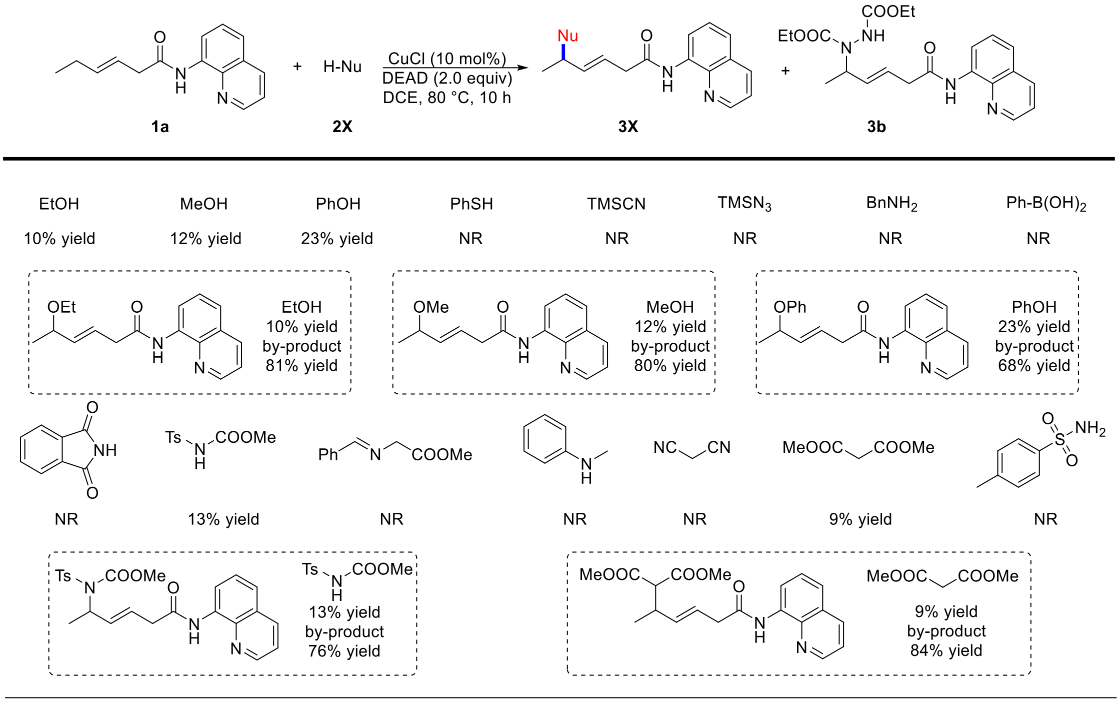

[a]Reaction conditions: **1a** (0.2 mmol, 1.0 equiv), **2X** (0.6 mmol, 3.0 equiv), DEAD (0.4 mmol, 2.0 equiv), CuCl (10 mol%) in DCE (2.0 mL) at the 80 °C for 10 h. [b]Isolated yield.

Table S2. Screening of Cu salts[a,b]

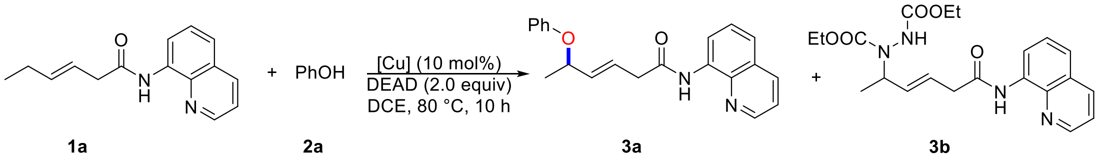

entry	catalyst	**3a**-yield%	**3b**-yield%
1	CuCl	23%	68%
2	CuI	21%	67%
3	CuTc	18%	71%
4	Cu_2O	30%	59%
5	$Cu(acac)_2$	13%	58%
6	$Cu(OTf)_2$	<10%	61%
7	$CuCl_2$	16%	56%

[a]Reaction conditions: **1a** (0.2 mmol, 1.0 equiv), **2a** (0.6 mmol, 3.0 equiv), DEAD (0.4 mmol, 2.0 equiv), catalyst (10 mol%) in DCE (2.0 mL) at the 80 °C for 10 h. [b]Isolated yield.

Table S3. Screening of solvents[a,b]

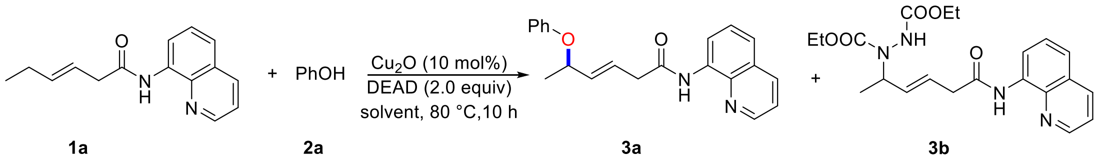

entry	solvent	**3a**-yield%	**3b**-yield%
1	Toluene	36%	45%
2	PhCl	32%	55%
3	Dioxane	21%	62%
4	DMF	15%	67%
5	THF	23%	51%
6	DMSO	14%	64%
7	EtOH	trace	trace

[a]Reaction conditions: **1a** (0.2 mmol, 1.0 equiv), **2a** (0.6 mmol, 3.0 equiv), DEAD (0.4 mmol, 2.0 equiv), Cu_2O (10 mol%) in solvent (2.0 mL) at the 80 °C for 10 h. [b]Isolated yield.

Table S4. Screening of ligand-1[a,b]

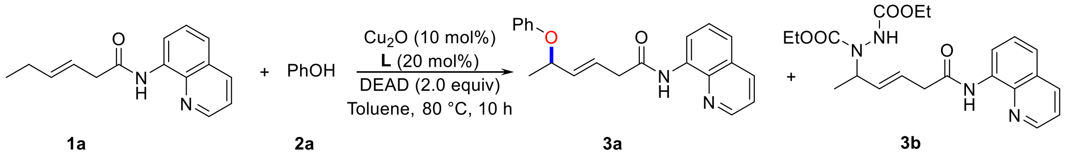

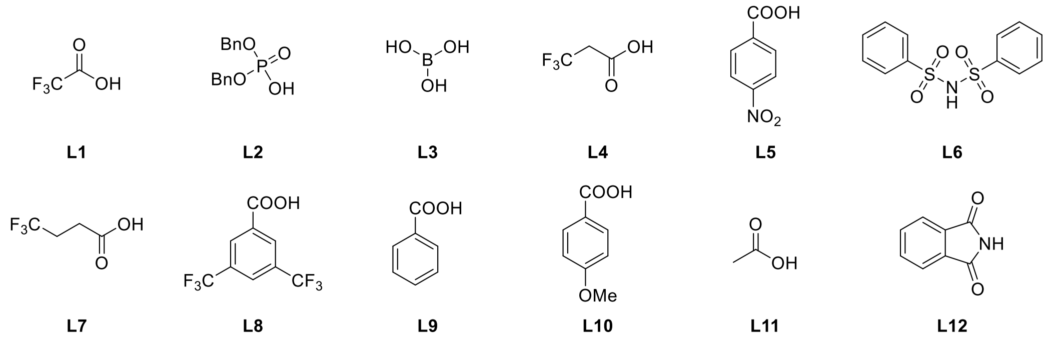

entry	ligand	PKa-(DMSO)	**3a**-yield%	**3b**-yield%
1	$\mathbf{L_1}$	5.11	NR	NR
2	$\mathbf{L_2}$	7.39	NR	NR
3	$\mathbf{L_3}$	7.84	18%	Trace
4	$\mathbf{L_4}$	8.08	34%	Trace
5	$\mathbf{L_5}$	8.60	46%	Trace
6	$\mathbf{L_6}$	8.82	68%	12%
7	$\mathbf{L_7}$	9.12	57%	16%
8	$\mathbf{L_8}$	9.82	61%	20%
9	$\mathbf{L_9}$	10.60	50%	36%
10	$\mathbf{L_{10}}$	11.38	41%	42%
11	$\mathbf{L_{11}}$	12.60	36%	43%
12	$\mathbf{L_{12}}$	13.40	34%	46%

[a]Reaction conditions: **1a** (0.2 mmol, 1.0 equiv), **2a** (0.6 mmol, 3.0 equiv), DEAD (0.4 mmol, 2.0 equiv), Cu_2O (10 mol%) and Ligand (20 mol%) in Toluene (2.0 mL) at the 80 °C for 10 h. [b]Isolated yield.

Table S5. Screening of ligand-2[a,b]

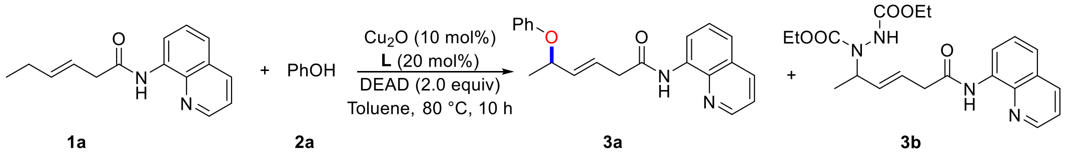

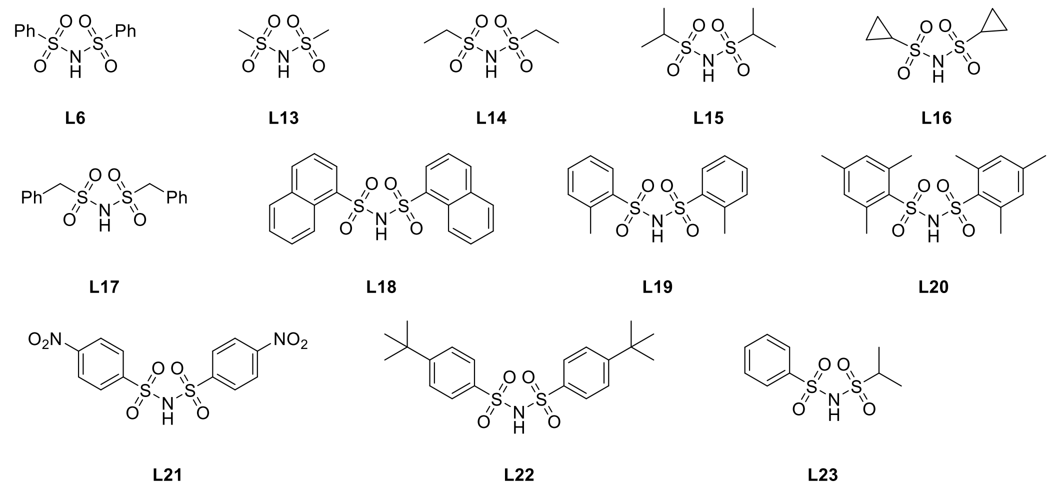

entry	ligand	**3a**-yield%	**3b**-yield%
1	**L6**	68%	12%
2	**L13**	67%	12%
3	**L14**	72%	Trace
4	**L15**	88%	Trace
5	**L16**	71%	Trace
6	**L17**	42%	Trace
7	**L18**	48%	16%
8	**L19**	45%	Trace
9	**L20**	NR	Trace
10	**L21**	56%	Trace
11	**L22**	58%	Trace
12	**L23**	70%	Trace

[a]Reaction conditions: **1a** (0.2 mmol, 1.0 equiv), **2a** (0.6 mmol, 3.0 equiv), DEAD (0.4 mmol, 2.0 equiv), Cu_2O (10 mol%) and Ligand (20 mol%) in Toluene (2.0 mL) at the 80 °C for 10 h. [b]Isolated yield.

Table S6. Substrates with poor reactivity[a,b]

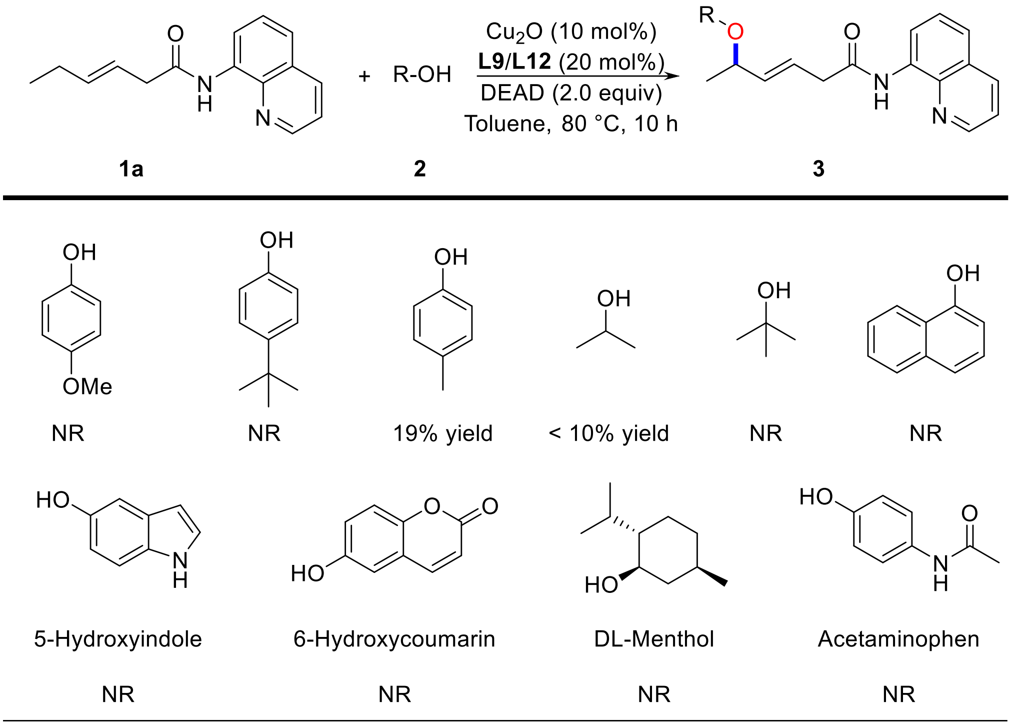

[a]Reaction conditions: **1a** (0.2 mmol, 1.0 equiv), **2** (0.6 mmol, 3.0 equiv), DEAD (0.4 mmol, 2.0 equiv), Cu_2O (10 mol%) and Ligand (20 mol%) in Toluene (2.0 mL) at the 80 °C for 10 h. [b]Isolated yield.

4. General procedure for Cu-catalyzed allylic C−H oxygenation reactions

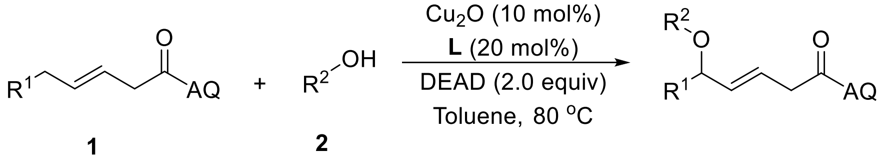

A mixture of amide (0.20 mmol, 1.0 equiv), Cu_2O (0.02 mmol, 0.1 equiv), **L** (0.04 mmol, 0.2 equiv), DEAD (0.40 mmol, 2.0 equiv) and alcohol/phenol (0.60 mmol, 3.0 equiv) in Toluene (2.0 mL) in a 10 mL glass vial (sealed with PTFE cap) was heated at 80 °C for indicated time. The reaction progress was monitored by thin layer chromatography. Upon completion, the reaction mixture was concentrated *in vacuo* and purified by silica gel column chromatography to afford the desired products.

(*E*)-5-phenoxy-*N*-(quinolin-8-yl)hex-3-enamide

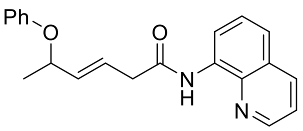

Compound **3a** was isolated in 88 % yield with Cu_2O for 10 h. Flash silica gel chromatography (PE : EA = 4:1). Colorless liquid. **^{1}H NMR** (400 MHz, Chloroform-*d*) δ 10.0 (s, 1H), 9.0 – 8.6 (m, 2H), 8.1 (dd, *J* = 8.3, 1.7 Hz, 1H), 7.7 – 7.3 (m, 3H), 7.3 – 7.1 (m, 2H), 7.1 – 6.7 (m, 3H), 6.3 – 5.5 (m, 2H), 4.9 (q, *J* = 6.2 Hz, 1H), 3.3 (d, *J* = 7.0 Hz, 2H), 1.6 (d, *J* = 6.4 Hz, 3H). **^{13}C NMR** (101 MHz, Chloroform-*d*) δ 169.1, 157.9, 148.2, 138.5, 137.0, 136.4, 134.3, 129.4, 127.9, 127.4, 124.1, 121.6, 121.6, 120.8, 116.4, 116.0, 73.9, 41.6, 21.5. **HRMS**: calculated for : $C_{21}H_{20}N_2O_2$ [M+Na$^+$] 355.1417, found 355.1414.

(*E*)-2,2-dimethyl-5-phenoxy-*N*-(quinolin-8-yl)hex-3-enamide

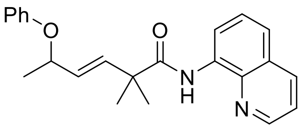

Compound **4** was isolated in 68 % yield with Cu_2O for 10 h. Flash silica gel chromatography (PE : EA = 4:1). Colorless liquid. **^{1}H NMR** (400 MHz, Chloroform-*d*) δ 10.2 (s, 1H), 8.8 – 8.6 (m, 2H), 8.1 (dd, *J* = 8.3, 1.7 Hz, 1H), 7.7 – 7.3 (m, 3H), 7.2 – 7.1 (m, 2H), 7.0 – 6.9 (m, 2H), 6.9 – 6.8 (m, 1H), 6.1 (dd, *J* = 16.0, 1.0 Hz, 1H), 6.0 (dd, *J* = 16.0, 5.9 Hz, 1H), 5.1 – 4.9 (m, 1H), 1.6 (d, *J* = 6.4 Hz, 3H), 1.5 (s, 6H). **^{13}C NMR** (101 MHz, Chloroform-*d*) δ 174.7, 158.0, 148.1, 138.8, 136.2, 135.9, 134.6, 132.0, 129.3, 127.9, 127.4, 121.5, 121.4, 120.8, 116.1, 116.1, 74.5, 46.0, 25.2, 25.1, 21.6. **HRMS**: calculated for : $C_{23}H_{24}N_2O_2$ [M+Na$^+$] 383.1730, found 383.1727.

(*E*)-2-methyl-5-phenoxy-*N*-(quinolin-8-yl)hex-3-enamide

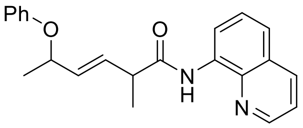

Compound **5** was isolated in 81 % yield with Cu_2O for 10 h. Flash silica gel chromatography (PE : EA = 4:1). Colorless liquid. **^{1}H NMR** (400 MHz, Chloroform-*d*) δ 10.1 (d, *J* = 18.1 Hz, 1H), 9.0 – 8.5 (m, 2H), 8.3 – 7.9 (m, 1H), 7.8 – 7.3 (m, 3H), 7.2 – 7.1 (m, 2H), 7.0 – 6.7 (m, 3H), 6.2 – 5.7 (m, 2H), 5.3 – 4.6 (m, 1H), 3.7 – 3.1 (m, 1H), 1.5 (t, *J* = 6.1 Hz, 3H), 1.4 (dd, *J* = 7.0, 1.4 Hz, 3H). **^{13}C NMR** (101 MHz, Chloroform-*d*) δ 172.4, 157.9, 148.1, 138.6, 136.3, 134.5, 134.1, 131.3, 130.9, 129.3, 127.9, 127.4, 121.5, 120.7, 116.1, 73.9, 45.6, 21.4, 17.1. **HRMS**: calculated for : $C_{22}H_{22}N_2O_2$ [M+Na$^+$] 369.1573, found 369.1567.

(*E*)-5-phenoxy-*N*-(quinolin-8-yl)oct-3-enamide

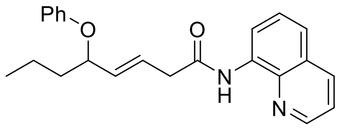

Compound **6** was isolated in 83 % yield with Cu_2O for 10 h. Flash silica gel chromatography (PE : EA = 4:1). Colorless liquid. **^{1}H NMR** (400 MHz, Chloroform-*d*) δ 10.0 (s, 1H), 8.9 – 8.6 (m, 2H), 8.1 (dd, *J* = 8.3, 1.7 Hz, 1H), 7.6 – 7.5 (m, 2H), 7.4 (dd, *J* = 8.3, 4.2 Hz, 1H), 7.2 – 7.1 (m, 2H), 7.0 – 6.9 (m, 2H), 6.9 – 6.8 (m, 1H), 6.1 – 6.0 (m, 1H), 5.9 – 5.8 (m, 1H), 4.7 (q, *J* = 6.3 Hz, 1H), 3.3 (dd, *J* = 7.1, 1.2 Hz, 2H), 2.0 – 1.8 (m, 1H), 1.8 – 1.7 (m, 1H), 1.7 – 1.4 (m, 2H), 1.0 (t, *J* = 7.4 Hz, 3H). **^{13}C NMR** (101 MHz, Chloroform-*d*) δ 169.2, 158.5, 148.2, 138.5, 136.4, 136.1, 134.4, 129.4, 128.0, 127.4, 124.6, 121.7, 121.7, 120.8, 116.5, 116.1, 78.1, 41.8, 38.0, 18.8, 14.1. **HRMS**: calculated for : $C_{23}H_{24}N_2O_2$ [M+Na$^+$] 383.1730, found 383.1727.

(*E*)-5-phenoxy-*N*-(quinolin-8-yl)tetradec-3-enamide

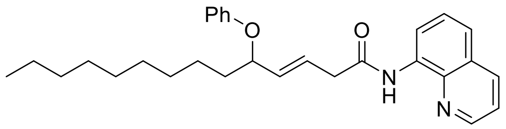

Compound **7** was isolated in 61 % yield with Cu_2O for 10 h. Flash silica gel chromatography (PE : EA = 4:1). Colorless liquid. **^{1}H NMR** (400 MHz, Chloroform-*d*) δ 10.0 (s, 1H), 9.1 – 8.5 (m, 2H), 8.1 (dd, *J* = 8.3, 1.7 Hz, 1H), 7.6 – 7.5 (m, 2H), 7.4 (dd, *J* = 8.3, 4.2 Hz, 1H), 7.2 – 7.1 (m, 2H), 7.0 – 6.9 (m, 2H), 6.9 – 6.8 (m, 1H), 6.1 – 6.0 (m, 1H), 5.9 – 5.7 (m, 1H), 4.7 (q, *J* = 6.3 Hz, 1H), 3.7 – 2.9 (m, 2H), 2.1 – 1.6 (m, 2H), 1.6 – 1.4 (m, 2H), 1.4 – 1.1 (m, 12H), 0.9 (t, *J* = 6.9 Hz, 3H). **^{13}C NMR** (101 MHz, Chloroform-*d*) δ 169.2, 158.4, 148.1, 138.5, 136.4, 136.1, 134.3, 129.4, 127.9, 127.4, 124.5, 121.6, 121.6, 120.7, 116.5, 116.0, 78.3, 41.7, 35.9, 31.9, 29.6, 29.6, 29.6, 29.3, 25.5, 22.7, 14.1. **HRMS**: calculated for : $C_{29}H_{36}N_2O_2$ [M+Na$^+$] 467.2669, found 467.2670.

2-(3-phenoxycyclohex-1-en-1-yl)-*N*-(quinolin-8-yl)acetamide

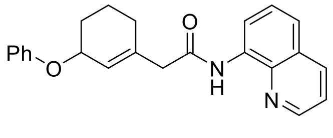

Compound **8** was isolated in 28 % yield with Cu_2O for 10 h. Flash silica gel chromatography (PE : EA = 4:1). Colorless liquid. **^{1}H NMR** (400 MHz, Chloroform-*d*) δ 10.2 (s, 1H), 8.8 (dd, *J* = 4.2, 1.7 Hz, 1H), 8.7 (dd, *J* = 7.0, 2.1 Hz, 1H), 8.2 (dd, *J* = 8.3, 1.7 Hz, 1H), 7.6 – 7.5 (m, 2H), 7.5 (dd, *J* = 8.3, 4.2 Hz, 1H), 7.3 – 7.3 (m, 2H), 7.0 – 6.9 (m, 3H), 6.0 (dd, *J* = 3.2, 1.8 Hz, 1H), 5.0 (d, *J* = 4.7 Hz, 1H), 3.3 (q, *J* = 15.2 Hz, 2H), 2.3 – 2.1 (m, 2H), 2.1 – 1.9 (m, 3H), 1.8 – 1.7 (m, 1H). **^{13}C NMR** (101

MHz, Chloroform-*d*) δ 168.9, 157.9, 148.3, 137.9, 136.4, 134.4, 129.6, 128.0, 127.4, 126.7, 121.7, 121.6, 120.8, 116.4, 116.0, 71.4, 47.5, 28.8, 28.0, 19.3. **HRMS**: calculated for : $C_{23}H_{22}N_2O_2$ [M+Na$^+$] 381.1573, found 381.1573.

(*E*)-5-phenoxy-6-phenyl-*N*-(quinolin-8-yl)hex-3-enamide

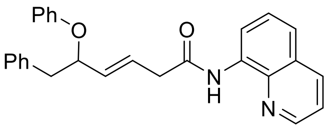

Compound **9** was isolated in 78 % yield with Cu_2O for 10 h. Flash silica gel chromatography (PE : EA = 4:1). Colorless liquid. **^{1}H NMR** (400 MHz, Chloroform-*d*) δ 10.0 (s, 1H), 9.1 – 8.6 (m, 2H), 8.1 (dd, *J* = 8.3, 1.7 Hz, 1H), 7.6 – 7.5 (m, 2H), 7.4 (dd, *J* = 8.3, 4.2 Hz, 1H), 7.3 – 7.3 (m, 2H), 7.3 – 7.2 (m, 2H), 7.2 – 7.1 (m, 3H), 7.0 – 6.9 (m, 2H), 6.9 – 6.8 (m, 1H), 6.1 – 5.9 (m, 1H), 5.9 – 5.8 (m, 1H), 4.9 (q, *J* = 6.2 Hz, 1H), 3.4 – 3.2 (m, 3H), 3.1 (dd, *J* = 13.8, 5.6 Hz, 1H). **^{13}C NMR** (101 MHz, Chloroform-*d*) δ 168.9, 158.1, 148.1, 138.4, 137.6, 136.3, 135.1, 134.3, 129.7, 129.3, 128.3, 127.9, 127.4, 126.4, 124.9, 121.6, 121.6, 120.9, 116.4, 116.1, 79.0, 42.4, 41.7. **HRMS**: calculated for : $C_{27}H_{24}N_2O_2$ [M+Na$^+$] 431.1730, found 431.1726.

(*E*)-7-(benzyloxy)-5-phenoxy-*N*-(quinolin-8-yl)hept-3-enamide

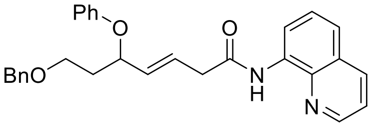

Compound **10** was isolated in 86 % yield with Cu_2O for 10 h. Flash silica gel chromatography (PE : EA = 4:1). Colorless liquid. **^{1}H NMR** (400 MHz, Chloroform-*d*) δ 10.0 (s, 1H), 9.0 – 8.4 (m, 2H), 8.1 (dd, *J* = 8.3, 1.6 Hz, 1H), 7.6 – 7.4 (m, 2H), 7.4 (dd, *J* = 8.3, 4.2 Hz, 1H), 7.3 – 7.1 (m, 7H), 7.0 – 6.9 (m, 2H), 6.9 – 6.8 (m, 1H), 6.1 – 6.0 (m, 1H), 5.9 (dd, *J* = 15.7, 6.1 Hz, 1H), 5.1 – 4.8 (m, 1H), 4.5 – 4.4 (m, 2H), 3.8 – 3.6 (m, 2H), 3.3 (d, *J* = 7.1 Hz, 2H), 2.4 – 2.0 (m, 2H). **^{13}C NMR** (101 MHz, Chloroform-*d*) δ 168.9, 158.3, 148.1, 138.4, 138.3, 136.3, 135.7, 134.3, 129.3, 128.3, 127.9, 127.6, 127.5, 127.3, 124.8, 121.6, 121.6, 120.8, 116.4, 116.0, 75.0, 73.0, 66.3, 41.7, 36.2. **HRMS**: calculated for : $C_{29}H_{28}N_2O_3$ [M+Na$^+$] 475.1992, found 475.1985.

Methyl (*E*)-8-oxo-4-phenoxy-8-(quinolin-8-ylamino)oct-5-enoate

Compound **11** was isolated in 71 % yield with Cu_2O for 10 h. Flash silica gel

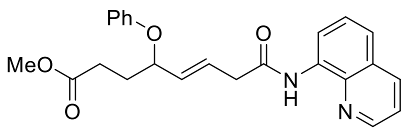

chromatography (PE : EA = 3:1). Colorless liquid. **^{1}H NMR** (400 MHz, Chloroform-*d*) δ 10.0 (s, 1H), 9.1 – 8.5 (m, 2H), 8.1 (dd, *J* = 8.3, 1.7 Hz, 1H), 7.7 – 7.4 (m, 3H), 7.2 – 7.1 (m, 2H), 7.1 – 6.8 (m, 3H), 6.2 – 6.0 (m, 1H), 5.9 (dd, *J* = 15.7, 6.0 Hz, 1H), 4.8 (q, *J* = 6.2 Hz, 1H), 3.7 (s, 3H), 3.3 (d, *J* = 7.1 Hz, 2H), 2.8 – 2.4 (m, 2H), 2.3 – 2.1 (m, 2H). **^{13}C NMR** (101 MHz, Chloroform-*d*) δ 173.7, 168.8, 158.0, 148.3, 138.4, 136.3, 135.1, 134.2, 129.4, 127.9, 127.3, 125.4, 121.7, 121.6, 121.0, 116.4, 116.0, 76.9, 51.6, 41.6, 30.9, 29.9. **HRMS**: calculated for : $C_{24}H_{24}N_2O_4$ [M+Na$^+$] 427.1628, found 427.1626.

(*E*)-8-azido-5-phenoxy-*N*-(quinolin-8-yl)oct-3-enamide

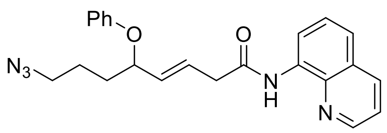

Compound **12** was isolated in 65 % yield with Cu_2O for 10 h. Flash silica gel chromatography (PE : EA = 4:1). Colorless liquid. **^{1}H NMR** (400 MHz, Chloroform-*d*) δ 9.9 (s, 1H), 9.0 – 8.6 (m, 2H), 8.2 (dd, *J* = 8.3, 1.7 Hz, 1H), 7.6 – 7.5 (m, 2H), 7.5 (dd, *J* = 8.3, 4.2 Hz, 1H), 7.2 – 7.1 (m, 2H), 7.0 – 6.9 (m, 2H), 6.9 – 6.8 (m, 1H), 6.2 – 6.0 (m, 1H), 5.9 – 5.7 (m, 1H), 4.8 (q, *J* = 6.0 Hz, 1H), 3.6 – 3.3 (m, 4H), 2.2 – 1.7 (m, 4H). **^{13}C NMR** (101 MHz, Chloroform-*d*) δ 168.9, 158.1, 148.2, 138.5, 136.4, 135.3, 134.3, 129.5, 128.0, 127.4, 125.3, 121.7, 121.7, 121.0, 116.5, 116.0, 77.6, 51.4, 41.6, 33.0, 25.0. **HRMS**: calculated for : $C_{23}H_{23}N_5O_2$ [M+Na$^+$] 424.1744, found 424.1740.

(*E*)-8-chloro-5-phenoxy-*N*-(quinolin-8-yl)oct-3-enamide

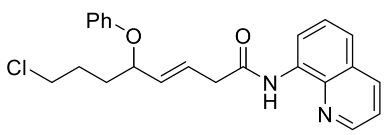

Compound **13** was isolated in 76 % yield with Cu_2O for 10 h. Flash silica gel chromatography (PE : EA = 4:1). Colorless liquid. **^{1}H NMR** (400 MHz, Chloroform-*d*) δ 9.9 (s, 1H), 8.9 – 8.6 (m, 2H), 8.1 (dd, *J* = 8.2, 1.7 Hz, 1H), 7.6 – 7.4 (m, 3H), 7.2 – 7.1 (m, 2H), 7.0 – 6.7 (m, 3H), 6.2 – 6.0 (m, 1H), 5.9 – 5.7 (m, 1H), 4.8 (q, *J* = 5.9 Hz, 1H), 3.8 – 3.5 (m, 2H), 3.3 (dd, *J* = 7.1, 1.2 Hz, 2H), 2.2 – 1.9 (m, 4H). **^{13}C NMR** (101 MHz, Chloroform-*d*) δ 168.9, 158.1, 148.2, 138.4, 136.3, 135.2, 134.2, 129.4, 127.9, 127.3, 125.2, 121.6, 121.6, 120.9, 116.4, 115.9, 77.3, 45.0, 41.6, 33.1, 28.5. **HRMS**: calculated for : $C_{23}H_{23}ClN_2O_2$ [M+Na$^+$] 417.1340, found 417.1334.

(*E*)-8-iodo-5-phenoxy-*N*-(quinolin-8-yl)oct-3-enamide

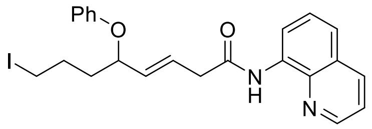

Compound **14** was isolated in 78 % yield with Cu_2O for 10 h. Flash silica gel chromatography (PE : EA = 4:1). Yellow liquid. **^{1}H NMR** (400 MHz, Chloroform-*d*) δ 9.9 (s, 1H), 9.1 – 8.5 (m, 2H), 8.1 (dd, *J* = 8.3, 1.7 Hz, 1H), 7.7 – 7.3 (m, 3H), 7.2 – 7.1 (m, 2H), 7.0 – 6.8 (m, 3H), 6.1 – 6.0 (m, 1H), 5.9 – 5.8 (m, 1H), 4.7 (q, *J* = 5.9 Hz, 1H), 3.3 (dd, *J* = 6.9, 1.2 Hz, 2H), 3.3 – 3.1 (m, 2H), 2.2 – 1.8 (m, 4H). **^{13}C NMR** (101 MHz, Chloroform-*d*) δ 168.9, 158.1, 148.2, 138.4, 136.4, 135.1, 134.3, 129.4, 127.9, 127.4, 125.2, 121.7, 121.6, 120.9, 116.4, 115.9, 77.1, 41.6, 36.6, 29.4, 6.6. **HRMS**: calculated for : $C_{23}H_{23}IN_2O_2$ [M+Na$^+$] 509.0696, found 509.0690.

(*E*)-5-phenoxy-*N*-(quinolin-8-yl)non-3-en-8-ynamide

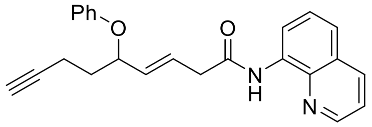

Compound **15** was isolated in 77 % yield with Cu_2O for 10 h. Flash silica gel chromatography (PE : EA = 4:1). Colorless liquid. **^{1}H NMR** (400 MHz, Chloroform-*d*) δ 10.0 (s, 1H), 8.8 – 8.7 (m, 2H), 8.2 (dd, *J* = 8.3, 1.7 Hz, 1H), 7.6 – 7.5 (m, 2H), 7.5 – 7.4 (m, 1H), 7.2 – 7.2 (m, 2H), 7.0 – 6.9 (m, 3H), 6.1 – 6.0 (m, 1H), 6.0 – 5.8 (m, 1H), 5.0 – 4.8 (m, 1H), 3.3 (dd, *J* = 7.1, 1.2 Hz, 2H), 2.5 – 2.4 (m, 2H), 2.2 – 2.0 (m, 2H), 2.0 – 2.0 (m, 1H). **^{13}C NMR** (101 MHz, Chloroform-*d*) δ 168.9, 158.1, 148.3, 138.5, 136.4, 135.2, 134.3, 129.4, 127.9, 127.4, 125.4, 121.7, 121.6, 121.0, 116.5, 116.1, 83.7, 76.6, 69.0, 41.7, 34.6, 14.8. **HRMS**: calculated for : $C_{24}H_{22}N_2O_2$ [M+Na$^+$] 393.1573, found 393.1568.

(*E*)-5-phenoxy-*N*-(quinolin-8-yl)trideca-3,12-dienamide

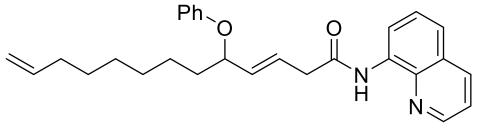

Compound **16** was isolated in 87 % yield with Cu_2O for 10 h. Flash silica gel chromatography (PE : EA = 3:1). Colorless liquid. **^{1}H NMR** (400 MHz, Chloroform-*d*) δ 10.0 (s, 1H), 9.0 – 8.5 (m, 2H), 8.1 (dd, *J* = 8.3, 1.7 Hz, 1H), 7.6 – 7.5 (m, 2H), 7.4 (dd, *J* = 8.3, 4.2 Hz, 1H), 7.2 – 7.1 (m, 2H), 7.0 – 6.9 (m, 2H), 6.9 (t, *J* = 7.3 Hz, 1H), 6.1 – 6.0 (m, 1H), 5.9 – 5.7 (m, 2H), 5.0 – 4.9 (m, 2H), 4.7 (q, *J* = 6.3 Hz, 1H), 3.3 (d, *J* = 7.0 Hz, 2H), 2.0 (q, *J* = 6.9 Hz, 2H), 1.9 – 1.8 (m,

1H), 1.8 – 1.7 (m, 1H), 1.6 – 1.4 (m, 2H), 1.4 – 1.2 (m, 6H). **^{13}C NMR** (101 MHz, Chloroform-*d*) δ 169.1, 158.4, 148.1, 139.1, 138.5, 136.3, 136.0, 134.3, 129.3, 127.9, 127.4, 124.6, 121.6, 121.6, 120.7, 116.4, 116.0, 114.2, 78.2, 41.7, 35.8, 33.7, 29.4, 29.0, 28.8, 25.4. **HRMS**: calculated for : $C_{28}H_{32}N_2O_2$ [M+Na$^+$] 451.2356, found 451.2356.

(*E*)-5-phenoxy-*N*-(quinolin-8-yl)-8-(2,2,2-trifluoroacetamido)oct-3-enamide

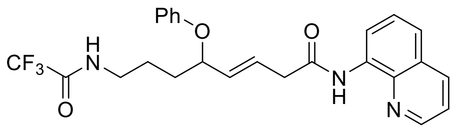

Compound **17** was isolated in 52 % yield with Cu_2O for 10 h. Flash silica gel chromatography (PE : EA = 2:1). White solid. **^{1}H NMR** (400 MHz, Chloroform-*d*) δ 9.9 (s, 1H), 9.1 – 8.5 (m, 2H), 8.2 (dd, *J* = 8.2, 1.7 Hz, 1H), 7.6 – 7.5 (m, 2H), 7.4 (dd, *J* = 8.3, 4.2 Hz, 1H), 7.2 – 7.1 (m, 2H), 7.1 – 6.8 (m, 4H), 6.1 – 5.9 (m, 1H), 5.9 – 5.7 (m, 1H), 4.8 (q, *J* = 6.2, 5.8 Hz, 1H), 3.7 – 3.3 (m, 2H), 3.3 – 3.2 (m, 2H), 2.1 – 1.6 (m, 4H). **^{13}C NMR** (101 MHz, Chloroform-*d*) δ 169.0, 157.8, 157.3 (q, *J* = 45.0 Hz), 148.2, 138.4, 136.4, 134.9, 134.2, 129.5, 128.0, 127.4, 125.4, 121.8, 121.7, 121.1, 116.5, 116.0, 115.9 (q, *J* = 357.5 Hz), 77.5, 41.4, 39.7, 32.4, 24.7. **^{19}F NMR** (376 MHz, Chloroform-*d*) δ -75.8. **HRMS**: calculated for : $C_{25}H_{24}F_3N_3O_3$ [M+Na$^+$] 494.1662, found 494.1658.

(*E*)-5-(3-bromophenoxy)-*N*-(quinolin-8-yl)hex-3-enamide

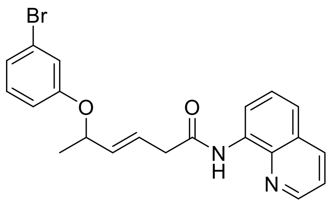

Compound **18** was isolated in 78 % yield with Cu_2O for 10 h. Flash silica gel chromatography (PE : EA = 4:1). Colorless liquid. **^{1}H NMR** (400 MHz, Chloroform-*d*) δ 10.0 (s, 1H), 9.0 – 8.6 (m, 2H), 8.2 (dd, *J* = 8.2, 1.7 Hz, 1H), 7.6 – 7.5 (m, 3H), 7.5 – 7.4 (m, 1H), 7.2 – 7.1 (m, 1H), 7.0 (dd, *J* = 8.2, 1.4 Hz, 1H), 6.8 – 6.7 (m, 1H), 6.2 – 6.0 (m, 1H), 6.0 – 5.8 (m, 1H), 4.9 (p, *J* = 6.3 Hz, 1H), 3.3 (d, *J* = 6.9 Hz, 2H), 1.6 (d, *J* = 6.3 Hz, 3H). **^{13}C NMR** (101 MHz, Chloroform-*d*) δ 169.0, 154.5, 148.2, 138.5, 136.4, 136.3, 134.3, 133.4, 128.2, 127.9, 127.4, 124.7, 122.2, 121.7, 121.6, 116.4, 116.3, 113.6, 76.0, 41.6, 21.5. **HRMS**: calculated for : $C_{21}H_{19}BrN_2O_2$ [M+Na$^+$] 433.0522, found 433.0520.

(*E*)-5-(4-nitrophenoxy)-*N*-(quinolin-8-yl)hex-3-enamide

Compound **19** was isolated in 94 % yield with Cu_2O for 10 h. Flash silica gel

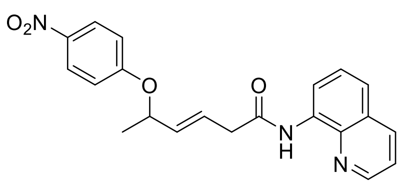

chromatography (PE : EA = 3:1). Colorless liquid. **^{1}H NMR** (400 MHz, Chloroform-*d*) δ 9.9 (s, 1H), 9.0 – 8.5 (m, 2H), 8.2 – 8.1 (m, 1H), 8.1 – 8.0 (m, 2H), 7.6 – 7.5 (m, 2H), 7.4 (dd, *J* = 8.3, 4.2 Hz, 1H), 7.1 – 6.9 (m, 2H), 6.2 – 6.0 (m, 1H), 6.0 – 5.8 (m, 1H), 5.1 – 4.9 (m, 1H), 3.6 – 3.2 (m, 2H), 1.6 (d, *J* = 6.4 Hz, 3H). **^{13}C NMR** (101 MHz, Chloroform-*d*) δ 168.7, 163.1, 148.1, 141.3, 138.3, 136.5, 135.1, 134.1, 128.0, 127.4, 125.8, 125.5, 121.8, 121.7, 116.4, 115.6, 74.9, 41.4, 21.4. **HRMS**: calculated for : $C_{21}H_{19}N_3O_4$ [M+Na$^+$] 400.1268, found 400.1268.

(*E*)-*N*-(quinolin-8-yl)-5-(4-(trifluoromethyl)phenoxy)hex-3-enamide

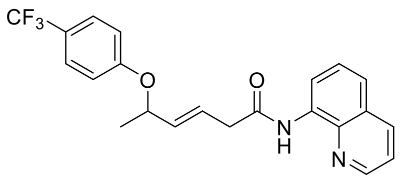

Compound **20** was isolated in 94 % yield with Cu_2O for 10 h. Flash silica gel chromatography (PE : EA = 4:1). Colorless liquid. **^{1}H NMR** (400 MHz, Chloroform-*d*) δ 10.0 (s, 1H), 8.9 – 8.4 (m, 2H), 8.1 (dd, *J* = 8.3, 1.7 Hz, 1H), 7.6 – 7.4 (m, 5H), 7.1 – 6.8 (m, 2H), 6.2 – 6.0 (m, 1H), 6.0 – 5.7 (m, 1H), 5.2 – 4.7 (m, 1H), 3.3 (dd, *J* = 7.1, 1.1 Hz, 2H), 1.6 (d, *J* = 6.4 Hz, 3H). **^{13}C NMR** (101 MHz, Chloroform-*d*) δ 168.8, 160.4, 148.1, 138.4, 136.4, 136.0, 134.2, 127.9, 127.4, 126.8 (q, *J* = 3.8 Hz), 124.8, 122.7 (q, *J* = 32.6 Hz), 121.7, 121.6, 116.4, 115.7, 74.2, 41.5, 21.4. **^{19}F NMR** (376 MHz, Chloroform-*d*) δ -61.5. **HRMS**: calculated for : $C_{22}H_{19}F_3N_2O_2$ [M+Na$^+$] 423.1291, found 423.1290.

Methyl (*E*)-4-((6-oxo-6-(quinolin-8-ylamino)hex-3-en-2-yl)oxy)benzoate

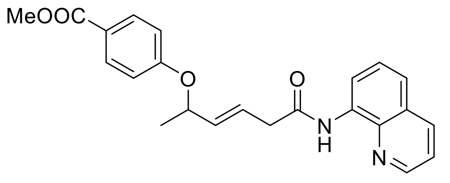

Compound **21** was isolated in 90 % yield with Cu_2O for 10 h. Flash silica gel chromatography (PE : EA = 4:1). Colorless liquid. **^{1}H NMR** (400 MHz, Chloroform-*d*) δ 10.0 (s, 1H), 8.9 – 8.5 (m, 2H), 8.2 (dd, *J* = 8.3, 1.7 Hz, 1H), 8.0 – 7.8 (m, 2H), 7.6 – 7.5 (m, 2H), 7.4 (dd, *J* = 8.3, 4.2 Hz, 1H), 7.0 – 6.9 (m, 2H), 6.1 – 6.0 (m, 1H), 6.0 – 5.8 (m, 1H), 5.0 (p, *J* = 6.3 Hz, 1H), 3.9 (s, 3H), 3.5 – 3.1 (m, 2H), 1.6 (d, *J* = 6.4 Hz, 3H). **^{13}C NMR** (101 MHz, Chloroform-*d*) δ 168.9, 166.8, 161.8, 148.2, 138.4, 136.4, 136.1, 134.3, 131.6, 128.0, 127.4, 124.8, 122.5, 121.7, 121.7, 116.5, 115.3, 74.1, 51.8, 41.6, 21.4. **HRMS**: calculated for : $C_{23}H_{22}N_2O_4$ [M+Na$^+$] 413.1472, found 413.1470.

(*E*)-5-(4-formylphenoxy)-*N*-(quinolin-8-yl)hex-3-enamide

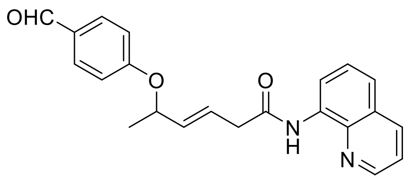

Compound **22** was isolated in 88 % yield with Cu_2O for 10 h. Flash silica gel chromatography (PE : EA = 4:1). Colorless liquid. **^{1}H NMR** (400 MHz, Chloroform-*d*) δ 9.9 (s, 1H), 9.8 (s, 1H), 9.1 – 8.5 (m, 2H), 8.1 (dd, *J* = 8.3, 1.7 Hz, 1H), 7.9 – 7.6 (m, 2H), 7.6 – 7.5 (m, 2H), 7.4 (dd, *J* = 8.3, 4.2 Hz, 1H), 7.1 – 6.9 (m, 2H), 6.3 – 6.0 (m, 1H), 6.0 – 5.7 (m, 1H), 5.2 – 4.8 (m, 1H), 3.4 – 3.1 (m, 2H), 1.6 (d, *J* = 6.3 Hz, 3H). **^{13}C NMR** (101 MHz, Chloroform-*d*) δ 190.7, 168.7, 163.0, 148.1, 138.3, 136.4, 135.6, 134.2, 131.9, 129.7, 127.9, 127.3, 125.0, 121.7, 121.6, 116.4, 115.9, 74.3, 41.5, 21.4. **HRMS**: calculated for : $C_{22}H_{20}N_2O_3$ [M+Na$^+$] 383.1366, found 383.1359.

(*E*)-5-(4-benzoylphenoxy)-*N*-(quinolin-8-yl)hex-3-enamide

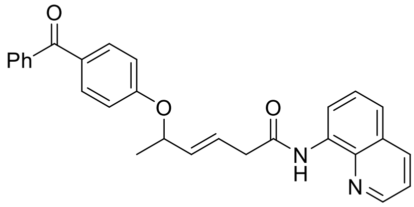

Compound **23** was isolated in 83 % yield with Cu_2O for 10 h. Flash silica gel chromatography (PE : EA = 4:1). Colorless liquid. **^{1}H NMR** (400 MHz, Chloroform-*d*) δ 9.9 (s, 1H), 8.9 – 8.6 (m, 2H), 8.2 (dd, *J* = 8.3, 1.7 Hz, 1H), 7.7 – 7.4 (m, 5H), 7.0 – 6.8 (m, 2H), 6.2 – 6.0 (m, 1H), 5.9 – 5.8 (m, 1H), 5.3 – 4.7 (m, 1H), 3.3 (dd, *J* = 7.1, 1.2 Hz, 2H), 1.6 (d, *J* = 6.4 Hz, 3H). **^{13}C NMR** (101 MHz, Chloroform-*d*) δ 195.5, 168.9, 161.7, 148.1, 138.4, 138.2, 136.4, 136.0, 134.2, 132.5, 131.8, 130.0, 129.7, 128.1, 127.9, 127.4, 124.7, 121.8, 121.6, 116.4, 115.2, 74.1, 41.5, 21.4. **HRMS**: calculated for : $C_{28}H_{24}N_2O_3$ [M+Na$^+$] 459.1679, found 459.1677.

(*E*)-5-(4-(methylsulfonyl)phenoxy)-*N*-(quinolin-8-yl)hex-3-enamide

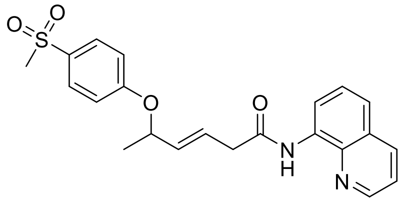

Compound **24** was isolated in 92 % yield with Cu_2O for 10 h. Flash silica gel chromatography (PE : EA = 4:1). Yellow solid. **^{1}H NMR** (400 MHz, Chloroform-*d*) δ 9.9 (s, 1H), 8.7 (dq, *J* = 4.7, 2.7, 2.2 Hz, 2H), 8.2 (dd, *J* = 8.3, 1.7 Hz, 1H), 7.9 – 7.7 (m, 2H), 7.6 – 7.4 (m, 3H), 7.1 – 6.9 (m, 2H), 6.2 – 6.0 (m, 1H), 5.9 – 5.8 (m, 1H), 5.0 (p, *J* = 6.3 Hz, 1H), 3.5 – 3.1 (m, 2H), 3.0 (s, 3H), 1.6 (d, *J* = 6.3 Hz, 3H). **^{13}C NMR** (101 MHz, Chloroform-*d*) δ 168.7, 162.1, 148.2, 138.3, 136.4, 135.3, 134.1, 132.1, 129.4, 127.9, 127.3, 125.1, 121.8,

121.7, 116.4, 116.1, 74.5, 44.8, 41.4, 21.4. **HRMS**: calculated for : $C_{22}H_{22}N_2O_4S$ [M+Na$^+$] 433.1192, found 433.1192.

(*E*)-5-(4-cyanophenoxy)-*N*-(quinolin-8-yl)hex-3-enamide

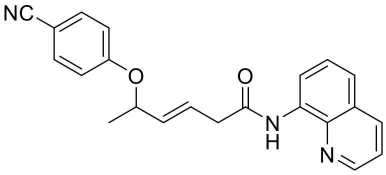

Compound **25** was isolated in 89 % yield with Cu_2O for 10 h. Flash silica gel chromatography (PE : EA = 4:1). Colorless liquid. **^{1}H NMR** (400 MHz, Chloroform-*d*) δ 9.9 (s, 1H), 8.9 – 8.6 (m, 2H), 8.2 (dd, *J* = 8.3, 1.7 Hz, 1H), 7.7 – 7.4 (m, 5H), 7.0 – 6.8 (m, 2H), 6.2 – 6.0 (m, 1H), 5.9 – 5.8 (m, 1H), 5.3 – 4.7 (m, 1H), 3.3 (dd, *J* = 7.1, 1.2 Hz, 2H), 1.6 (d, *J* = 6.4 Hz, 3H). **^{13}C NMR** (101 MHz, Chloroform-*d*) δ 168.7, 161.3, 148.1, 138.3, 136.5, 135.4, 134.2, 133.9, 127.9, 127.4, 125.2, 121.8, 121.7, 116.4, 116.4, 103.7, 74.4, 41.4, 21.4. **HRMS**: calculated for : $C_{22}H_{19}N_3O_2$ [M+Na$^+$] 380.1369, found 380.1371.

(*E*)-5-(4-(3-oxobutyl)phenoxy)-*N*-(quinolin-8-yl)hex-3-enamide

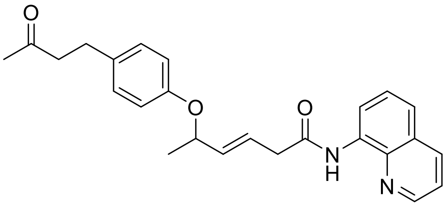

Compound **26** was isolated in 76 % yield with Cu_2O for 10 h. Flash silica gel chromatography (PE : EA = 4:1). Colorless liquid. **^{1}H NMR** (400 MHz, Chloroform-*d*) δ 10.0 (s, 1H), 8.9 – 8.6 (m, 2H), 8.2 (dd, *J* = 8.3, 1.7 Hz, 1H), 7.6 – 7.4 (m, 3H), 7.1 – 6.9 (m, 2H), 6.9 – 6.7 (m, 2H), 6.2 – 5.6 (m, 2H), 5.1 – 4.6 (m, 1H), 3.7 – 3.1 (m, 2H), 2.9 – 2.7 (m, 2H), 2.7 – 2.5 (m, 2H), 2.1 (s, 3H), 1.5 (d, *J* = 6.4 Hz, 3H). **^{13}C NMR** (101 MHz, Chloroform-*d*) δ 208.2, 169.1, 156.3, 148.1, 138.5, 137.0, 136.3, 134.3, 133.2, 129.1, 127.9, 127.4, 124.1, 121.6, 121.6, 116.4, 116.1, 74.0, 45.4, 41.6, 30.1, 28.9, 21.5. **HRMS**: calculated for : $C_{25}H_{26}N_2O_3$ [M+Na$^+$] 425.1836, found 425.1832.

(*E*)-5-(4-pentylphenoxy)-*N*-(quinolin-8-yl)hex-3-enamide

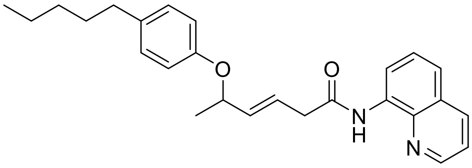

Compound **27** was isolated in 53 % yield with Cu_2O for 10 h. Flash silica gel chromatography (PE : EA = 4:1). Colorless liquid. **^{1}H NMR** (400 MHz, Chloroform-*d*) δ 10.0 (s, 1H), 9.4 – 8.3 (m, 2H), 8.1 (dd, *J* = 8.3, 1.7 Hz, 1H), 7.7 – 7.3 (m, 3H), 7.1 – 6.9 (m, 2H), 6.9 – 6.8 (m, 2H), 6.2 – 5.7 (m, 2H), 4.9 (p, *J* = 6.2 Hz, 1H), 3.5 – 3.1 (m, 2H), 2.7 – 2.3 (m, 2H), 1.6 – 1.4 (m, 5H), 1.4 – 1.2 (m,

4H), 1.0 – 0.6 (m, 3H). **^{13}C NMR** (101 MHz, Chloroform-*d*) δ 169.1, 155.9, 148.2, 138.5, 137.3, 136.3, 135.2, 134.3, 129.2, 127.9, 127.4, 123.9, 121.6, 121.6, 116.4, 115.9, 74.0, 41.7, 35.0, 31.5, 31.3, 22.6, 21.5, 14.1. **HRMS**: calculated for : $C_{26}H_{30}N_2O_2$ [M+Na$^+$] 425.2199, found 425.2191.

(*E*)-5-(perfluorophenoxy)-*N*-(quinolin-8-yl)hex-3-enamide

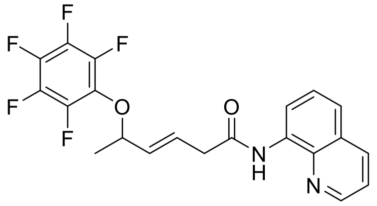

Compound **28** was isolated in 91 % yield with Cu_2O for 10 h. Flash silica gel chromatography (PE : EA = 4:1). White solid. **^{1}H NMR** (400 MHz, Chloroform-*d*) δ 9.9 (s, 1H), 8.8 (dd, *J* = 4.2, 1.7 Hz, 1H), 8.7 (dd, *J* = 6.6, 2.4 Hz, 1H), 8.2 (dd, *J* = 8.2, 1.7 Hz, 1H), 7.6 – 7.5 (m, 2H), 7.5 (dd, *J* = 8.3, 4.2 Hz, 1H), 6.0 – 5.9 (m, 1H), 5.9 – 5.8 (m, 1H), 4.8 (p, *J* = 6.6 Hz, 1H), 3.5 – 3.0 (m, 2H), 1.6 (d, *J* = 6.3 Hz, 3H). **^{13}C NMR** (101 MHz, Chloroform-*d*) δ 168.5, 148.2, 138.4, 136.4, 134.4, 134.2, 128.0, 127.7, 127.4, 121.8, 121.7, 116.5, 82.2, 41.3, 21.1. **^{19}F NMR** (376 MHz, Chloroform-*d*) δ -154.0 – -156.2 (m, 2F), -161.7 – -163.3 (m, 1F), -163.3 – -164.4 (m, 2F). **HRMS**: calculated for : $C_{21}H_{15}F_5N_2O_2$ [M+Na$^+$] 445.0946, found 445.0945.

(*E*)-5-((5-oxo-5,6,7,8-tetrahydronaphthalen-1-yl)oxy)-*N*-(quinolin-8-yl)hex-3-enamide

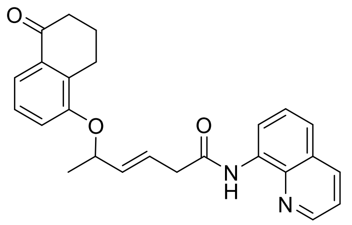

Compound **29** was isolated in 64 % yield with Cu_2O for 10 h. Flash silica gel chromatography (PE : EA = 4:1). Colorless liquid. **^{1}H NMR** (400 MHz, Chloroform-*d*) δ 10.0 (s, 1H), 9.0 – 8.5 (m, 2H), 8.2 (dd, *J* = 8.3, 1.7 Hz, 1H), 7.7 – 7.4 (m, 4H), 7.2 – 6.9 (m, 2H), 6.2 – 5.6 (m, 2H), 4.9 (p, *J* = 6.3 Hz, 1H), 3.3 (d, *J* = 7.0 Hz, 2H), 2.9 (dd, *J* = 6.9, 5.5 Hz, 2H), 2.6 (dd, *J* = 7.6, 5.6 Hz, 2H), 2.1 (p, *J* = 6.3 Hz, 2H), 1.6 (d, *J* = 6.3 Hz, 3H). **^{13}C NMR** (101 MHz, Chloroform-*d*) δ 198.7, 169.0, 155.1, 148.1, 138.4, 136.5, 136.4, 134.4, 134.3, 133.8, 128.0, 127.4, 126.6, 124.3, 121.7, 121.7, 119.0, 117.7, 116.5, 74.6, 41.6, 38.9, 23.1, 22.6, 21.6. **HRMS**: calculated for : $C_{25}H_{24}N_2O_3$ [M+Na$^+$] 423.1679, found 423.1680.

(*E*)-5-(4-cyano-3-(trifluoromethyl)phenoxy)-*N*-(quinolin-8-yl)hex-3-enamide

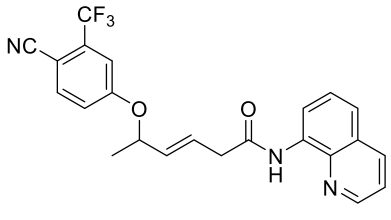

Compound **30** was isolated in 90 % yield with Cu_2O for 10 h. Flash silica gel chromatography (PE : EA = 4:1). Yellow liquid. **^{1}H NMR** (400 MHz, Chloroform-*d*) δ 9.9 (s, 1H), 8.8 – 8.6 (m, 2H), 8.2 (dd, *J* = 8.3, 1.7 Hz, 1H), 7.8 (d, *J* = 2.1 Hz, 1H), 7.6 (dd, *J* = 8.8, 2.2 Hz, 1H), 7.6 – 7.5 (m, 2H), 7.5 (dd, *J* = 8.3, 4.2 Hz, 1H), 6.4 – 5.9 (m, 1H), 6.0 – 5.7 (m, 1H), 5.1 (p, *J* = 6.4 Hz, 1H), 3.3 (dd, *J* = 7.2, 1.3 Hz, 2H), 1.6 (d, *J* = 6.3 Hz, 3H). **^{13}C NMR** (101 MHz, Chloroform-*d*) δ 168.7, 159.2, 148.2, 138.4, 137.2, 136.5, 134.3 (d, *J* = 45.2 Hz), 131.4 (q, *J* = 5.3 Hz), 128.0, 127.4, 126.0, 122.0, 121.7, 120.7 (q, *J* = 31.9 Hz), 117.9, 116.5, 115.2, 103.6, 76.1, 41.4, 21.4. **^{19}F NMR** (376 MHz, Chloroform-*d*) δ -63.2. **HRMS**: calculated for : $C_{23}H_{19}F_3N_3O_2$ [M+H$^+$] 426.1424, found 426.1421.

(*E*)-5-(2-bromo-4-nitrophenoxy)-*N*-(quinolin-8-yl)hex-3-enamide

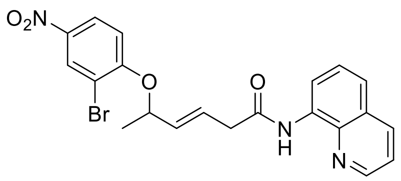

Compound **31** was isolated in 92 % yield with Cu_2O for 10 h. Flash silica gel chromatography (PE : EA = 4:1). Colorless liquid. **^{1}H NMR** (400 MHz, Chloroform-*d*) δ 9.9 (s, 1H), 9.0 – 8.5 (m, 2H), 8.4 (d, *J* = 2.8 Hz, 1H), 8.2 (dd, *J* = 8.3, 1.7 Hz, 1H), 8.0 (dd, *J* = 9.1, 2.8 Hz, 1H), 7.6 – 7.4 (m, 3H), 7.0 (d, *J* = 9.2 Hz, 1H), 6.3 – 6.0 (m, 1H), 6.0 – 5.7 (m, 1H), 5.1 (p, *J* = 6.4 Hz, 1H), 3.4 (d, *J* = 7.1 Hz, 2H), 1.7 (d, *J* = 6.4 Hz, 3H). **^{13}C NMR** (101 MHz, Chloroform-*d*) δ 168.5, 159.6, 148.1, 141.2, 138.3, 136.5, 134.5, 134.1, 129.1, 127.9, 127.4, 126.1, 124.3, 121.8, 121.7, 116.4, 113.5, 112.8, 76.6, 41.4, 21.5. **HRMS**: calculated for : $C_{21}H_{18}BrN_3O_4$ [M+Na$^+$] 478.0373, found 478.0373.

(*E*)-5-(2-bromo-3-nitrophenoxy)-*N*-(quinolin-8-yl)hex-3-enamide

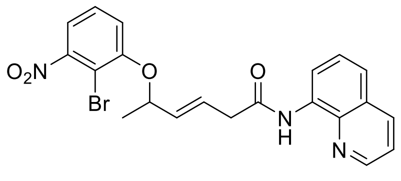

Compound **32** was isolated in 90 % yield with Cu_2O for 10 h. Flash silica gel chromatography (PE : EA = 4:1). Yellow liquid. **^{1}H NMR** (400 MHz, Chloroform-*d*) δ 9.9 (s, 1H), 8.8 – 8.6 (m, 2H), 8.1

(dd, *J* = 8.3, 1.7 Hz, 1H), 7.7 – 7.3 (m, 3H), 7.2 – 7.0 (m, 3H), 6.2 – 6.0 (m, 1H), 6.0 – 5.7 (m, 1H), 5.0 (p, *J* = 6.3 Hz, 1H), 3.3 (dd, *J* = 7.1, 1.2 Hz, 2H), 1.6 (d, *J* = 6.3 Hz, 3H). **^{13}C NMR** (101 MHz, Chloroform-*d*) δ 168.6, 155.8, 151.8, 148.1, 138.3, 136.3, 135.0, 134.1, 128.3, 127.8, 127.3, 125.6, 121.7, 121.6, 118.2, 116.7, 116.3, 105.9, 76.8, 41.4, 21.4. **HRMS**: calculated for : $C_{21}H_{18}BrN_3O_4$ [M+Na$^+$] 478.0373, found 478.0373.

Methyl (*E*)-3-bromo-4-((6-oxo-6-(quinolin-8-ylamino)hex-3-en-2-yl)oxy)benzoate

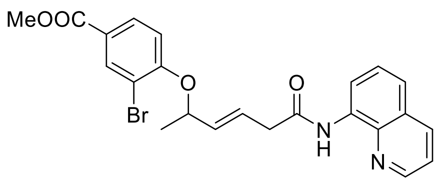

Compound **33** was isolated in 92 % yield with Cu_2O for 10 h. Flash silica gel chromatography (PE : EA = 4:1). Yellow liquid. **^{1}H NMR** (400 MHz, Chloroform-*d*) δ 9.9 (s, 1H), 8.9 – 8.6 (m, 2H), 8.3 – 8.0 (m, 2H), 7.8 (dd, *J* = 8.6, 2.1 Hz, 1H), 7.6 – 7.3 (m, 3H), 7.0 (d, *J* = 8.6 Hz, 1H), 6.2 – 6.0 (m, 1H), 6.0 – 5.7 (m, 1H), 5.0 (p, *J* = 6.3 Hz, 1H), 3.8 (s, 3H), 3.3 (d, *J* = 7.1 Hz, 2H), 1.6 (d, *J* = 6.3 Hz, 3H). **^{13}C NMR** (101 MHz, Chloroform-*d*) δ 168.7, 165.6, 158.1, 148.1, 138.4, 136.3, 135.4, 134.9, 134.2, 130.2, 127.9, 127.3, 125.3, 123.6, 121.7, 121.6, 116.4, 114.1, 112.7, 75.8, 52.1, 41.5, 21.4. **HRMS**: calculated for : $C_{23}H_{21}BrN_2O_4$ [M+Na$^+$] 491.0577, found 491.0573.

(*E*)-5-(3-bromo-5-fluorophenoxy)-*N*-(quinolin-8-yl)hex-3-enamide

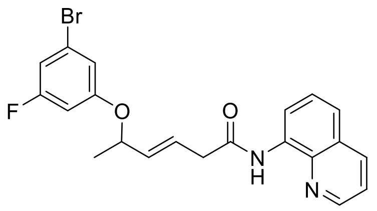

Compound **34** was isolated in 83 % yield with Cu_2O for 10 h. Flash silica gel chromatography (PE : EA = 4:1). Yellow liquid. **^{1}H NMR** (400 MHz, Chloroform-*d*) δ 10.0 (s, 1H), 9.1 – 8.5 (m, 2H), 8.1 (dd, *J* = 8.3, 1.7 Hz, 1H), 7.7 – 7.3 (m, 3H), 7.0 – 6.8 (m, 1H), 6.8 (dt, *J* = 8.0, 2.0 Hz, 1H), 6.6 (dt, *J* = 10.6, 2.3 Hz, 1H), 6.2 – 6.0 (m, 1H), 6.0 – 5.7 (m, 1H), 5.0 – 4.7 (m, 1H), 3.6 – 2.9 (m, 2H), 1.5 (d, *J* = 6.4 Hz, 3H). **^{13}C NMR** (101 MHz, Chloroform-*d*) δ 168.8, 164.5, 162.0, 159.7, 159.6, 148.2, 138.4, 136.4, 135.5, 134.2, 127.9, 127.4, 125.0, 122.5 (d, *J* = 12.6 Hz), 121.7 (d, *J* = 7.7 Hz), 116.4, 115.4 (d, *J* = 3.2 Hz), 111.5 (d, *J* = 25.0 Hz), 102.7 (d, *J* = 24.7 Hz), 74.8, 41.5, 21.3. **^{19}F NMR** (376 MHz, Chloroform-*d*) δ -110.1.

HRMS: calculated for : $C_{21}H_{18}BrFN_2O_2$ [M+Na$^+$] 451.0428, found 451.0432.

Methyl (*E*)-2-((6-oxo-6-(quinolin-8-ylamino)hex-3-en-2-yl)oxy)benzoate

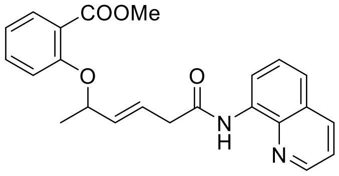

Compound **35** was isolated in 47 % yield with Cu_2O for 10 h. Flash silica gel chromatography (PE : EA = 3:1). Colorless liquid. **^{1}H NMR** (400 MHz, Chloroform-*d*) δ 10.0 (s, 1H), 9.0 – 8.6 (m, 2H), 8.2 (dd, *J* = 8.3, 1.7 Hz, 1H), 7.7 (dd, *J* = 7.7, 1.8 Hz, 1H), 7.6 – 7.5 (m, 2H), 7.4 (dd, *J* = 8.3, 4.2 Hz, 1H), 7.4 – 7.3 (m, 1H), 7.1 – 7.0 (m, 1H), 7.0 – 6.8 (m, 1H), 6.1 – 6.0 (m, 1H), 6.0 – 5.9 (m, 1H), 5.0 (p, *J* = 6.3 Hz, 1H), 3.9 (s, 3H), 3.4 – 3.3 (m, 2H), 1.6 (d, *J* = 6.3 Hz, 3H). **^{13}C NMR** (101 MHz, Chloroform-*d*) δ 169.0, 167.0, 157.5, 148.2, 138.5, 136.6, 136.4, 134.3, 133.1, 131.5, 128.0, 127.4, 124.5, 121.7, 121.7, 121.6, 120.6, 116.5, 116.2, 75.8, 51.9, 41.6, 21.5. **HRMS**: calculated for : $C_{23}H_{22}N_2O_4$ [M+Na$^+$] 413.1472, found 413.1467.

(*E*)-5-(4-((1*r*,4*r*)-4-ethylcyclohexyl)phenoxy)-*N*-(quinolin-8-yl)hex-3-enamide

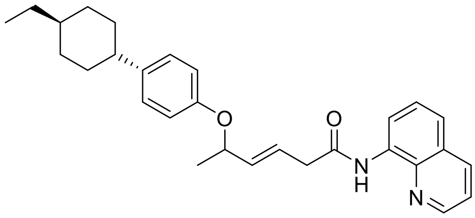

Compound **36** was isolated in 48 % yield with Cu_2O for 10 h. Flash silica gel chromatography (PE : EA = 4:1). Colorless liquid. **^{1}H NMR** (400 MHz, Chloroform-*d*) δ 10.0 (s, 1H), 9.1 – 8.6 (m, 2H), 8.2 (dd, *J* = 8.3, 1.7 Hz, 1H), 7.7 – 7.4 (m, 3H), 7.1 – 7.0 (m, 2H), 7.0 – 6.8 (m, 2H), 6.4 – 5.8 (m, 2H), 4.9 (p, *J* = 6.2 Hz, 1H), 3.3 (d, *J* = 6.8 Hz, 2H), 2.3 (tt, *J* = 12.1, 3.3 Hz, 1H), 1.9 – 1.8 (m, 4H), 1.5 (d, *J* = 6.4 Hz, 3H), 1.4 – 1.1 (m, 5H), 1.1 – 1.0 (m, 2H), 0.9 (t, *J* = 7.4 Hz, 3H). **^{13}C NMR** (101 MHz, Chloroform-*d*) δ 169.1, 156.0, 148.2, 140.3, 138.5, 137.3, 136.3, 134.4, 128.0, 127.6, 127.4, 123.9, 121.6, 116.4, 115.8, 74.0, 43.7, 41.7, 39.1, 34.5, 34.5, 33.2, 30.0, 21.5, 11.6. **HRMS**: calculated for : $C_{29}H_{34}N_2O_2$ [M+Na$^+$] 465.2512, found 465.2507.

(*E*)-5-(((8*R*,9*S*,13*S*,14*S*)-13-methyl-17-oxo-7,8,9,11,12,13,14,15,16,17-decahydro-6*H*-cyclopenta[*a*]phenanthren-3-yl)oxy)-*N*-(quinolin-8-yl)hex-3-enamide

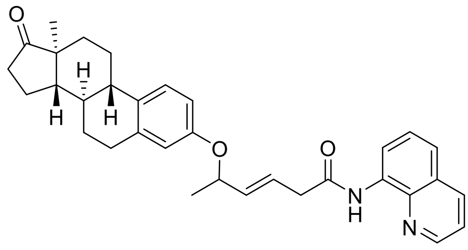

Compound **37** was isolated in 75 % yield with Cu_2O for 10 h. Flash silica gel chromatography (PE : EA = 2:1). Colorless liquid. **^{1}H NMR** (400 MHz, Chloroform-*d*) δ 10.0 (s, 1H), 8.9 – 8.6 (m, 2H), 8.1 (dd, *J* = 8.3, 1.7 Hz, 1H), 7.6 – 7.4 (m, 3H), 7.1 (t, *J* = 7.7 Hz, 1H), 6.8 – 6.6 (m, 2H), 6.3 – 5.7 (m, 2H), 4.9 (p, *J* = 6.3 Hz, 1H), 3.3 (d, *J* = 7.0 Hz, 2H), 2.9 – 2.7 (m, 2H), 2.6 – 2.4 (m, 1H), 2.4 – 2.2 (m, 1H), 2.2 – 1.8 (m, 6H), 1.7 – 1.6 (m, 1H), 1.5 – 1.2 (m, 7H), 1.0 – 0.7 (m, 3H). **^{13}C NMR** (101 MHz, Chloroform-*d*) δ 169.1, 155.8, 148.1, 138.4, 137.6, 137.0, 136.3, 134.3, 132.1, 127.9, 127.3, 126.2, 124.0, 121.6, 116.4, 116.2, 116.1, 113.4, 73.8, 50.3, 48.0, 43.9, 41.6, 38.2, 35.8, 31.5, 29.5, 26.5, 25.8, 21.4, 13.8. **HRMS**: calculated for : $C_{33}H_{36}N_2O_3$ [M+Na$^+$] 531.2618, found 531.2612.

4-(((*E*)-6-oxo-6-(quinolin-8-ylamino)hex-3-en-2-yl)oxy)phenyl (2*R*)-2-(4-isobutylphenyl)propanoate

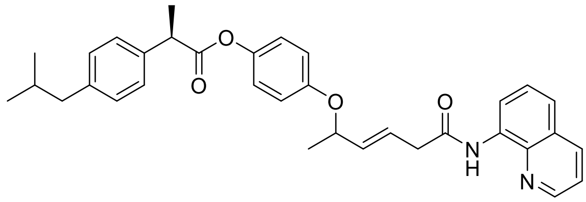

Compound **38** was isolated in 75 % yield with Cu_2O for 10 h. Flash silica gel chromatography (PE : EA = 3:1). Colorless liquid. **^{1}H NMR** (400 MHz, Chloroform-*d*) δ 10.0 (s, 1H), 9.0 – 8.5 (m, 2H), 8.1 (dd, *J* = 8.3, 1.7 Hz, 1H), 7.6 – 7.4 (m, 2H), 7.4 – 7.4 (m, 1H), 7.3 – 7.2 (m, 2H), 7.2 – 7.1 (m, 2H), 6.9 – 6.8 (m, 4H), 6.2 – 5.6 (m, 2H), 5.1 – 4.4 (m, 1H), 4.1 – 3.8 (m, 1H), 3.3 (d, *J* = 7.0 Hz, 2H), 2.5 (d, *J* = 7.2 Hz, 2H), 2.0 – 1.7 (m, 1H), 1.5 (dd, *J* = 20.3, 6.7 Hz, 6H), 0.9 (d, *J* = 6.6 Hz, 6H). **^{13}C NMR** (101 MHz, Chloroform-*d*) δ 173.5, 169.0, 155.5, 148.1, 144.4, 140.7, 138.4, 137.3, 136.8, 136.3, 134.3, 129.5, 127.9, 127.3, 127.2, 124.2, 122.1, 121.6, 121.6, 116.5, 116.4, 74.5, 45.2, 45.0, 41.5, 30.2, 22.4, 21.4, 18.5. **HRMS**: calculated for : $C_{34}H_{36}N_2O_4$ [M+Na$^+$] 559.2567, found 559.2569.

4-(((*E*)-6-oxo-6-(quinolin-8-ylamino)hex-3-en-2-yl)oxy)phenyl (2*R*)-2-(6-methoxynaphthalen-2-yl)propanoate

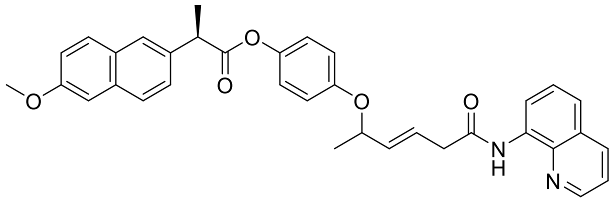

Compound **39** was isolated in 83 % yield with Cu_2O for 10 h. Flash silica gel chromatography (PE : EA = 3:1). White solid. **^{1}H NMR** (400 MHz, Chloroform-*d*) δ 10.0 (s, 1H), 8.9 – 8.6 (m, 2H), 8.2 – 8.0 (m, 1H), 7.8 – 7.6 (m, 3H), 7.6 – 7.4 (m, 3H), 7.4 – 7.3 (m, 1H), 7.2 – 7.0 (m, 2H), 6.9 – 6.6 (m, 4H), 6.1 – 5.9 (m, 1H), 5.9 – 5.8 (m, 1H), 4.8 (p, *J* = 6.2 Hz, 1H), 4.0 (q, *J* = 7.1 Hz, 1H), 3.9 (s, 3H), 3.3 (d, *J* = 6.8 Hz, 2H), 1.7 (d, *J* = 7.1 Hz, 3H), 1.5 (d, *J* = 6.4 Hz, 3H). **^{13}C NMR** (101 MHz, Chloroform-*d*) δ 173.4, 168.9, 157.7, 155.5, 148.1, 144.4, 138.4, 136.8, 136.2, 135.2, 134.3, 133.8, 129.3, 129.0, 127.9, 127.3, 127.3, 126.1, 126.1, 124.2, 122.0, 121.6, 121.6, 119.1, 116.5, 116.3, 105.6, 74.5, 55.3, 45.5, 41.5, 21.4, 18.5. **HRMS**: calculated for : $C_{35}H_{32}N_2O_5$ [M+Na$^+$] 583.2203, found 583.2201.

(*E*)-5-methoxy-*N*-(quinolin-8-yl)hex-3-enamide

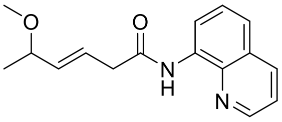

Compound **40** was isolated in 51 % yield with CuCl for 10 h. Flash silica gel chromatography (PE : EA = 4:1). Colorless liquid. **^{1}H NMR** (400 MHz, Chloroform-*d*) δ 10.0 (s, 1H), 9.0 – 8.5 (m, 2H), 8.1 (dd, *J* = 8.3, 1.7 Hz, 1H), 7.6 – 7.5 (m, 2H), 7.4 (dd, *J* = 8.3, 4.2 Hz, 1H), 6.1 – 5.9 (m, 1H), 5.8 – 5.6 (m, 1H), 3.9 (p, *J* = 6.4 Hz, 1H), 3.4 (s, 3H), 3.4 (d, *J* = 7.1 Hz, 2H), 1.4 (d, *J* = 6.4 Hz, 3H). **^{13}C NMR** (101 MHz, Chloroform-*d*) δ 169.3, 148.2, 138.5, 138.1, 136.4, 134.4, 128.0, 127.5, 124.7, 121.7, 116.4, 77.8, 56.2, 41.8, 21.5. **HRMS**: calculated for : $C_{16}H_{18}N_2O_2$ [M+Na$^+$] 293.1260, found 293.1255.

(*E*)-5-ethoxy-*N*-(quinolin-8-yl)hex-3-enamide

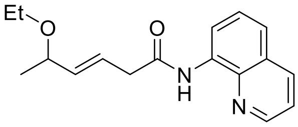

Compound **41** was isolated in 48 % yield with CuCl for 10 h. Flash silica gel chromatography (PE : EA = 4:1). Colorless liquid. **^{1}H NMR** (400 MHz, Chloroform-*d*) δ 10.0 (s, 1H), 9.1 – 8.6 (m, 2H), 8.1 (dd, *J* = 8.3, 1.7 Hz, 1H), 7.6 – 7.3 (m, 3H), 6.1 – 5.8 (m, 1H), 5.8 – 5.6 (m, 1H), 4.0 (p, *J* = 6.6 Hz, 1H), 3.8 – 3.5 (m, 1H), 3.5 – 3.4 (m, 1H), 3.4 –

3.3 (m, 2H), 1.4 (d, *J* = 6.3 Hz, 3H), 1.2 (t, *J* = 7.0 Hz, 3H). **^{13}C NMR** (101 MHz, Chloroform-*d*) δ 169.1, 147.9, 138.5, 138.3, 136.2, 134.2, 127.8, 127.2, 123.8, 121.5, 121.5, 116.2, 75.7, 63.5, 41.6, 21.5, 15.4. **HRMS**: calculated for : $C_{17}H_{20}N_2O_2$ [M+Na$^+$] 307.1417, found 307.1414.

(*E*)-5-(cyclopropylmethoxy)-*N*-(quinolin-8-yl)hex-3-enamide

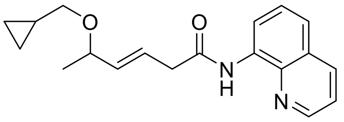

Compound **42** was isolated in 46 % yield with CuCl for 10 h. Flash silica gel chromatography (PE : EA = 4:1). Colorless liquid. **^{1}H NMR** (400 MHz, Chloroform-*d*) δ 10.0 (s, 1H), 9.0 – 8.6 (m, 2H), 8.2 (dd, *J* = 8.3, 1.7 Hz, 1H), 7.7 – 7.3 (m, 3H), 6.1 – 5.9 (m, 1H), 5.9 – 5.6 (m, 1H), 4.1 – 3.8 (m, 1H), 3.4 – 3.3 (m, 3H), 3.3 – 3.2 (m, 1H), 1.4 (d, *J* = 6.4 Hz, 3H), 1.1 – 0.9 (m, 1H), 0.6 – 0.4 (m, 2H), 0.3 – 0.1 (m, 2H). **^{13}C NMR** (101 MHz, Chloroform-*d*) δ 169.3, 148.1, 138.7, 138.5, 136.4, 134.4, 127.9, 127.4, 123.9, 121.6, 121.6, 116.4, 75.8, 73.2, 41.7, 21.7, 10.9, 3.2, 3.0. **HRMS**: calculated for : $C_{19}H_{22}N_2O_2$ [M+Na$^+$] 333.1573, found 333.1571.

(*E*)-5-(benzyloxy)-*N*-(quinolin-8-yl)hex-3-enamide

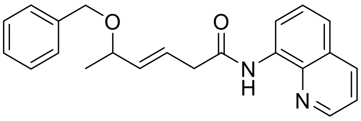

Compound **43** was isolated in 58 % yield with CuCl for 10 h. Flash silica gel chromatography (PE : EA = 4:1). Colorless liquid. **^{1}H NMR** (400 MHz, Chloroform-*d*) δ 10.0 (s, 1H), 9.1 – 8.5 (m, 2H), 8.1 (dd, *J* = 8.3, 1.7 Hz, 1H), 7.6 – 7.5 (m, 2H), 7.4 (dd, *J* = 8.3, 4.2 Hz, 1H), 7.4 – 7.2 (m, 5H), 6.1 – 5.9 (m, 1H), 5.9 – 5.6 (m, 1H), 4.7 (d, *J* = 11.9 Hz, 1H), 4.5 (d, *J* = 11.9 Hz, 1H), 4.1 (p, *J* = 6.6 Hz, 1H), 3.5 – 3.2 (m, 2H), 1.4 (d, *J* = 6.4 Hz, 3H). **^{13}C NMR** (101 MHz, Chloroform-*d*) δ 169.3, 148.2, 138.7, 138.5, 138.3, 136.4, 134.4, 128.4, 127.9, 127.8, 127.5, 127.4, 124.6, 121.7, 121.6, 116.4, 75.4, 70.2, 41.8, 21.6. **HRMS**: calculated for : $C_{22}H_{22}N_2O_2$ [M+Na$^+$] 369.1573, found 369.1567.

(*E*)-*N*-(quinolin-8-yl)-5-((4-(trifluoromethyl)benzyl)oxy)hex-3-enamide

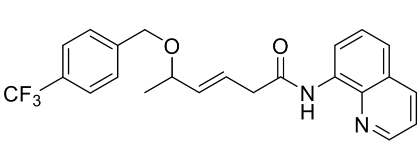

Compound **44** was isolated in 65 % yield with CuCl for 10 h. Flash silica gel chromatography

(PE : EA = 4:1). Colorless liquid. **^{1}H NMR** (400 MHz, Chloroform-*d*) δ 10.0 (s, 1H), 8.9 – 8.5 (m, 2H), 8.2 (dd, *J* = 8.3, 1.7 Hz, 1H), 7.6 – 7.3 (m, 7H), 6.1 – 5.9 (m, 1H), 5.9 – 5.6 (m, 1H), 4.7 (d, *J* = 12.5 Hz, 1H), 4.5 (d, *J* = 12.6 Hz, 1H), 4.1 (p, *J* = 6.6 Hz, 1H), 3.4 (dt, *J* = 7.0, 1.2 Hz, 2H), 1.4 (d, *J* = 6.3 Hz, 3H). **^{13}C NMR** (101 MHz, Chloroform-*d*) δ 169.2, 148.1, 142.9, 138.4, 137.7, 136.4, 134.3, 128.0, 127.7, 127.4, 125.3 (q, *J* = 3.9 Hz), 125.1, 121.7, 121.6, 116.5, 76.0, 69.3, 41.7, 21.6. **^{19}F NMR** (376 MHz, Chloroform-*d*) δ -62.4. **HRMS**: calculated for : $C_{23}H_{21}F_3N_2O_2$ [M+Na$^+$] 437.1447, found 437.1444.

(*E*)-5-(3-phenylpropoxy)-*N*-(quinolin-8-yl)hex-3-enamide

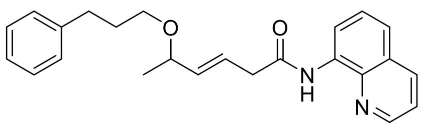

Compound **45** was isolated in 56 % yield with CuCl for 10 h. Flash silica gel chromatography (PE : EA = 4:1). Colorless liquid. **^{1}H NMR** (400 MHz, Chloroform-*d*) δ 10.0 (s, 1H), 9.3 – 8.6 (m, 2H), 8.2 (dd, *J* = 8.3, 1.7 Hz, 1H), 7.6 – 7.5 (m, 2H), 7.4 (dd, *J* = 8.3, 4.2 Hz, 1H), 7.3 – 7.2 (m, 2H), 7.2 – 7.1 (m, 3H), 6.0 – 5.9 (m, 1H), 5.8 – 5.6 (m, 1H), 3.9 (p, *J* = 6.5 Hz, 1H), 3.7 – 3.5 (m, 1H), 3.5 – 3.3 (m, 1H), 3.3 (dd, *J* = 7.1, 1.2 Hz, 2H), 2.8 – 2.5 (m, 2H), 2.1 – 1.8 (m, 2H), 1.4 (d, *J* = 6.4 Hz, 3H). **^{13}C NMR** (101 MHz, Chloroform-*d*) δ 169.4, 148.1, 142.1, 138.6, 138.5, 136.4, 134.4, 128.5, 128.3, 128.0, 127.4, 125.7, 124.0, 121.6, 116.4, 76.1, 67.5, 41.7, 32.4, 31.5, 21.6. **HRMS**: calculated for : $C_{24}H_{26}N_2O_2$ [M+Na$^+$] 397.1886, found 397.1882.

(*E*)-5-(2-(naphthalen-1-yl)ethoxy)-*N*-(quinolin-8-yl)hex-3-enamide

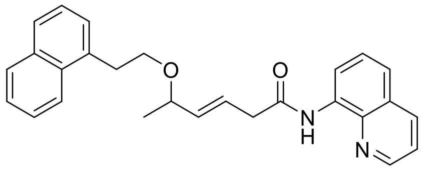

Compound **46** was isolated in 66 % yield with CuCl for 10 h. Flash silica gel chromatography (PE : EA = 4:1). Colorless liquid. **^{1}H NMR** (400 MHz, Chloroform-*d*) δ 10.0 (s, 1H), 8.7 (dd, *J* = 7.4, 1.7 Hz, 1H), 8.5 (dd, *J* = 4.3, 1.7 Hz, 1H), 8.1 (ddd, *J* = 22.2, 8.0, 1.6 Hz, 2H), 7.8 (dd, *J* = 7.5, 1.9 Hz, 1H), 7.7 (q, *J* = 4.1, 3.3 Hz, 1H), 7.6 – 7.4 (m, 2H), 7.4 (td, *J* = 7.5, 1.5 Hz, 2H), 7.3 – 7.3 (m, 3H), 5.9 (dt, *J* = 14.7, 7.1 Hz, 1H), 5.8 – 5.6 (m, 1H),

4.1 – 4.0 (m, 1H), 4.0 – 3.8 (m, 1H), 3.8 – 3.7 (m, 1H), 3.5 – 3.3 (m, 2H), 3.3 (dd, *J* = 7.1, 1.2 Hz, 2H), 1.4 (d, *J* = 6.4 Hz, 3H). **^{13}C NMR** (101 MHz, Chloroform-*d*) δ 169.2, 148.0, 138.5, 138.4, 136.2, 134.9, 134.3, 133.8, 132.1, 128.7, 127.9, 127.3, 127.0, 126.9, 125.9, 125.5, 125.5, 124.1, 123.7, 121.6, 121.6, 116.3, 76.3, 68.7, 41.7, 33.7, 21.6. **HRMS**: calculated for : $C_{27}H_{26}N_2O_2$ [M+Na$^+$] 433.1886, found 433.1885.

Methyl (*E*)-3-((6-oxo-6-(quinolin-8-ylamino)hex-3-en-2-yl)oxy)propanoate

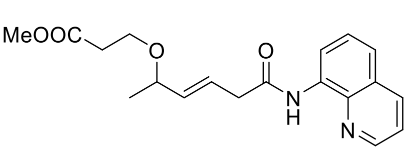

Compound **47** was isolated in 68 % yield with CuCl for 10 h. Flash silica gel chromatography (PE : EA = 3:1). Colorless liquid. **^{1}H NMR** (400 MHz, Chloroform-*d*) δ 10.0 (s, 1H), 9.2 – 8.5 (m, 2H), 8.2 (dd, *J* = 8.2, 1.7 Hz, 1H), 7.8 – 7.4 (m, 3H), 6.0 (dt, *J* = 14.8, 7.1 Hz, 1H), 5.7 (ddt, *J* = 15.5, 7.4, 1.5 Hz, 1H), 4.0 (p, *J* = 6.5 Hz, 1H), 3.9 – 3.8 (m, 1H), 3.7 – 3.6 (m, 4H), 3.4 – 3.1 (m, 2H), 2.6 (t, *J* = 6.5 Hz, 2H), 1.4 (d, *J* = 6.4 Hz, 3H). **^{13}C NMR** (101 MHz, Chloroform-*d*) δ 172.2, 169.2, 148.1, 138.5, 138.1, 136.4, 134.3, 127.9, 127.4, 124.3, 121.6, 116.4, 76.5, 63.7, 51.7, 41.7, 35.2, 21.5. **HRMS**: calculated for : $C_{19}H_{22}N_2O_4$ [M+Na$^+$] 365.1472, found 365.1467.

(*E*)-5-(2-bromoethoxy)-*N*-(quinolin-8-yl)hex-3-enamide

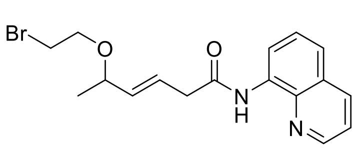

Compound **48** was isolated in 58 % yield with CuCl for 10 h. Flash silica gel chromatography (PE : EA = 4:1). Colorless liquid. **^{1}H NMR** (400 MHz, Chloroform-*d*) δ 10.0 (s, 1H), 9.0 – 8.6 (m, 2H), 8.2 (dd, *J* = 8.3, 1.7 Hz, 1H), 7.7 – 7.4 (m, 3H), 6.1 – 5.9 (m, 1H), 5.9 – 5.5 (m, 1H), 4.0 (p, *J* = 6.6 Hz, 1H), 4.0 – 3.8 (m, 1H), 3.8 – 3.7 (m, 1H), 3.5 (t, *J* = 6.2 Hz, 2H), 3.4 (dd, *J* = 7.2, 1.2 Hz, 2H), 1.4 (d, *J* = 6.4 Hz, 3H). **^{13}C NMR** (101 MHz, Chloroform-*d*) δ 169.1, 148.2, 138.4, 137.7, 136.4, 134.3, 127.9, 127.4, 124.8, 121.7, 121.7, 116.4, 76.7, 68.3, 41.6, 30.9, 21.5. **HRMS**: calculated for : $C_{17}H_{19}BrN_2O_2$ [M+Na$^+$] 385.0522, found 385.0517.

(*E*)-*N*-(quinolin-8-yl)-5-(2-(thiophen-2-yl)ethoxy)hex-3-enamide

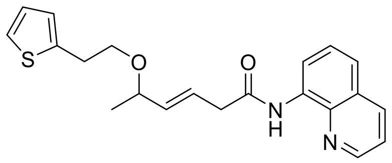

Compound **49** was isolated in 60 % yield with CuCl for 10 h. Flash silica gel chromatography (PE : EA = 4:1). Yellow liquid. **^{1}H NMR** (400 MHz, Chloroform-*d*) δ 10.0 (s, 1H), 8.8 (dd, *J* = 7.1, 1.9 Hz, 1H), 8.7 (dd, *J* = 4.2, 1.7 Hz, 1H), 8.1 (dd, *J* = 8.3, 1.7 Hz, 1H), 7.6 – 7.5 (m, 2H), 7.4 (dd, *J* = 8.3, 4.2 Hz, 1H), 7.1 (dd, *J* = 5.1, 1.2 Hz, 1H), 6.9 (dd, *J* = 5.1, 3.4 Hz, 1H), 6.8 (dd, *J* = 3.5, 1.2 Hz, 1H), 6.1 – 5.9 (m, 1H), 5.8 – 5.7 (m, 1H), 4.0 (p, *J* = 6.6 Hz, 1H), 3.9 – 3.7 (m, 1H), 3.7 – 3.5 (m, 1H), 3.4 – 3.2 (m, 2H), 3.2 – 2.9 (m, 2H), 1.4 (d, *J* = 6.4 Hz, 3H). **^{13}C NMR** (101 MHz, Chloroform-*d*) δ 169.3, 148.1, 141.4, 138.5, 138.3, 136.3, 134.3, 127.9, 127.4, 126.6, 125.1, 124.2, 123.6, 121.6, 116.4, 76.4, 69.1, 41.7, 30.7, 21.5. **HRMS**: calculated for : $C_{21}H_{22}N_2O_2S$ [M+Na$^+$] 389.1294, found 389.1288.

(*E*)-5-(2-phenoxyethoxy)-*N*-(quinolin-8-yl)hex-3-enamide

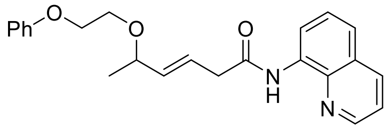

Compound **50** was isolated in 58 % yield with CuCl for 10 h. Flash silica gel chromatography (PE : EA = 4:1). Colorless liquid. **^{1}H NMR** (400 MHz, Chloroform-*d*) δ 10.0 (s, 1H), 9.0 – 8.5 (m, 2H), 8.1 (dd, *J* = 8.2, 1.7 Hz, 1H), 7.6 – 7.5 (m, 2H), 7.4 (dd, *J* = 8.2, 4.2 Hz, 1H), 7.3 – 7.1 (m, 2H), 7.0 – 6.8 (m, 3H), 6.1 – 5.9 (m, 1H), 5.9 – 5.6 (m, 1H), 4.3 – 4.0 (m, 3H), 4.0 – 3.8 (m, 1H), 3.8 – 3.6 (m, 1H), 3.5 – 3.2 (m, 2H), 1.4 (d, *J* = 6.4 Hz, 3H). **^{13}C NMR** (101 MHz, Chloroform-*d*) δ 169.2, 158.8, 148.1, 138.4, 138.1, 136.3, 134.3, 129.4, 127.9, 127.4, 124.6, 121.6, 121.6, 120.8, 116.4, 114.6, 76.7, 67.4, 66.7, 41.7, 21.6. **HRMS**: calculated for : $C_{23}H_{24}N_2O_3$ [M+Na$^+$] 399.1679, found 399.1677.

(*E*)-5-(hex-3-yn-1-yloxy)-*N*-(quinolin-8-yl)hex-3-enamide

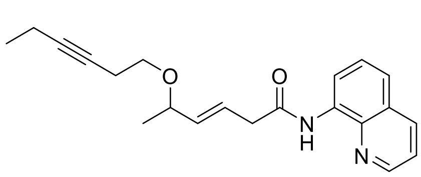

Compound **51** was isolated in 48 % yield with CuCl for 10 h. Flash silica gel chromatography (PE : EA = 4:1). Colorless liquid. **^{1}H NMR** (400 MHz, Chloroform-*d*) δ 10.0 (s, 1H), 8.9 – 8.6 (m,

2H), 8.2 (dd, J = 8.3, 1.7 Hz, 1H), 7.6 – 7.3 (m, 3H), 6.1 – 5.8 (m, 1H), 5.8 – 5.5 (m, 1H), 4.0 (p, J = 6.6 Hz, 1H), 3.7 – 3.6 (m, 1H), 3.5 – 3.4 (m, 1H), 3.4 – 3.2 (m, 2H), 2.5 – 2.3 (m, 2H), 2.3 – 2.0 (m, 2H), 1.4 (d, J = 6.4 Hz, 3H), 1.1 (t, J = 7.5 Hz, 3H). **^{13}C NMR** (101 MHz, Chloroform-*d*) δ 169.3, 148.2, 138.5, 138.4, 136.3, 134.3, 127.9, 127.4, 124.2, 121.6, 121.6, 116.4, 82.8, 76.2, 76.1, 67.2, 41.7, 21.6, 20.4, 14.2, 12.4. **HRMS**: calculated for : $C_{21}H_{24}N_2O_2$ [M+Na$^+$] 359.1730, found 357.1727.

(*E*)-5-(hex-5-yn-1-yloxy)-*N*-(quinolin-8-yl)hex-3-enamide

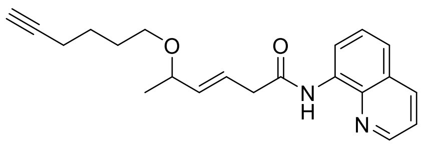

Compound **52** was isolated in 62 % yield with CuCl for 10 h. Flash silica gel chromatography (PE : EA = 3:1). Colorless liquid. **^{1}H NMR** (400 MHz, Chloroform-*d*) δ 10.0 (s, 1H), 9.1 – 8.7 (m, 2H), 8.2 (dd, J = 8.3, 1.7 Hz, 1H), 7.7 – 7.3 (m, 3H), 6.2 – 5.8 (m, 1H), 5.8 – 5.6 (m, 1H), 4.1 – 3.9 (m, 1H), 3.7 – 3.6 (m, 1H), 3.5 – 3.4 (m, 1H), 3.3 (dd, J = 7.1, 1.2 Hz, 2H), 2.4 – 2.2 (m, 2H), 1.9 (t, J = 2.6 Hz, 1H), 1.9 – 1.7 (m, 2H), 1.5 – 1.4 (m, 1H), 1.4 (d, J = 6.3 Hz, 3H). **^{13}C NMR** (101 MHz, Chloroform-*d*) δ 169.4, 148.2, 138.5, 136.4, 134.4, 128.0, 127.4, 124.0, 121.7, 116.5, 84.1, 76.2, 68.4, 66.6, 41.7, 28.9, 21.5, 15.3. **HRMS**: calculated for : $C_{21}H_{24}N_2O_2$ [M+Na$^+$] 359.1730, found 359.1723.

(*E*)-5-(hex-5-en-1-yloxy)-*N*-(quinolin-8-yl)hex-3-enamide

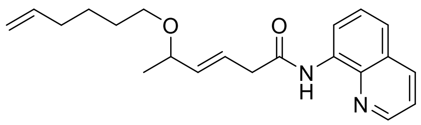

Compound **53** was isolated in 51 % yield with CuCl for 10 h. Flash silica gel chromatography (PE : EA = 3:1). Colorless liquid. **^{1}H NMR** (400 MHz, Chloroform-*d*) δ 10.0 (s, 1H), 9.1 – 8.5 (m, 2H), 8.2 (dd, J = 8.3, 1.7 Hz, 1H), 7.8 – 7.4 (m, 3H), 6.1 – 5.9 (m, 1H), 5.8 – 5.6 (m, 2H), 5.0 – 4.7 (m, 2H), 3.9 (p, J = 6.6 Hz, 1H), 3.6 (dt, J = 9.3, 6.6 Hz, 1H), 3.5 – 3.2 (m, 3H), 2.1 – 1.9 (m, 2H), 1.6 – 1.5 (m, 2H), 1.5 – 1.4 (m, 2H), 1.4 (d, J = 6.4 Hz, 3H). **^{13}C NMR** (101 MHz, Chloroform-*d*) δ 169.4, 148.1, 138.8, 138.7, 138.5, 136.4, 134.4, 127.9, 127.4, 123.9, 121.6, 121.6, 116.4, 114.5, 76.0, 68.3, 41.7, 33.6, 29.5, 25.6, 21.5. **HRMS**: calculated for : $C_{21}H_{26}N_2O_2$ [M+Na$^+$] 361.1886, found 361.1880.

(*E*)-5-((1,1,1,3,3,3-hexafluoropropan-2-yl)oxy)-*N*-(quinolin-8-yl)hex-3-enamide

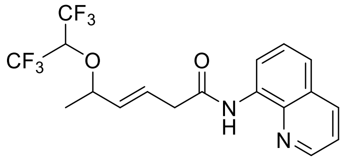

Compound **54** was isolated in 77 % yield with CuCl for 10 h. Flash silica gel chromatography (PE : EA = 3:1). Colorless liquid. **^{1}H NMR** (400 MHz, Chloroform-*d*) δ 9.9 (s, 1H), 9.0 – 8.5 (m, 2H), 8.2 (dd, *J* = 8.3, 1.7 Hz, 1H), 7.8 – 7.4 (m, 3H), 6.2 – 6.0 (m, 1H), 5.9 – 5.5 (m, 1H), 4.5 – 4.1 (m, 2H), 3.7 – 3.1 (m, 2H), 1.5 (d, *J* = 6.3 Hz, 3H). **^{13}C NMR** (101 MHz, Chloroform-*d*) δ 168.4, 148.2, 138.4, 136.5, 134.6, 134.2, 128.6, 128.0, 127.4, 121.9, 121.7, 116.5, 80.5, 72.6 (p, *J* = 32.3 Hz), 41.2, 21.2. **^{19}F NMR** (377 MHz, Chloroform-*d*) δ -73.6 (q, *J* = 9.0 Hz), -74.0 (q, *J* = 9.0 Hz). **HRMS**: calculated for : $C_{18}H_{16}F_6N_2O_2$ [M+Na$^+$] 429.1008, found 429.1001.

(*E*)-*N*-(quinolin-8-yl)-5-(2,2,2-trifluoroethoxy)hex-3-enamide

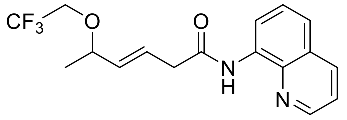

Compound **55** was isolated in 72 % yield with CuCl for 10 h. Flash silica gel chromatography (PE : EA = 3:1). Colorless liquid. **^{1}H NMR** (400 MHz, Chloroform-*d*) δ 10.0 (s, 1H), 8.9 – 8.6 (m, 2H), 8.2 (dd, *J* = 8.2, 1.7 Hz, 1H), 7.8 – 7.4 (m, 3H), 6.3 – 5.9 (m, 1H), 5.8 – 5.5 (m, 1H), 4.1 (p, *J* = 6.6 Hz, 1H), 4.0 – 3.9 (m, 1H), 3.9 – 3.7 (m, 1H), 3.5 – 3.2 (m, 2H), 1.4 (d, *J* = 6.3 Hz, 3H). **^{13}C NMR** (101 MHz, Chloroform-*d*) δ 168.8, 148.2, 138.4, 136.4, 136.4, 134.2, 128.0, 127.4, 126.1, 125.6, 122.8, 121.8, 121.7, 116.5, 78.0, 65.6 (q, *J* = 33.8 Hz), 41.5, 21.3. **^{19}F NMR** (376 MHz, Chloroform-*d*) δ -74.1. **HRMS**: calculated for : $C_{17}H_{17}F_3N_2O_2$ [M+Na$^+$] 361.1134 found 361.1128.

(*E*)-*N*-(quinolin-8-yl)-5-(3,3,3-trifluoropropoxy)hex-3-enamide

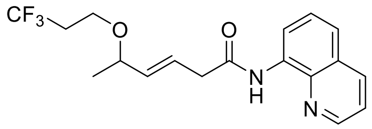

Compound **56** was isolated in 65 % yield with CuCl for 10 h. Flash silica gel chromatography (PE : EA = 3:1). Colorless liquid. **^{1}H NMR** (400 MHz, Chloroform-*d*) δ 10.0 (s, 1H), 9.0 – 8.5 (m, 2H), 8.2 (dd, *J* = 8.3, 1.7 Hz, 1H), 7.7 – 7.3 (m, 3H), 6.2 – 5.8 (m, 1H), 5.8 – 5.4 (m, 1H), 4.0 (p, *J* = 6.6 Hz, 1H), 3.9 – 3.7 (m, 1H), 3.7 – 3.5 (m,

1H), 3.5 – 3.2 (m, 2H), 2.6 – 2.2 (m, 2H), 1.4 (d, *J* = 6.4 Hz, 3H). **^{13}C NMR** (101 MHz, Chloroform-*d*) δ 169.1, 148.1, 138.4, 137.5, 136.4, 134.3, 128.0, 127.4, 124.8, 121.7, 121.7, 116.4, 61.2 (q, *J* = 3.7 Hz), 41.6, 34.6 (q, *J* = 28.1 Hz), 21.4. **^{19}F NMR** (376 MHz, Chloroform-*d*) δ -64.7. **HRMS**: calculated for : $C_{18}H_{19}F_3N_2O_2$ [M+Na$^+$] 375.1291, found 375.1285.

(*E*)-*N*-(quinolin-8-yl)-5-(4,4,4-trifluorobutoxy)hex-3-enamide

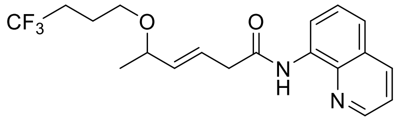

Compound **57** was isolated in 53 % yield with CuCl for 10 h. Flash silica gel chromatography (PE : EA = 3:1). Colorless liquid. **^{1}H NMR** (400 MHz, Chloroform-*d*) δ 10.0 (s, 1H), 9.0 – 8.5 (m, 2H), 8.2 (dd, *J* = 8.3, 1.7 Hz, 1H), 7.7 – 7.3 (m, 3H), 6.1 – 5.8 (m, 1H), 5.8 – 5.5 (m, 1H), 3.9 (p, *J* = 6.6 Hz, 1H), 3.8 – 3.5 (m, 1H), 3.5 – 3.4 (m, 1H), 3.3 (dd, *J* = 7.2, 1.3 Hz, 2H), 2.3 – 2.0 (m, 2H), 1.9 – 1.8 (m, 2H), 1.4 (d, *J* = 6.3 Hz, 3H). **^{13}C NMR** (101 MHz, Chloroform-*d*) δ 169.2, 148.1, 138.5, 138.0, 136.4, 134.3, 128.0, 127.4, 127.3 (q, *J* = 275.9 Hz), 124.4, 121.7, 116.4, 76.3, 66.4, 41.6, 30.9 (q, *J* = 28.9 Hz), 22.7 (q, *J* = 3.0 Hz), 21.4. **^{19}F NMR** (376 MHz, Chloroform-*d*) δ -66.3. **HRMS**: calculated for : $C_{19}H_{21}F_3N_2O_2$ [M+Na$^+$] 389.1447, found 389.1442.

(*E*)-*N*-(quinolin-8-yl)-4-(tetrahydrofuran-2-yl)but-3-enamide

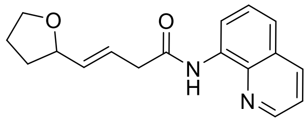

Compound **58** was isolated in 56 % yield with Cu_2O for 10 h. Flash silica gel chromatography (PE : EA = 4:1). Colorless liquid. **^{1}H NMR** (400 MHz, Chloroform-*d*) δ 10.0 (s, 1H), 8.9 – 8.7 (m, 2H), 8.2 (dd, *J* = 8.2, 1.7 Hz, 1H), 7.6 – 7.4 (m, 3H), 6.1 – 5.8 (m, 2H), 4.4 (q, *J* = 6.7 Hz, 1H), 4.0 (td, *J* = 7.8, 6.2 Hz, 1H), 3.8 (td, *J* = 7.9, 6.2 Hz, 1H), 3.3 (d, *J* = 7.1 Hz, 2H), 2.2 – 2.1 (m, 1H), 2.1 – 1.9 (m, 2H), 1.9 – 1.8 (m, 1H). **^{13}C NMR** (101 MHz, Chloroform-*d*) δ 169.4, 148.1, 138.5, 137.0, 136.4, 134.4, 128.0, 127.4, 123.6, 121.6, 116.5, 79.1, 68.2, 41.7, 32.1, 25.9. **HRMS**: calculated for : $C_{17}H_{18}N_2O_2$ [M+Na$^+$] 305.1260, found 305.1261.

(*E*)-*N*-(quinolin-8-yl)-4-(tetrahydro-2*H*-pyran-2-yl) but-3-enamide

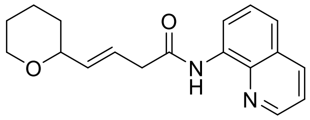

Compound **59** was isolated in 66 % yield with Cu_2O for 10 h. Flash silica gel chromatography (PE : EA = 4:1). Colorless liquid. **^{1}H NMR** (400 MHz, Chloroform-*d*) δ 10.0 (s, 1H), 9.2 – 8.6 (m, 2H), 8.1 (dd, *J* = 8.3, 1.7 Hz, 1H), 7.8 – 7.3 (m, 3H), 6.0 (dtd, *J* = 15.5, 7.1, 1.2 Hz, 1H), 5.8 (ddt, *J* = 15.6, 5.7, 1.3 Hz, 1H), 4.2 – 4.0 (m, 1H), 4.0 – 3.9 (m, 1H), 3.7 – 3.4 (m, 1H), 3.4 – 3.1 (m, 2H), 2.0 – 1.7 (m, 3H), 1.7 – 1.5 (m, 3H). **^{13}C NMR** (101 MHz, Chloroform-*d*) δ 169.4, 148.1, 138.5, 137.1, 136.3, 134.4, 127.9, 127.4, 123.3, 121.6, 121.6, 116.4, 77.7, 68.4, 41.8, 32.0, 25.9, 23.4. **HRMS**: calculated for : $C_{18}H_{20}N_2O_2$ [M+Na$^+$] 319.1417, found 319.1418.

(*E*)-*N*-(quinolin-8-yl)-4-(2-oxaspiro[5.5]undecan-3-yl)but-3-enamide

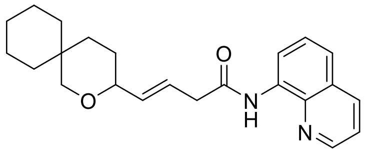

Compound **60** was isolated in 72 % yield with Cu_2O for 10 h. Flash silica gel chromatography (PE : EA = 4:1). Colorless liquid. **^{1}H NMR** (400 MHz, Chloroform-*d*) δ 10.0 (s, 1H), 8.8 (dt, *J* = 5.4, 1.7 Hz, 2H), 8.1 (dd, *J* = 8.3, 1.7 Hz, 1H), 7.8 – 7.4 (m, 3H), 6.2 – 5.9 (m, 1H), 5.9 – 5.8 (m, 1H), 3.8 (ddd, *J* = 14.0, 8.9, 2.8 Hz, 2H), 3.3 (d, *J* = 7.0 Hz, 2H), 3.2 (d, *J* = 11.3 Hz, 1H), 1.9 – 1.3 (m, 12H), 1.2 – 1.1 (m, 2H). **^{13}C NMR** (101 MHz, Chloroform-*d*) δ 169.4, 148.1, 138.5, 137.1, 136.3, 134.4, 127.9, 127.4, 123.4, 121.6, 121.6, 116.4, 78.2, 76.5, 41.8, 36.6, 34.1, 32.0, 31.2, 27.6, 26.8, 21.6, 21.5. **HRMS**: calculated for : $C_{23}H_{28}N_2O_2$ [M+Na$^+$] 387.2043, found 387.2038.

(*E*)-4-(2,3-dihydrobenzofuran-2-yl)-*N*-(quinolin-8-yl)but-3-enamide

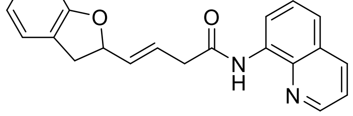

Compound **61** was isolated in 62 % yield with Cu_2O for 10 h. Flash silica gel chromatography (PE : EA = 4:1). Colorless liquid. **^{1}H NMR** (400 MHz, Chloroform-*d*) δ 10.0 (s, 1H), 9.0 – 8.6 (m, 2H), 8.2 (dd, *J* = 8.3, 1.7 Hz, 1H), 7.7 – 7.4 (m, 3H), 7.2 – 7.0 (m, 2H), 6.9 – 6.7 (m, 2H), 6.2 – 6.1 (m, 1H), 6.1 – 6.0 (m, 1H), 5.6 – 5.1 (m, 1H), 3.5 – 3.3 (m, 3H), 3.2 – 3.1 (m, 1H). **^{13}C NMR** (101 MHz, Chloroform-*d*) δ 168.9,

159.4, 148.2, 138.5, 136.4, 134.7, 134.3, 128.1, 128.0, 127.4, 126.6, 125.9, 124.9, 121.7, 121.7, 120.6, 116.5, 109.5, 82.9, 41.5, 36.1. **HRMS**: calculated for : $C_{21}H_{18}N_2O_2$ [M+Na$^+$] 353.1260, found 353.1259.

(*E*)-4-(chroman-2-yl)-*N*-(quinolin-8-yl)but-3-enamide

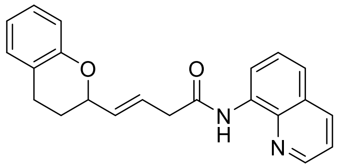

Compound **62** was isolated in 72 % yield with Cu_2O for 10 h. Flash silica gel chromatography (PE : EA = 4:1). Colorless liquid. **^{1}H NMR** (400 MHz, Chloroform-*d*) δ 10.0 (s, 1H), 8.9 – 8.6 (m, 2H), 8.2 (dd, *J* = 8.2, 1.7 Hz, 1H), 7.6 – 7.4 (m, 3H), 7.1 – 7.0 (m, 2H), 6.8 (t, *J* = 7.3 Hz, 2H), 6.2 – 6.1 (m, 1H), 6.1 – 5.9 (m, 1H), 4.8 – 4.5 (m, 1H), 3.4 (d, *J* = 7.1 Hz, 2H), 3.0 – 2.7 (m, 2H), 2.3 – 2.1 (m, 1H), 2.1 – 1.9 (m, 1H). **^{13}C NMR** (101 MHz, Chloroform-*d*) δ 169.1, 154.5, 148.2, 138.5, 136.4, 135.1, 134.4, 129.6, 128.0, 127.5, 127.4, 125.1, 121.8, 121.7, 121.7, 120.3, 116.9, 116.5, 75.8, 41.7, 27.8, 24.4. **HRMS**: calculated for : $C_{22}H_{20}N_2O_2$ [M+Na$^+$] 367.1417, found 367.1416.

(*E*)-*N*-(quinolin-8-yl)-4-(6-(trifluoromethyl)chroman-2-yl)but-3-enamide

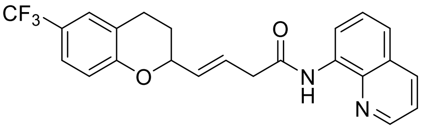

Compound **63** was isolated in 75 % yield with Cu_2O for 10 h. Flash silica gel chromatography (PE : EA = 4:1). Colorless liquid. **^{1}H NMR** (400 MHz, Chloroform-*d*) δ 10.0 (s, 1H), 9.2 – 8.6 (m, 2H), 8.2 (dd, *J* = 8.3, 1.7 Hz, 1H), 7.8 – 7.4 (m, 3H), 7.4 – 7.3 (m, 2H), 6.9 (d, *J* = 8.4 Hz, 1H), 6.3 – 6.1 (m, 1H), 6.0 – 5.8 (m, 1H), 5.0 – 4.5 (m, 1H), 3.4 (d, *J* = 7.1 Hz, 2H), 3.1 – 2.7 (m, 2H), 2.3 – 2.2 (m, 1H), 2.1 – 1.9 (m, 1H). **^{13}C NMR** (101 MHz, Chloroform-*d*) δ 168.9, 157.1, 148.2, 138.4, 136.4, 134.3, 134.2, 128.0, 127.4, 126.9 (q, *J* = 3.8 Hz), 125.6, 124.6 (q, *J* = 3.7 Hz), 122.0, 121.7, 121.7, 117.2, 116.5, 76.2, 41.6, 27.3, 24.2. **^{19}F NMR** (377 MHz, Chloroform-*d*) δ -61.4. **HRMS**: calculated for : $C_{23}H_{19}F_3N_2O_2$ [M+Na$^+$] 435.1291, found 435.1289.

(*E*)-*N*-(quinolin-8-yl)-4-(7-(trifluoromethyl)-2,3,4,5-tetrahydrobenzo[*b*]oxepin-2-yl)but-3-enamide

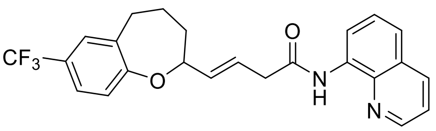

Compound **64** was isolated in 71 % yield with Cu_2O for 10 h. Flash silica gel chromatography (PE : EA = 4:1). Yellow liquid. **^{1}H NMR** (400 MHz, Chloroform-*d*) δ 10.1 (s, 1H), 9.1 – 8.6 (m, 2H), 8.2 (dd, *J* = 8.3, 1.7 Hz, 1H), 7.6 – 7.4 (m, 3H), 7.4 (d, *J* = 8.4 Hz, 2H), 7.1 (d, *J* = 8.0 Hz, 1H), 6.3 – 6.1 (m, 1H), 6.1 – 5.9 (m, 1H), 4.3 (dd, *J* = 10.8, 5.6 Hz, 1H), 3.4 (d, *J* = 7.1 Hz, 2H), 3.1 – 2.7 (m, 2H), 2.3 – 1.9 (m, 3H), 1.7 – 1.5 (m, 1H). **^{13}C NMR** (101 MHz, Chloroform-*d*) δ 169.2, 161.7, 148.2, 138.5, 136.4, 136.3, 136.2, 134.3, 128.0, 127.4, 124.7 (q, *J* = 3.8 Hz), 123.7, 122.2, 121.7, 121.7, 116.4, 83.0, 41.7, 37.2, 33.7, 25.3. **^{19}F NMR** (377 MHz, Chloroform-*d*) δ -61.7. **HRMS**: calculated for : $C_{24}H_{21}F_3N_2O_2$ [M+Na$^+$] 449.1447, found 449.1442.

(*E*)-*N*-(quinolin-8-yl)-4-(8-(trifluoromethyl)-3,4,5,6-tetrahydro-2*H*-benzo[*b*]oxocin-2-yl)but-3-enamide

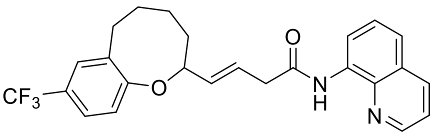

Compound **65** was isolated in 74 % yield with Cu_2O for 10 h. Flash silica gel chromatography (PE : EA = 4:1). Yellow liquid. **^{1}H NMR** (400 MHz, Chloroform-*d*) δ 10.1 (s, 1H), 9.0 – 8.6 (m, 2H), 8.2 (dd, *J* = 8.3, 1.7 Hz, 1H), 7.7 – 7.4 (m, 5H), 7.2 (d, *J* = 8.1 Hz, 1H), 6.3 – 6.1 (m, 1H), 6.1 – 5.8 (m, 1H), 4.8 – 4.5 (m, 1H), 3.7 – 3.3 (m, 2H), 3.1 – 2.9 (m, 1H), 2.9 – 2.4 (m, 1H), 2.0 – 1.8 (m, 2H), 1.8 – 1.6 (m, 2H), 1.6 – 1.4 (m, 2H). **^{13}C NMR** (101 MHz, Chloroform-*d*) δ 169.2, 159.3, 148.1, 138.5, 138.1, 136.4, 135.6, 134.4, 128.0, 127.4, 127.0 (q, *J* = 3.7 Hz), 124.7 (q, *J* = 3.8 Hz), 124.2, 122.6, 121.7, 121.7, 116.5, 85.2, 41.8, 32.0, 31.2, 30.4, 23.4. **^{19}F NMR** (377 MHz, Chloroform-*d*) δ -61.7. **HRMS**: calculated for : $C_{25}H_{23}F_3N_2O_2$ [M+Na$^+$] 463.1604, found 463.1602.

(*E*)-*N*-(quinolin-8-yl)-4-(9-(trifluoromethyl)-2,3,4,5,6,7-hexahydrobenzo[*b*]oxonin-2-yl)but-3-enamide

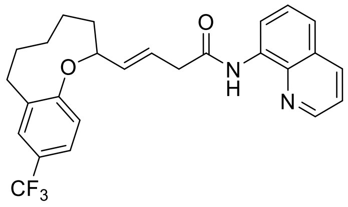

Compound **66** was isolated in 32 % yield with Cu_2O for 10 h. Flash silica gel chromatography (PE : EA = 4:1). White solid. **^{1}H NMR** (400 MHz, Chloroform-*d*) δ 10.1 (s, 1H), 9.0 – 8.5 (m, 2H), 8.2 (dd, *J* = 8.3, 1.7 Hz, 1H), 7.6 – 7.5 (m, 2H), 7.5 (dd, *J* = 8.3, 4.2 Hz, 1H), 7.4 – 7.3 (m, 2H), 7.2 (d, *J* = 8.4 Hz, 1H), 6.5 – 5.8 (m, 2H), 4.6 (dt, *J* = 9.1, 4.3 Hz, 1H), 3.6 – 3.3 (m, 2H), 3.3 – 2.8 (m, 1H), 2.7 – 2.3 (m, 1H), 2.0 – 1.7 (m, 4H), 1.7 – 1.5 (m, 2H), 1.5 – 1.4 (m, 1H), 1.2 – 1.0 (m, 1H). **^{13}C NMR** (101 MHz, Chloroform-*d*) δ 169.2, 162.5, 148.2, 138.5, 137.4, 136.5, 135.1, 134.3, 128.0, 127.5, 124.8 (q, *J* = 3.8 Hz), 122.9, 121.7, 121.7, 119.2, 116.5, 83.8, 41.7, 34.2, 28.7, 28.6, 22.7, 21.2. **^{19}F NMR** (377 MHz, Chloroform-*d*) δ -61.7. **HRMS**: calculated for : $C_{26}H_{25}F_3N_2O_2$ [M+Na$^+$] 477.1760, found 477.1752.

(*E*)-*N*-(quinolin-8-yl)-4-(10-(trifluoromethyl)-3,4,5,6,7,8-hexahydro-2*H*-benzo[*b*]oxecin-2-yl)but-3-enamide

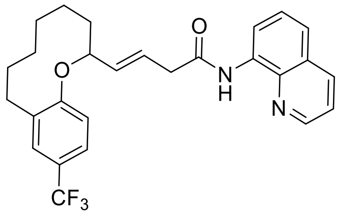

Compound **67** was isolated in 43 % yield with Cu_2O for 10 h. Flash silica gel chromatography (PE : EA = 4:1). Yellow solid. **^{1}H NMR** (400 MHz, Chloroform-*d*) δ 10.0 (s, 1H), 8.9 – 8.6 (m, 2H), 8.2 (dd, *J* = 8.3, 1.7 Hz, 1H), 7.6 – 7.5 (m, 2H), 7.4 (dd, *J* = 8.3, 4.2 Hz, 1H), 7.3 (d, *J* = 2.4 Hz, 1H), 7.3 – 7.2 (m, 1H), 7.1 (d, *J* = 8.5 Hz, 1H), 6.3 – 5.8 (m, 2H), 5.0 – 4.5 (m, 1H), 3.7 – 3.3 (m, 2H), 3.3 – 2.9 (m, 1H), 2.5 – 2.3 (m, 1H), 2.0 – 1.8 (m, 3H), 1.7 – 1.6 (m, 2H), 1.6 – 1.4 (m, 4H), 1.1 – 0.6 (m, 1H). **^{13}C NMR** (101 MHz, Chloroform-*d*) δ 169.1, 160.0, 148.1, 138.5, 136.4, 136.3, 134.3, 133.3, 128.0, 127.4, 127.2 (q, *J* = 3.6 Hz), 124.4 (q, *J* = 3.9 Hz), 123.4, 121.7, 121.7, 116.5, 113.2, 81.8, 41.7, 29.8, 27.2, 26.2, 25.3, 25.0. **^{19}F NMR** (377 MHz, Chloroform-*d*) δ -61.7. **HRMS**: calculated for : $C_{27}H_{27}F_3N_2O_2$ [M+Na$^+$] 491.1917, found 491.1913.

(*E*)-4-(12-oxo-2,11-dioxa-1(1,3)-benzenacyclododecaphane-3-yl)-*N*-(quinolin-8-yl)but-3-enamide

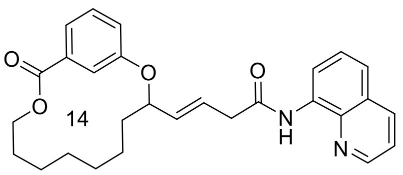

Compound **68** was isolated in 53 % yield with Cu_2O for 10 h. Flash silica gel chromatography (PE : EA = 4:1). Colorless liquid. **^{1}H NMR** (400 MHz, Chloroform-*d*) δ 10.0 (s, 1H), 9.0 – 8.6 (m, 2H), 8.2 (dd, *J* = 8.3, 1.7 Hz, 1H), 7.7 (t, *J* = 2.1 Hz, 1H), 7.7 – 7.6 (m, 1H), 7.6 – 7.5 (m, 2H), 7.4 (dd, *J* = 8.3, 4.2 Hz, 1H), 7.3 (t, *J* = 7.9 Hz, 1H), 7.2 – 7.0 (m, 1H), 6.3 – 6.1 (m, 1H), 6.1 – 5.8 (m, 1H), 5.0 – 4.6 (m, 1H), 4.6 – 4.4 (m, 1H), 4.3 – 4.0 (m, 1H), 3.4 (dd, *J* = 7.1, 1.2 Hz, 2H), 2.1 – 1.9 (m, 2H), 1.9 – 1.5 (m, 8H), 1.5 – 1.2 (m, 2H). **^{13}C NMR** (101 MHz, Chloroform-*d*) δ 169.0, 166.0, 157.2, 148.1, 138.5, 136.4, 134.4, 134.3, 131.6, 129.9, 127.9, 127.4, 126.1, 122.3, 121.7, 121.7, 121.4, 116.5, 113.5, 77.3, 66.9, 41.8, 29.0, 28.7, 28.5, 26.1, 25.8, 24.4. **HRMS**: calculated for : $C_{28}H_{30}N_2O_4$ [M+Na$^+$] 481.2098, found 481.2097.

(*E*)-4-(13-oxo-2,12-dioxa-1(1,3)-benzenacyclotridecaphane-3-yl)-*N*-(quinolin-8-yl)but-3-enamide

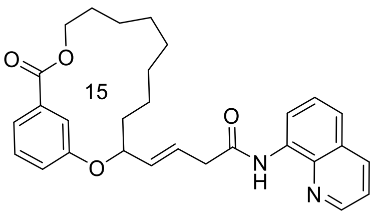

Compound **69** was isolated in 66 % yield with Cu_2O for 10 h. Flash silica gel chromatography (PE : EA = 4:1). Colorless liquid. **^{1}H NMR** (400 MHz, Chloroform-*d*) δ 10.0 (s, 1H), 8.9 – 8.5 (m, 2H), 8.2 (dd, *J* = 8.3, 1.6 Hz, 1H), 7.7 (d, *J* = 7.6 Hz, 1H), 7.6 – 7.5 (m, 3H), 7.4 (dd, *J* = 8.3, 4.2 Hz, 1H), 7.3 (t, *J* = 7.9 Hz, 1H), 7.2 (dd, *J* = 8.2, 2.5 Hz, 1H), 6.4 – 5.8 (m, 2H), 4.9 – 4.7 (m, 1H), 4.6 – 4.5 (m, 1H), 4.3 – 4.0 (m, 1H), 3.4 (d, *J* = 7.0 Hz, 2H), 2.3 – 2.1 (m, 1H), 1.9 – 1.7 (m, 2H), 1.7 – 1.3 (m, 11H). **^{13}C NMR** (101 MHz, Chloroform-*d*) δ 169.0, 166.3, 157.9, 148.1, 138.4, 136.4, 134.7, 134.3, 131.7, 129.7, 127.9, 127.4, 125.2, 123.5, 122.6, 121.7, 121.6, 116.4, 114.5, 79.6, 65.9, 41.8, 31.5, 28.1, 26.9, 26.9, 26.8, 26.1, 23.4. **HRMS**: calculated for : $C_{29}H_{32}N_2O_4$ [M+Na$^+$] 495.2254, found 495.2247.

(*E*)-4-(14-oxo-2,13-dioxa-1(1,3)-benzenacyclotetradecaphane-3-yl)-*N*-(quinolin-8-yl)but-3-enamide

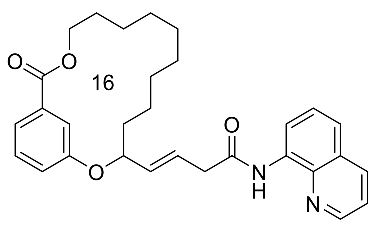

Compound **70** was isolated in 68 % yield with Cu_2O for 10 h. Flash silica gel chromatography (PE : EA = 4:1). White solid. **^{1}H NMR** (400 MHz, Chloroform-*d*) δ 10.0 (s, 1H), 8.9 – 8.6 (m, 2H), 8.1 (dd, *J* = 8.3, 1.7 Hz, 1H), 7.7 (dd, *J* = 7.6, 1.4 Hz, 1H), 7.6 – 7.6 (m, 1H), 7.6 – 7.5 (m, 2H), 7.4 (dd, *J* = 8.3, 4.2 Hz, 1H), 7.3 (t, *J* = 7.9 Hz, 1H), 7.1 (dd, *J* = 8.1, 2.5 Hz, 1H), 6.4 – 5.7 (m, 2H), 5.0 – 4.7 (m, 1H), 4.6 – 4.1 (m, 2H), 3.4 (dd, *J* = 7.2, 1.2 Hz, 2H), 2.3 – 2.0 (m, 1H), 2.0 – 1.7 (m, 2H), 1.7 – 1.2 (m, 13H). **^{13}C NMR** (101 MHz, Chloroform-*d*) δ 169.0, 166.2, 157.9, 148.1, 138.4, 136.4, 134.8, 134.3, 131.7, 129.7, 127.9, 127.4, 125.5, 123.7, 122.8, 121.7, 121.6, 116.4, 114.3, 78.7, 65.4, 41.7, 32.0, 27.7, 27.5, 27.4, 27.3, 27.2, 25.3, 24.2. **HRMS**: calculated for : $C_{30}H_{34}N_2O_4$ [M+Na$^+$] 509.2411, found 509.2409.

(*E*)-4-(15-oxo-2,14-dioxa-1(1,3)-benzenacyclopentadecaphane-3-yl)-*N*-(quinolin-8-yl)but-3-enamide

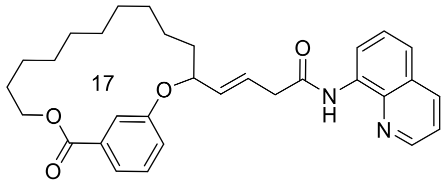

Compound **71** was isolated in 73 % yield with Cu_2O for 10 h. Flash silica gel chromatography (PE : EA = 4:1). White solid. **^{1}H NMR** (400 MHz, Chloroform-*d*) δ 10.0 (s, 1H), 9.0 – 8.6 (m, 2H), 8.1 (dd, *J* = 8.3, 1.7 Hz, 1H), 7.7 (d, *J* = 7.5 Hz, 1H), 7.6 (d, *J* = 2.2 Hz, 1H), 7.6 – 7.5 (m, 2H), 7.4 (dd, *J* = 8.3, 4.2 Hz, 1H), 7.3 – 7.1 (m, 2H), 6.2 – 5.8 (m, 2H), 4.6 (q, *J* = 6.4 Hz, 1H), 4.5 – 4.2 (m, 2H), 3.4 (d, *J* = 6.8 Hz, 2H), 2.1 – 1.9 (m, 1H), 1.8 – 1.6 (m, 3H), 1.7 – 1.1 (m, 16H). **^{13}C NMR** (101 MHz, Chloroform-*d*) δ 169.0, 166.2, 158.9, 148.1, 138.4, 136.3, 135.8, 134.3, 131.8, 129.6, 127.9, 127.4, 124.5, 123.5, 123.3, 121.6, 121.6, 117.7, 116.4, 81.5, 65.1, 41.7, 34.4, 28.3, 27.9, 27.7, 27.6, 27.4, 27.2, 25.2, 24.4. **HRMS**: calculated for : $C_{31}H_{36}N_2O_4$ [M+Na$^+$] 523.2567, found 523.2559.

(*E*)-4-(16-oxo-2,15-dioxa-1(1,3)-benzenacyclohexadecaphane-3-yl)-*N*-(quinolin-8-yl)but-3-enamide

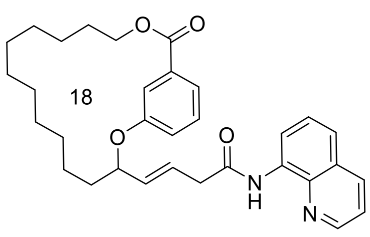

Compound **72** was isolated in 74 % yield with Cu_2O for 10 h. Flash silica gel chromatography (PE : EA = 4:1). White solid. **^{1}H NMR** (400 MHz, Chloroform-*d*) δ 10.0 (s, 1H), 9.1 – 8.5 (m, 2H), 8.2 (dd, *J* = 8.2, 1.7 Hz, 1H), 7.7 – 7.6 (m, 2H), 7.6 – 7.5 (m, 2H), 7.4 (dd, *J* = 8.3, 4.2 Hz, 1H), 7.3 – 7.2 (m, 1H), 7.2 (dd, *J* = 8.2, 2.6 Hz, 1H), 6.2 – 5.7 (m, 2H), 4.8 (q, *J* = 5.9 Hz, 1H), 4.4 – 4.2 (m, 2H), 3.4 (d, *J* = 7.0 Hz, 2H), 2.0 – 1.9 (m, 1H), 1.8 – 1.7 (m, 3H), 1.6 – 1.2 (m, 16H). **^{13}C NMR** (101 MHz, Chloroform-*d*) δ 169.0, 166.3, 158.5, 148.1, 138.4, 136.4, 135.7, 134.3, 131.8, 129.6, 127.9, 127.4, 124.7, 122.6, 121.7, 121.6, 121.3, 116.8, 116.4, 79.5, 65.2, 41.8, 34.4, 28.5, 28.4, 28.3, 27.7, 27.6, 27.6, 27.4, 25.7, 24.7. **HRMS**: calculated for : $C_{32}H_{38}N_2O_4$ [M+Na$^+$] 537.2724, found 537.2720.

(*E*)-4-(15-oxo-2,14-dioxa-1(1,3)-benzenacycloheptadecaphane-3-yl)-*N*-(quinolin-8-yl)but-3-enamide

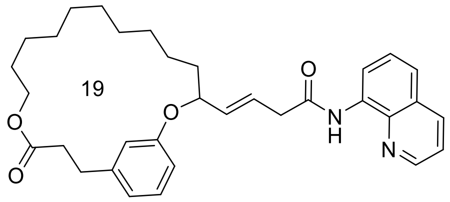

Compound **73** was isolated in 76 % yield with Cu_2O for 10 h. Flash silica gel chromatography (PE : EA = 4:1). Colorless liquid. **^{1}H NMR** (400 MHz, Chloroform-*d*) δ 10.0 (s, 1H), 9.1 – 8.5 (m, 2H), 8.1 (dd, *J* = 8.3, 1.7 Hz, 1H), 7.6 – 7.3 (m, 3H), 7.2 – 7.0 (m, 1H), 6.9 – 6.5 (m, 3H), 6.1 – 5.5 (m, 2H), 4.9 – 4.4 (m, 1H), 4.2 – 3.8 (m, 2H), 3.3 (d, *J* = 7.0 Hz, 2H), 2.9 (t, *J* = 6.5 Hz, 2H), 2.7 – 2.5 (m, 2H), 2.0 – 1.9 (m, 1H), 1.8 – 1.7 (m, 1H), 1.6 – 1.5 (m, 4H), 1.4 – 1.3 (m, 4H), 1.3 – 1.0 (m, 8H). **^{13}C NMR** (101 MHz, Chloroform-*d*) δ 172.9, 169.1, 158.6, 148.1, 142.0, 138.4, 136.4, 136.3, 134.3, 129.2, 127.9, 127.3, 124.0, 121.6, 120.8, 116.4, 115.8, 113.1, 77.2, 64.4, 41.7, 35.2, 35.1, 30.6, 28.2, 28.0, 27.7, 27.6, 27.2, 27.1, 25.2, 23.9. **HRMS**: calculated for : $C_{33}H_{40}N_2O_4$ [M+Na$^+$] 551.2880, found 551.2875.

(*E*)-4-(16-oxo-2,15-dioxa-1(1,3)-benzenacyclooctadecaphane-3-yl)-*N*-(quinolin-8-yl)but-3-enamide

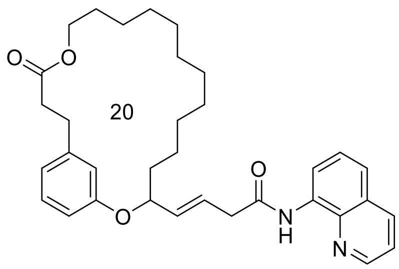

Compound **74** was isolated in 78 % yield with Cu_2O for 10 h. Flash silica gel chromatography (PE : EA = 4:1). Colorless liquid. **^{1}H NMR** (400 MHz, Chloroform-*d*) δ 10.0 (s, 1H), 9.0 – 8.5 (m, 2H), 8.1 (dd, *J* = 8.2, 1.6 Hz, 1H), 7.7 – 7.3 (m, 3H), 7.1 (t, *J* = 7.8 Hz, 1H), 6.9 – 6.5 (m, 3H), 6.2 – 5.7 (m, 2H), 4.7 (q, *J* = 6.2 Hz, 1H), 4.2 – 3.8 (m, 2H), 3.3 (d, *J* = 7.0 Hz, 2H), 2.9 (t, *J* = 7.2 Hz, 2H), 2.6 (t, *J* = 7.3 Hz, 2H), 2.0 – 1.9 (m, 1H), 1.8 – 1.7 (m, 1H), 1.6 – 1.5 (m, 4H), 1.4 – 1.2 (m, 14H). **^{13}C NMR** (101 MHz, Chloroform-*d*) δ 172.8, 169.1, 158.5, 148.1, 142.0, 138.4, 136.3, 136.1, 134.3, 129.4, 127.9, 127.3, 124.3, 121.6, 121.6, 120.8, 116.4, 115.6, 114.1, 77.7, 64.5, 41.7, 35.9, 34.6, 31.0, 28.3, 28.2, 28.0, 27.9, 27.7, 27.7, 25.3, 24.6. **HRMS**: calculated for : $C_{34}H_{42}N_2O_4$ [M+Na$^+$] 565.3037, found 565.3028.

5. Synthetic utilities

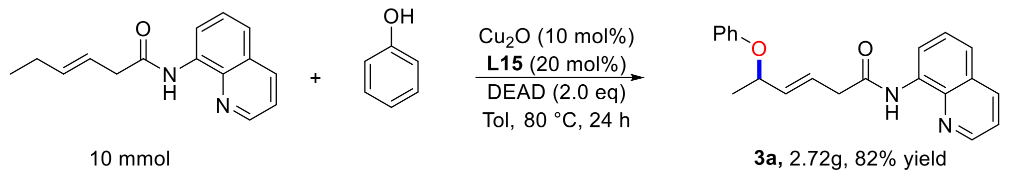

A mixture of amide (10 mmol, 1.0 equiv), Cu_2O (1.0 mmol, 0.1 equiv), L15 (2.0 mmol, 0.2 equiv), DEAD (20.0 mmol, 2.0 equiv) and phenol (30.0 mmol, 3.0 equiv) in Toluene (20 mL) in a 50 mL glass vial (sealed with PTFE cap) was heated at 80 °C for indicated time. The reaction progress was monitored by thin layer chromatography. Upon completion, the crude mixture was concentrated under reduced pressure and the residue was purified by silica gel column chromatography (PE/EA = 4:1) to afford **3a** (2.72 g, 82% yield).

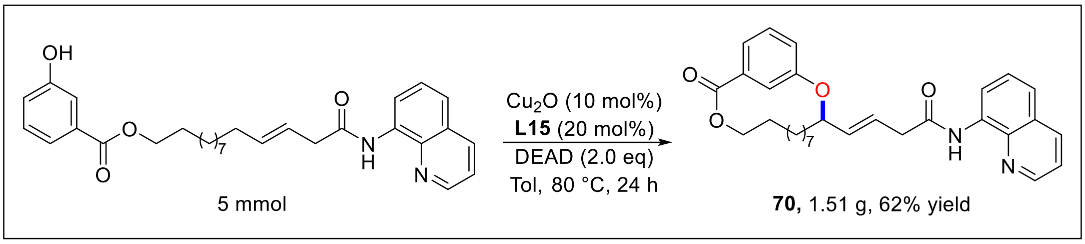

A mixture of amide (5 mmol, 1.0 equiv), Cu_2O (0.5 mmol, 0.1 equiv), L15 (1.0 mmol, 0.2 equiv), and DEAD (10.0 mmol, 2.0 equiv) in Toluene (10 mL) in a 50 mL glass vial (sealed with PTFE cap) was heated at 80 °C for indicated time. The reaction progress was monitored by thin layer chromatography. Upon completion, the crude mixture was concentrated under reduced pressure and the residue was purified by silica gel column chromatography (PE/EA = 4:1) to afford **70** (1.51 g, 62% yield).

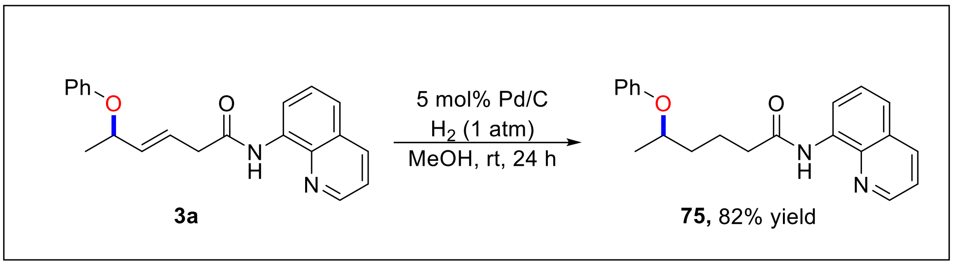

A 10 mL tube equipped with a magnetic stirring bar was charged with Pd/C (2.6 mg, 5 wt%, 10.0 mol%) and **3a** (66.4 mg, 0.2 mmol, 1.0 eq) in MeOH (5.0 mL). The reaction mixture was flushed with H_2 (3x) and stirred at rt with a H_2 balloon for 24 hours. After cooling down, the reaction mixture was filtered through a short pad of silica gel eluting with dichloromethane and concentrated. The solvent was removed under reduced pressure and the residue was purified by silica gel column chromatography (PE/EA = 4:1) to obtain **75** (54.7 mg, 82% yield).

5-phenoxy-*N*-(quinolin-8-yl) hexanamide

Colorless liquid. **^{1}H NMR** (400 MHz, Chloroform-*d*) δ 9.8 (s, 1H), 9.1 – 8.6 (m, 2H), 8.2 (dd, *J* = 8.3, 1.7 Hz, 1H), 7.6 – 7.5 (m, 2H), 7.4 (dd, *J* = 8.3, 4.2 Hz, 1H), 7.3 – 7.2 (m, 2H), 7.0 – 6.8 (m, 3H), 4.6 – 4.2 (m, 1H), 2.6 (t, *J* = 7.3 Hz, 2H), 2.1 – 1.8 (m, 3H), 1.8 – 1.7 (m, 1H), 1.3 (d, *J* = 6.1 Hz, 3H). **^{13}C NMR** (101 MHz, Chloroform-*d*) δ 171.5, 158.1, 148.1, 138.4, 136.4, 134.5, 129.5, 128.0, 127.5, 121.6, 121.4, 120.6, 116.5, 116.0, 73.6, 37.9, 36.0, 21.8, 19.8. **HRMS**: calculated for : $C_{21}H_{22}N_2O_2$ [M+Na$^+$] 357.1573, found 357.1572.

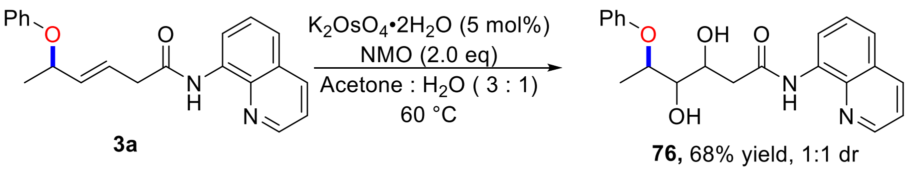

To a solution of **3a** (66.4 mg, 0.2 mmol, 1.0 eq) in Acetone/H_2O (2.0 mL, 3:1) was added $K_2OsO_4·2H_2O$ (3.68 mg, 0.01 mmol, 0.05 eq) and NMO (46.8 mg, 0.4 mmol, 2.0 eq) at 60 °C. The reaction progress was monitored by thin layer chromatography. Upon completion, the reaction mixture was washed by NaCl saturated solution (10 mL × 3). The organic phase was dried by anhydrous Na_2SO_4. The solvent was removed under reduced pressure and the residue was purified by flash column chromatography (PE/EA = 1:1) to obtain **76** (49.8 mg, 68% yield).

3,4-dihydroxy-5-phenoxy-*N*-(quinolin-8-yl)hexanamide

Colorless liquid. **^{1}H NMR** (400 MHz, Chloroform-*d*) δ 10.1 (d, *J* = 10.6 Hz, 1H), 8.8 – 8.8 (m, 1H), 8.8 – 8.6 (m, 1H), 8.2 – 8.0 (m, 1H), 7.6 – 7.5 (m, 2H), 7.4 (ddd, *J* = 8.3, 4.2, 2.7 Hz, 1H), 7.3 (ddd, *J* = 8.4, 7.4, 2.3 Hz, 2H), 7.0 – 6.7 (m, 3H), 4.6 (p, *J* = 6.2 Hz, 0.5H), 4.6 – 4.5 (m, 1H), 4.4 (dd, *J* = 8.3, 4.1 Hz, 0.5H), 4.1 (s, 0.4H), 3.8 (s, 0.4H), 3.7 (q, *J* = 3.0 Hz, 0.5H), 3.6 – 3.5 (m, 0.5H), 3.2 – 3.1 (m, 1H), 3.0 (ddd, *J* = 15.4, 8.8, 4.8 Hz, 1.0 H), 2.9 (d, *J* = 3.9 Hz, 0.3H), 2.8 (dd, *J* = 4.9, 3.7 Hz, 0.5H), 2.8 (d, *J* = 3.5 Hz, 0.2H), 1.8 (s, 1H), 1.4 (dd, *J* = 18.7, 6.2 Hz, 3H). **^{13}C NMR** (101 MHz, Chloroform-*d*) δ 171.0, 157.4, 148.4, 138.4, 136.4, 134.2, 129.6, 128.0, 127.3, 122.1, 121.7, 121.5, 117.0, 116.3, 76.2, 75.4, 68.5, 41.8, 16.4. **HRMS**: calculated for : $C_{21}H_{22}N_2O_4$ [M+Na$^+$] 389.1472, found 389.1471.

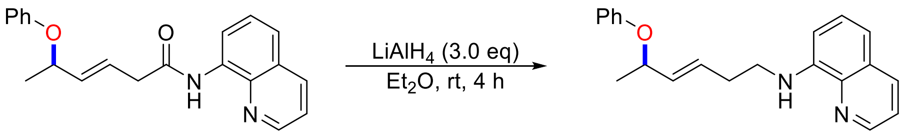

To a solution of $LiAlH_4$ (22.8 mg, 0.60 mmol, 3.0 equiv) in anhydrous Et_2O (2.5 mL) at 0 °C was added dropwise a solution of compound **3a** (66.4 mg, 0.20 mmol, 1.0 equiv) in Et_2O (1.5 mL). Then the mixture was stirred for 4 h at the same temperature. Once completion, an aqueous solution of NaOH (10 mL, 10% in water) was added

dropwise into the reaction mixture. The mixture was extracted with Et_2O (15 mL × 3). The combined organic phase was dried over $MgSO_4$ and concentrated *in vacuo*. And the residue was purified by column chromatography using eluents (PE/EA = 2:1) to afford the desired product **77** as yellow oil (42.6 mg, 67% yield).

(*E*)-*N*-(5-phenoxyhex-3-en-1-yl)quinolin-8-amine

Yellow liquid. **^{1}H NMR** (400 MHz, Chloroform-*d*) δ 8.7 (dd, *J* = 4.2, 1.7 Hz, 1H), 8.0 (dd, *J* = 8.3, 1.7 Hz, 1H), 7.4 – 7.3 (m, 2H), 7.3 – 7.1 (m, 2H), 7.0 (dd, *J* = 8.2, 1.2 Hz, 1H), 7.0 – 6.8 (m, 3H), 6.6 (dd, *J* = 7.6, 1.2 Hz, 1H), 6.2 (t, *J* = 5.6 Hz, 1H), 5.9 – 5.5 (m, 2H), 4.8 (p, *J* = 6.2 Hz, 1H), 3.5 – 3.1 (m, 2H), 2.5 (q, *J* = 6.7 Hz, 2H), 1.4 (d, *J* = 6.4 Hz, 3H). **^{13}C NMR** (101 MHz, Chloroform-*d*) δ 158.0, 146.8, 144.7, 138.2, 135.9, 133.6, 129.3, 129.0, 128.7, 127.8, 121.4, 120.6, 116.1, 113.8, 104.6, 74.2, 42.6, 32.0, 21.6. **HRMS**: calculated for : $C_{21}H_{22}N_2O$ [M+Na$^+$] 341.1624, found 341.1623.

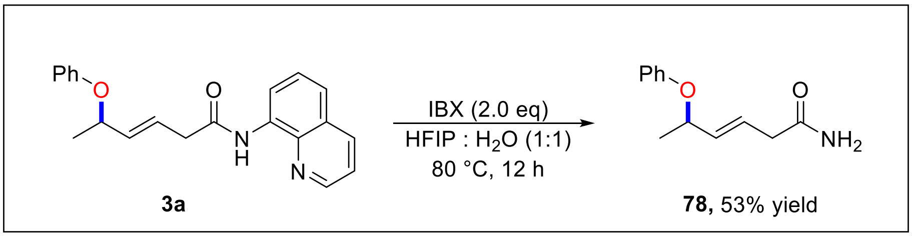

To a solution of IBX (102.2 mg, 0.40 mmol, 2.0 equiv) in HFIP/H_2O (1:1) (2.0 mL) at 80 °C was added compound **3a** (66.4 mg, 0.20 mmol, 1.0 equiv). Then the mixture was stirred for 12 h at the same temperature. Once completion, the mixture was extracted with EA (10 mL × 3). The combined organic phase was dried over $MgSO_4$ and concentrated *in vacuo*. And the residue was purified by column chromatography using eluents (PE/EA = 1:1) to afford the desired product **78** as yellow oil (21.7 mg, 53% yield).

(*E*)-5-phenoxyhex-3-enamide

Yellow liquid. **^{1}H NMR** (400 MHz, Chloroform-*d*) δ 7.3 – 7.2 (m, 2H), 7.0 – 6.8 (m, 3H), 6.0 – 5.7 (m, 2H), 5.5 (d, *J* = 61.9 Hz, 2H), 4.8 (p, *J* = 6.3 Hz, 1H), 3.1 – 2.7 (m, 2H), 1.4 (d, *J* = 6.3 Hz, 3H). **^{13}C NMR** (101 MHz, Chloroform-*d*) δ 173.0, 157.7, 136.3, 129.5, 124.7, 121.1, 116.4, 73.8, 39.5, 21.3. **HRMS**: calculated for : $C_{12}H_{15}NO_2$ [M+Na$^+$] 228.0995, found 228.0990.

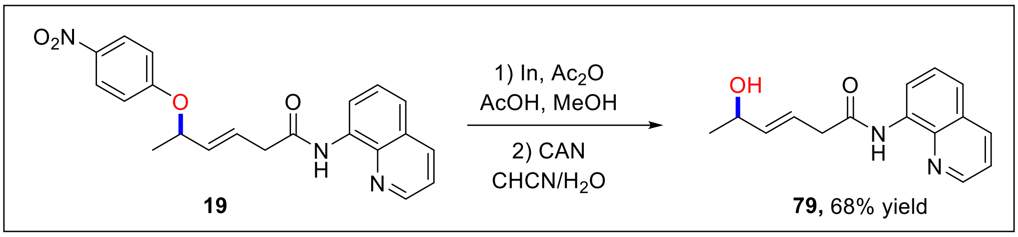

Acetic acid (250 μL, 4.0 mmol) was added to a mixture of indium powder (69 mg, 0.60 mmol), nitroarene, **19** (66.4 mg, 0.2 mmol) and acetic anhydride (284 μL, 3.0 mmol) in MeOH (0.5 mL). The reaction mixture was stirred overnight. After the reaction was complete, the reaction mixture was centrifuged to remove white solid. The recovered solution was concentrated under high vac. And re-dissolved in acetonitrile/water (10/1, 2 mL). CAN (131 mg, 0.24 mmol) was added to the reaction mixture at 0 °C. After 4 h, the reaction mixture was diluted with water and extracted with EA. The combined organic extracts were dried over $MgSO_4$, filtered, and concentrated. This crude material was purified by flash chromatography to afford the desired product **79** as colorless liquid (34.8 mg, 68% yield).

(*E*)-5-hydroxy-*N*-(quinolin-8-yl)hex-3-enamide

Colorless liquid. **^{1}H NMR** (400 MHz, Chloroform-*d*) δ 10.0 (s, 1H), 8.9 – 8.6 (m, 2H), 8.1 (dd, *J* = 8.3, 1.7 Hz, 1H), 7.7 – 7.4 (m, 3H), 6.2 – 5.6 (m, 2H), 4.8 – 4.3 (m, 1H), 3.3 (dd, *J* = 6.2, 1.6 Hz, 2H), 2.4 (s, 1H), 1.4 (d, *J* = 6.4 Hz, 3H). **^{13}C NMR** (101 MHz, Chloroform-*d*) δ 169.2, 148.2, 140.6, 138.4, 136.5, 134.1, 128.0, 127.4, 123.1, 121.7, 121.6, 116.4, 68.6, 41.4, 22.9. **HRMS**: calculated for : $C_{15}H_{16}N_2O_2$ [M+Na$^+$] 279.1104, found 279.1098.

6. Primary kinetic isotope effects

[1]**H NMR**-spectrum (400 MHz, $CDCl_3$) of **83**-*d*$_2$

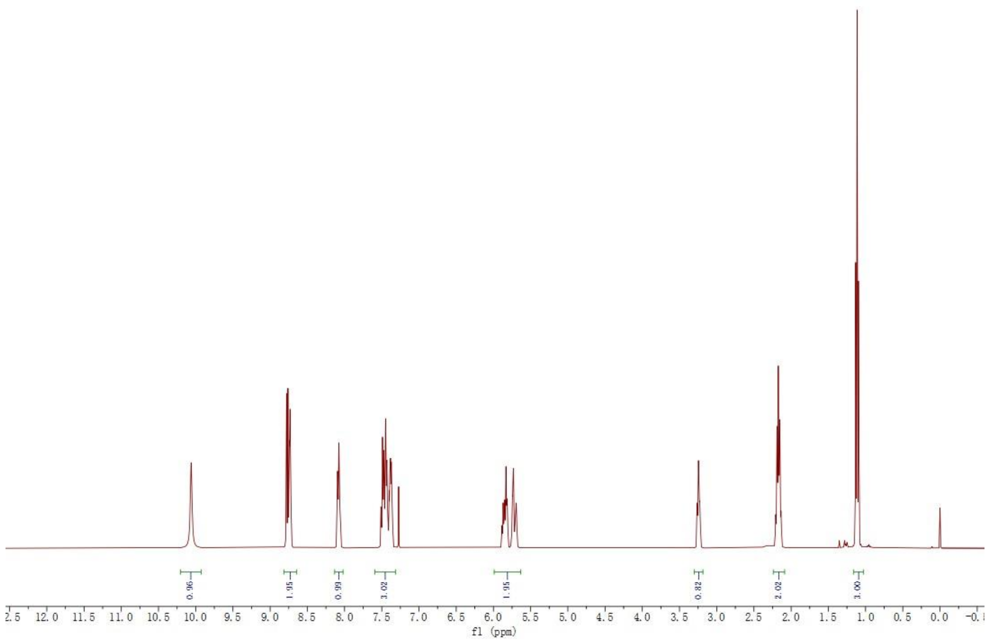

Substrate **83**-*d*$_2$ was treated in standard conditions to give **86**-*d*$_2$

[1]**H NMR**-spectrum (400 MHz, $CDCl_3$) of **86**-*d*$_2$

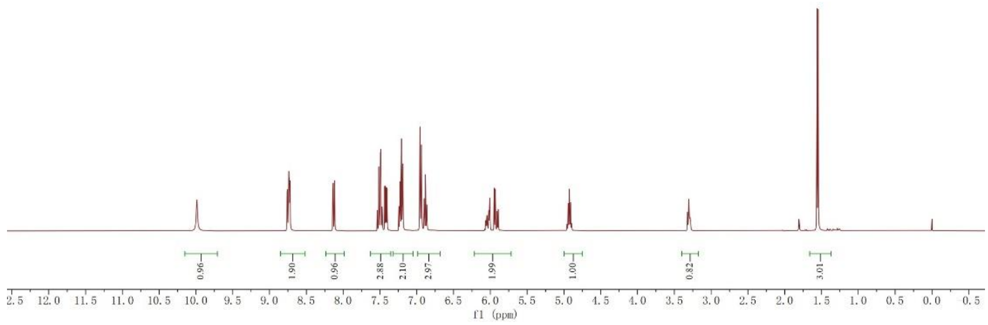

[1]**H NMR**-spectrum (400 MHz, $CDCl_3$) of **84**-d_2

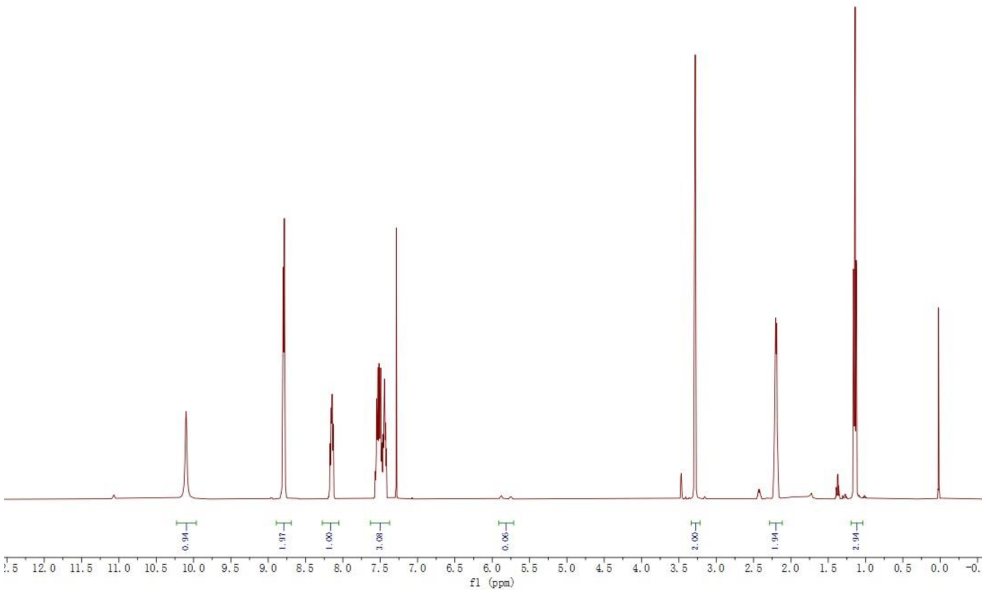

Substrate **84**-d_2 was treated in standard conditions to give **87**-d_2

[1]**H NMR**-spectrum (400 MHz, $CDCl_3$) of **87**-d_2

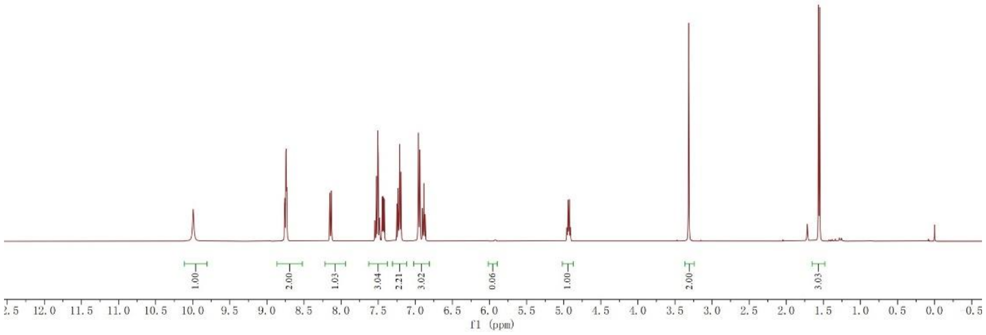

^{1}H NMR-spectrum (400 MHz, $CDCl_3$) of **85**-*d_3*

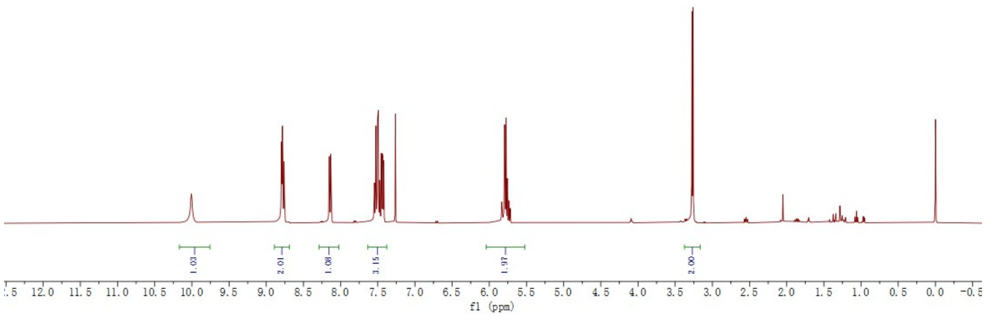

Substrate **85**-*d_3* was treated in standard conditions to give **88**-*d_2*

^{1}H NMR-spectrum (400 MHz, $CDCl_3$) of **88**-*d_2*

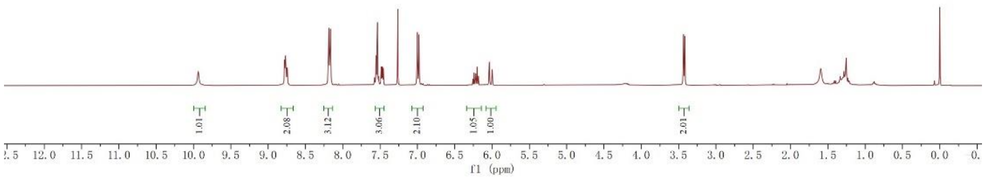

7. General Procedure for KIE Experiments

a) Parellel kinetic isotope effect

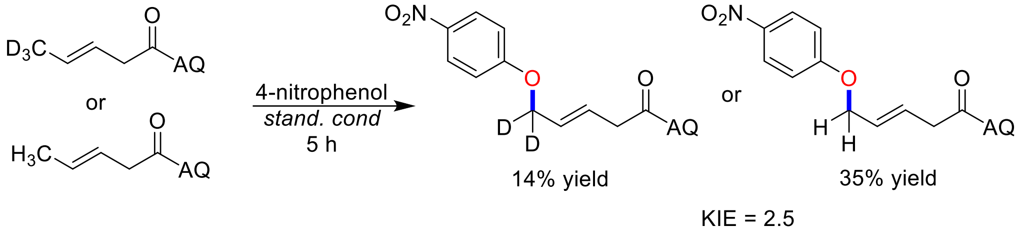

One mixture of trans-3-pentenamide (0.4 mmol, 1.0 equiv), Cu_2O (0.04 mmol, 0.1 equiv), **L15** (0.08 mmol, 0.2 equiv), DEAD (0.80 mmol, 2.0 equiv) and 4-nitrophenol (1.20 mmol, 3.0 equiv) in toluene (2 mL) in a 10 mL glass vial (sealed with PTFE cap). The other mixture of amide **85-*d*₃** (0.4 mmol, 96 mg), Cu_2O (0.04 mmol, 0.1 equiv), **L15** (0.08 mmol, 0.2 equiv), DEAD (0.80 mmol, 2.0 equiv) and 4-nitrophenol (1.20 mmol, 3.0 equiv) in toluene (2 mL) in a 10 mL glass vial (sealed with PTFE cap). After heated at 80 °C for 5 h and cooled to room temperature, the reaction mixtures were diluted with DCM, filtered through a pad of Celite and concentrated *in vacuo*. The resulting residues were purified by flash column chromatography to give the etherification product **19** as a colorless oil. (50.8 mg, 35% yield) and the etherification product **88-d_2** as a colorless oil. (20.4 mg, 14% yield) respectively. By the yield of the product, KIE=35%/14%=2.5.

b) Intermoleculr kinetic isotope effect

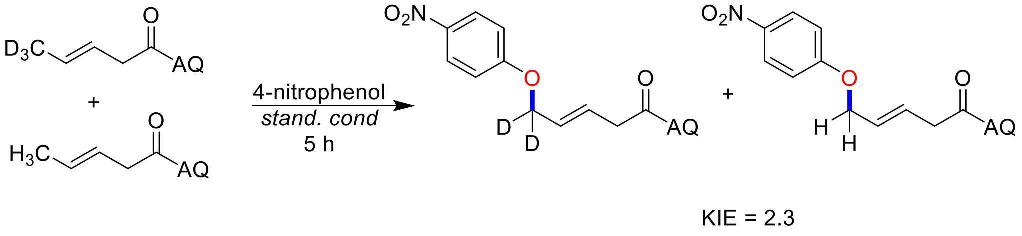

A mixture of trans-3-pentenamide (0.2 mmol, 47.2 mg), amide **85-d_3** (0.2 mmol, 47.2 mg), Cu_2O (0.04 mmol, 0.1 equiv), **L15** (0.08 mmol, 0.2 equiv), DEAD (0.80 mmol, 2.0 equiv) and 4-nitrophenol (1.20 mmol, 3.0 equiv) in Toluene (2 mL) in a 10 mL glass vial (sealed with PTFE cap). After heated at 80 °C for 5 h and cooled to room temperature, the reaction mixtures were diluted with DCM, filtered through a pad of celite and concentrated *in vacuo*. The resulting residue was purified by flash column chromatography to give the desired product **19/88-d_2** as a colorless oil. By the analysis of the **^{1}H NMR** of **19/88-d_2**, KIE=1.4/0.6=2.333.

^{1}**H NMR**-spectrum (400 MHz, $CDCl_3$) of **19/88-d_2**

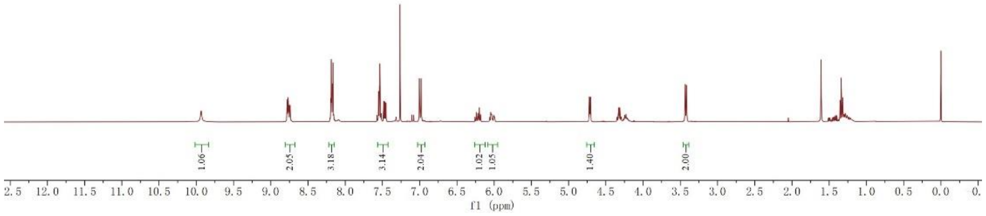

8. Crystal structure information

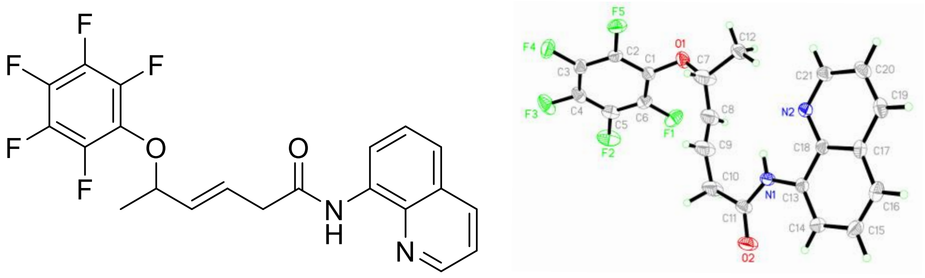

X-ray structure of **28** (CCDC 2322949)

A colorless block shaped crystal of **28** ($C_{21}H_{15}F_5N_2O_2$) was used for the X-ray crystallographic analysis. The X-ray intensity data were measured at 173(2) K, on a Bruker D8 VENTURE CMOS photon 100 diffractometer with helios mx multilayer monochrmator Cu Kα radiation (λ = 1.54178 Å).

Crystal Data and Structure Refinement for **28**.

Empirical formula	$C_{21}H_{15}F_5N_2O_2$
Formula weight	422.35
Temperature	173(2) K
Wavelength	1.54178 A
Crystal system, space group	Monoclinic, P-2(1)/n
Unit cell dimensions	a = 7.8624(2) A alpha = 90 deg.<br>b = 12.8110(3) A beta = 91.0250(10) deg.<br>c = 18.6195(4) A gamma = 90 deg.

Volume	1875.15(8) A^3
Z, Calculated density	4, 1.496 Mg/m^3
Absorption coefficient	1.139 mm^-1
F(000)	864
Crystal size	0.180 x 0.160 x 0.140 mm
Theta range for data collection	4.189 to 68.449 deg.
Limiting indices	-9<=h<=9, -15<=k<=15, -22<=l<=21
Reflections collected / unique	16386 / 3412 [R(int) = 0.0413]
Completeness to theta =66.386	99.1 %
Refinement method	Full-matrix least-squares on F^2
Data / restraints / parameters	3412 / 0 / 275
Goodness-of-fit on F^2	1.060
Final R indices [I>2sigma(I)]	R_1 = 0.0624, wR_2 = 0.1559
R indices (all data)	R_1 = 0.0683, wR_2 = 0.1605
Extinction coefficient	n/a
Largest diff. peak and hole	0.796 and -0.703 e.A^-3

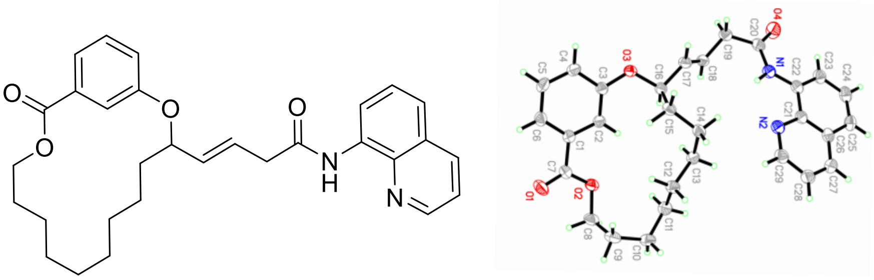

X-ray structure of **70** (CCDC 2322950)

A colorless block shaped crystal of **70** ($C_{29}H_{32}N_2O_4$) was used for the X-ray crystallographic analysis. The X-ray intensity data were measured at 173(2) K, on a Bruker D8 VENTURE CMOS photon 100 diffractometer with helios mx multilayer monochrmator Cu Kα radiation (λ = 1.54178 Å).

Crystal Data and Structure Refinement for **70**.

Empirical formula	$C_{29}H_{32}N_2O_4$
Formula weight	472.56
Temperature	173(2) K
Wavelength	1.54178 A
Crystal system, space group	Monoclinic, P-2(1)/n

Unit cell dimensions	a = 22.6584(10) A alpha = 90 deg.<br>b = 24.0726(7) A beta = 98.3130(10) deg.<br>c = 18.0754(5) A gamma = 90 deg.
Volume	2436.84(11) A^3
Z, Calculated density	4, 1.288 Mg/m^3
Absorption coefficient	0.688 mm^-1
F(000)	1008
Crystal size	0.180 x 0.160 x 0.140 mm
Theta range for data collection	3.078 to 68.178 deg.
Limiting indices	-6<=h<=6, -28<=k<=28, -21<=l<=20
Reflections collected / unique	26462 / 4444 [R(int) = 0.0707]
Completeness to theta =66.386	99.9 %
Refinement method	Full-matrix least-squares on F^2
Data / restraints / parameters	4444 / 0 / 319
Goodness-of-fit on F^2	1.024
Final R indices [I>2sigma(I)]	R_1 = 0.0419, wR_2 = 0.1074
R indices (all data)	R_1 = 0.0532, wR_2 = 0.1168
Extinction coefficient	n/a
Largest diff. peak and hole	0.180 and -0.206 e.A^-3

9. Computational details

The UM06 density functional[4] was employed for the computational study. The Lanl2DZ basis set together with the Lanl2DZ pseudopotential[5] was used to describe Cu atoms and 6-31g(d,p) basis set[6] was utilized for other atoms. Vibrational frequency analyses were carried out at the same level to confirm all the optimized structures as transition states (only one imaginary frequency), and provided the thermal relative Gibbs free energy correction. The solvent effect of toluene in the reaction was evaluated using the SMD solvation model developed by Truhlar and Cramer[7]. This model was used for single point energy calculations based on the gas phase optimized geometries with M06 at a larger basis set (SDD[8] for Cu atoms and 6-311+g(d,p) for other atoms). The wavefunction stability of all the computed results were checked. For the purpose of discussion, the solvation Gibbs free energy was used and it was obtained from the addition of solvation single point energy and gas-phase thermal correction to Gibbs free energy. All calculations were carried out by Gaussian 16 program package[9].

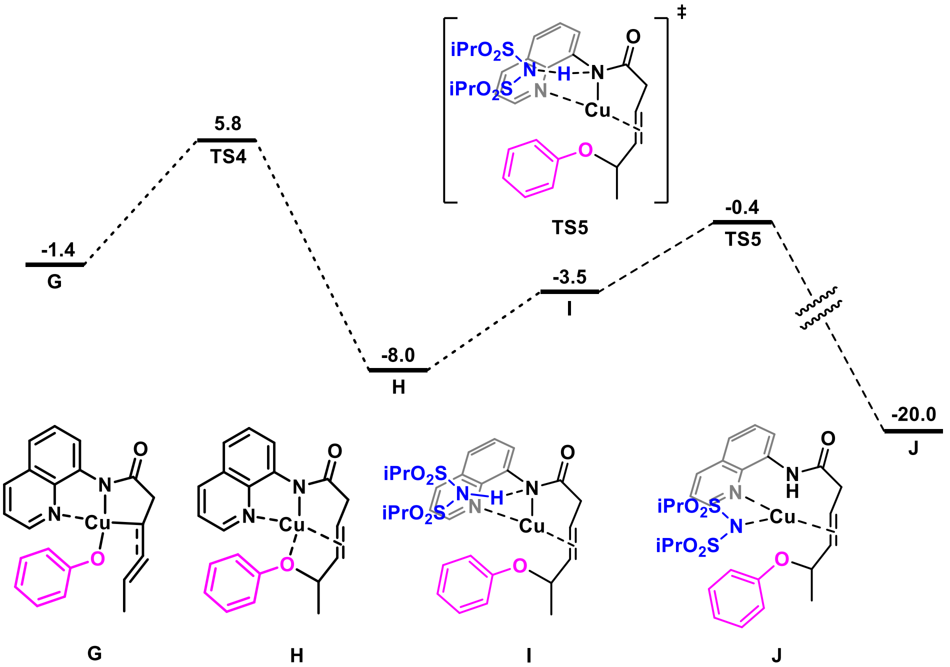

Figure S1. The subsequent transformation starts from **H**

10. NMR Spectra

^{1}H NMR-spectrum (400 MHz, $CDCl_3$) of **3a**

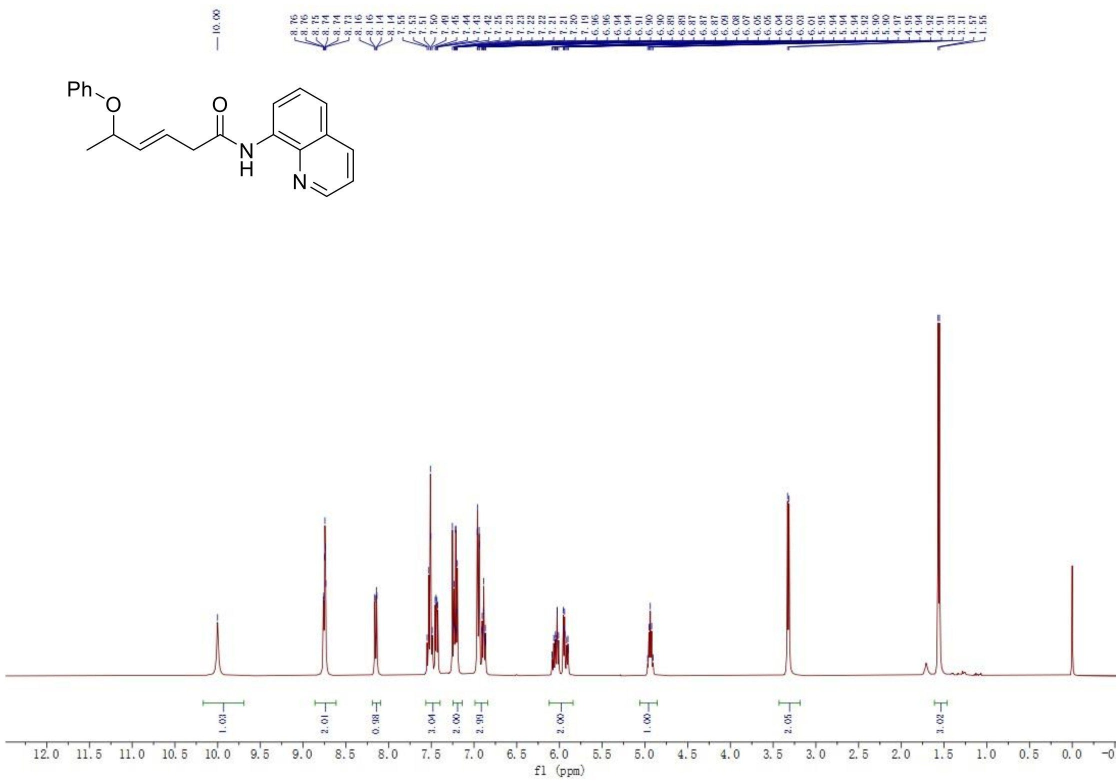

^{13}C NMR-spectrum (101 MHz, $CDCl_3$) of **3a**

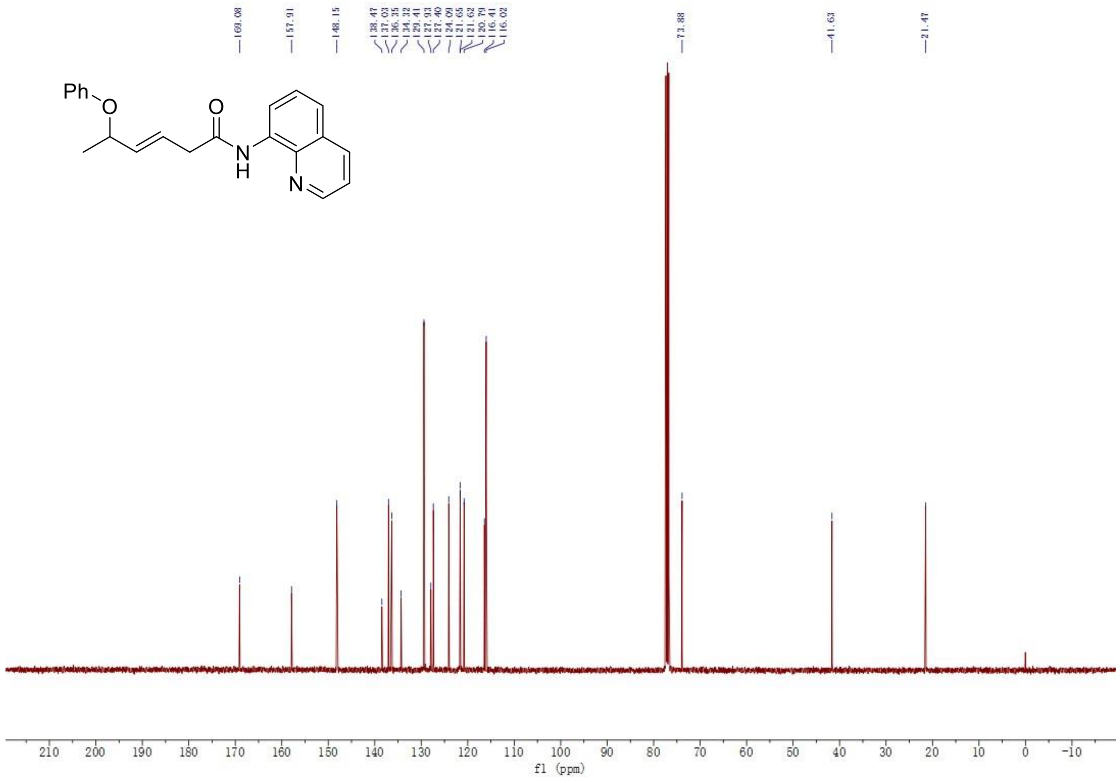

^{1}H NMR-spectrum (400 MHz, $CDCl_3$) of **4**

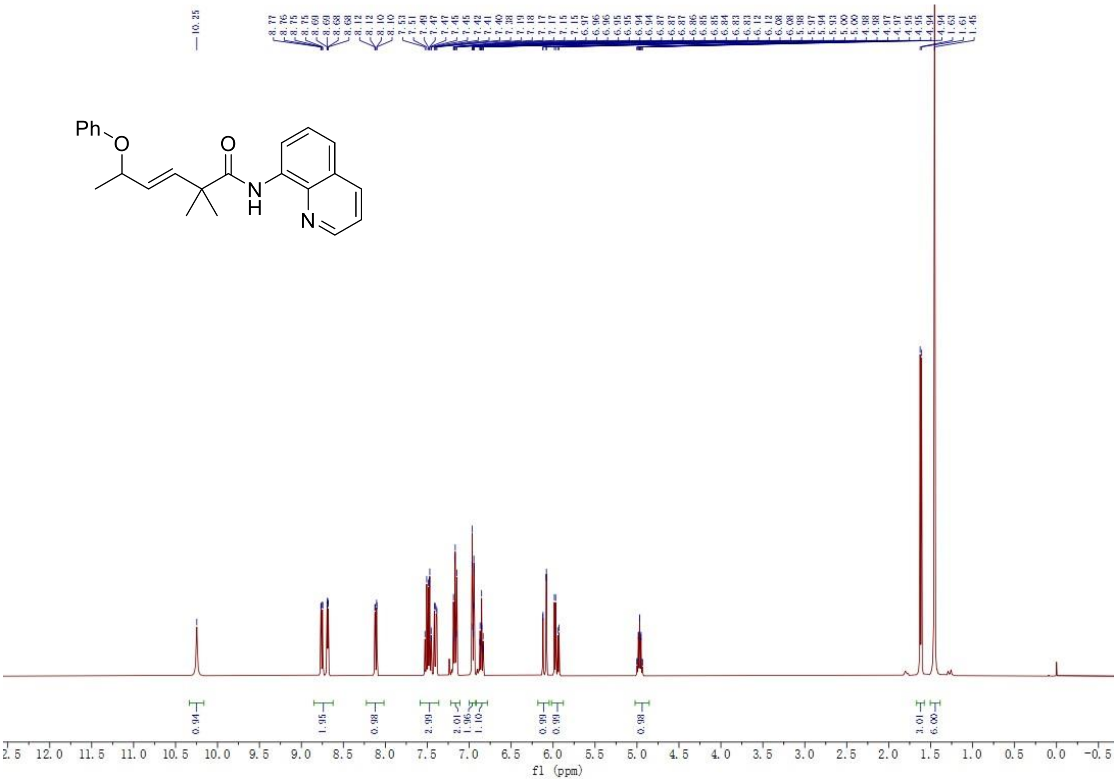

^{13}C NMR-spectrum (101 MHz, $CDCl_3$) of **4**

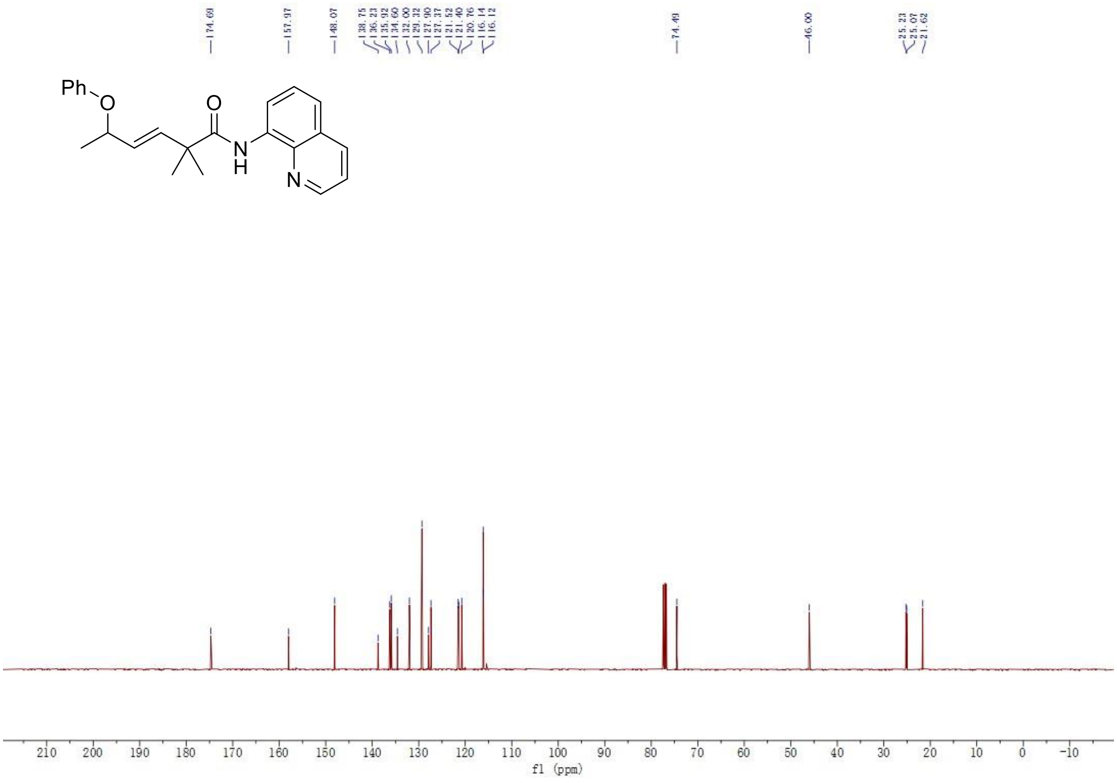

^{1}H NMR-spectrum (400 MHz, $CDCl_3$) of **5**

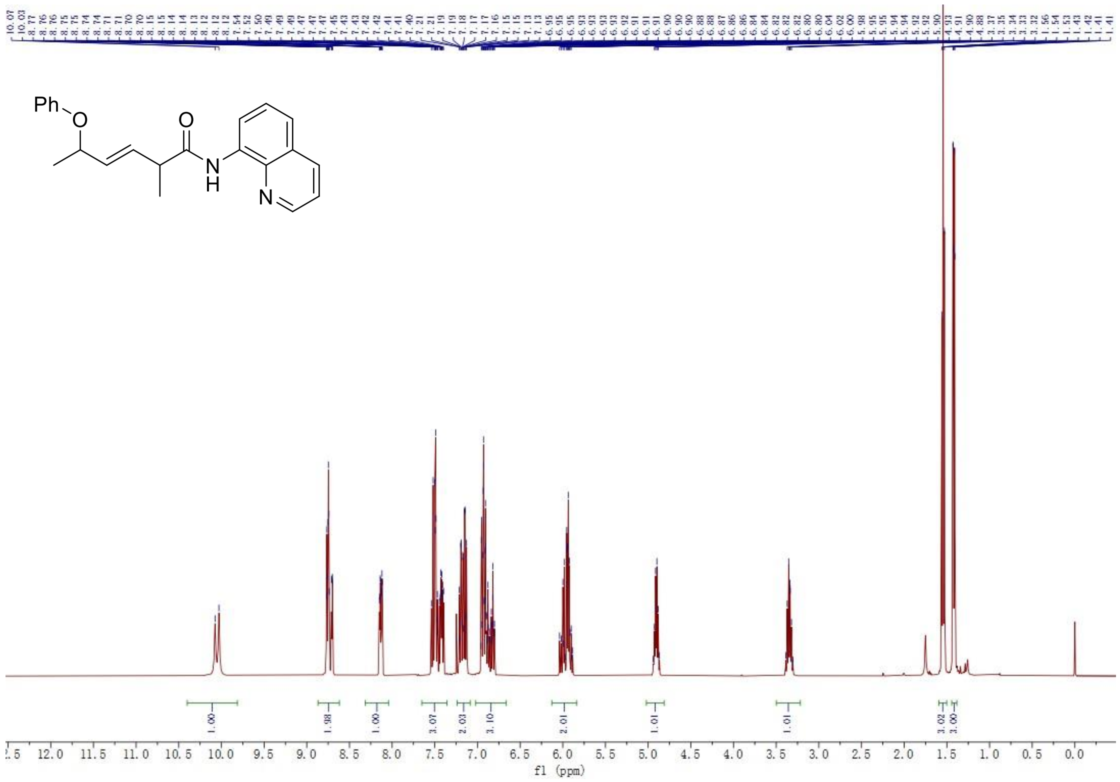

^{13}C NMR-spectrum (101 MHz, $CDCl_3$) of **5**

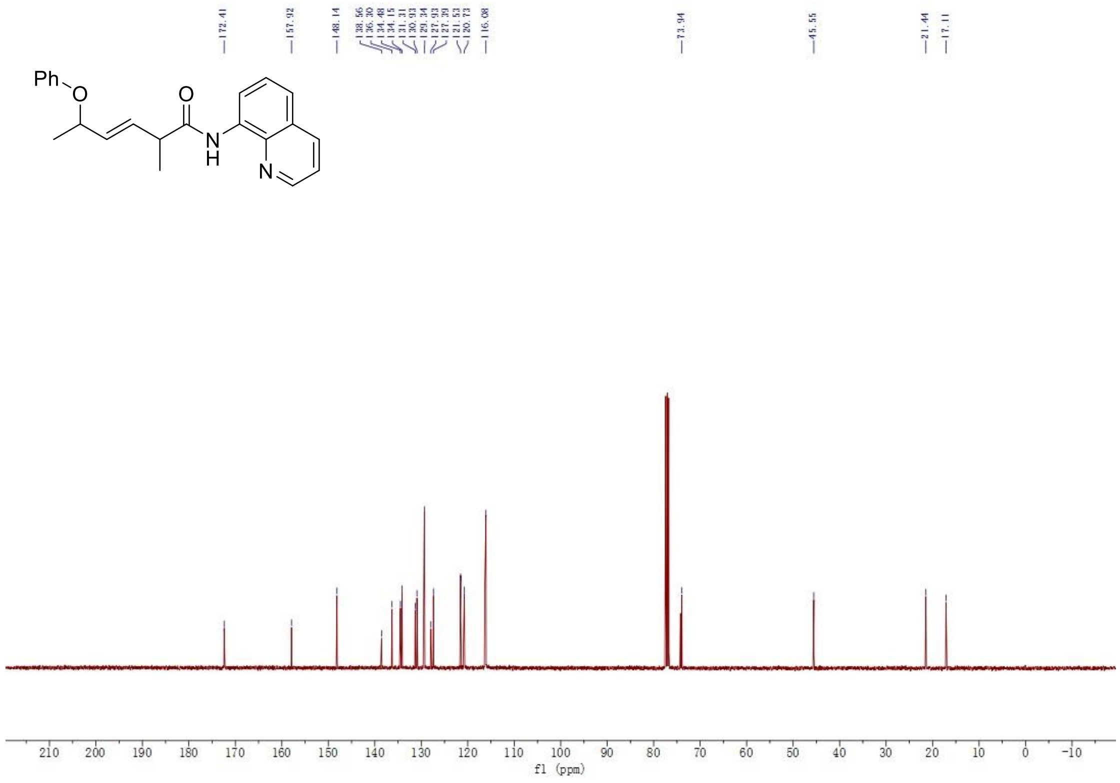

^{1}**H NMR**-spectrum (400 MHz, CDCl$_3$) of **6**

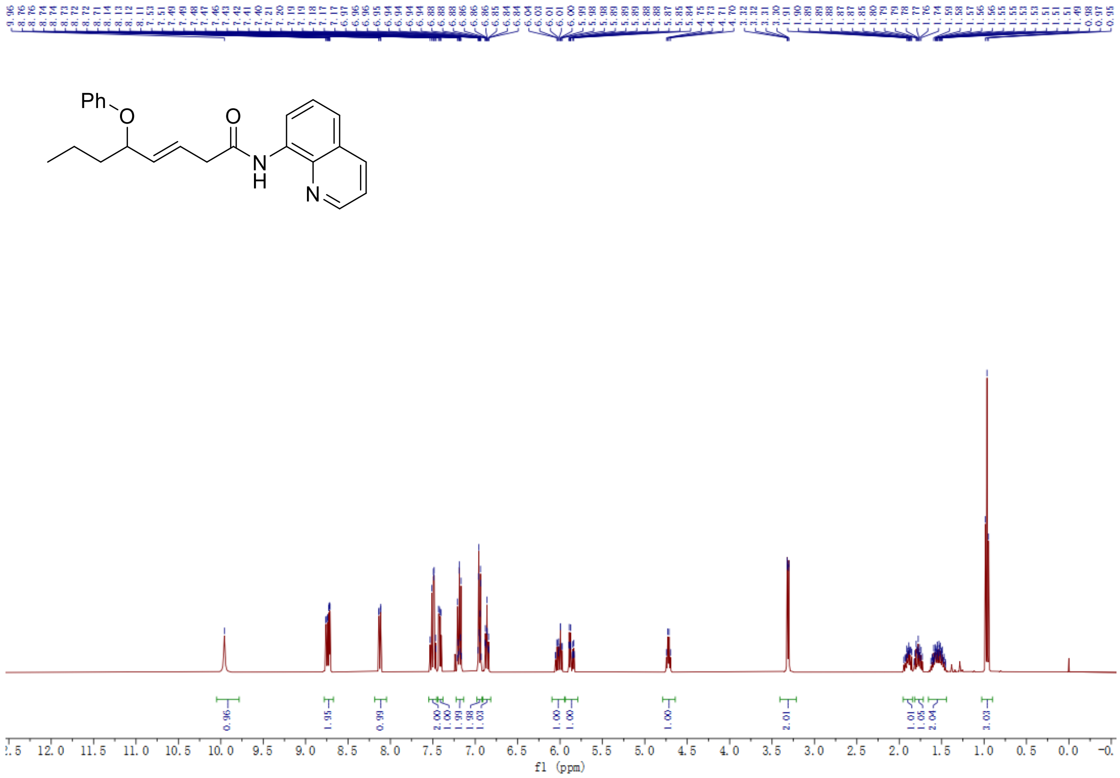

^{13}**C NMR**-spectrum (101 MHz, CDCl$_3$) of **6**

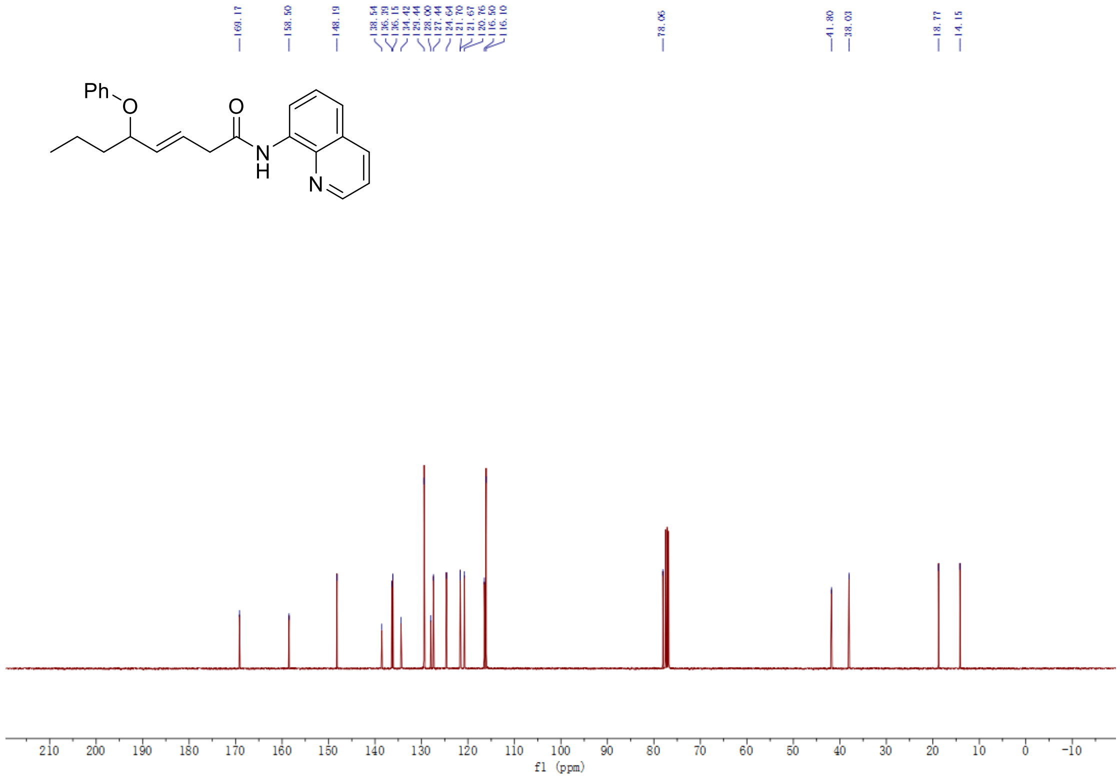

^{1}**H NMR**-spectrum (400 MHz, CDCl3) of **7**

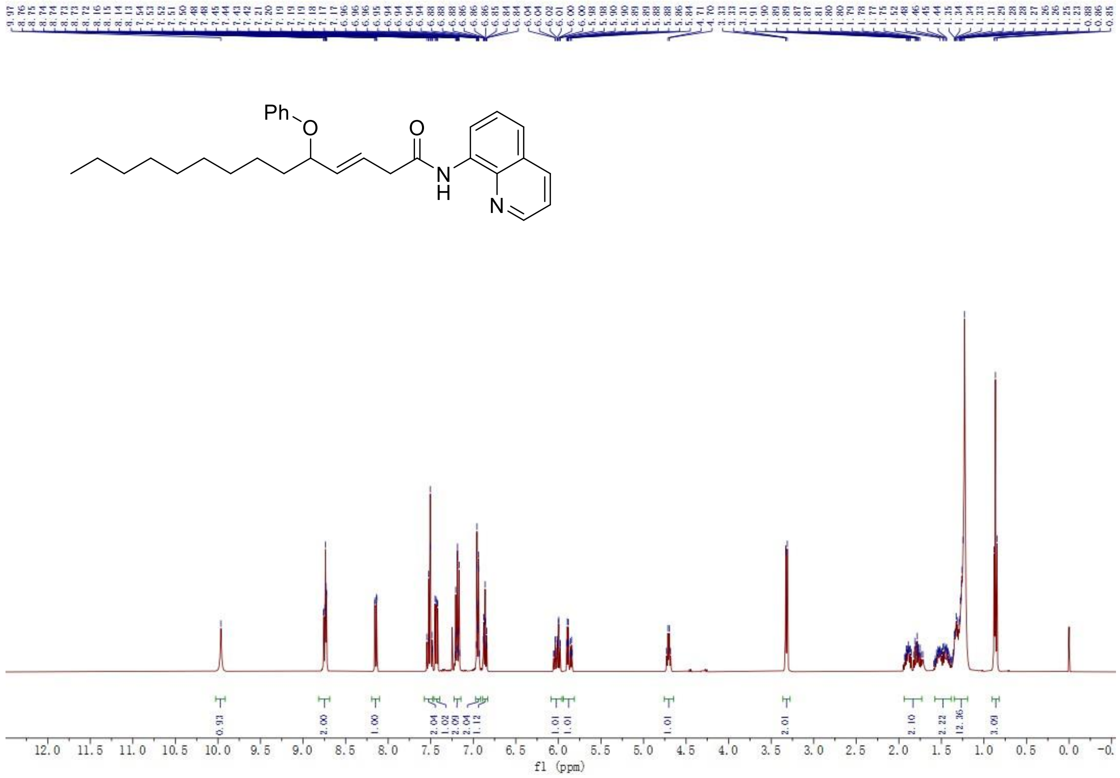

^{13}**C NMR**-spectrum (101 MHz, CDCl3) of **7**

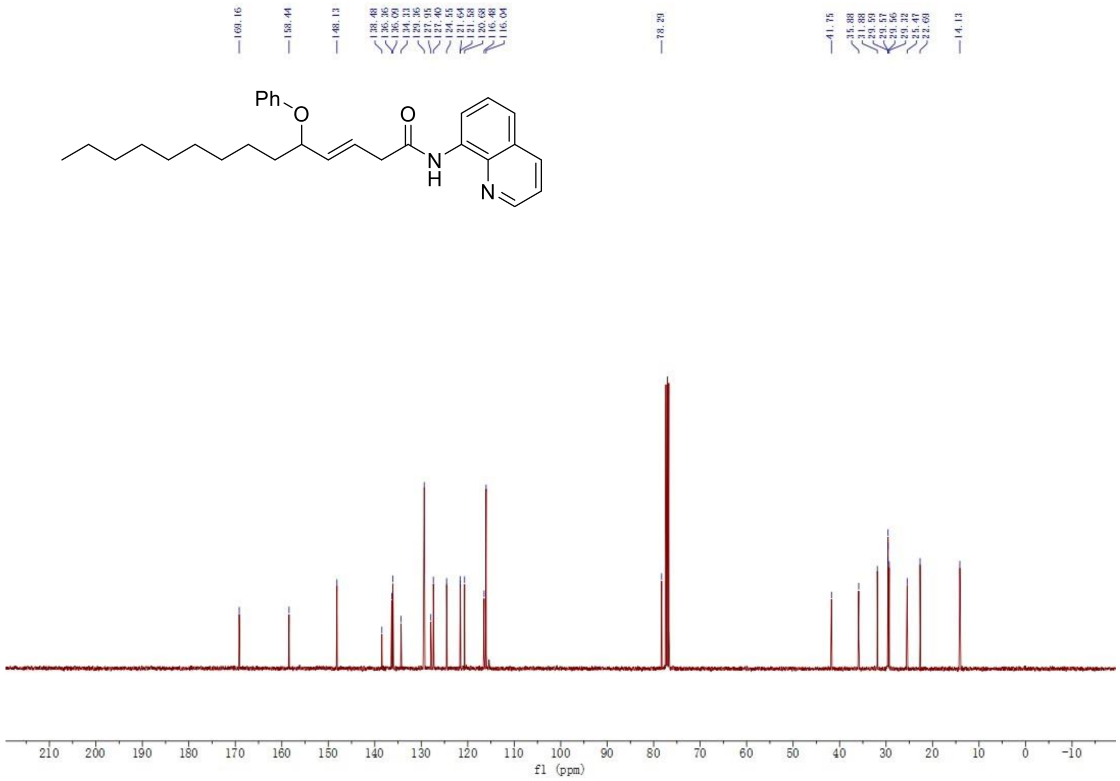

[1]**H NMR**-spectrum (400 MHz, $CDCl_3$) of **8**

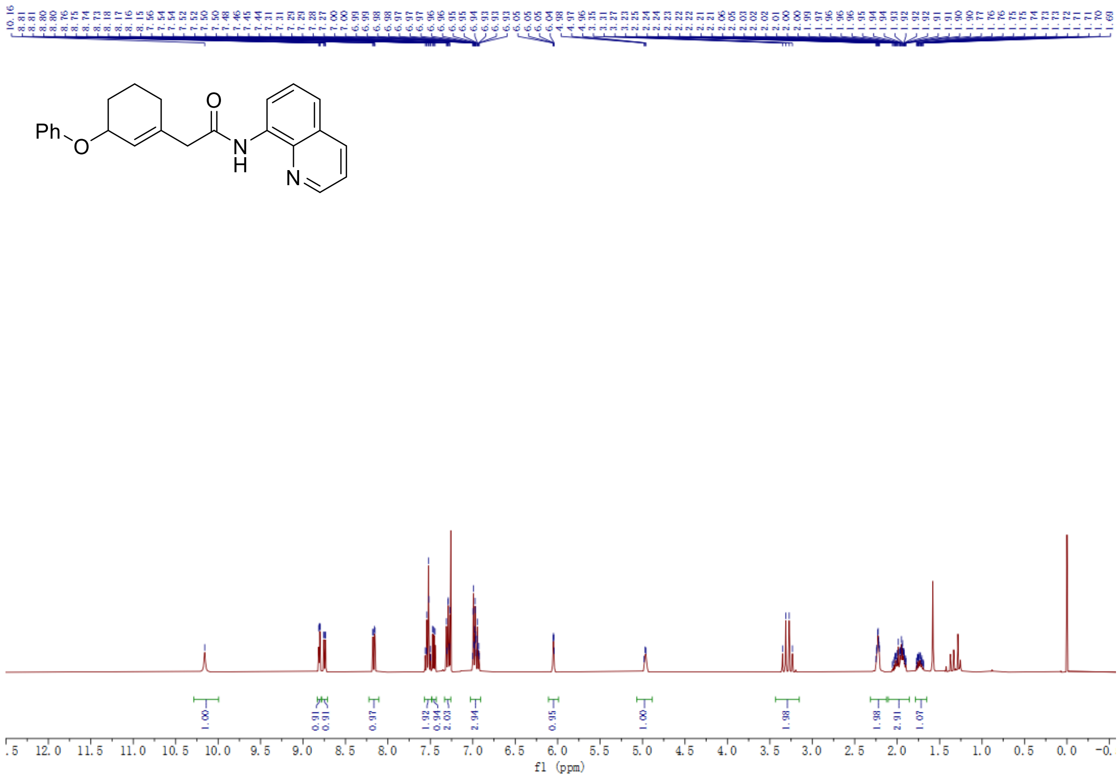

[13]**C NMR**-spectrum (101 MHz, $CDCl_3$) of **8**

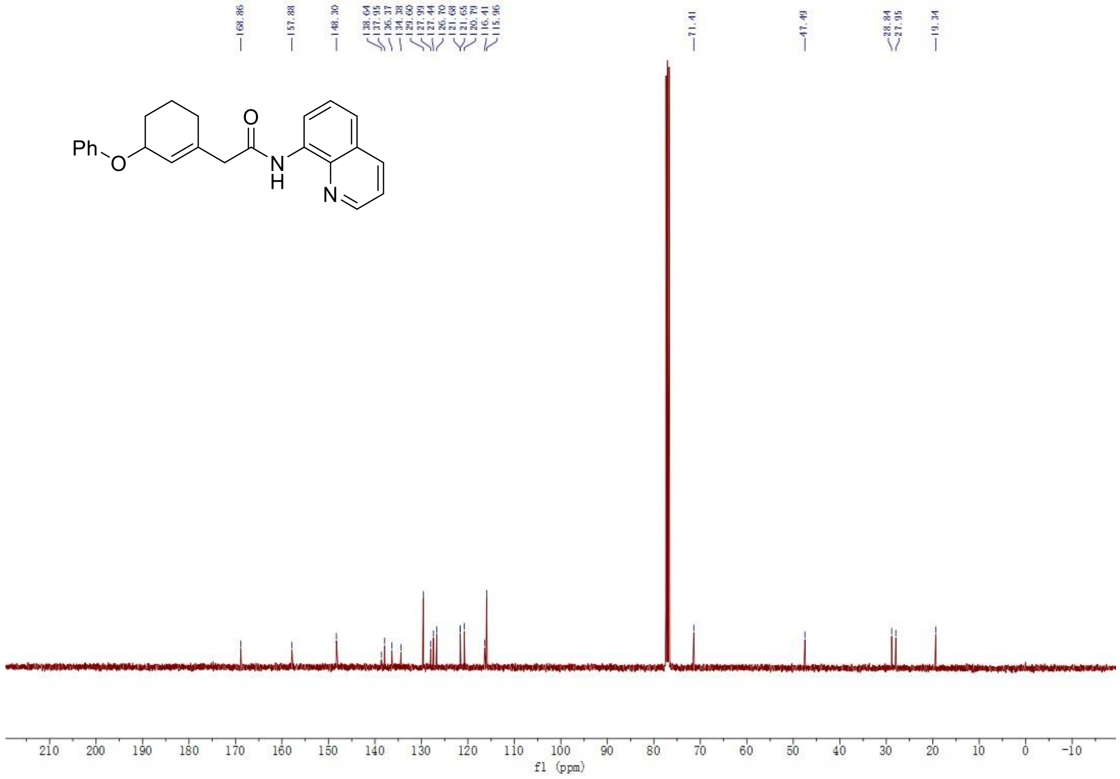

^{1}**H NMR**-spectrum (400 MHz, $CDCl_3$) of **9**

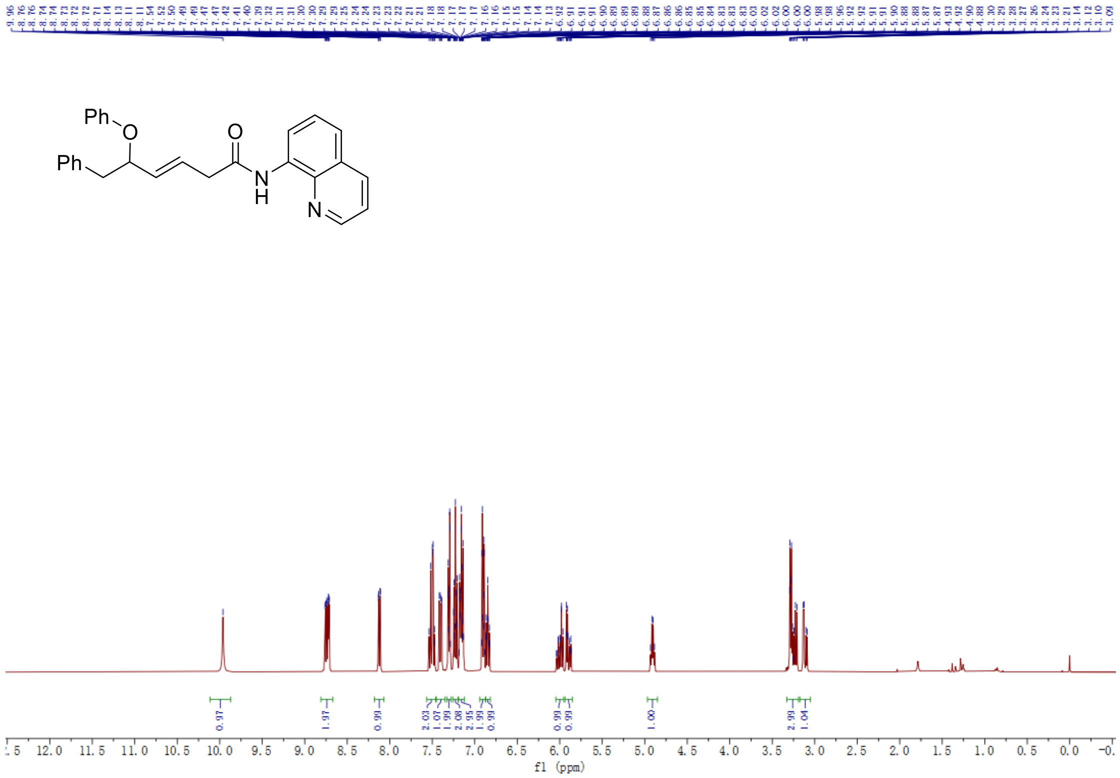

^{13}**C NMR**-spectrum (101 MHz, $CDCl_3$) of **9**

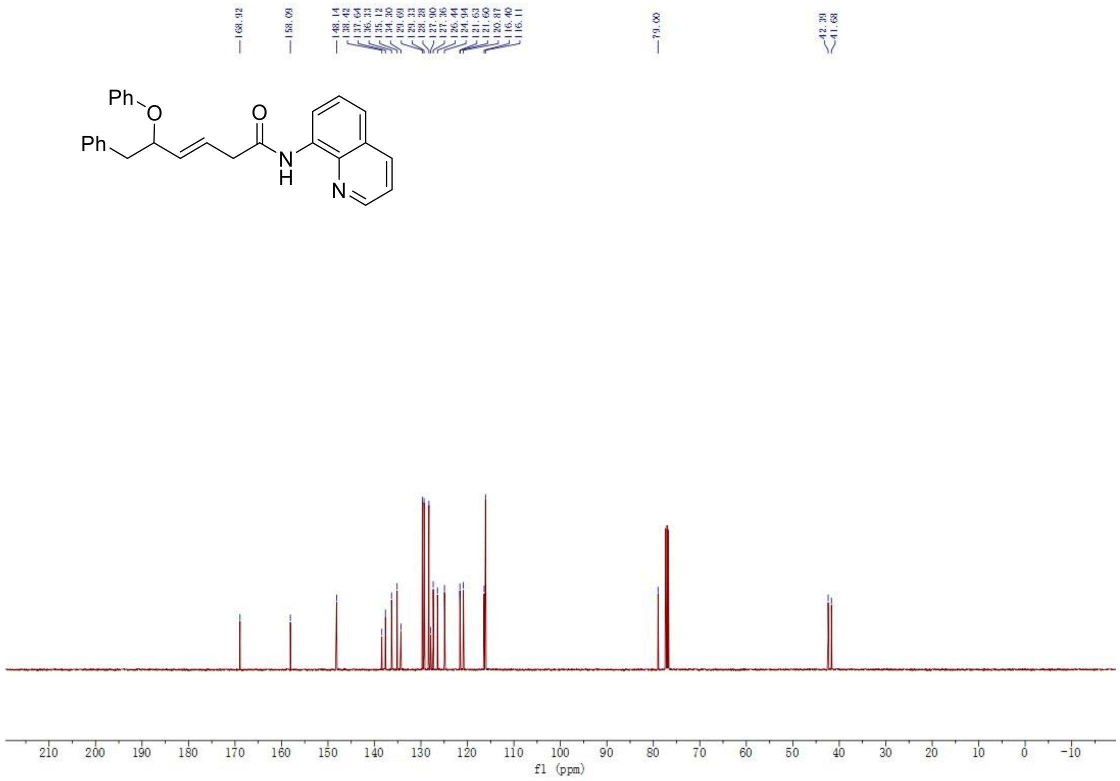

^{1}**H NMR**-spectrum (400 MHz, $CDCl_3$) of **10**

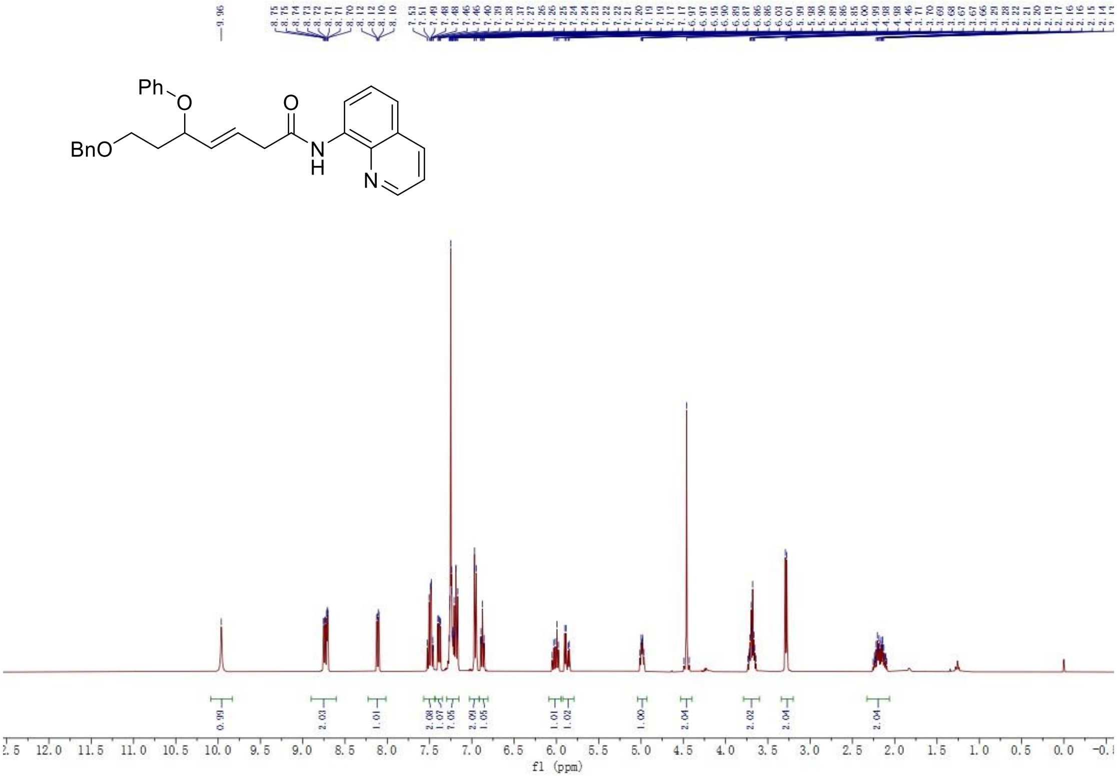

^{13}**C NMR**-spectrum (101 MHz, $CDCl_3$) of **10**

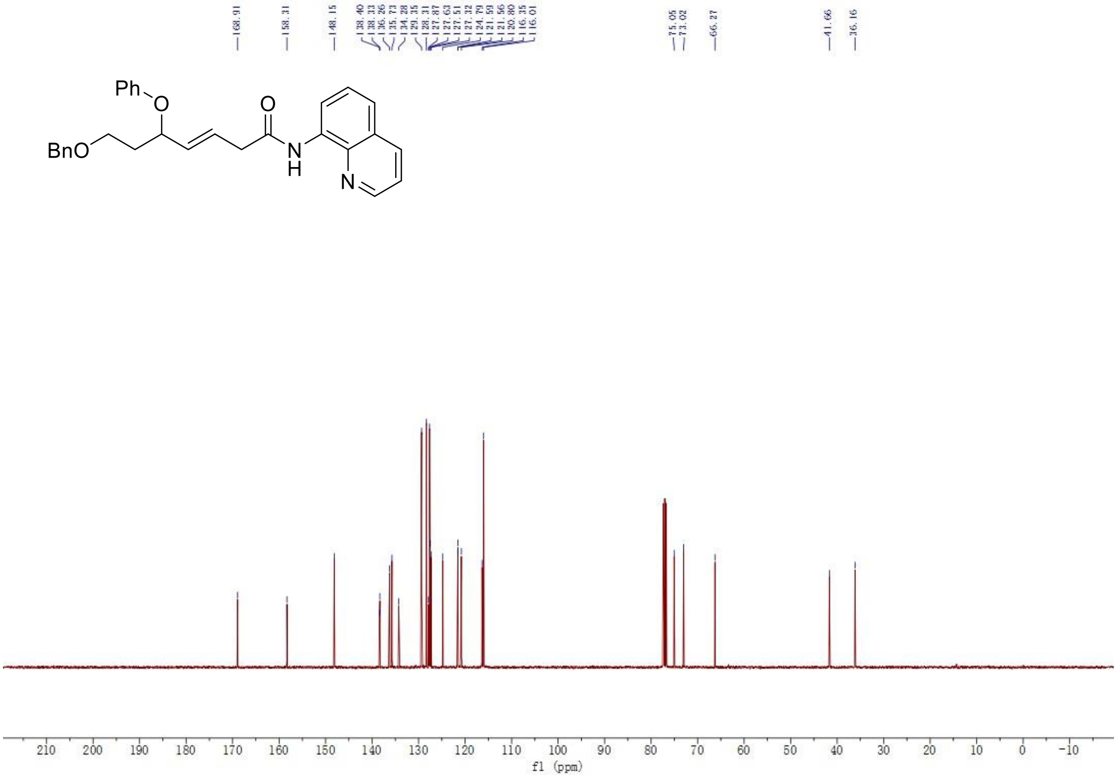

^{1}H NMR-spectrum (400 MHz, $CDCl_3$) of **11**

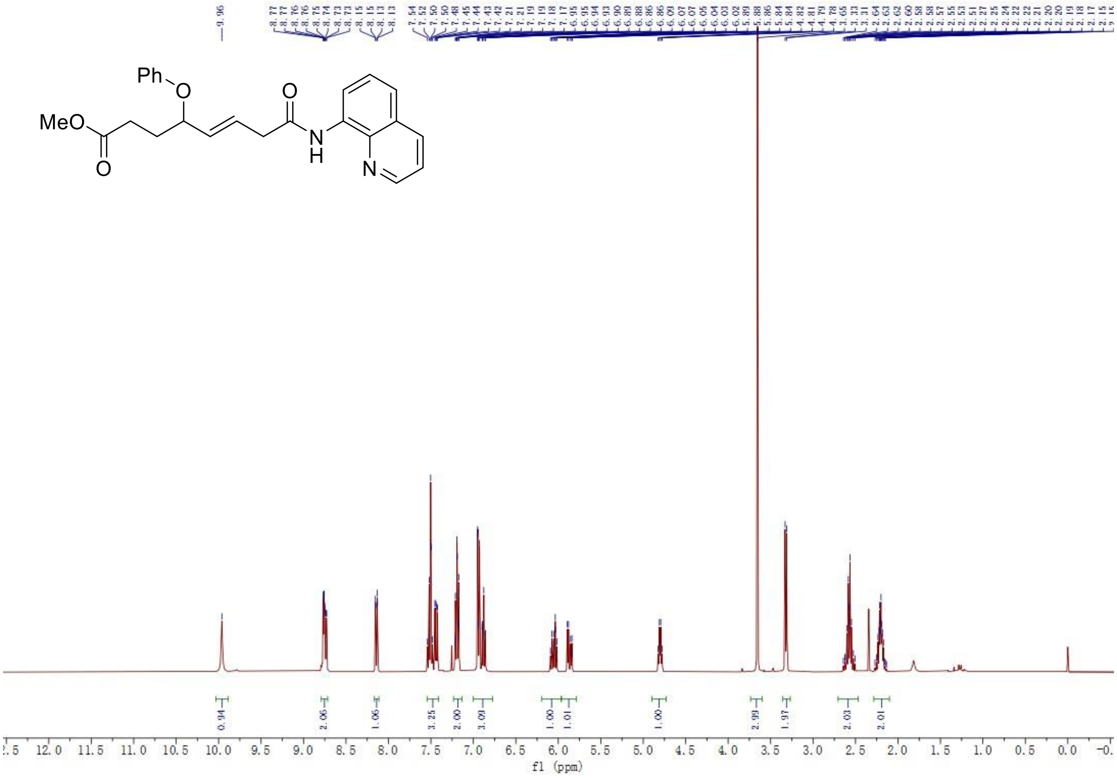

^{13}C NMR-spectrum (101 MHz, $CDCl_3$) of **11**

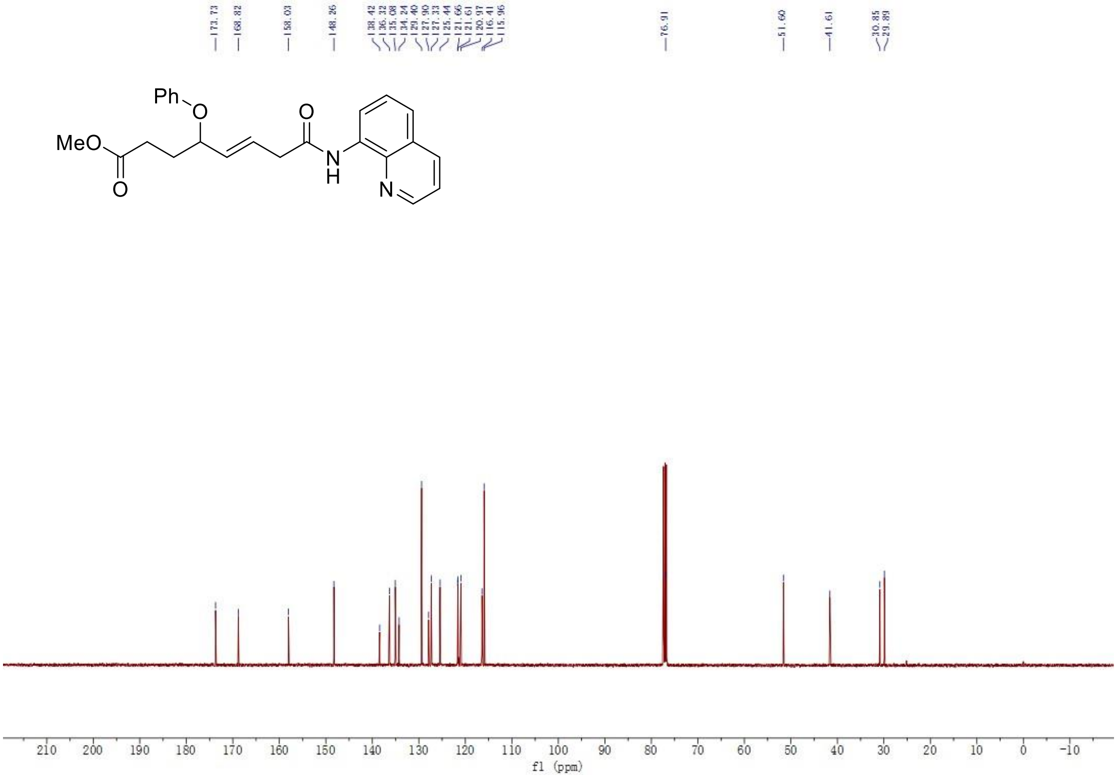

^{1}**H NMR**-spectrum (400 MHz, $CDCl_3$) of **12**

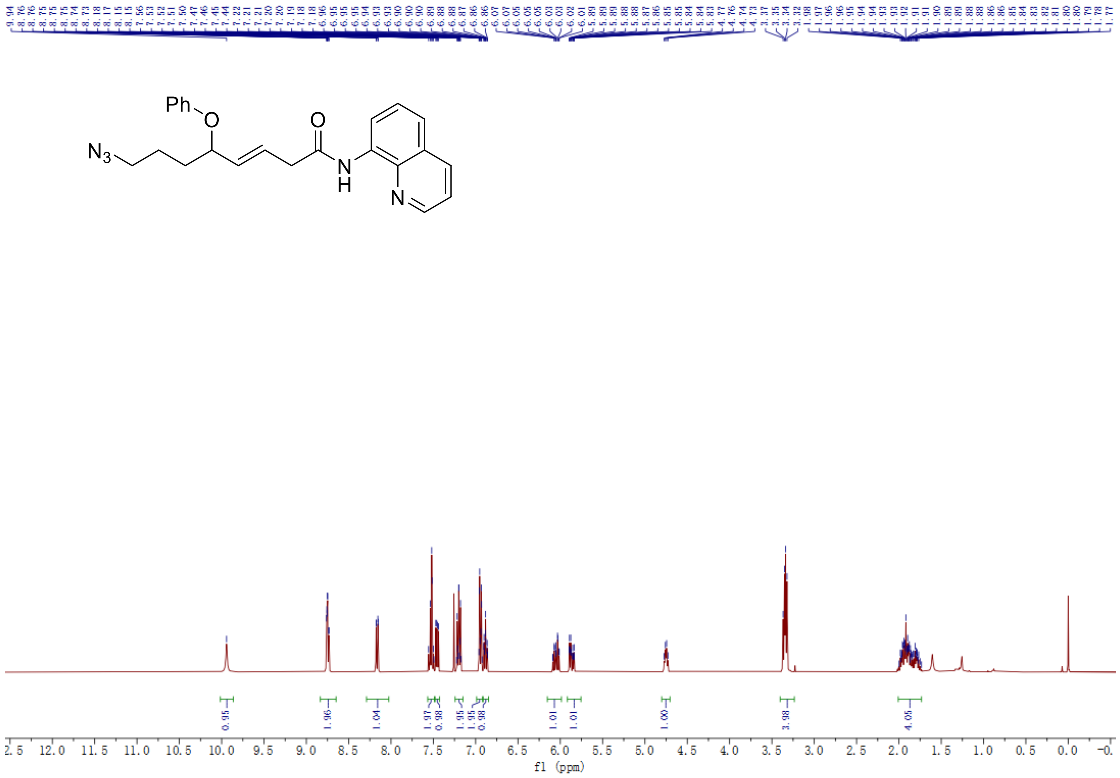

^{13}**C NMR**-spectrum (101 MHz, $CDCl_3$) of **12**

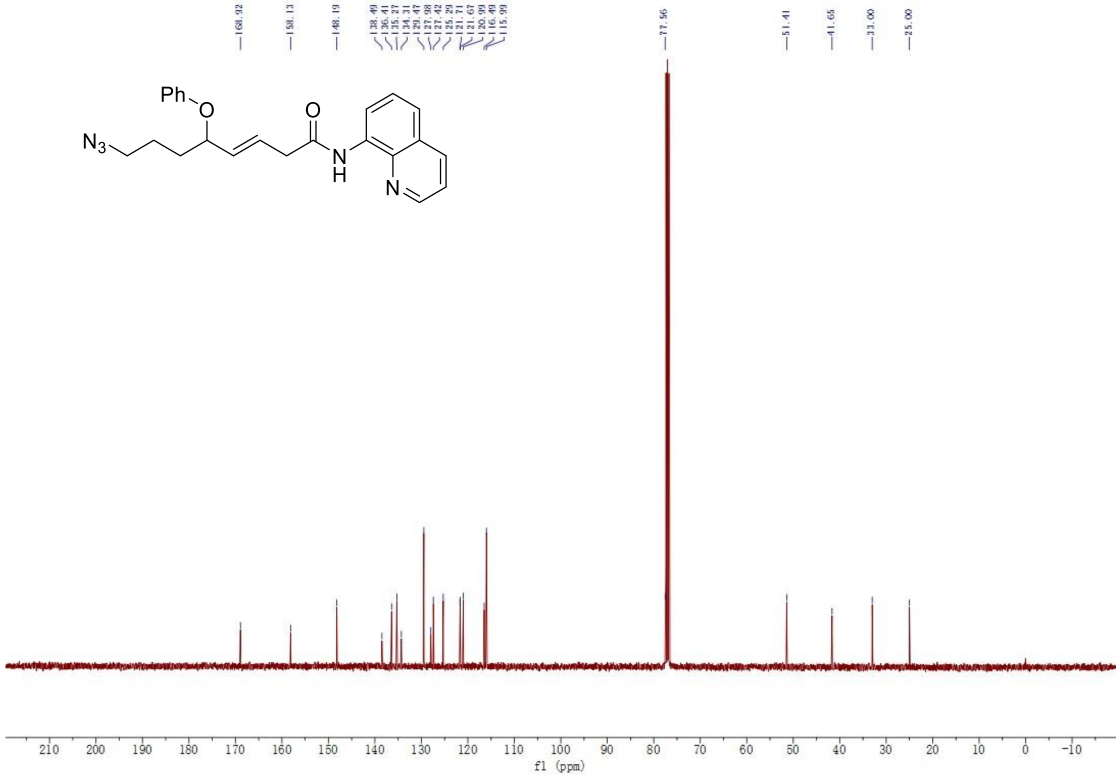

^{1}H NMR-spectrum (400 MHz, $CDCl_3$) of **13**

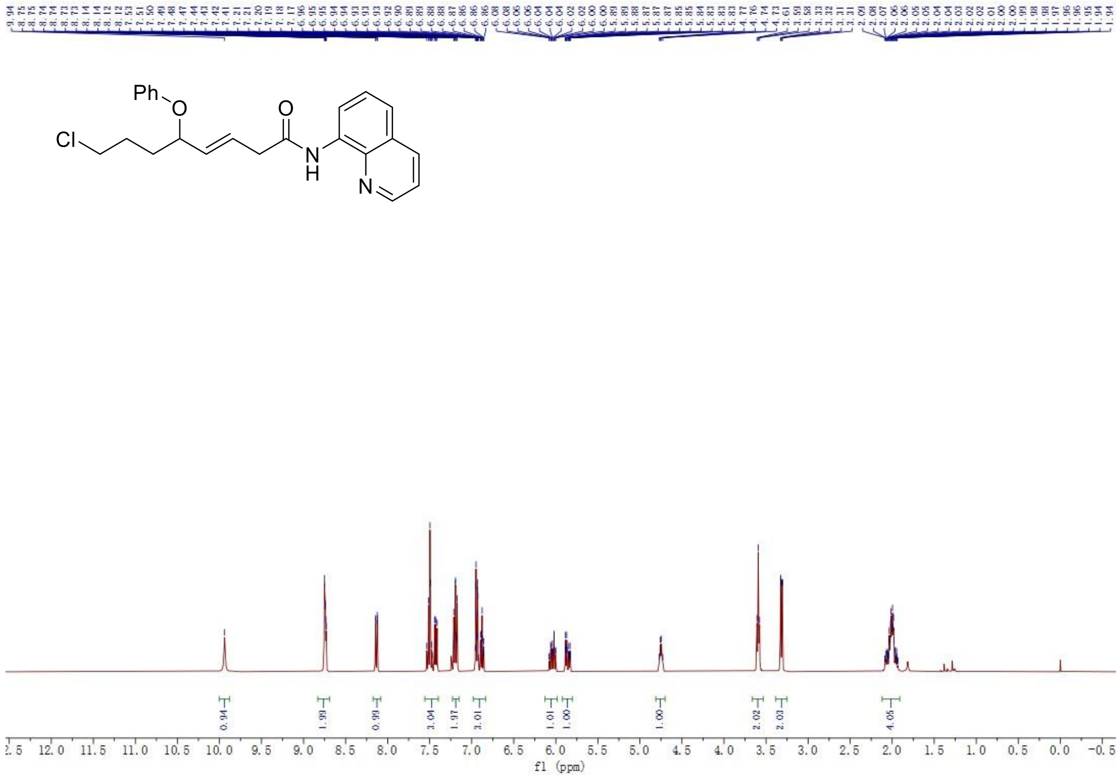

^{13}C NMR-spectrum (101 MHz, $CDCl_3$) of **13**

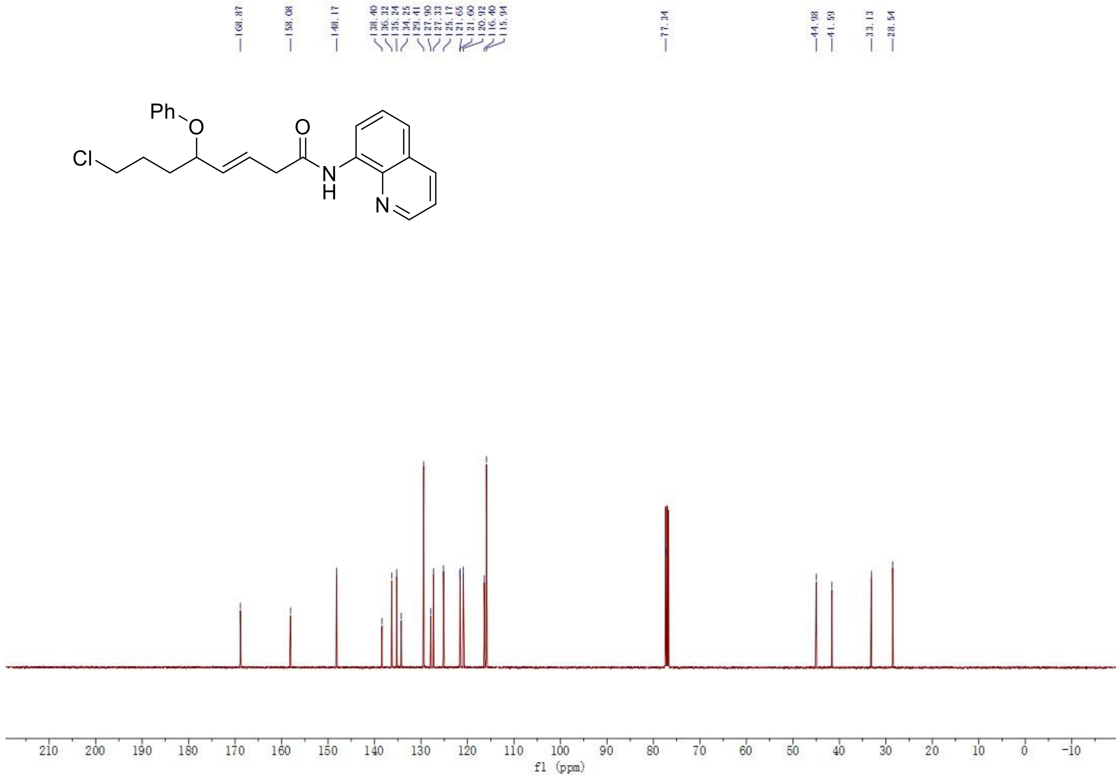

^{1}H NMR-spectrum (400 MHz, $CDCl_3$) of **14**

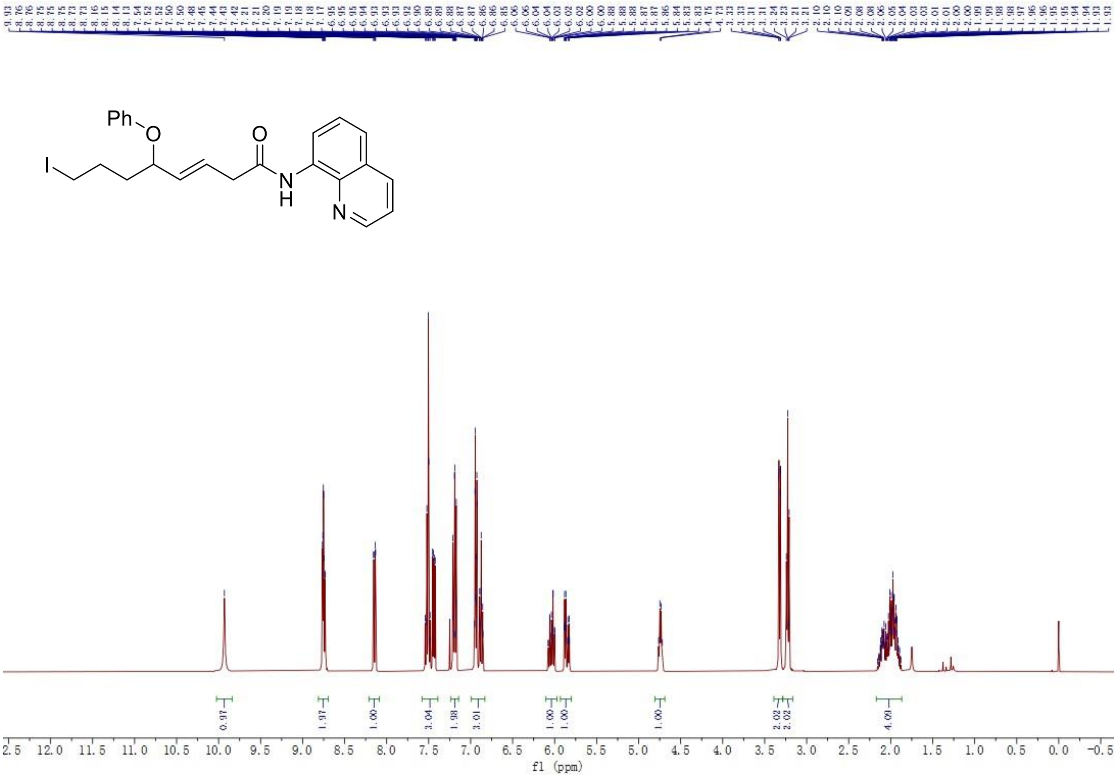

^{13}C NMR-spectrum (101 MHz, $CDCl_3$) of **14**

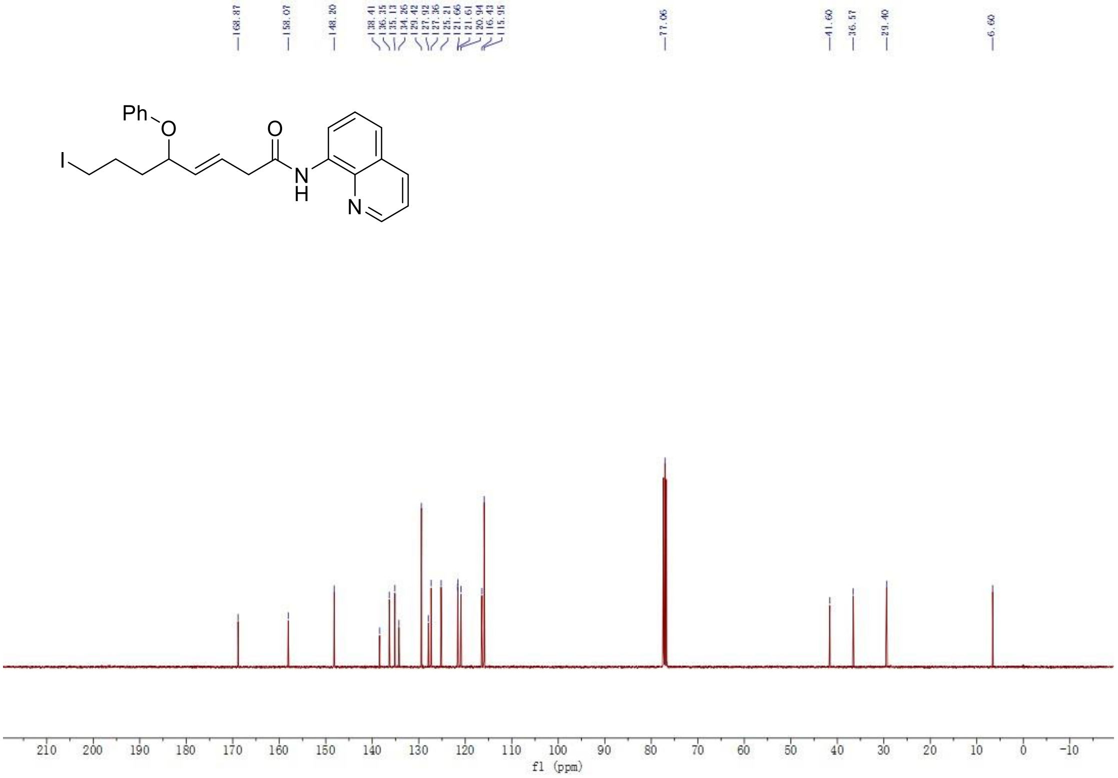

[1]**H NMR**-spectrum (400 MHz, CDCl3) of **15**

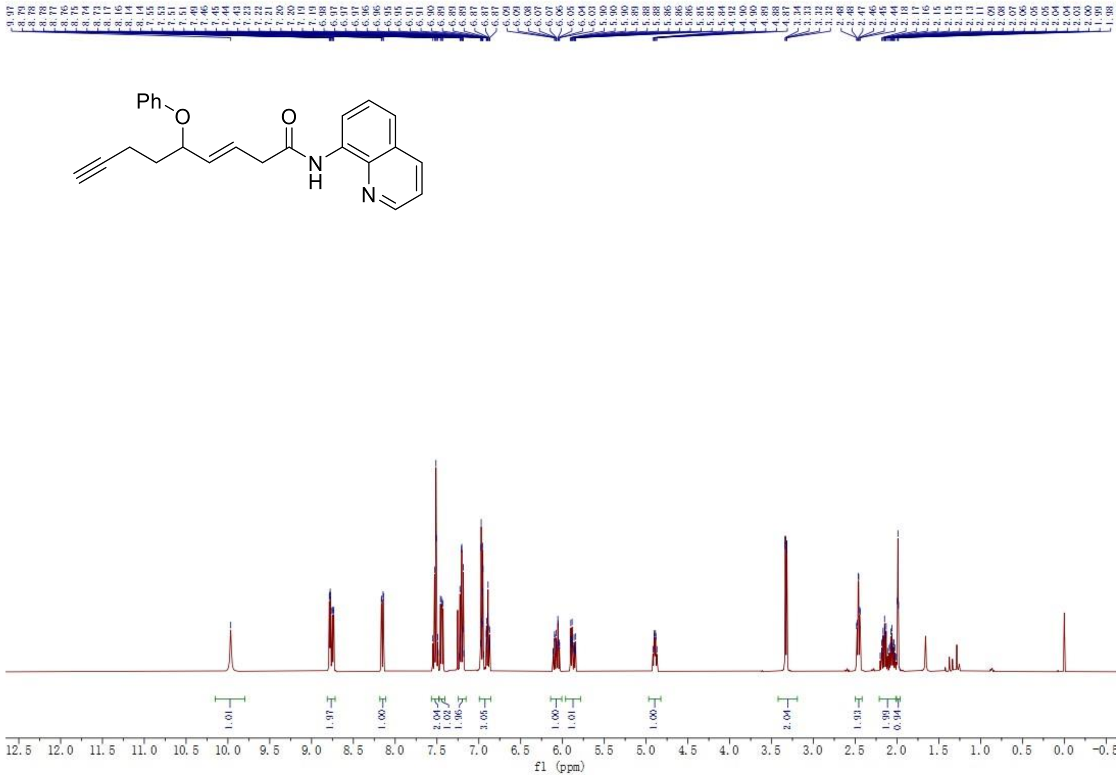

[13]**C NMR**-spectrum (101 MHz, CDCl3) of **15**

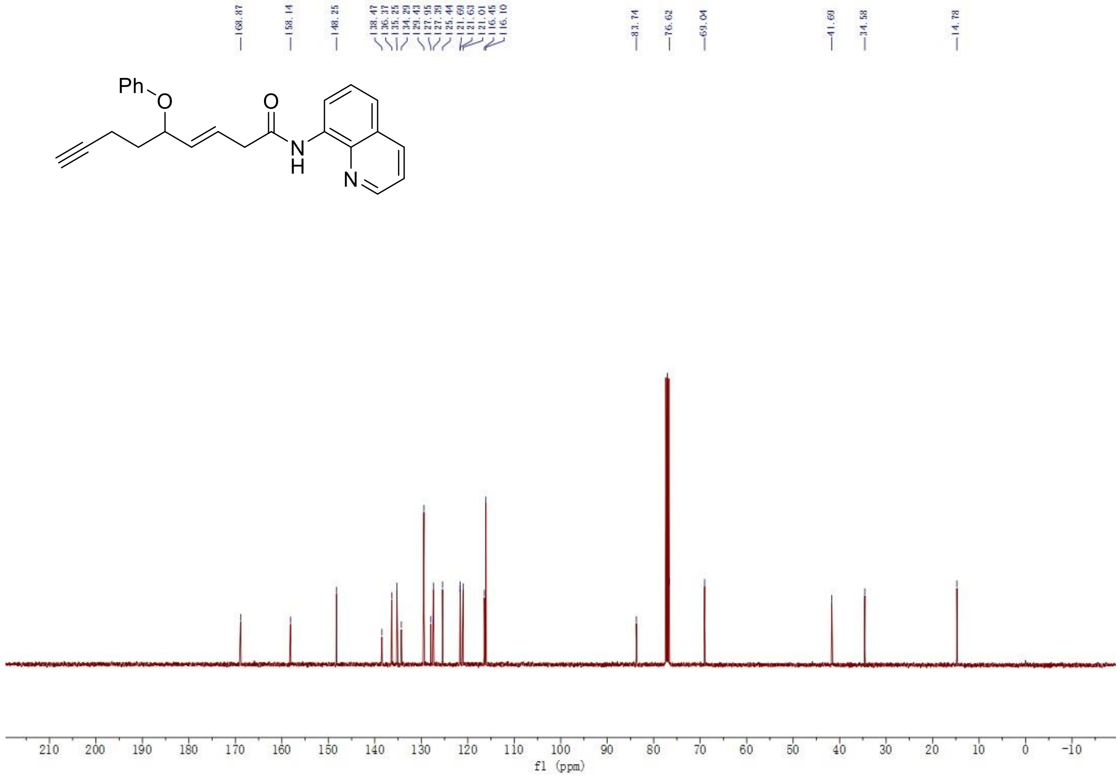

[1]**H NMR**-spectrum (400 MHz, $CDCl_3$) of **16**

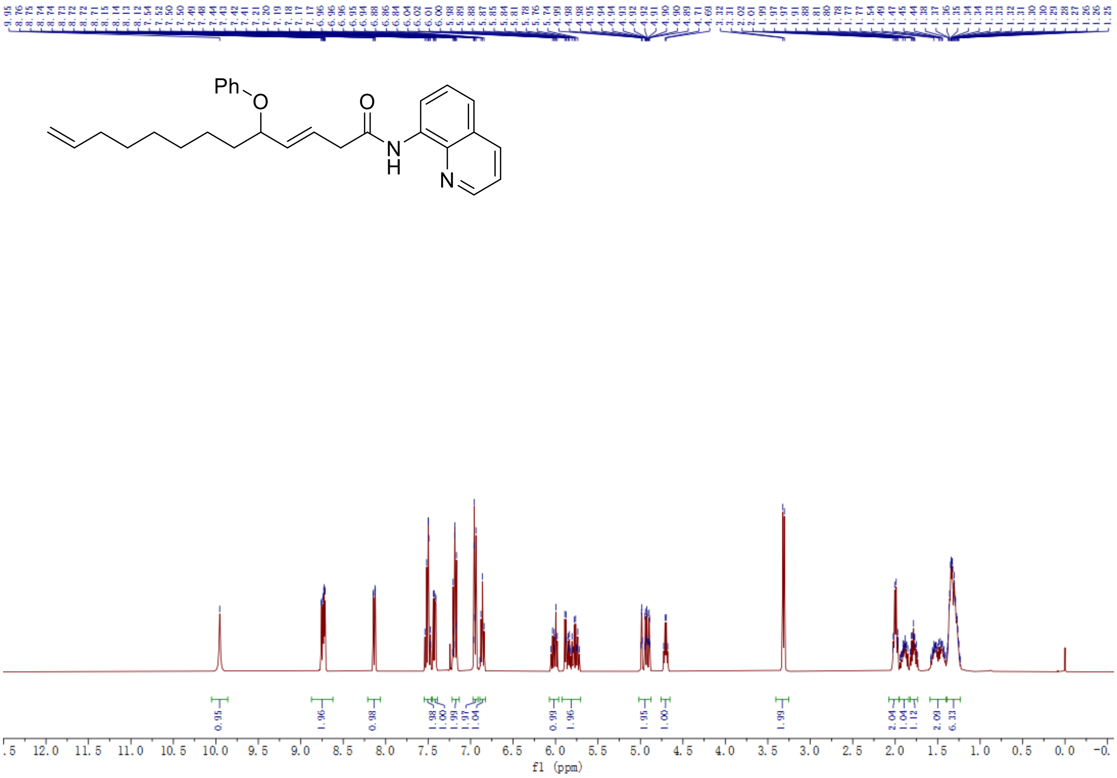

[13]**C NMR**-spectrum (101 MHz, $CDCl_3$) of **16**

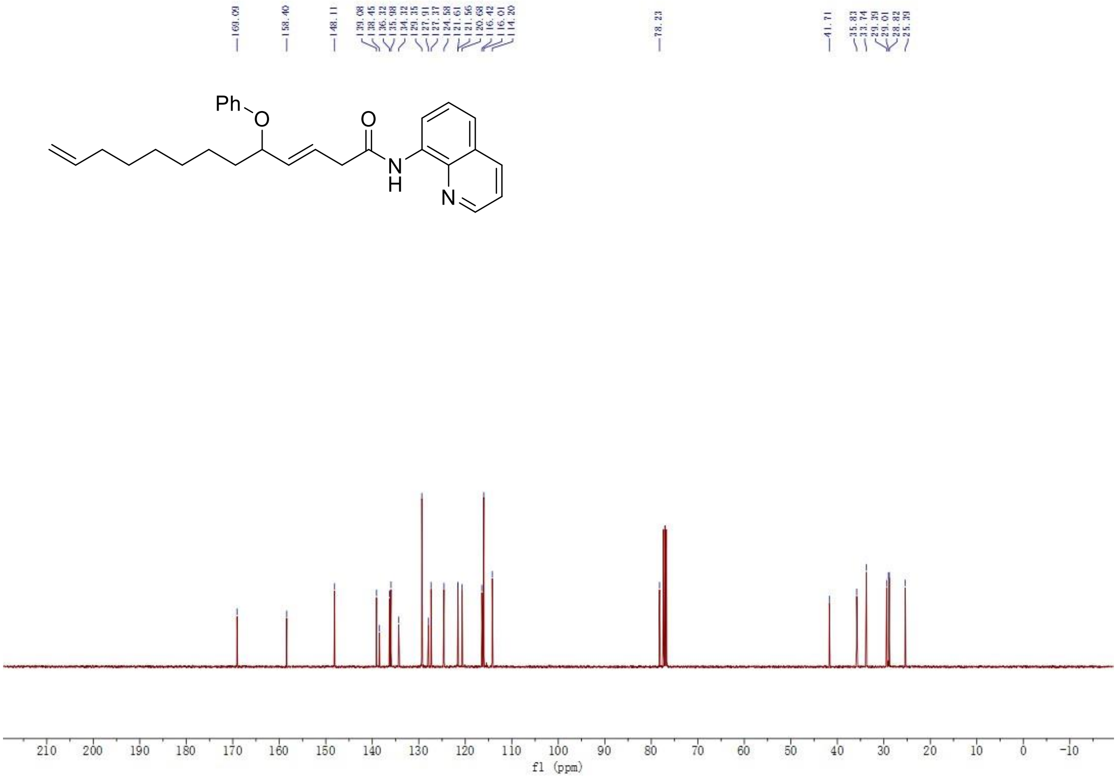

[1]**H NMR**-spectrum (400 MHz, $CDCl_3$) of **17**

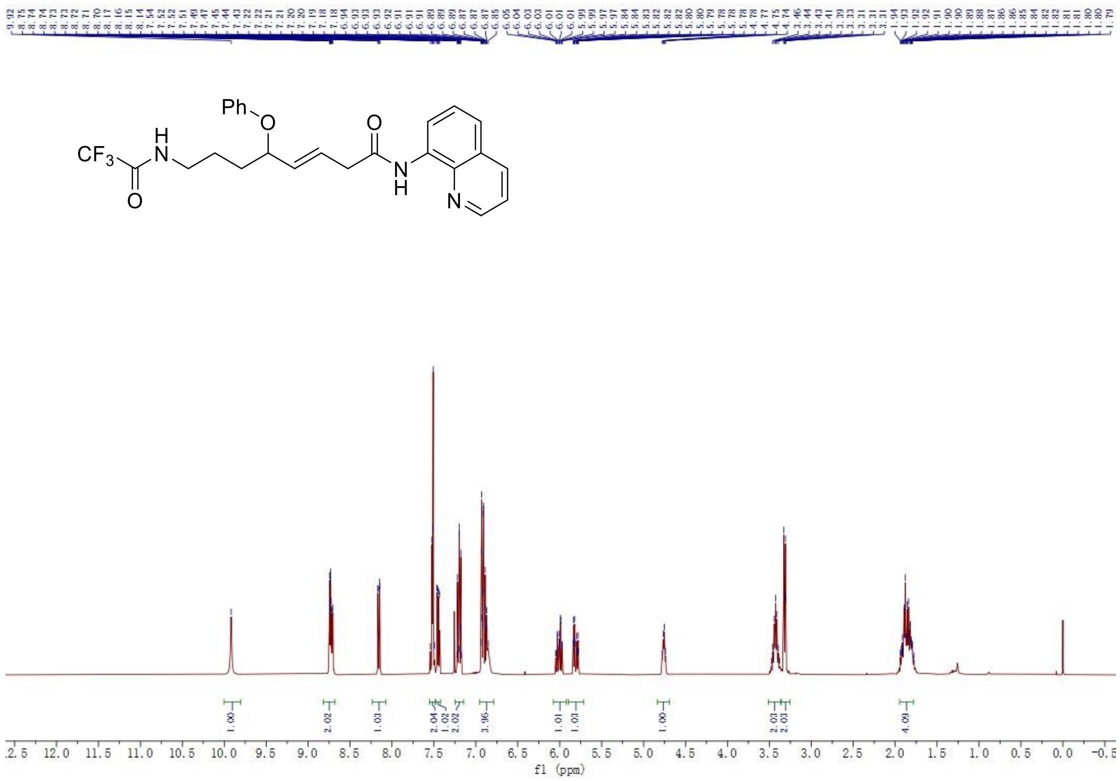

[13]**C NMR**-spectrum (101 MHz, $CDCl_3$) of **17**

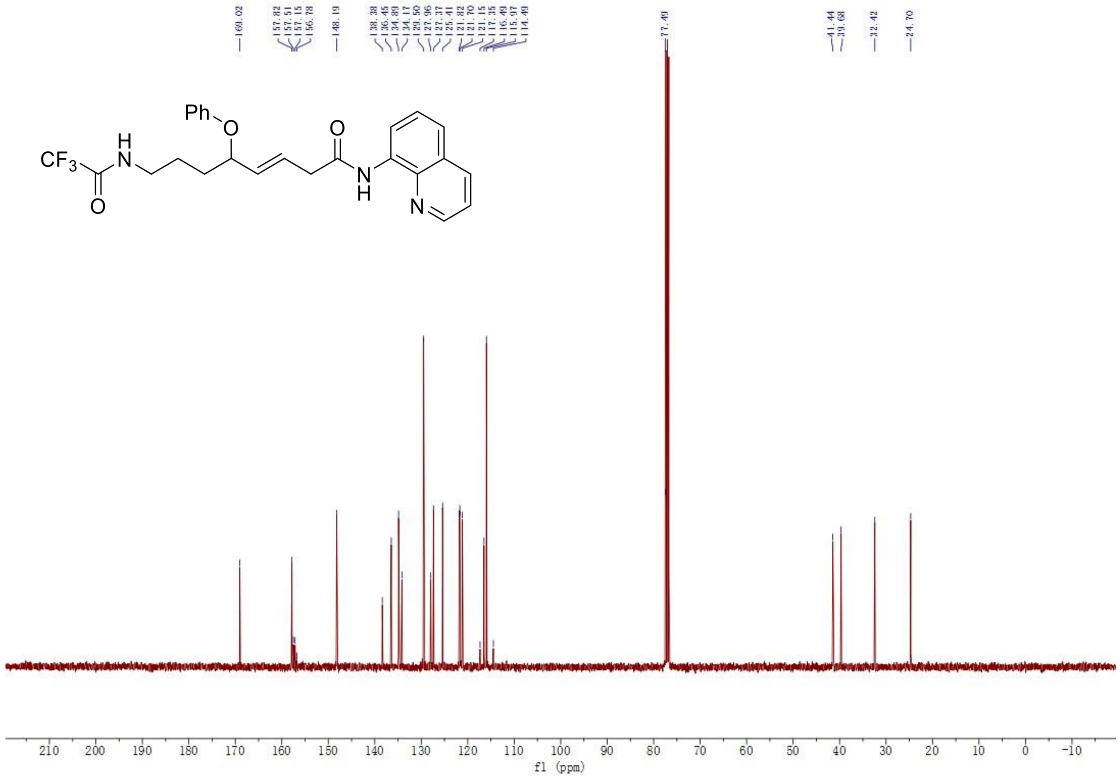

^{19}**F NMR**-spectrum (376 MHz, $CDCl_3$) of **17**

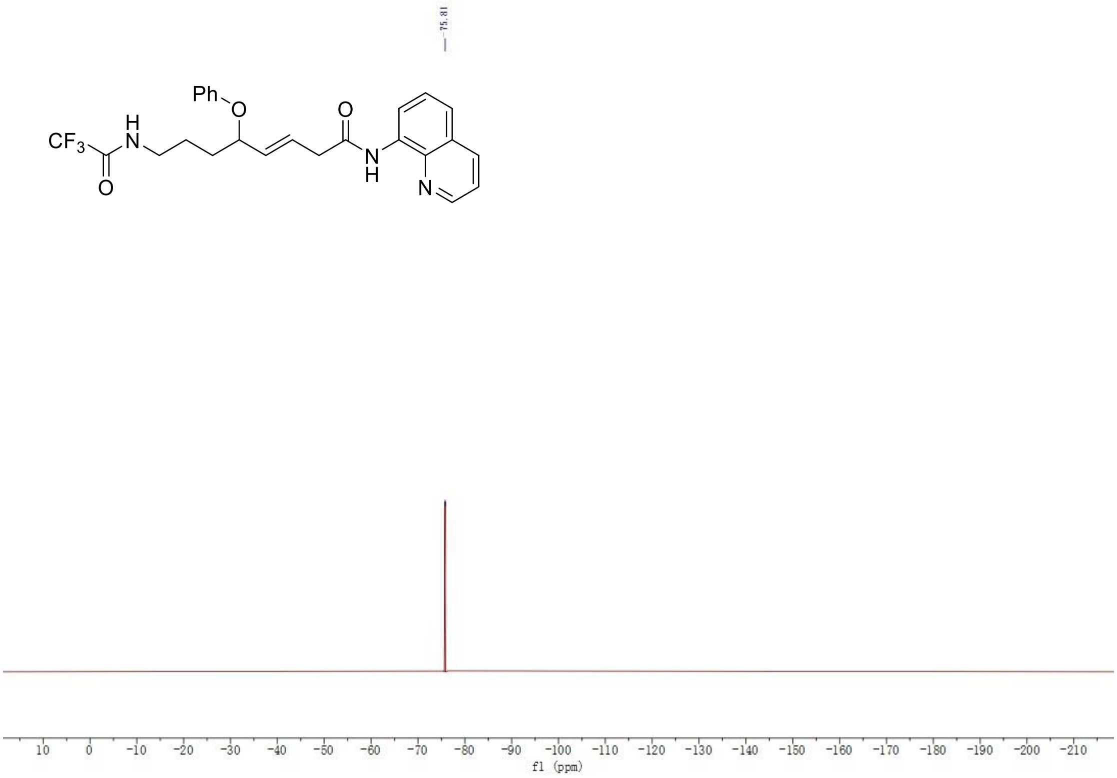

^{1}**H NMR**-spectrum (400 MHz, $CDCl_3$) of **18**

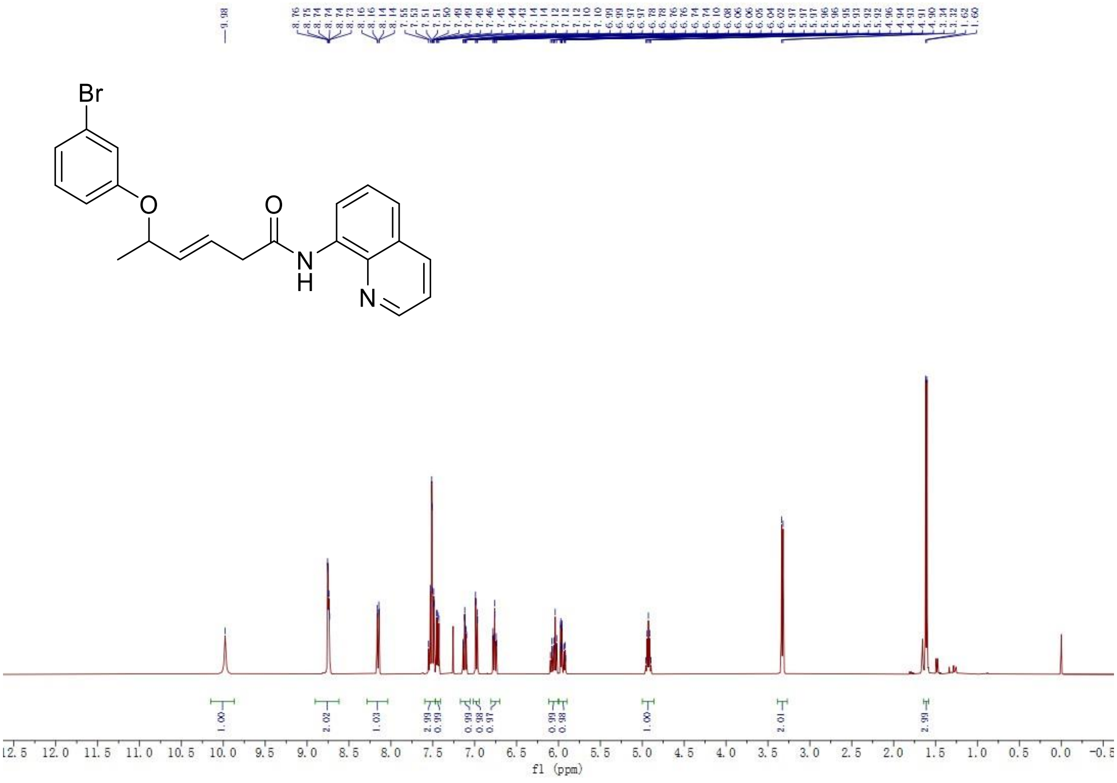

^{13}**C NMR**-spectrum (101 MHz, $CDCl_3$) of **18**

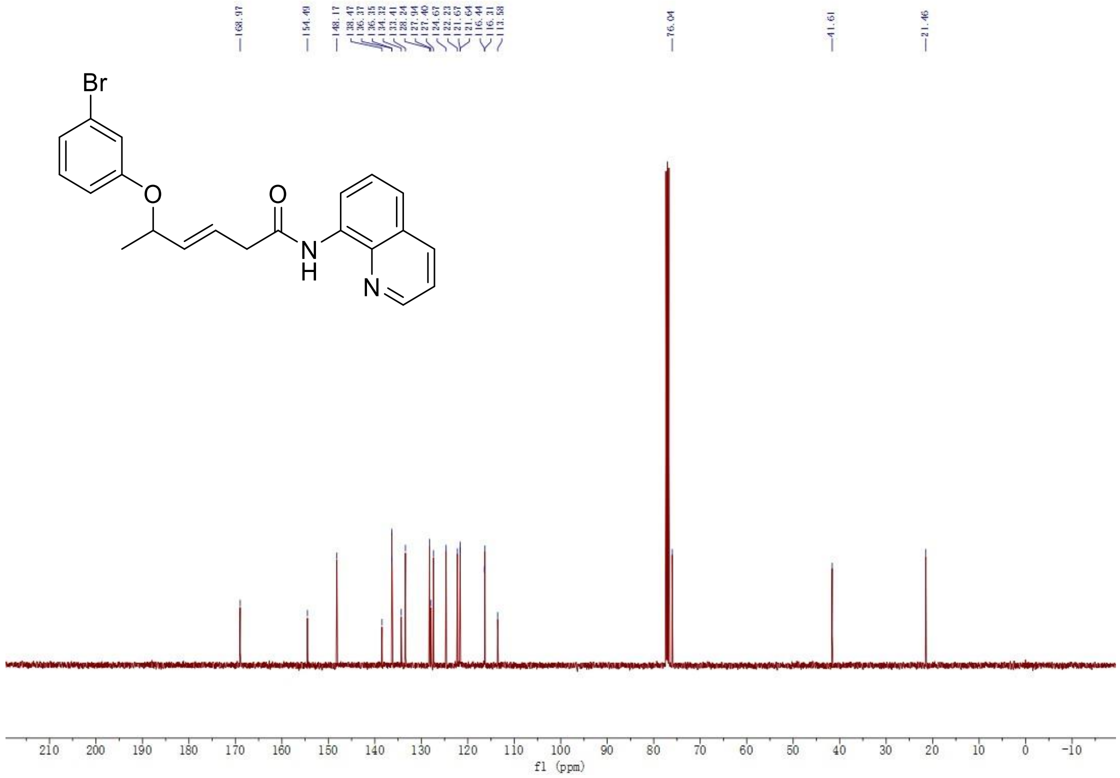

^{1}**H NMR**-spectrum (400 MHz, $CDCl_3$) of **19**

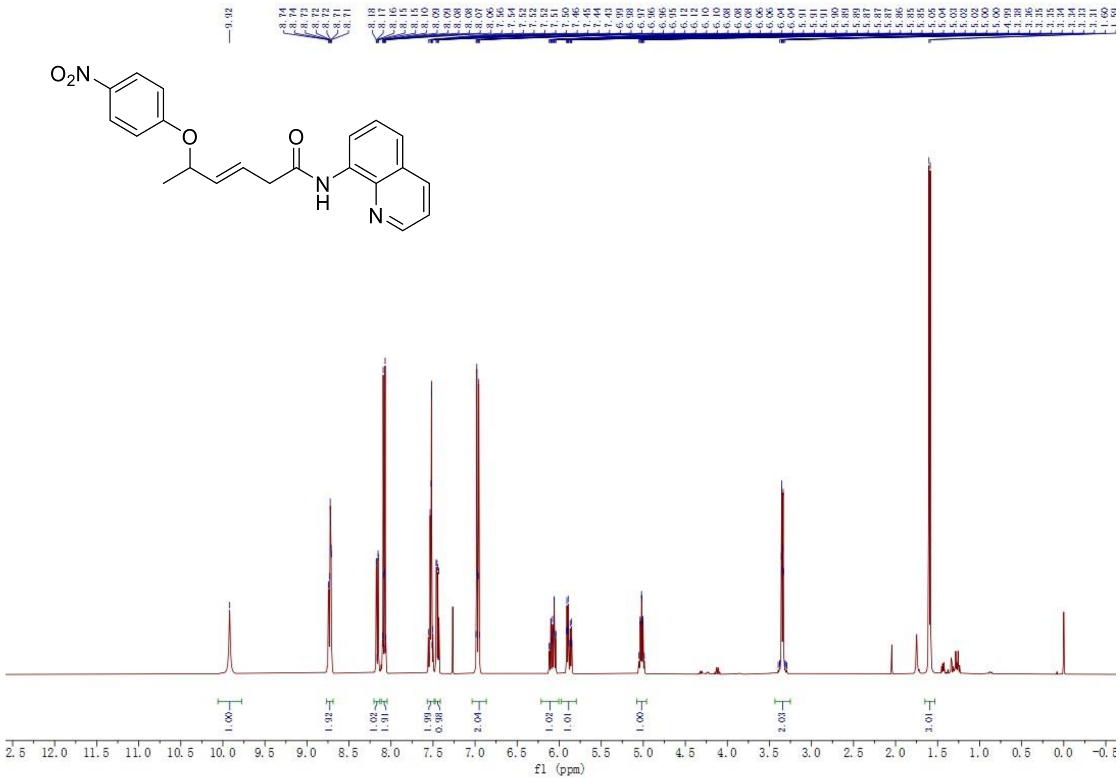

^{13}**C NMR**-spectrum (101 MHz, $CDCl_3$) of **19**

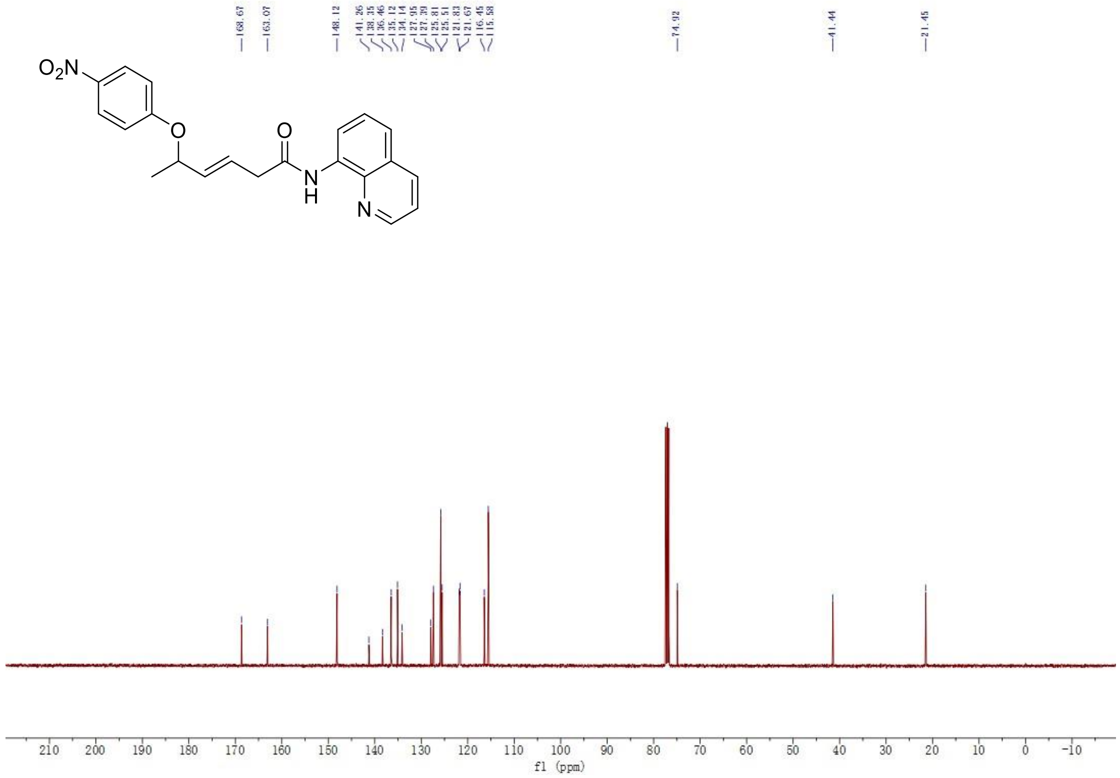

^{1}**H NMR**-spectrum (400 MHz, $CDCl_3$) of **20**

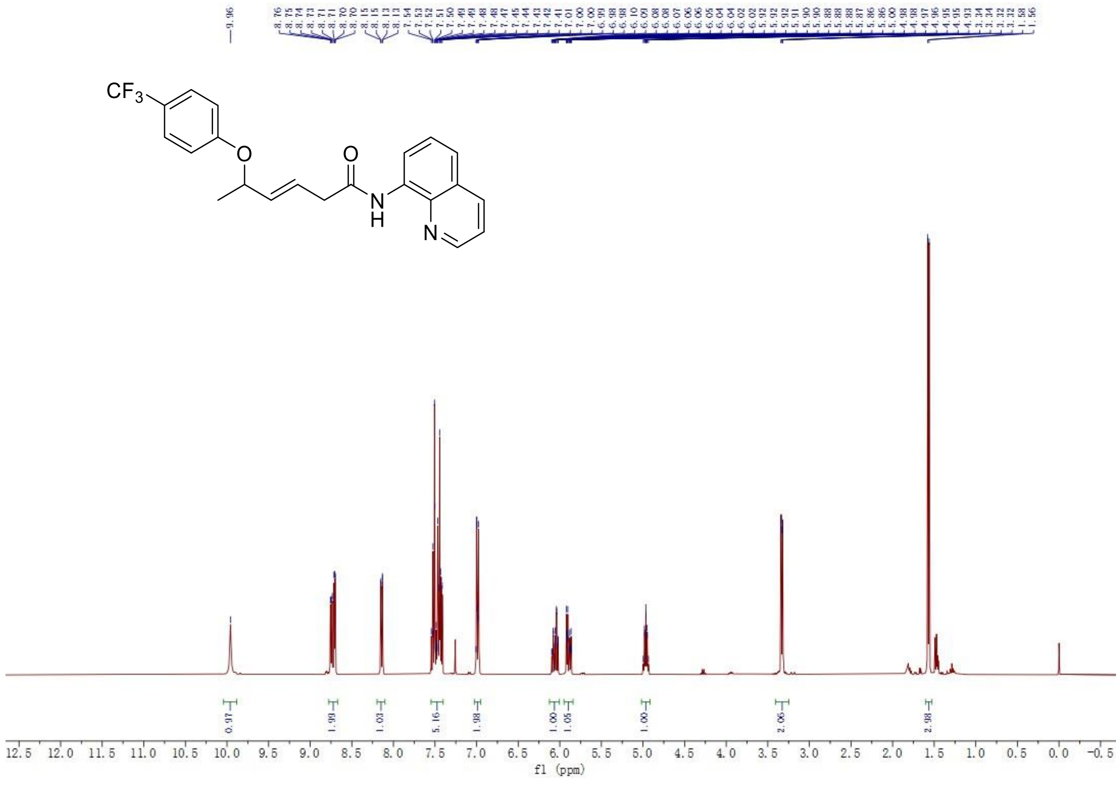

^{13}C NMR-spectrum (101 MHz, $CDCl_3$) of **20**

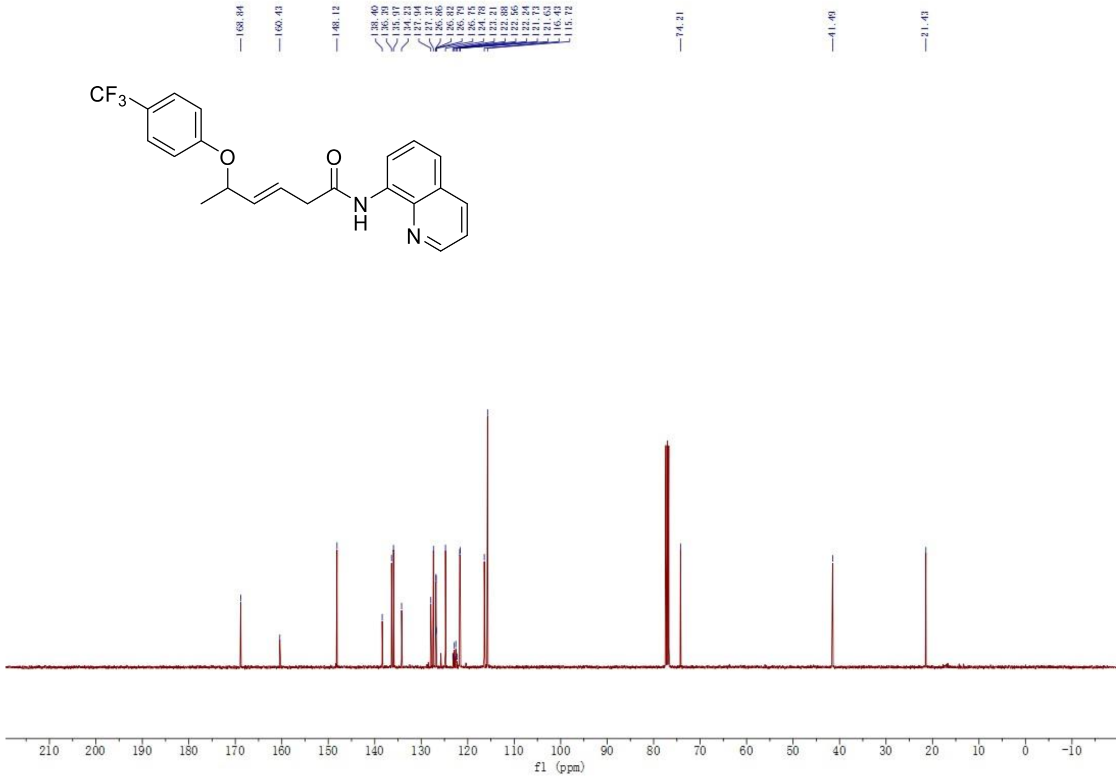

^{19}F NMR-spectrum (376 MHz, $CDCl_3$) of **20**

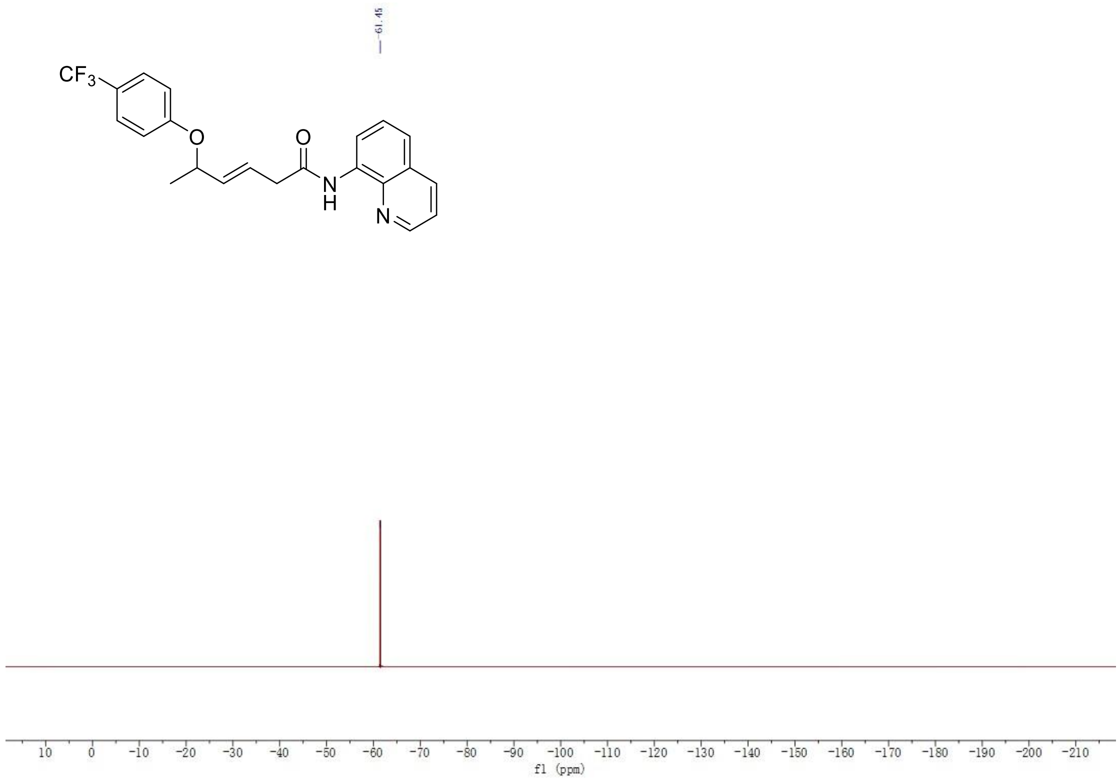

^{1}**H NMR**-spectrum (400 MHz, $CDCl_3$) of **21**

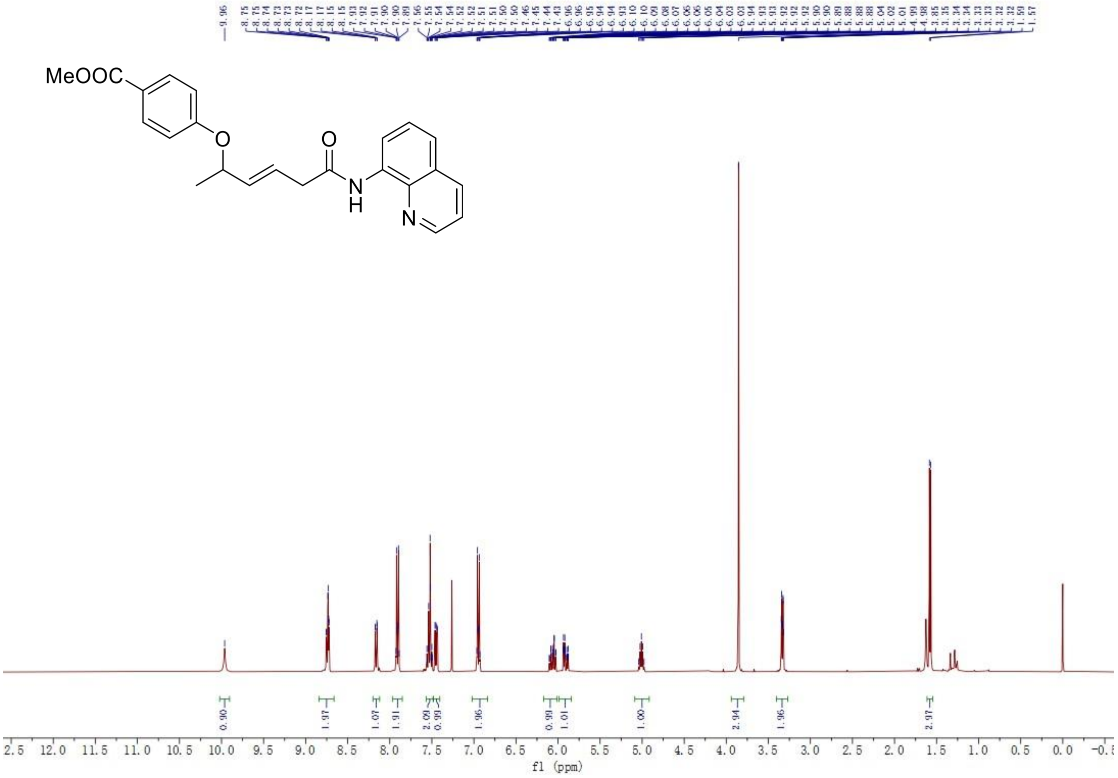

^{13}**C NMR**-spectrum (101 MHz, $CDCl_3$) of **21**

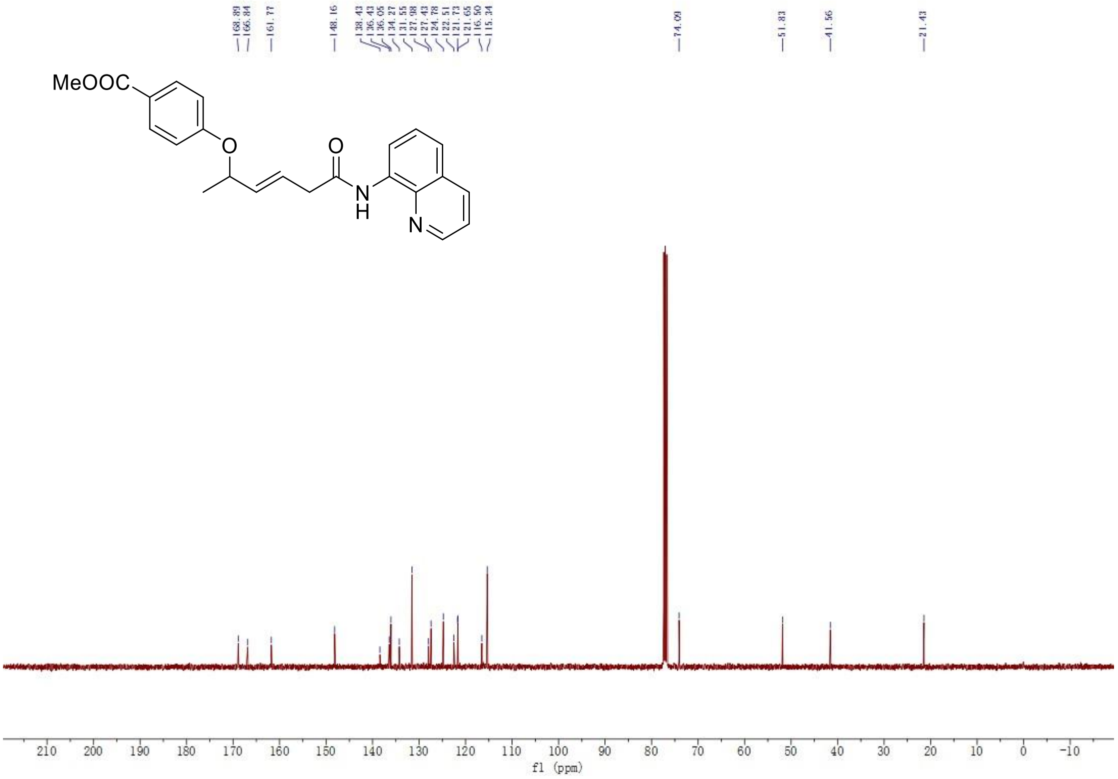

^{1}H NMR-spectrum (400 MHz, CDCl3) of **22**

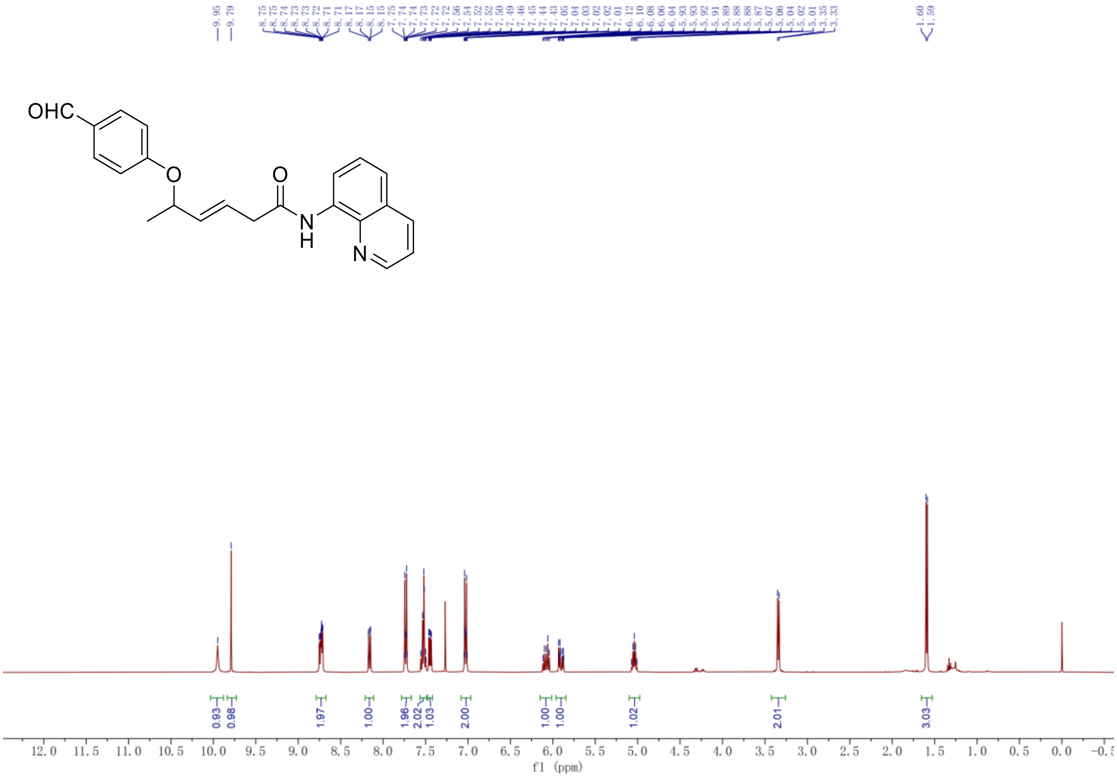

^{13}C NMR-spectrum (101 MHz, CDCl3) of **22**

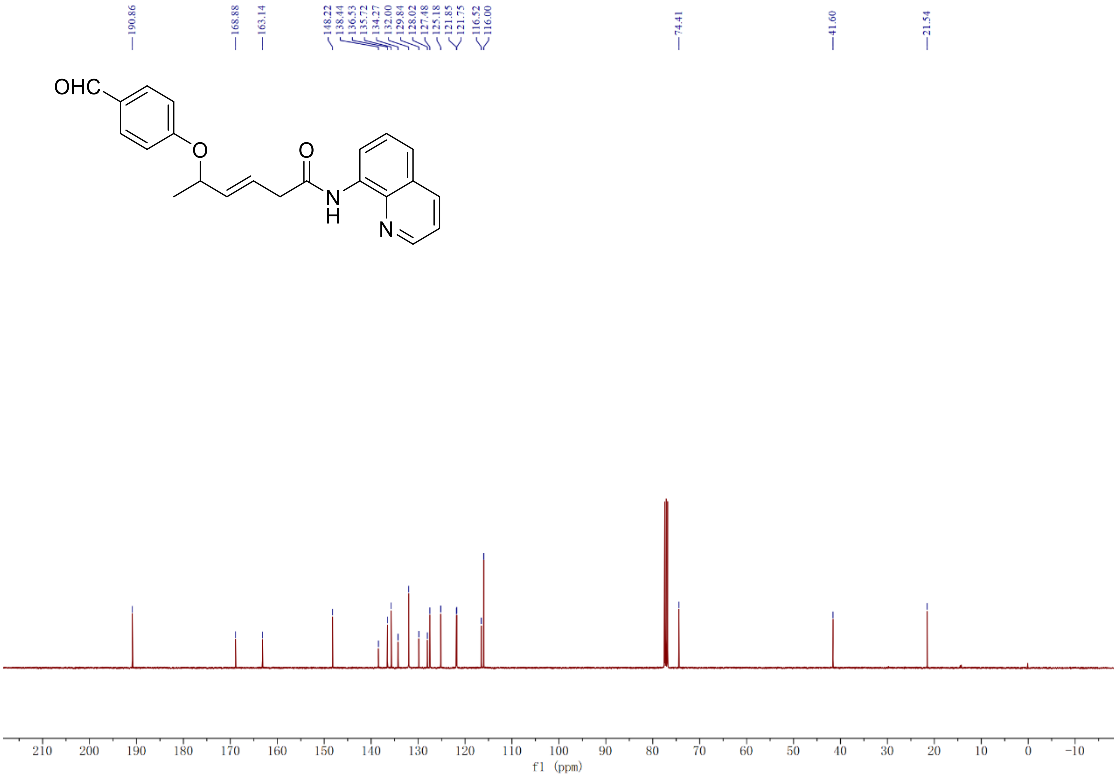

[1]**H NMR**-spectrum (400 MHz, CDCl3) of **23**

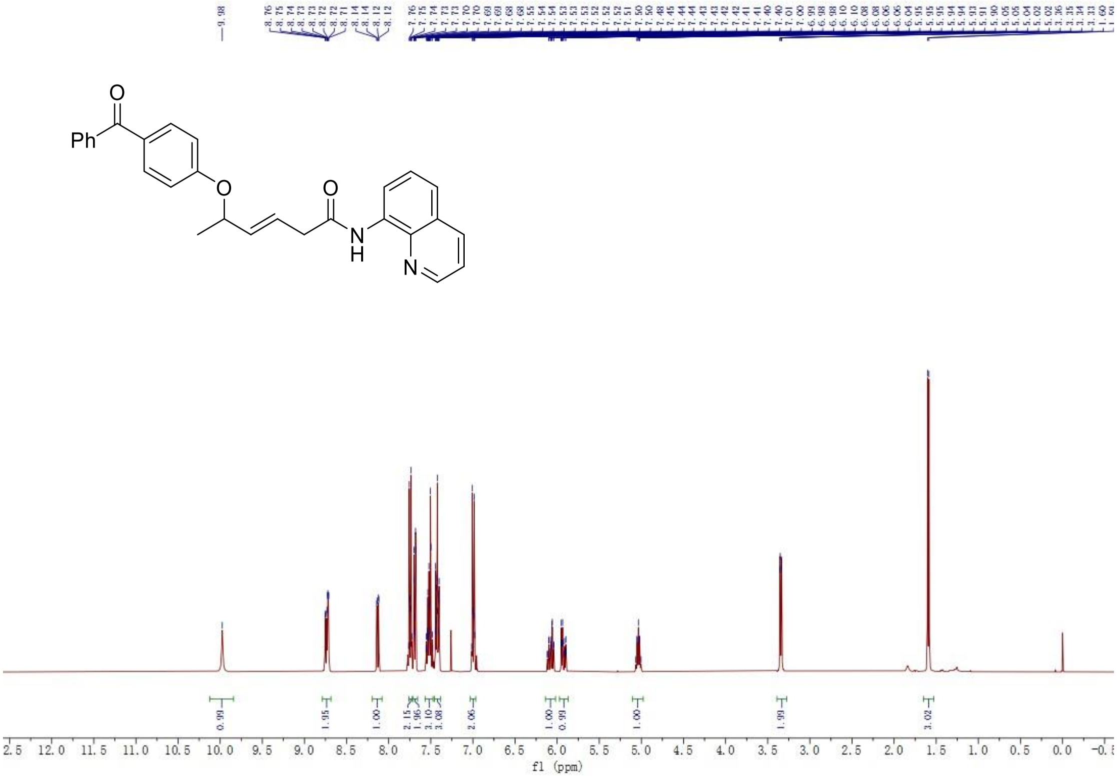

[13]**C NMR**-spectrum (101 MHz, CDCl3) of **23**

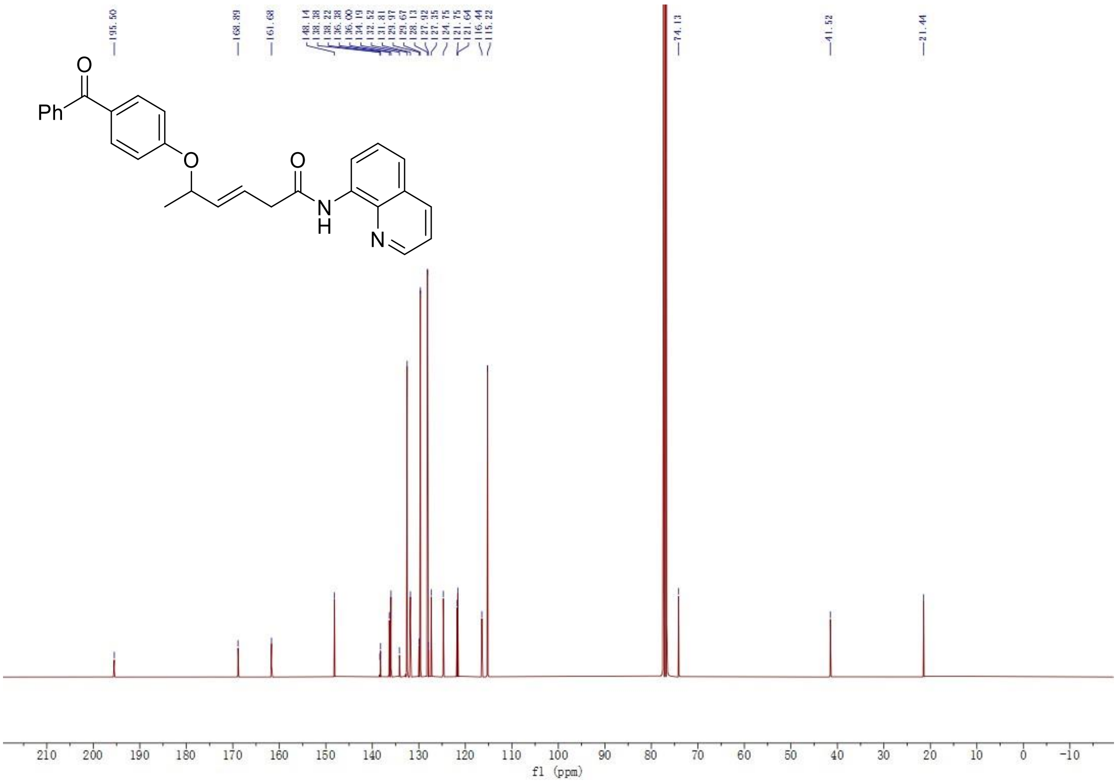

^{1}H NMR-spectrum (400 MHz, $CDCl_3$) of **24**

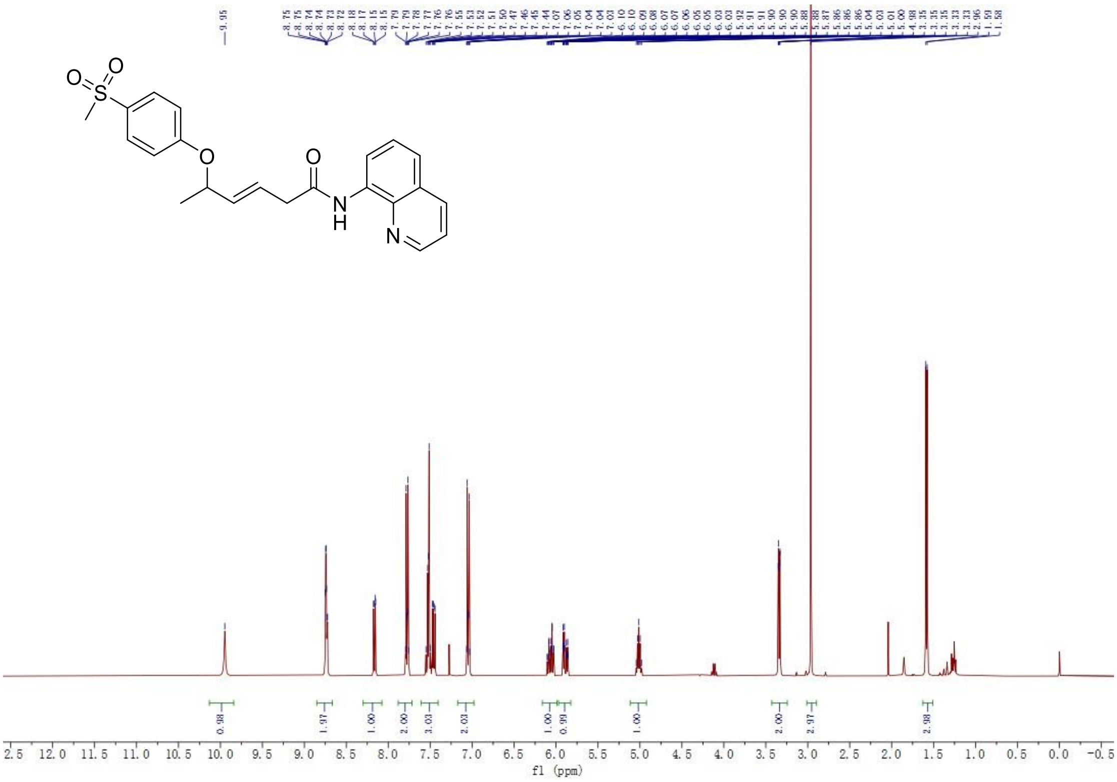

^{13}C NMR-spectrum (101 MHz, $CDCl_3$) of **24**

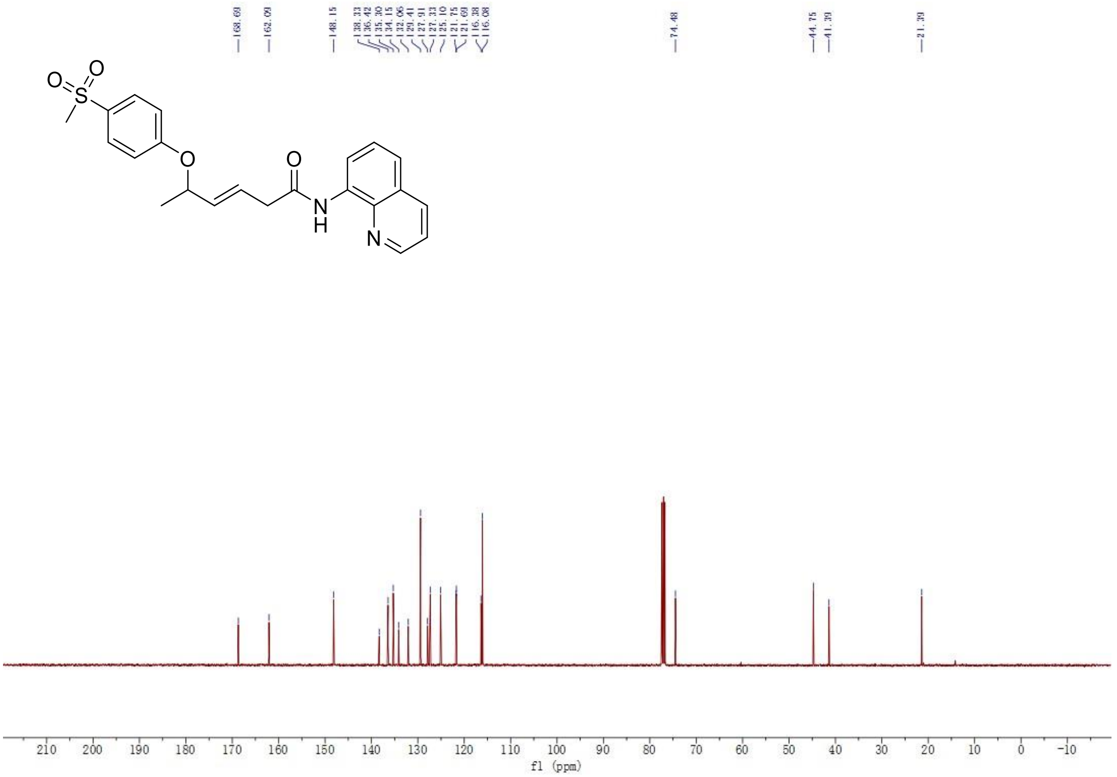

^{1}H NMR-spectrum (400 MHz, $CDCl_3$) of **25**

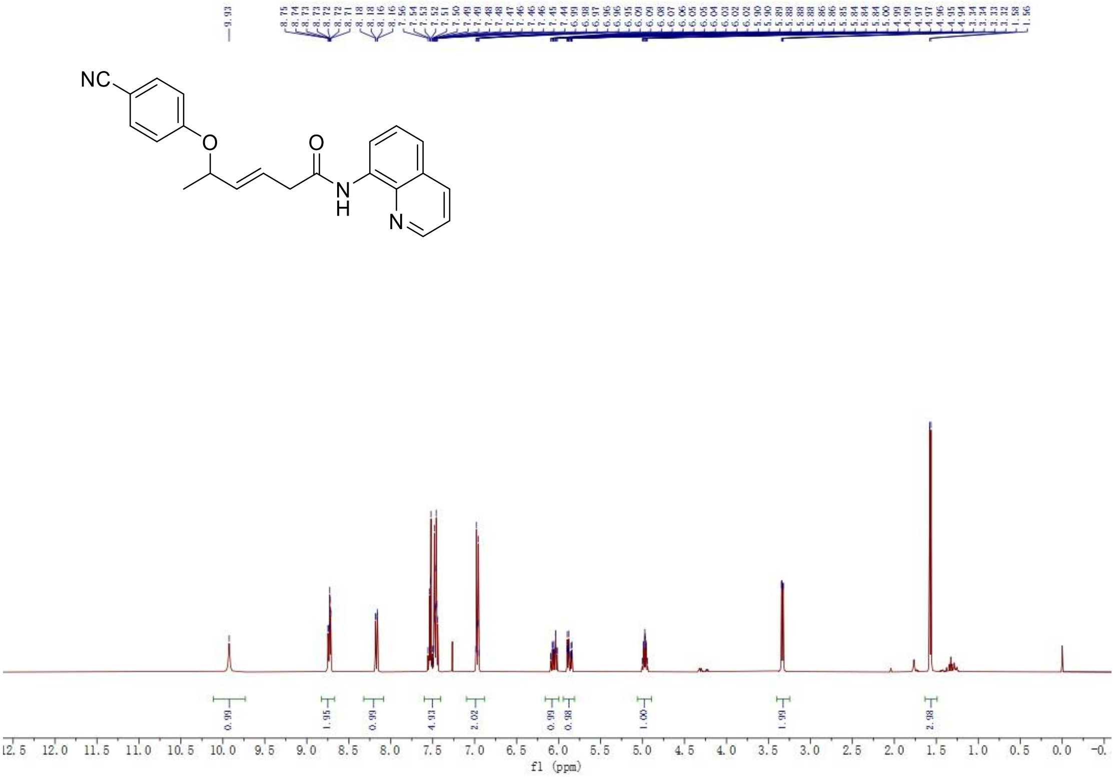

^{13}C NMR-spectrum (101 MHz, $CDCl_3$) of **25**

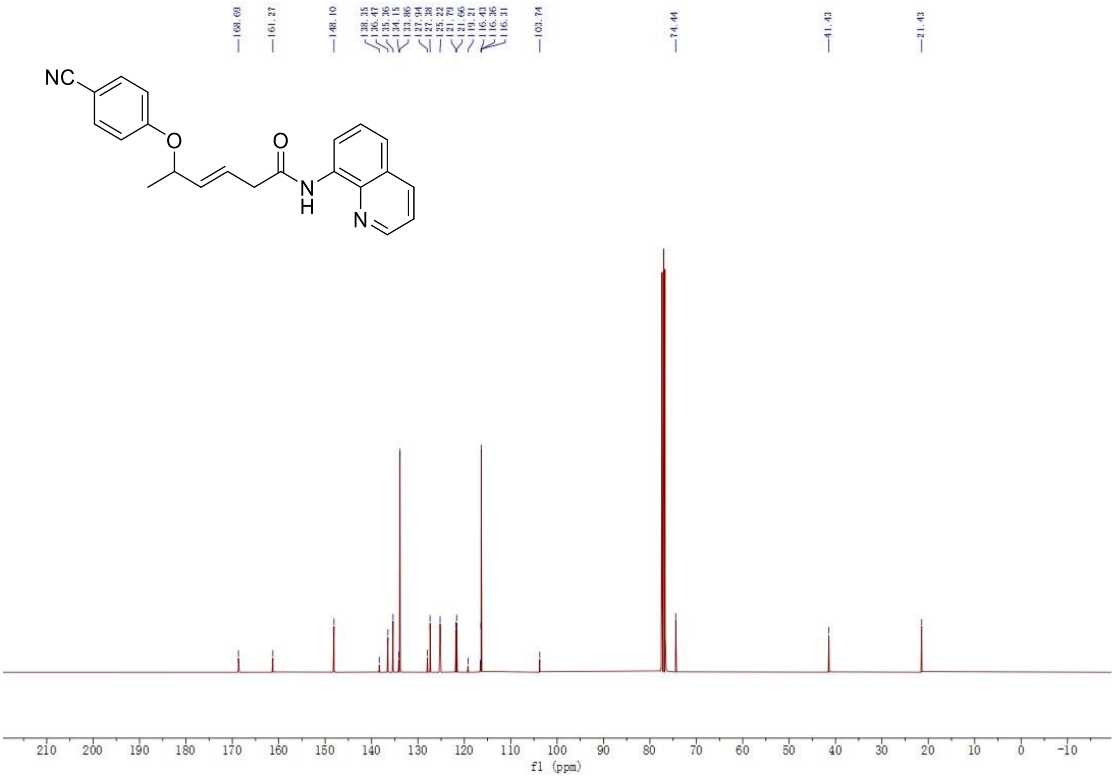

[1]**H NMR**-spectrum (400 MHz, CDCl3) of **26**

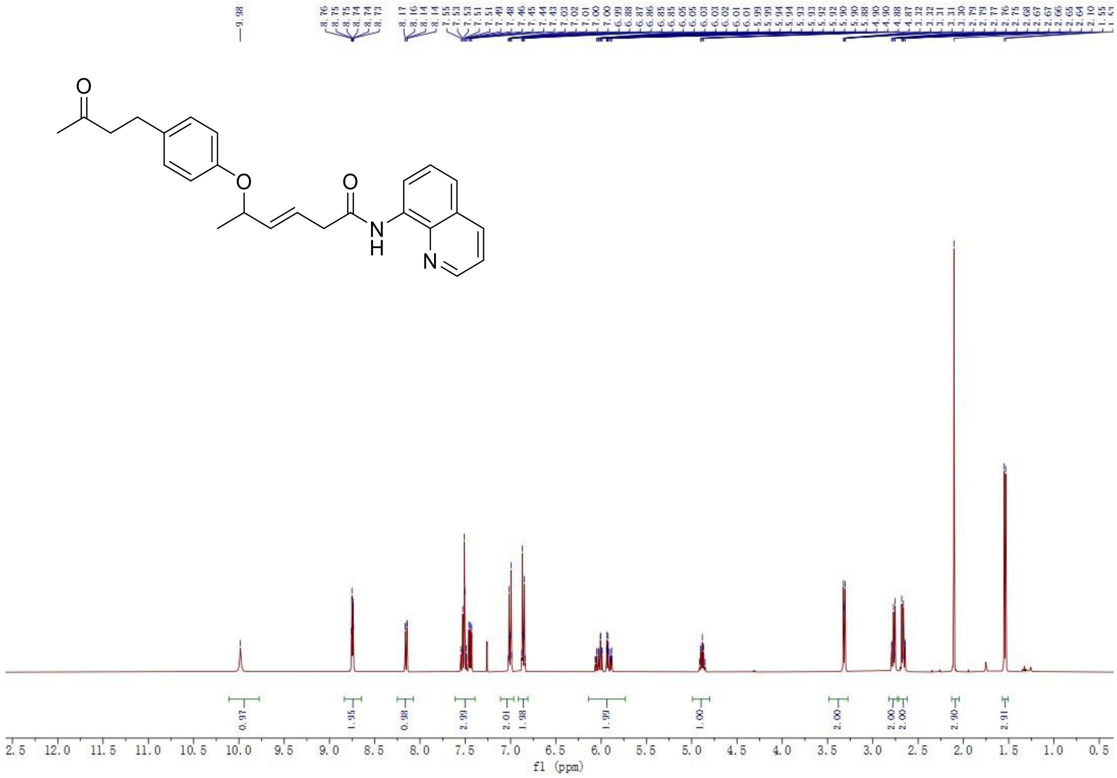

[13]**C NMR**-spectrum (101 MHz, CDCl3) of **26**

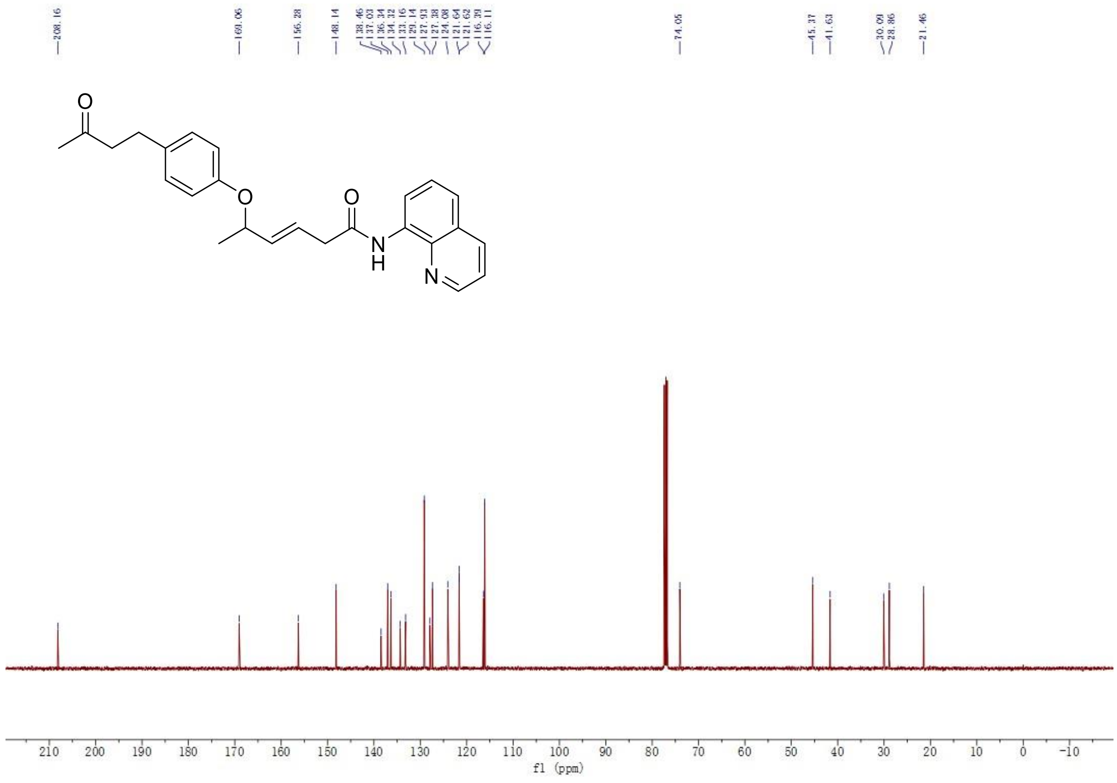

^{1}H NMR-spectrum (400 MHz, CDCl3) of **27**

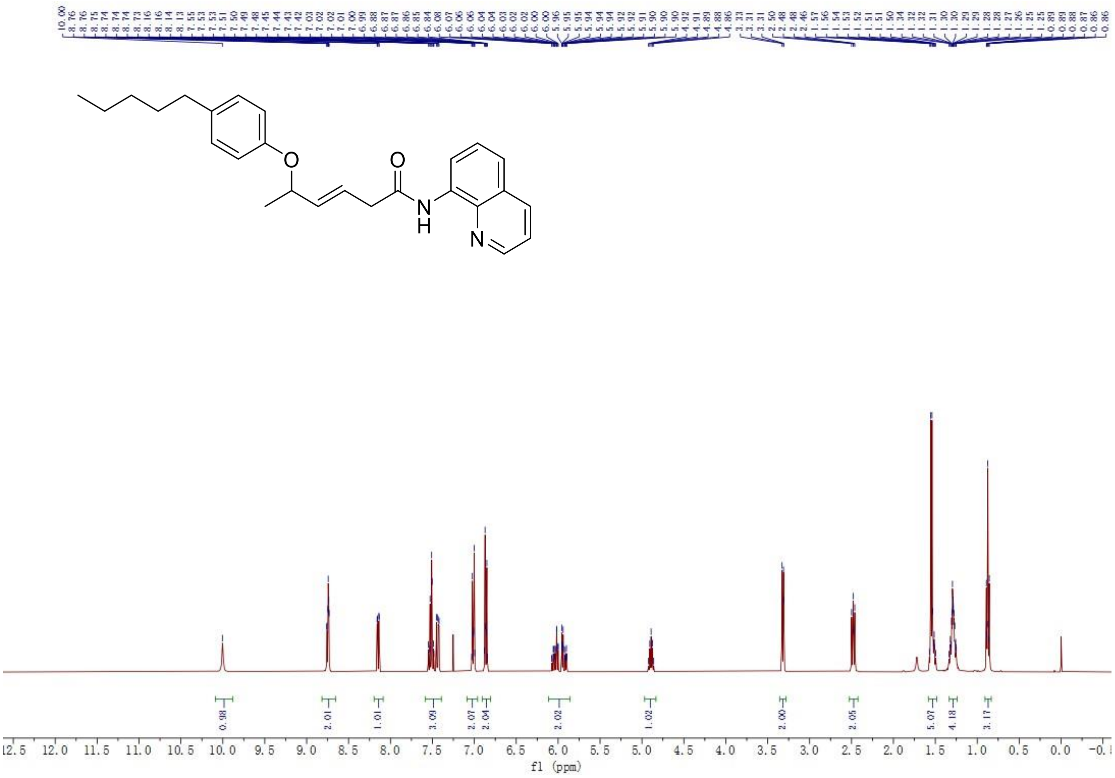

^{13}C NMR-spectrum (101 MHz, CDCl3) of **27**

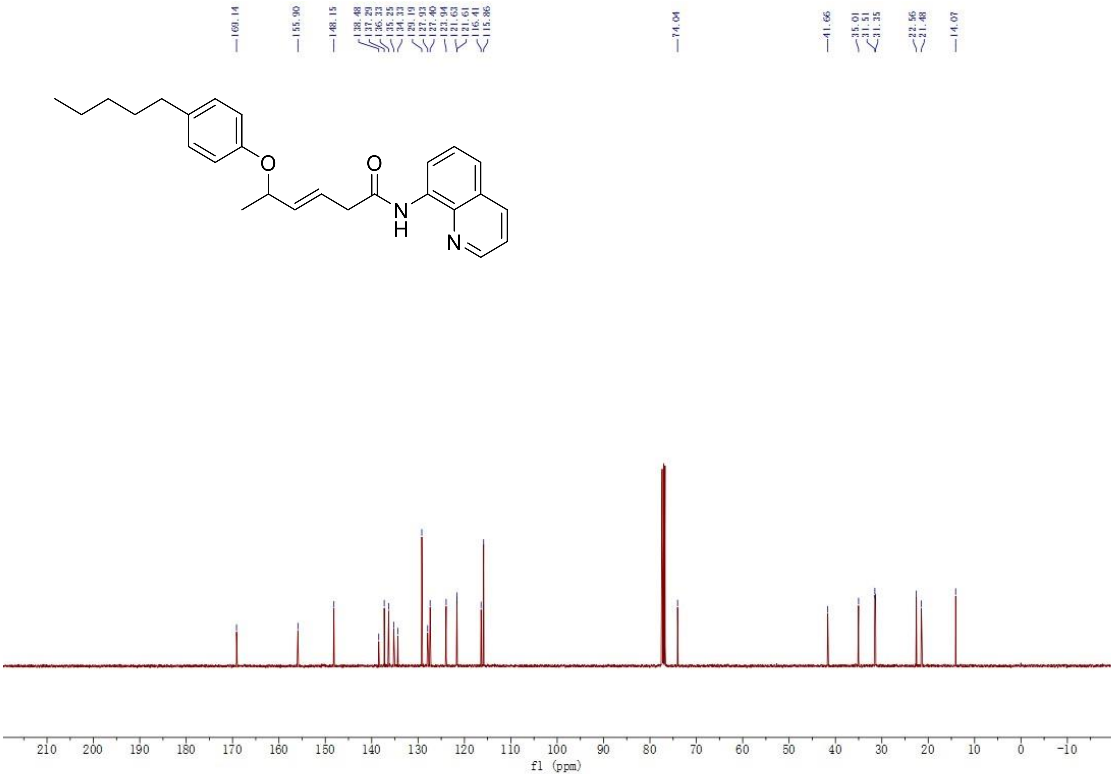

^{1}H NMR-spectrum (400 MHz, CDCl3) of **28**

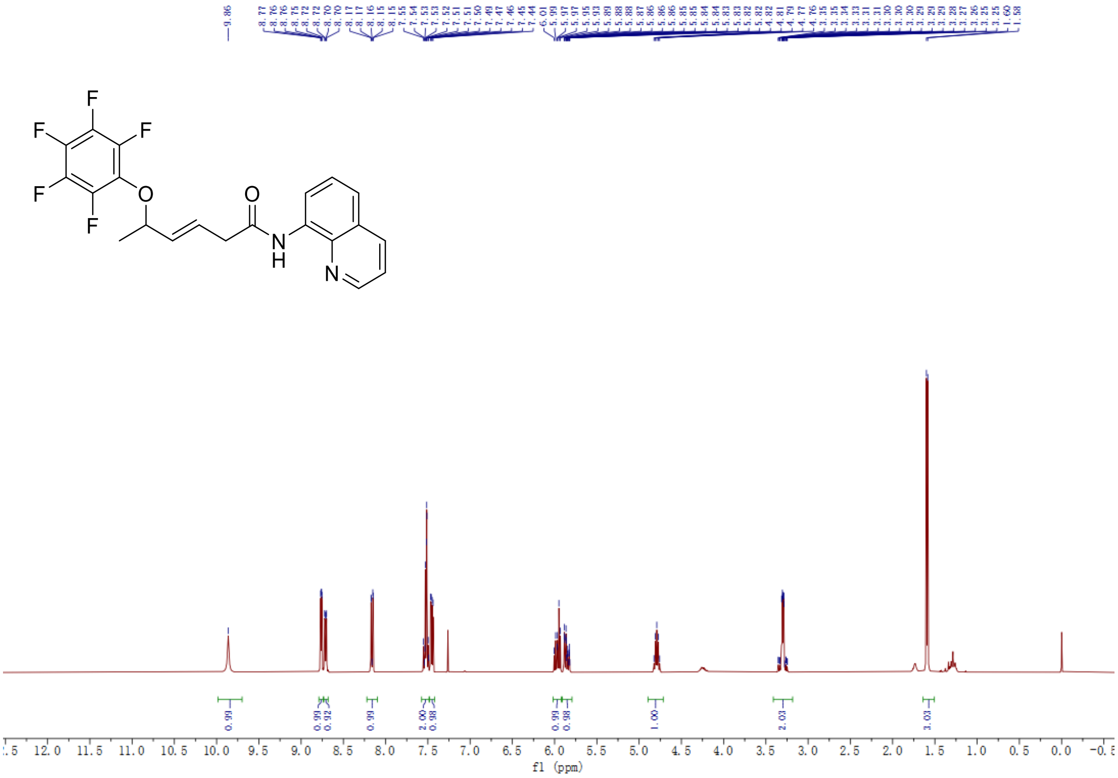

^{13}C NMR-spectrum (101 MHz, CDCl3) of **28**

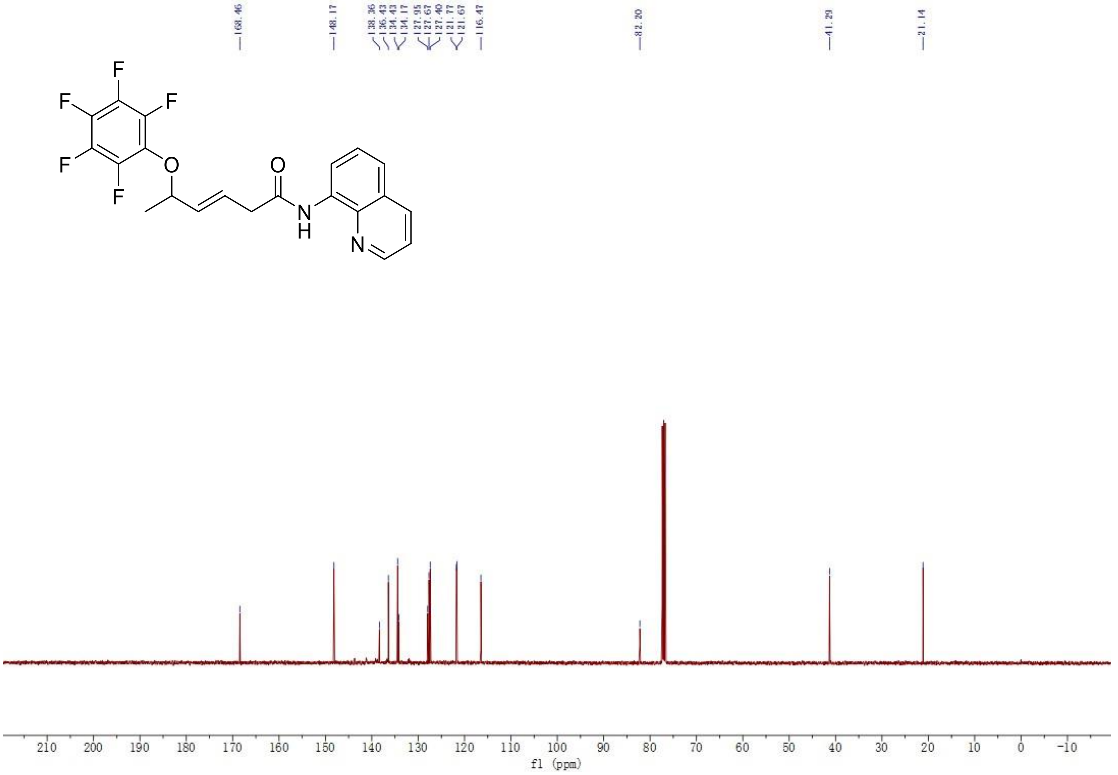

^{19}**F NMR**-spectrum (376 MHz, $CDCl_3$) of **28**

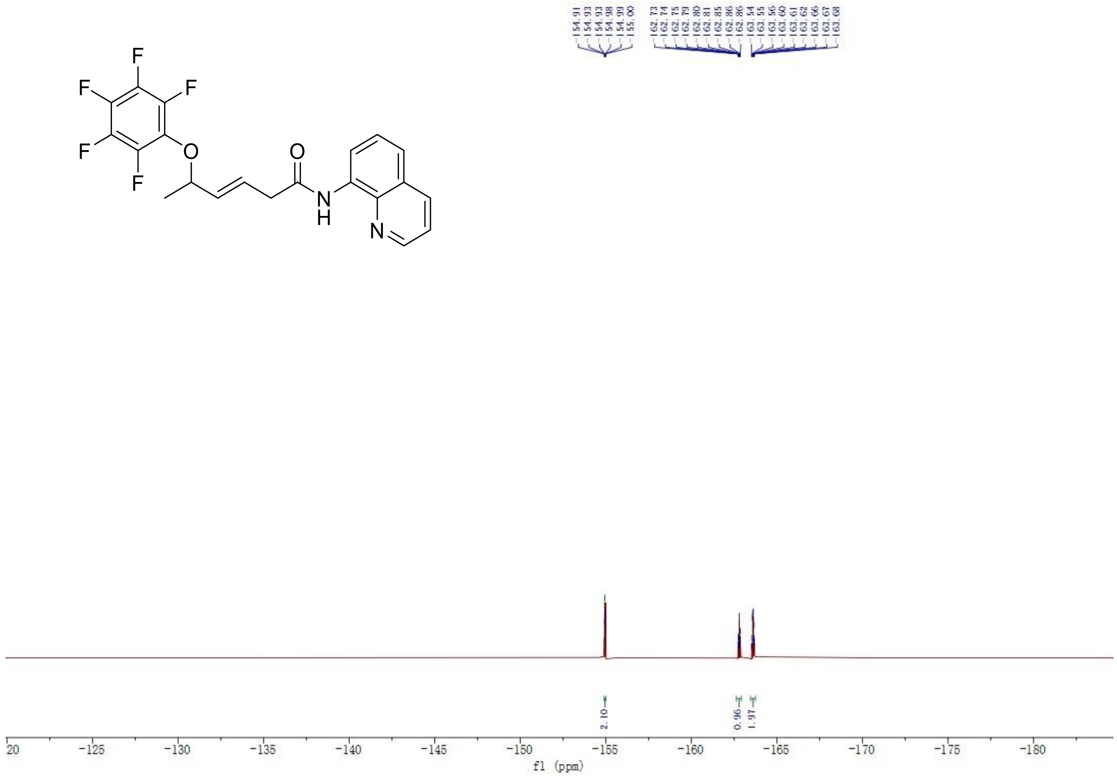

^{1}**H NMR**-spectrum (400 MHz, $CDCl_3$) of **29**

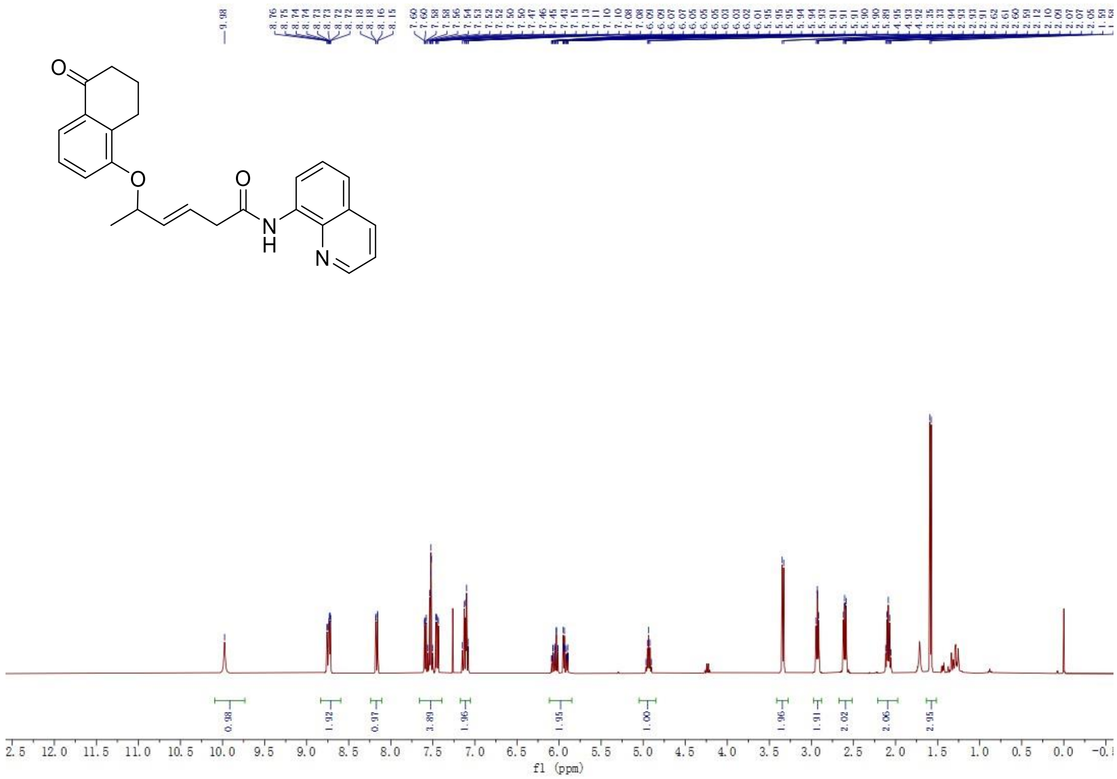

^{13}**C NMR**-spectrum (101 MHz, $CDCl_3$) of **29**

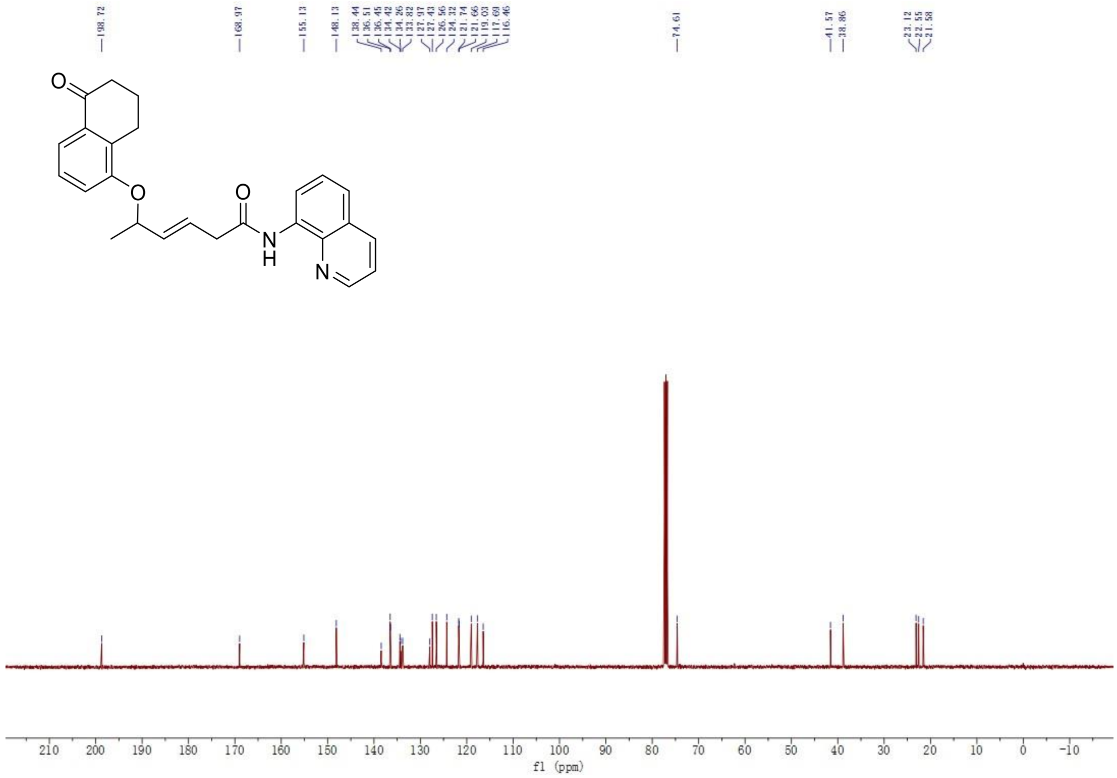

^{1}**H NMR**-spectrum (400 MHz, $CDCl_3$) of **30**

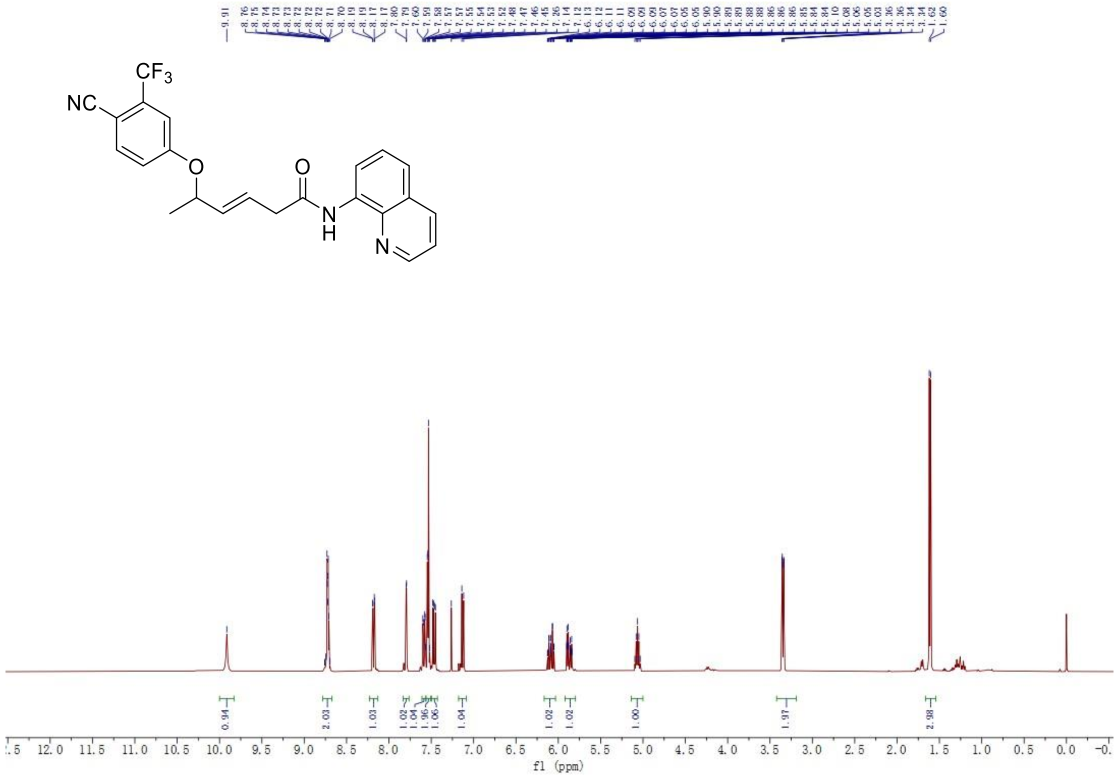

^{13}C NMR-spectrum (101 MHz, $CDCl_3$) of **30**

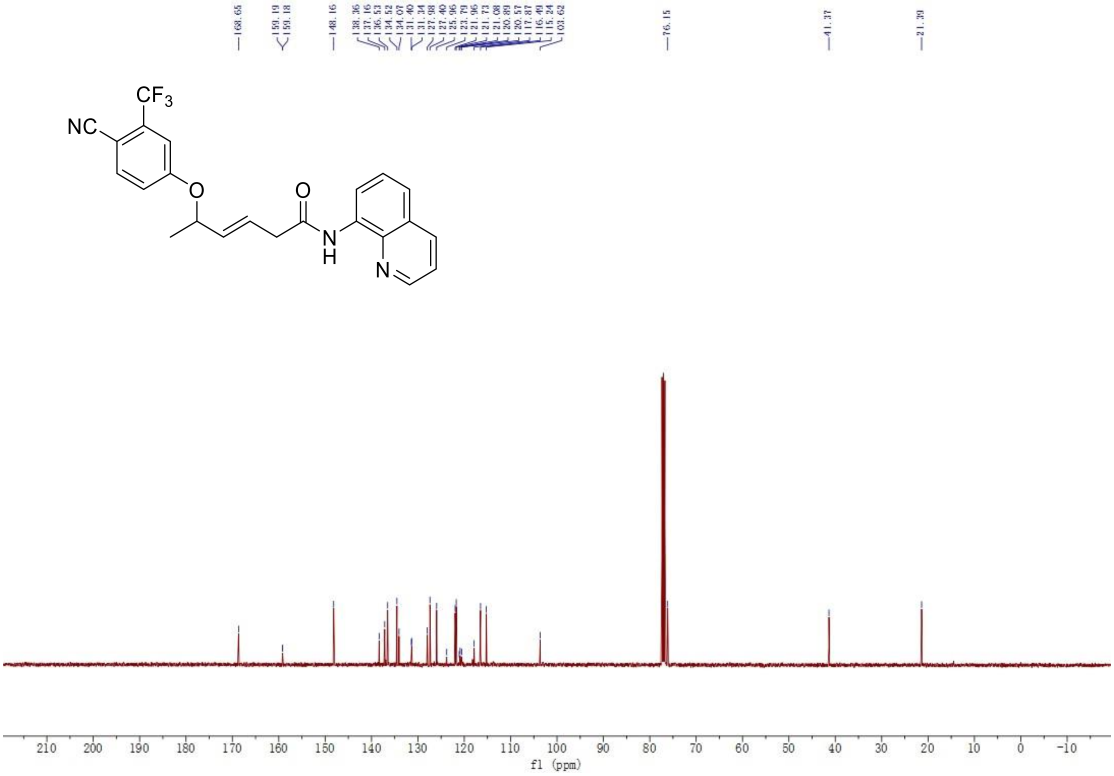

^{19}F NMR-spectrum (376 MHz, $CDCl_3$) of **30**

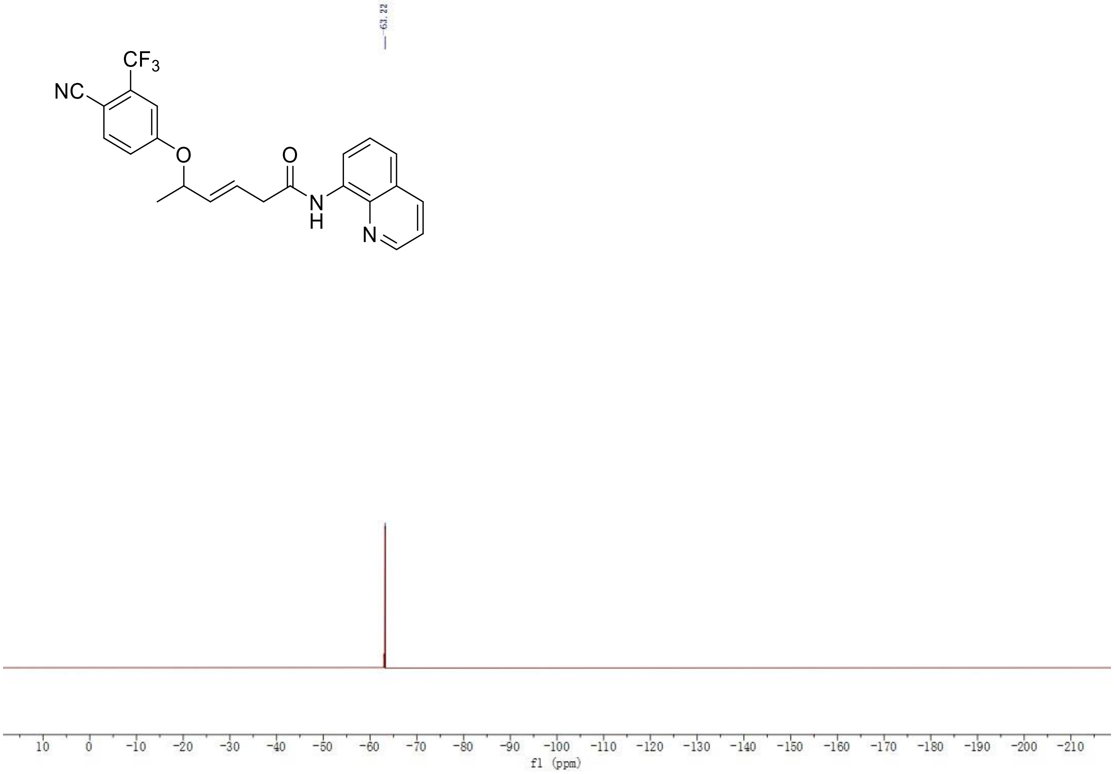

[1]**H NMR**-spectrum (400 MHz, CDCl3) of **31**

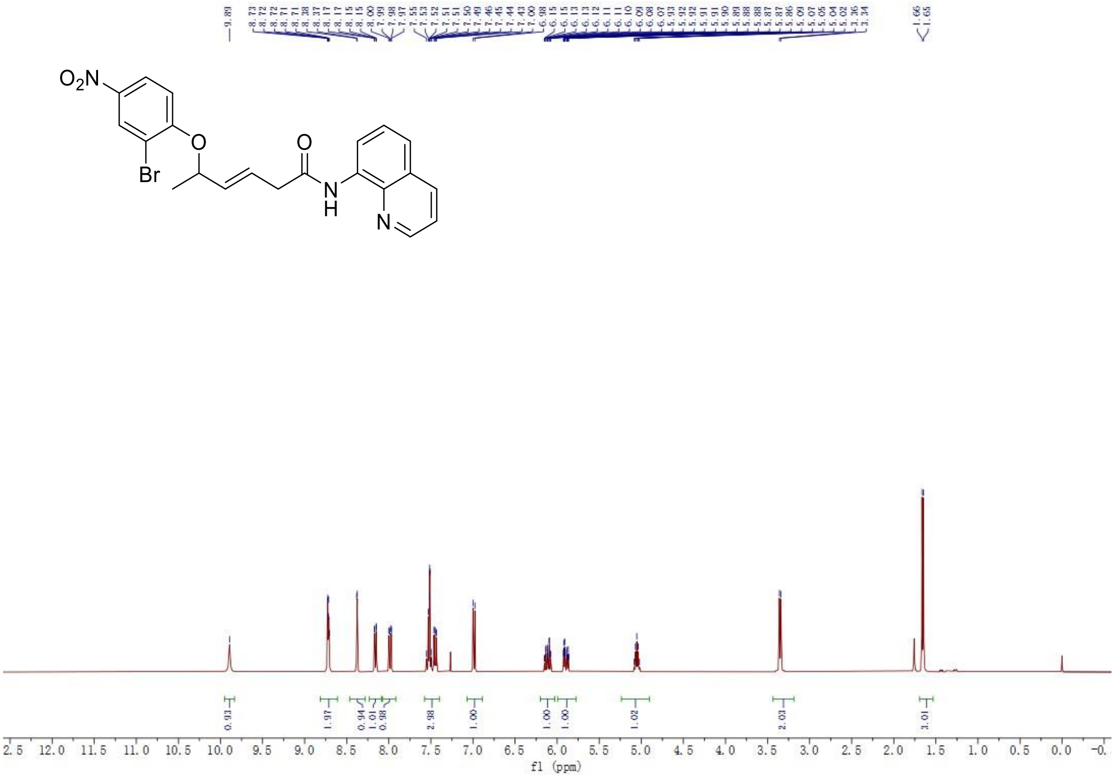

[13]**C NMR**-spectrum (101 MHz, CDCl3) of **31**

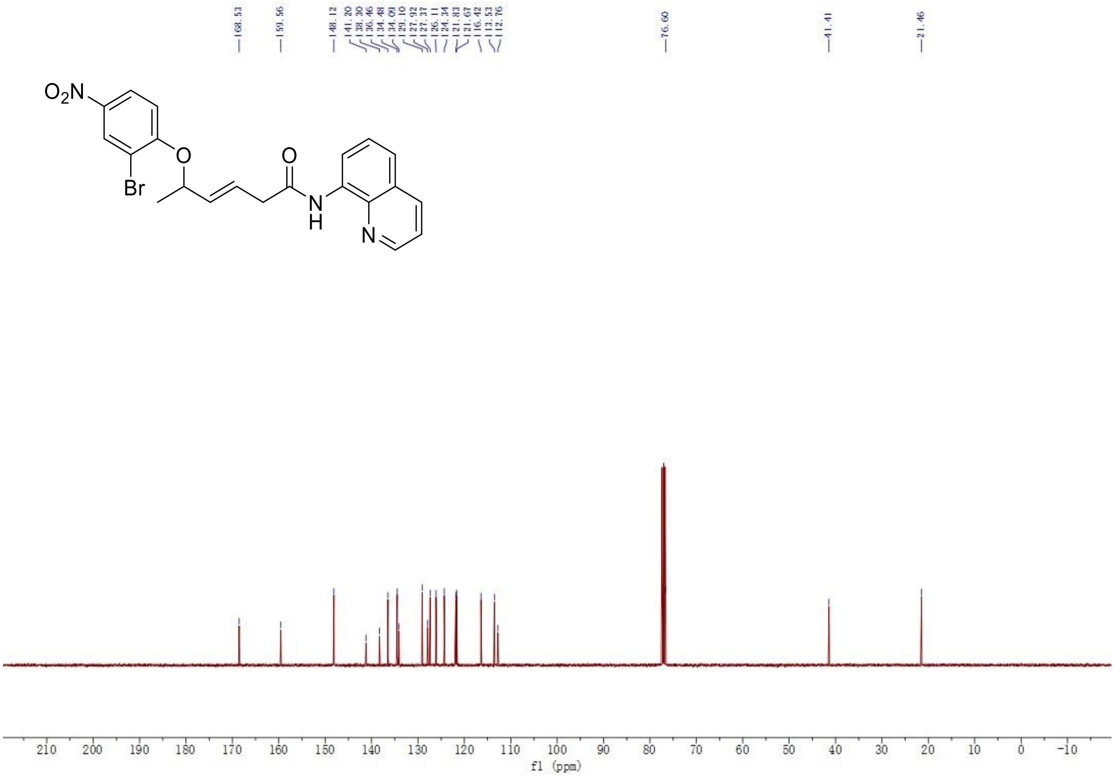

^{1}**H NMR**-spectrum (400 MHz, $CDCl_3$) of **32**

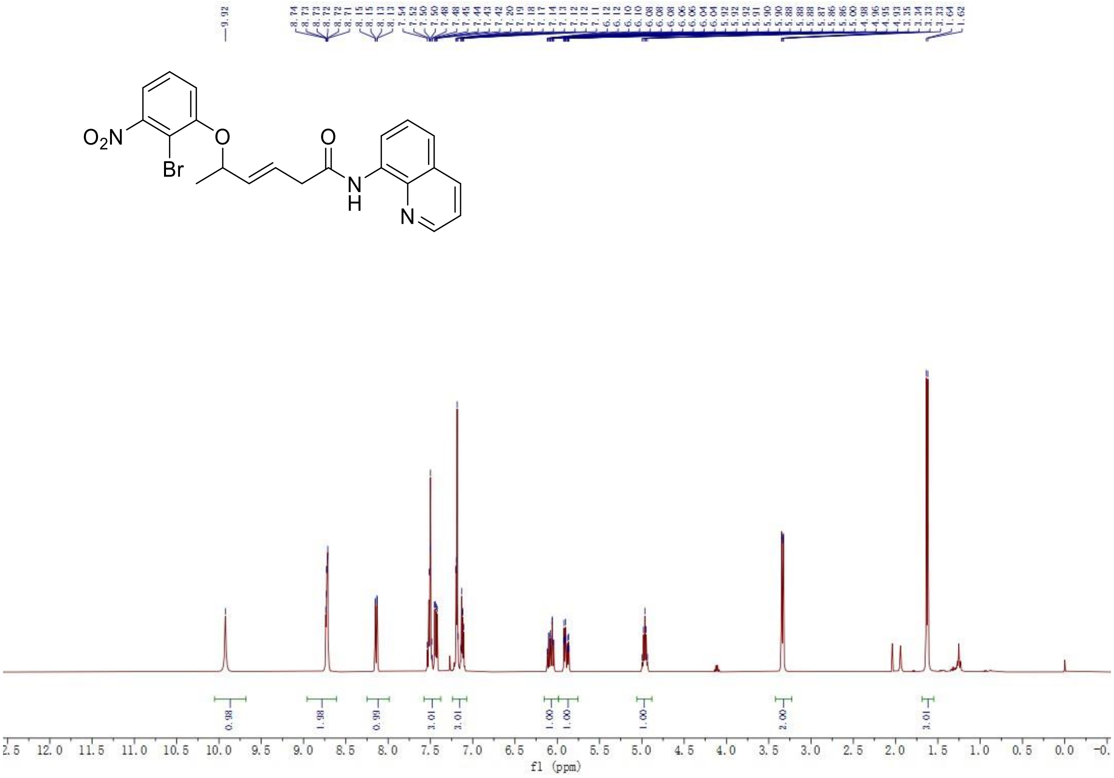

^{13}**C NMR**-spectrum (101 MHz, $CDCl_3$) of **32**

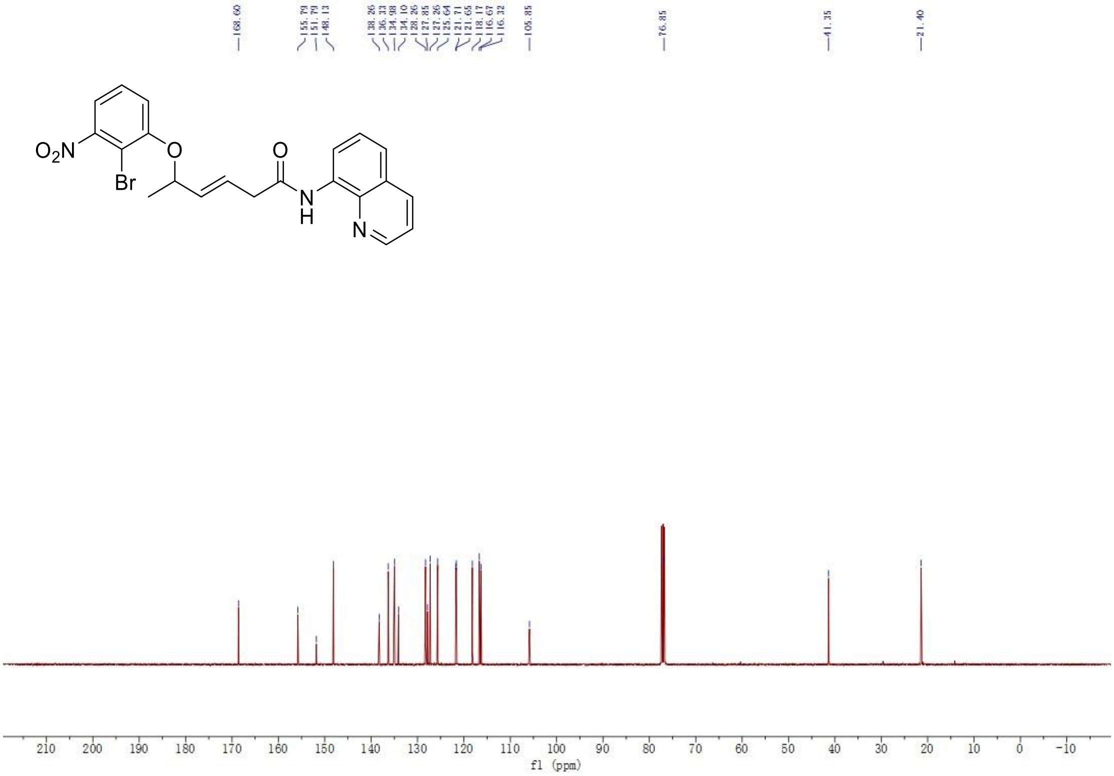

[1]**H NMR**-spectrum (400 MHz, $CDCl_3$) of **33**

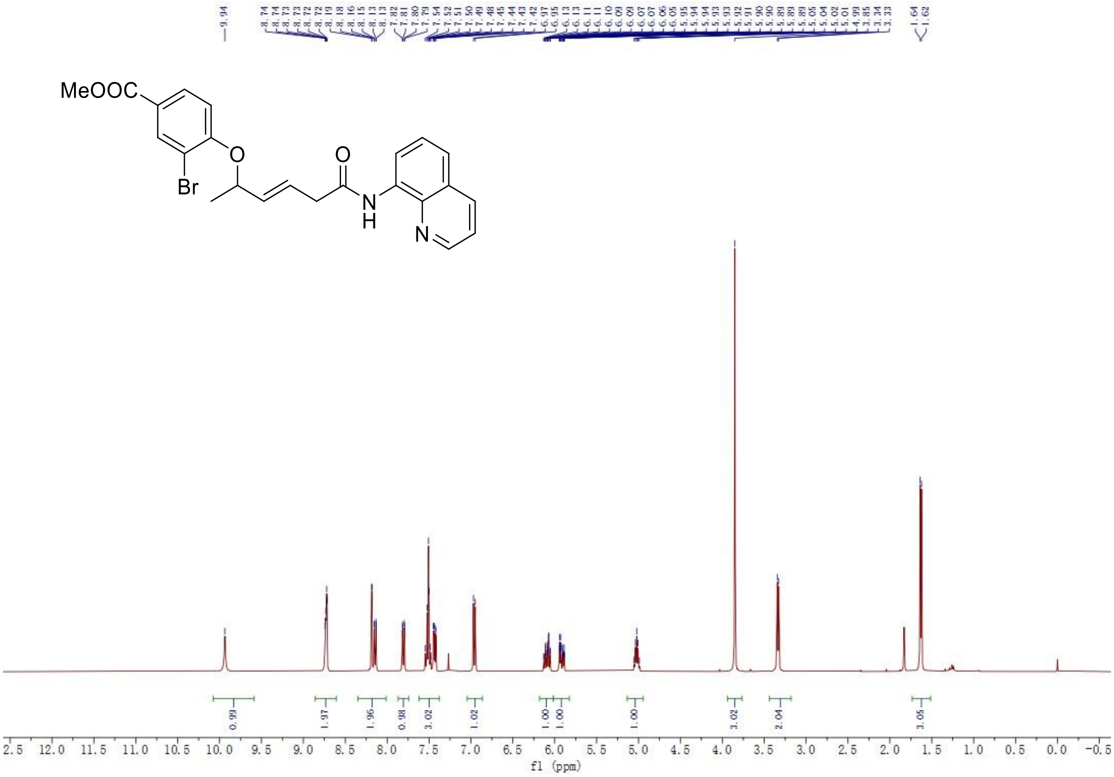

[13]**C NMR**-spectrum (101 MHz, $CDCl_3$) of **33**

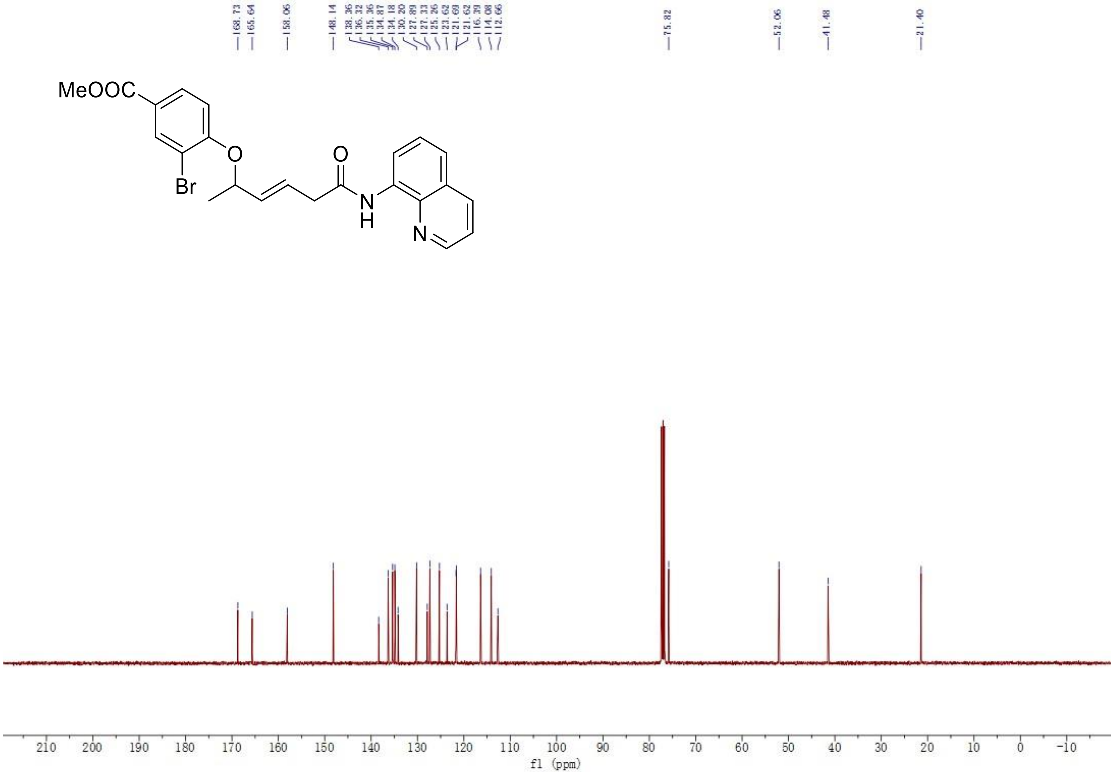

^{1}**H NMR**-spectrum (400 MHz, CDCl3) of **34**

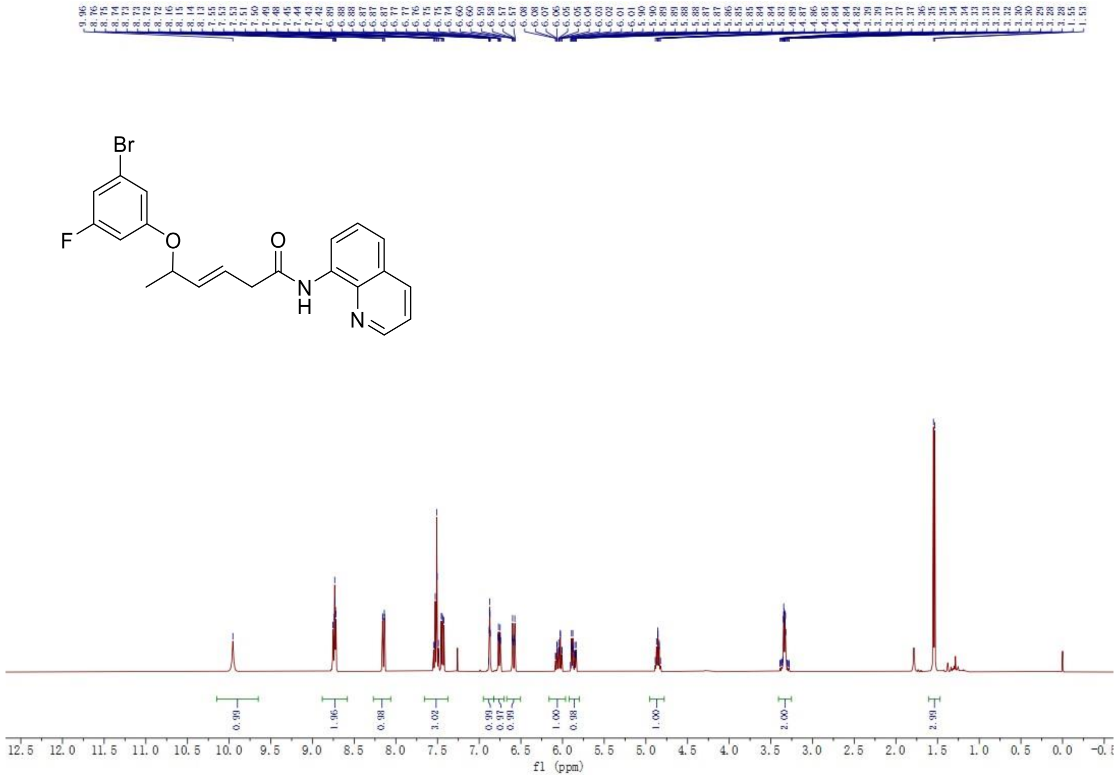

^{13}**C NMR**-spectrum (101 MHz, CDCl3) of **34**

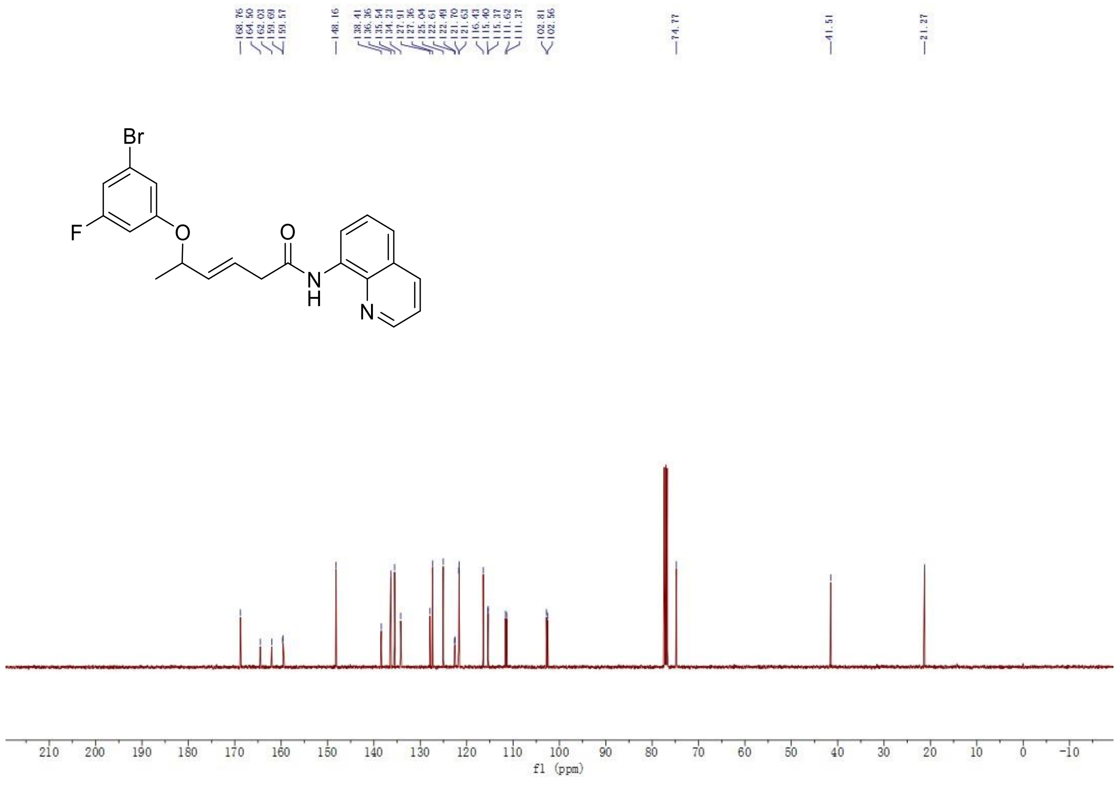

[19]**F NMR**-spectrum (376 MHz, $CDCl_3$) of **34**

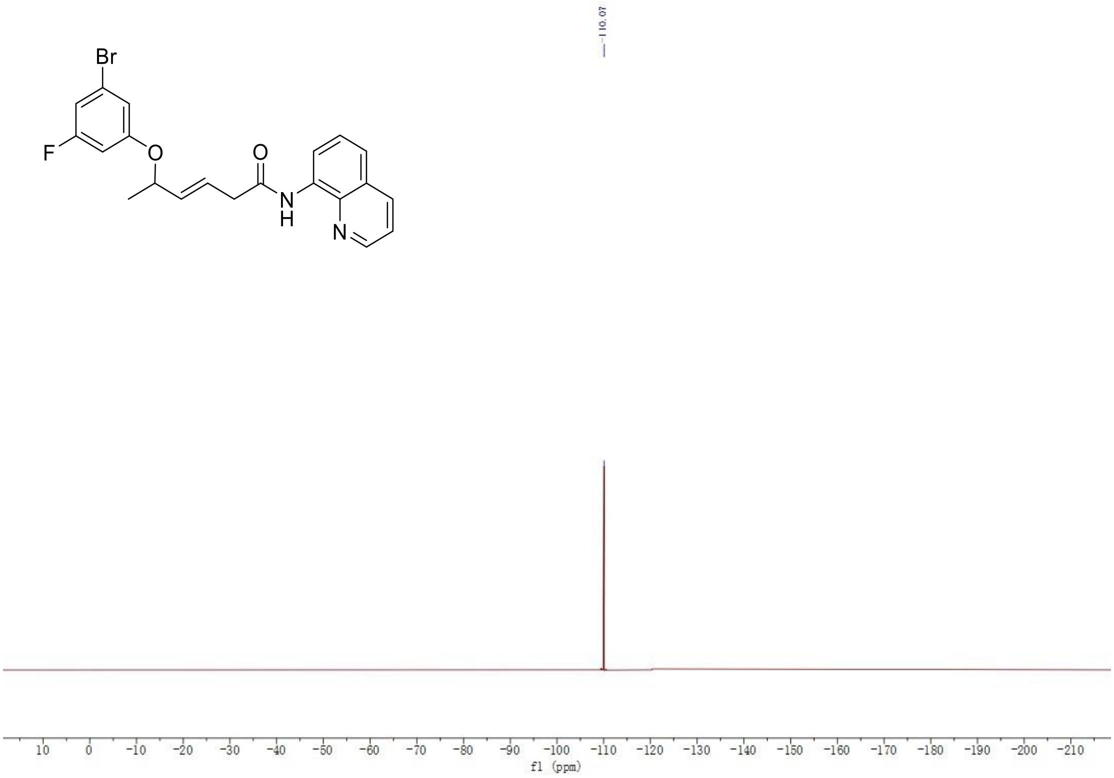

[1]**H NMR**-spectrum (400 MHz, $CDCl_3$) of **35**

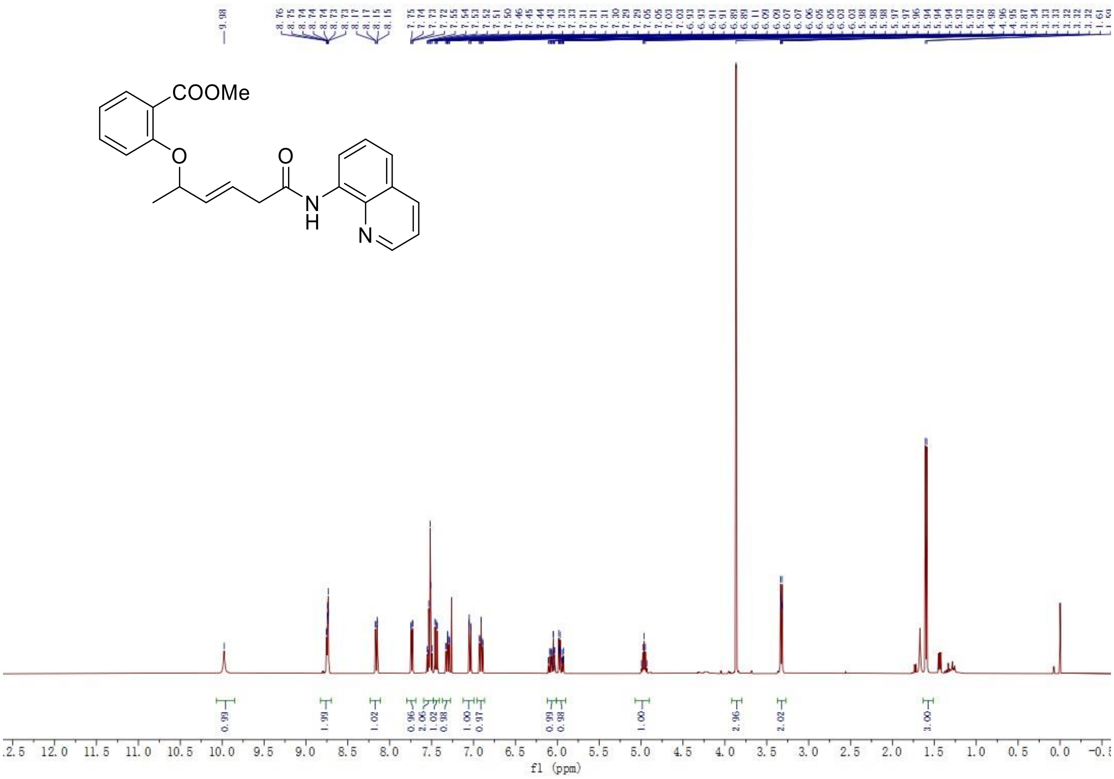

^{13}C NMR-spectrum (101 MHz, CDCl3) of **35**

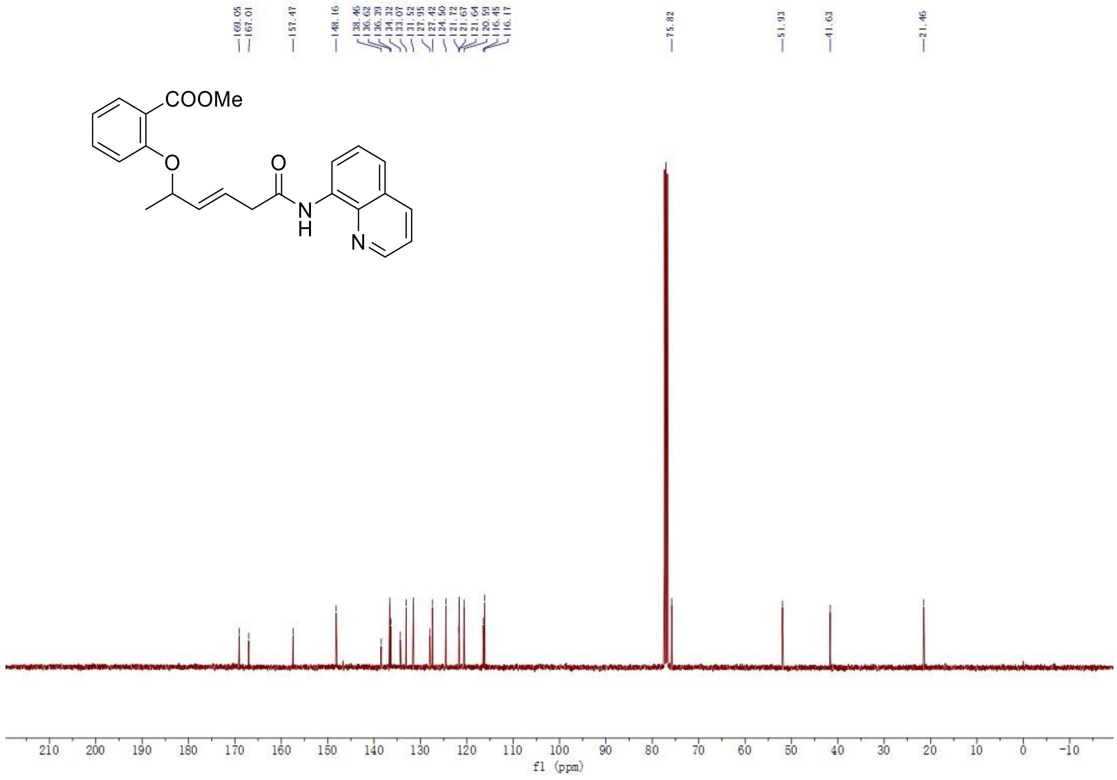

^{1}H NMR-spectrum (400 MHz, CDCl3) of **36**

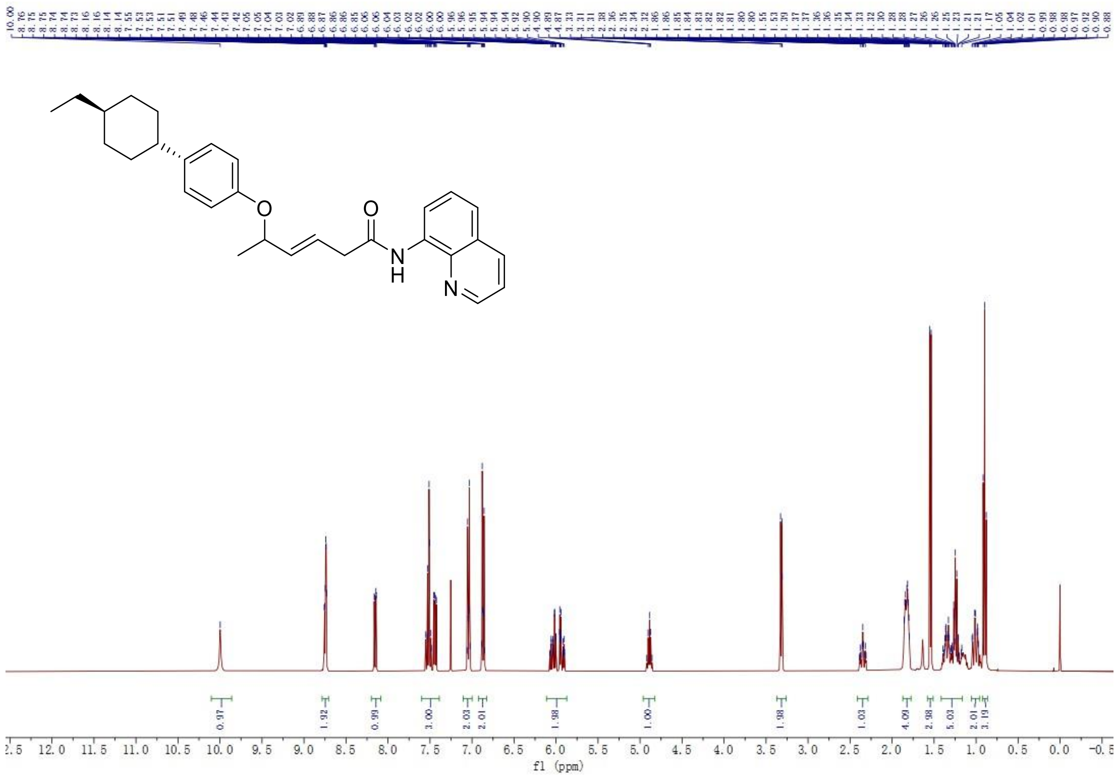

^{13}**C NMR**-spectrum (101 MHz, CDCl3) of **36**

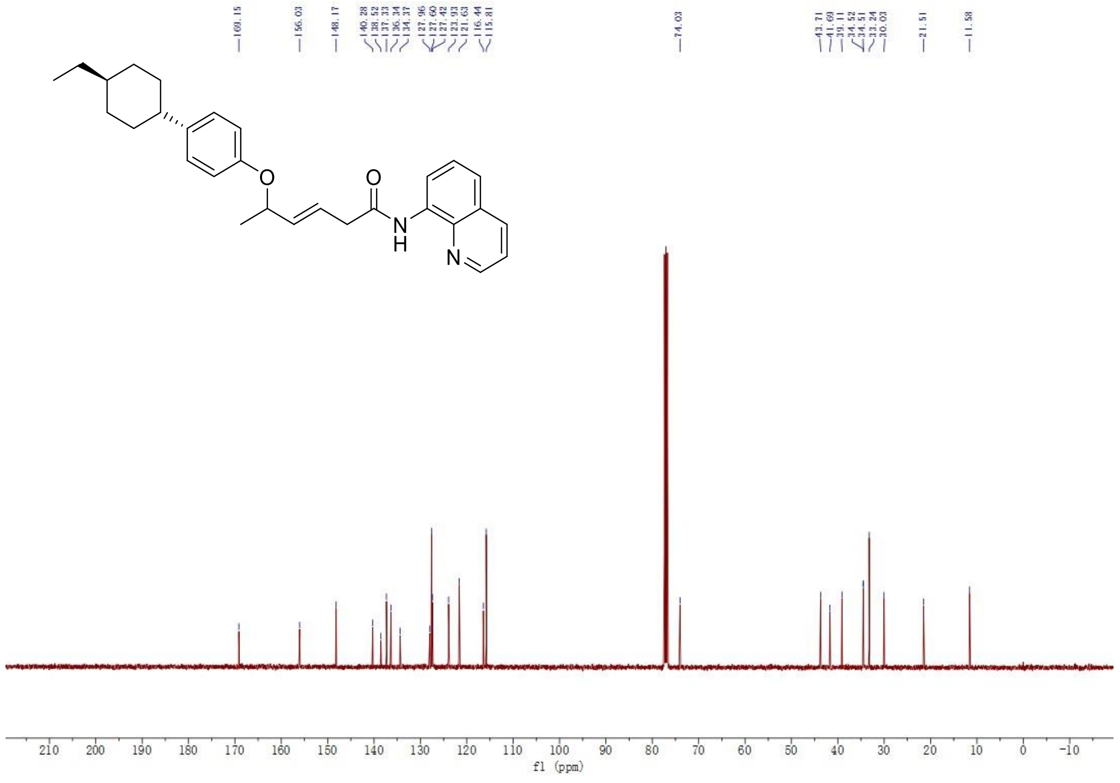

^{1}**H NMR**-spectrum (400 MHz, CDCl3) of **37**

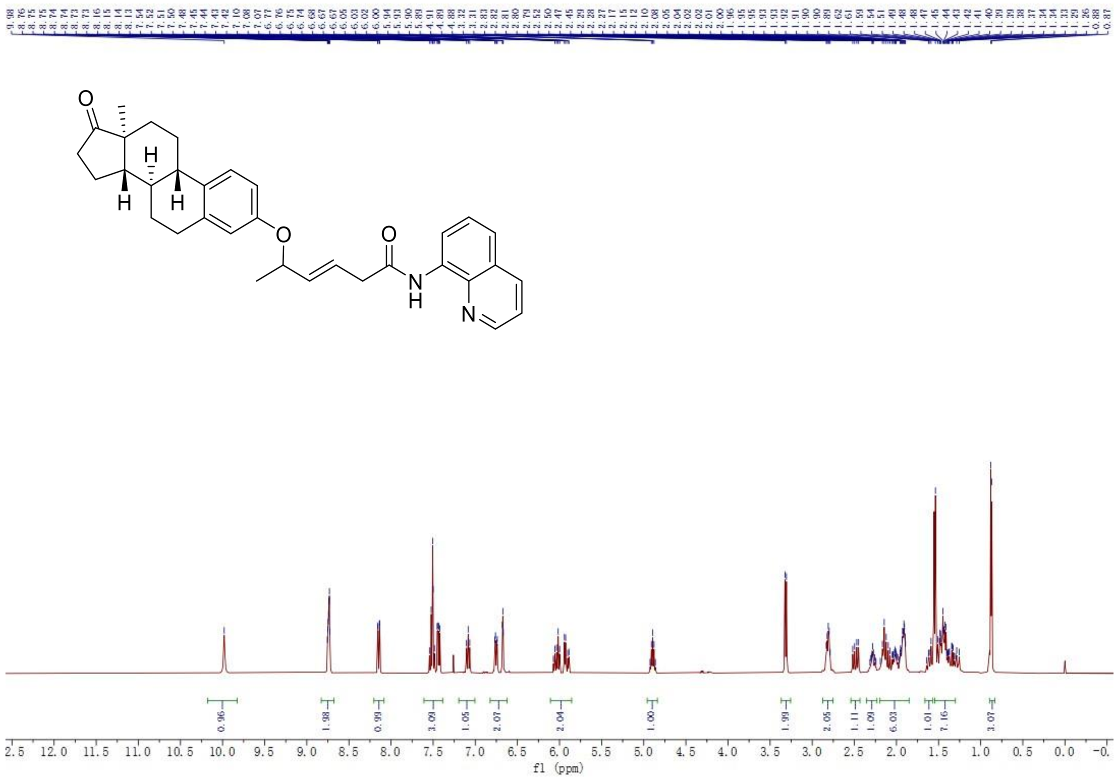

[13]**C NMR**-spectrum (101 MHz, $CDCl_3$) of **37**

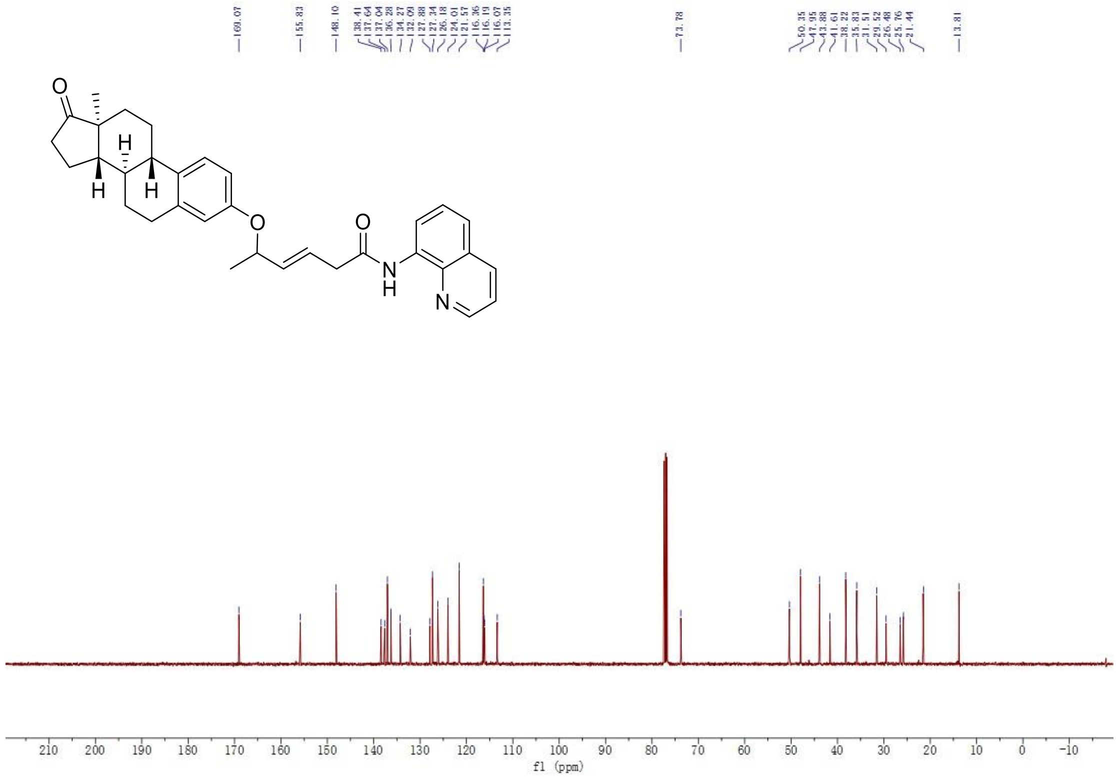

[1]**H NMR**-spectrum (400 MHz, $CDCl_3$) of **38**

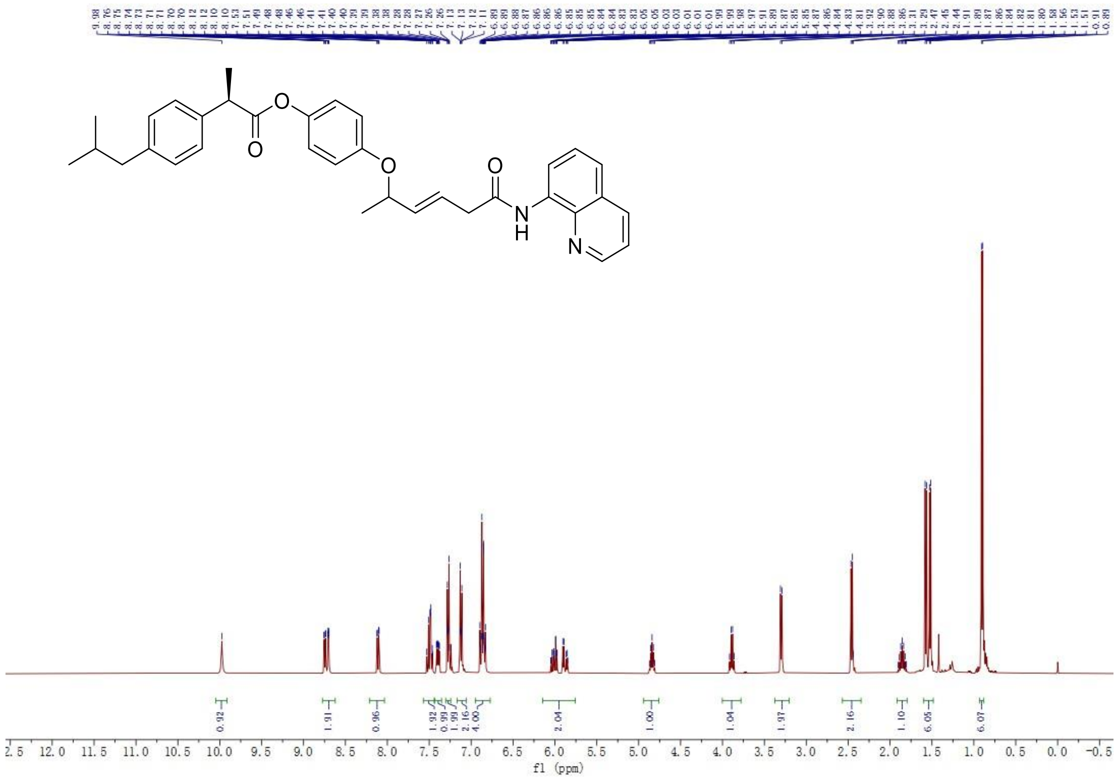

^{13}C NMR-spectrum (101 MHz, $CDCl_3$) of **38**

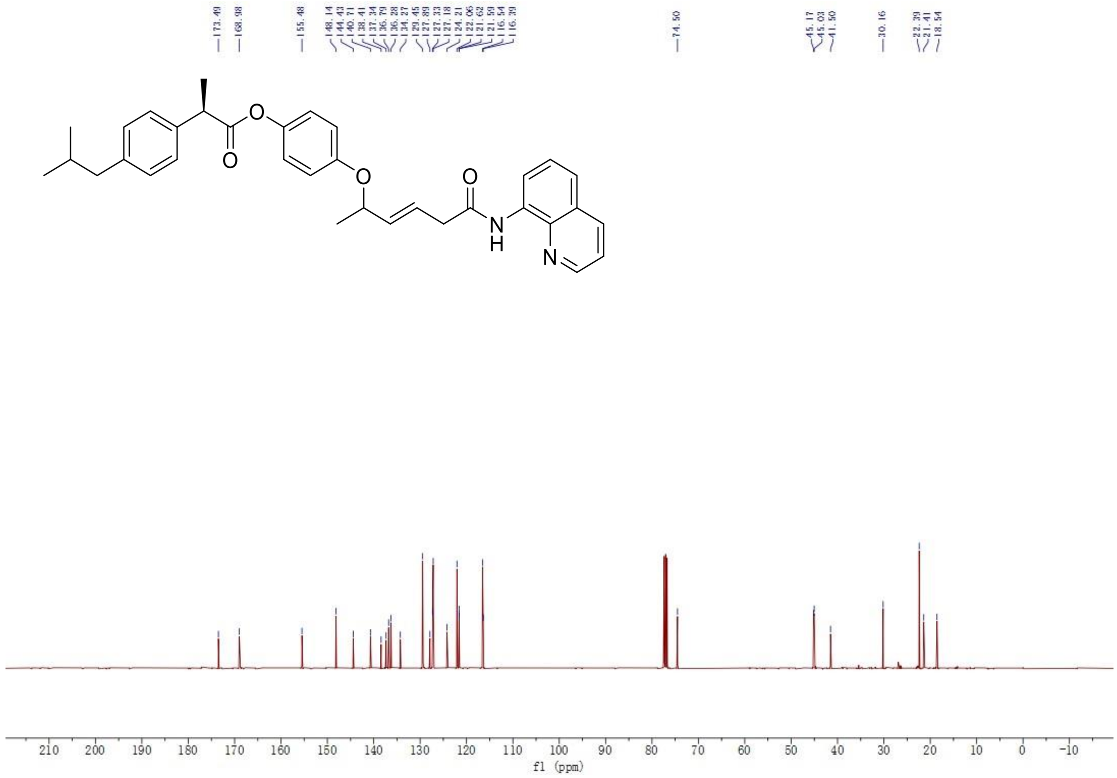

^{1}H NMR-spectrum (400 MHz, $CDCl_3$) of **39**

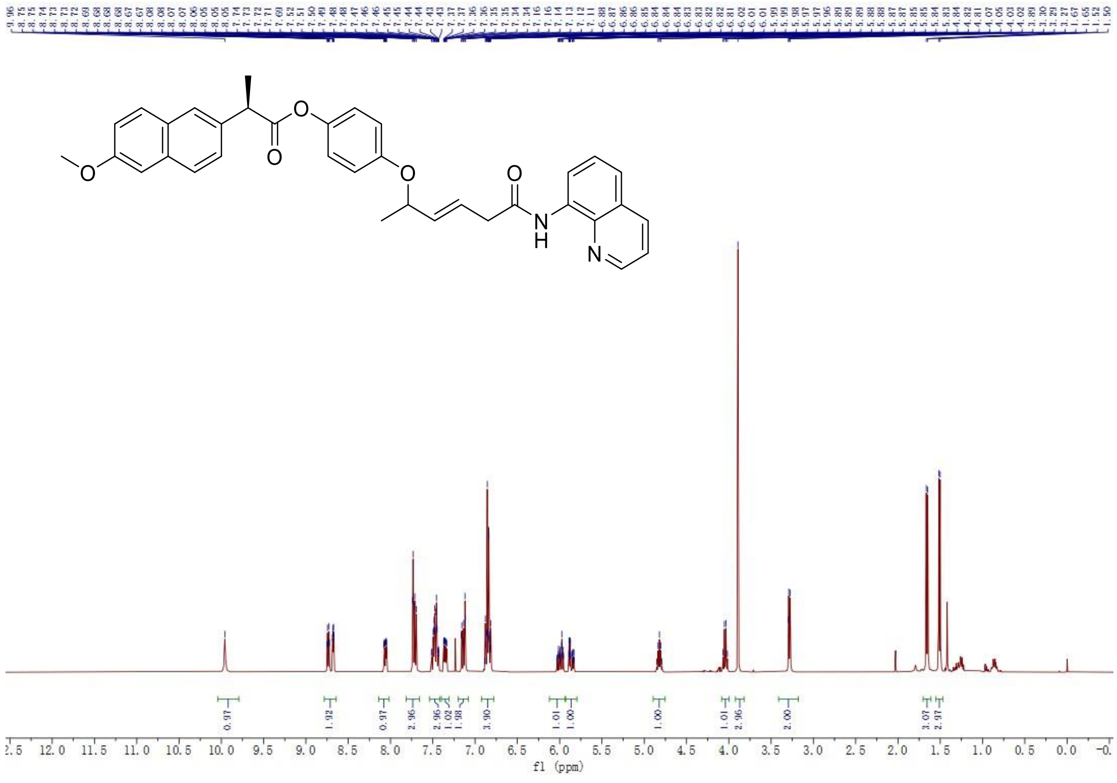

[13] **C NMR**-spectrum (101 MHz, $CDCl_3$) of **39**

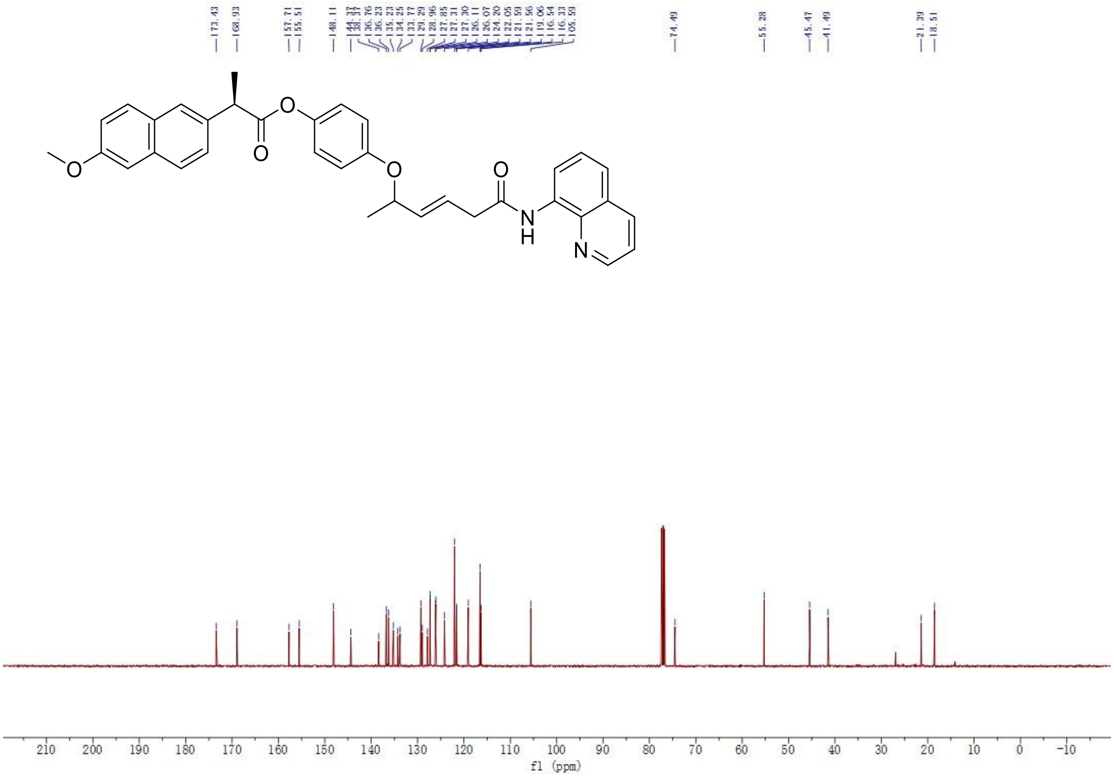

[1] **H NMR**-spectrum (400 MHz, $CDCl_3$) of **40**

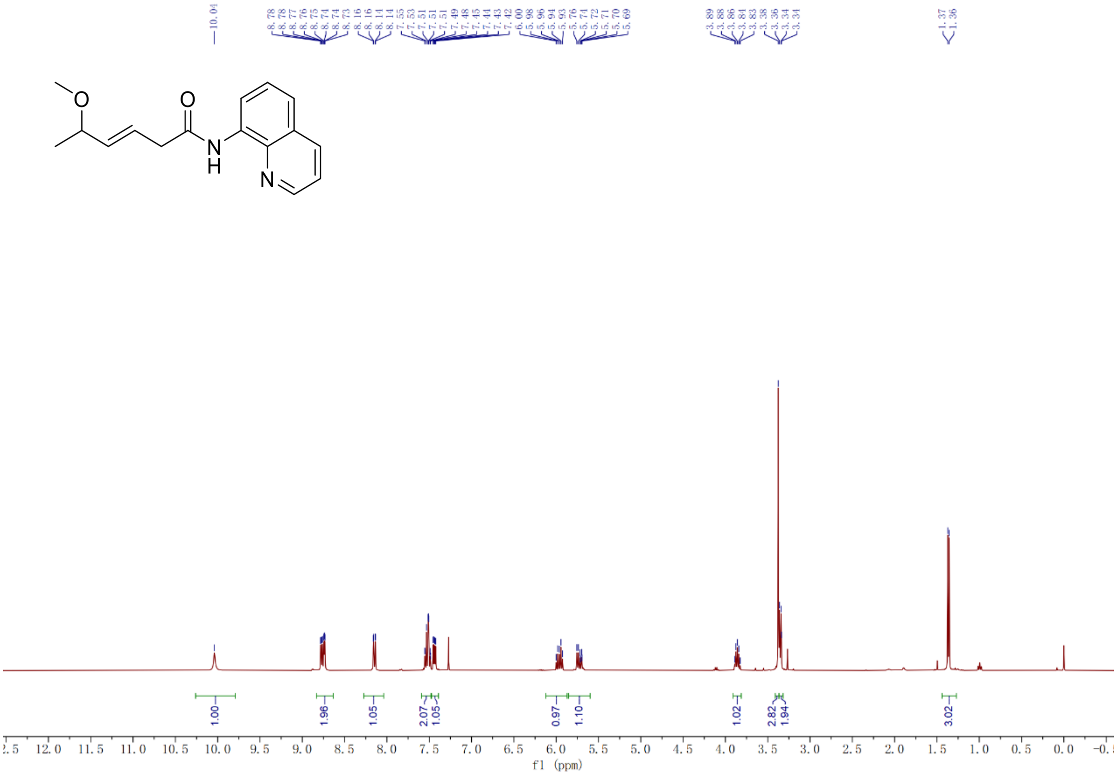

[13] **C NMR**-spectrum (101 MHz, CDCl3) of **40**

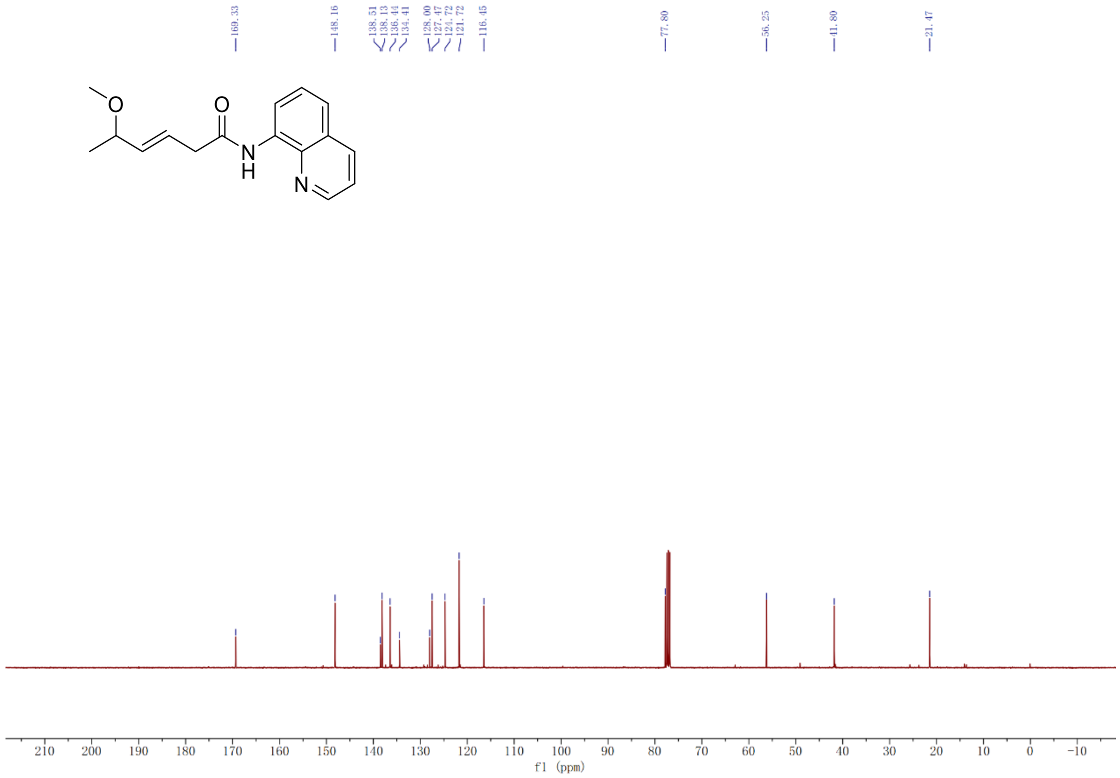

[1] **H NMR**-spectrum (400 MHz, CDCl3) of **41**

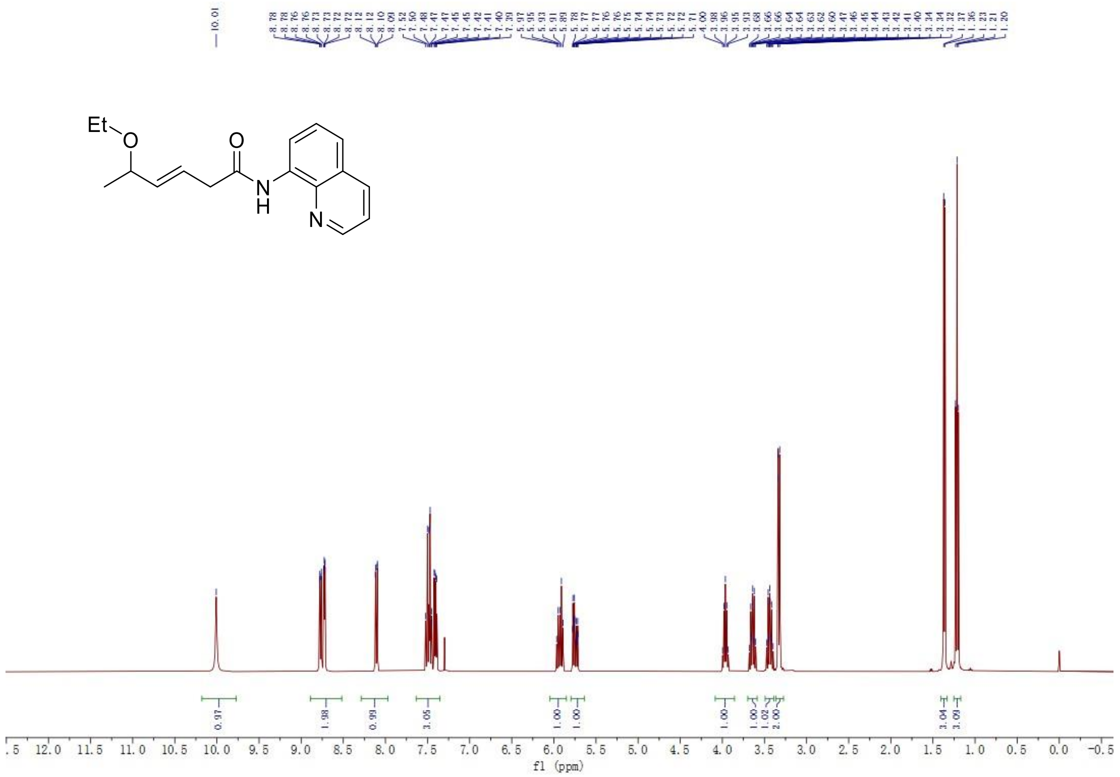

^{13}C NMR-spectrum (101 MHz, CDCl3) of **41**

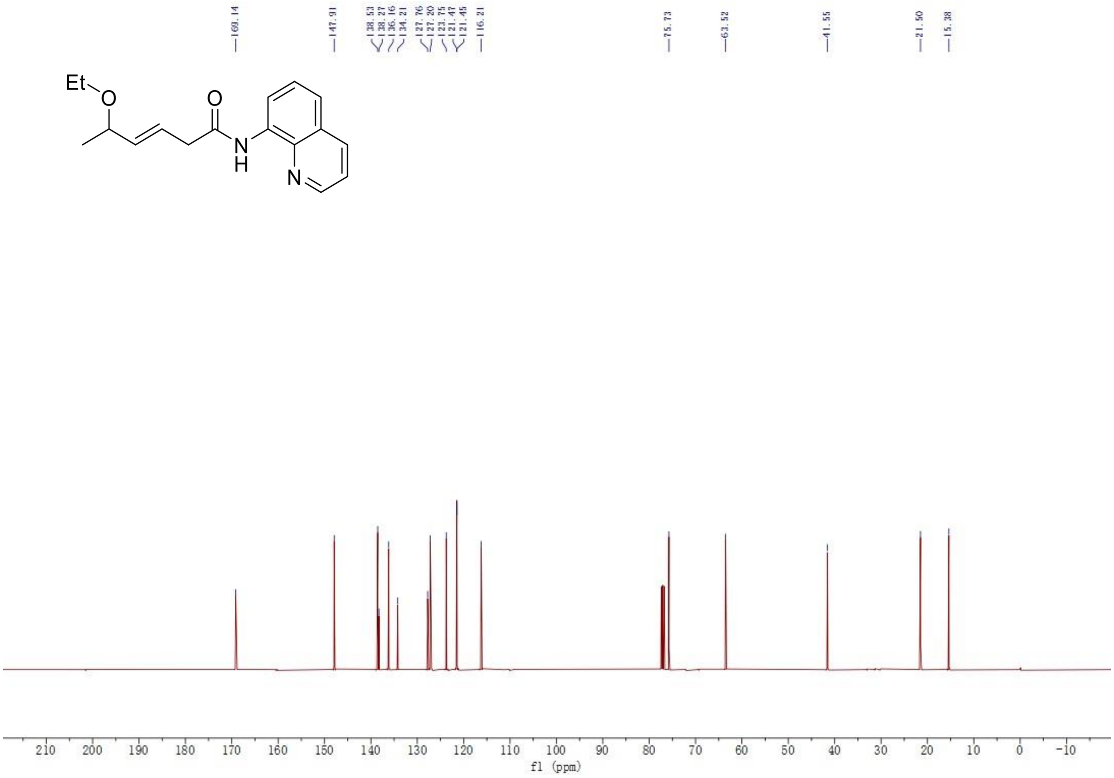

^{1}H NMR-spectrum (400 MHz, CDCl3) of **42**

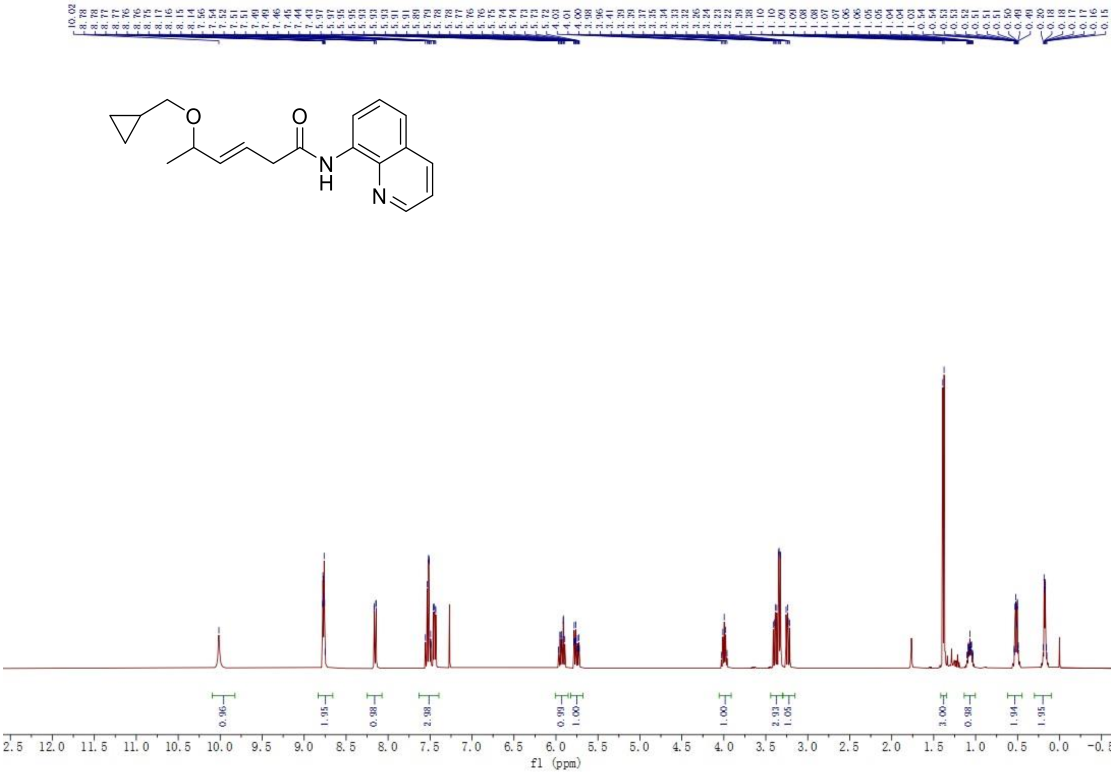

[13]**C NMR**-spectrum (101 MHz, $CDCl_3$) of **42**

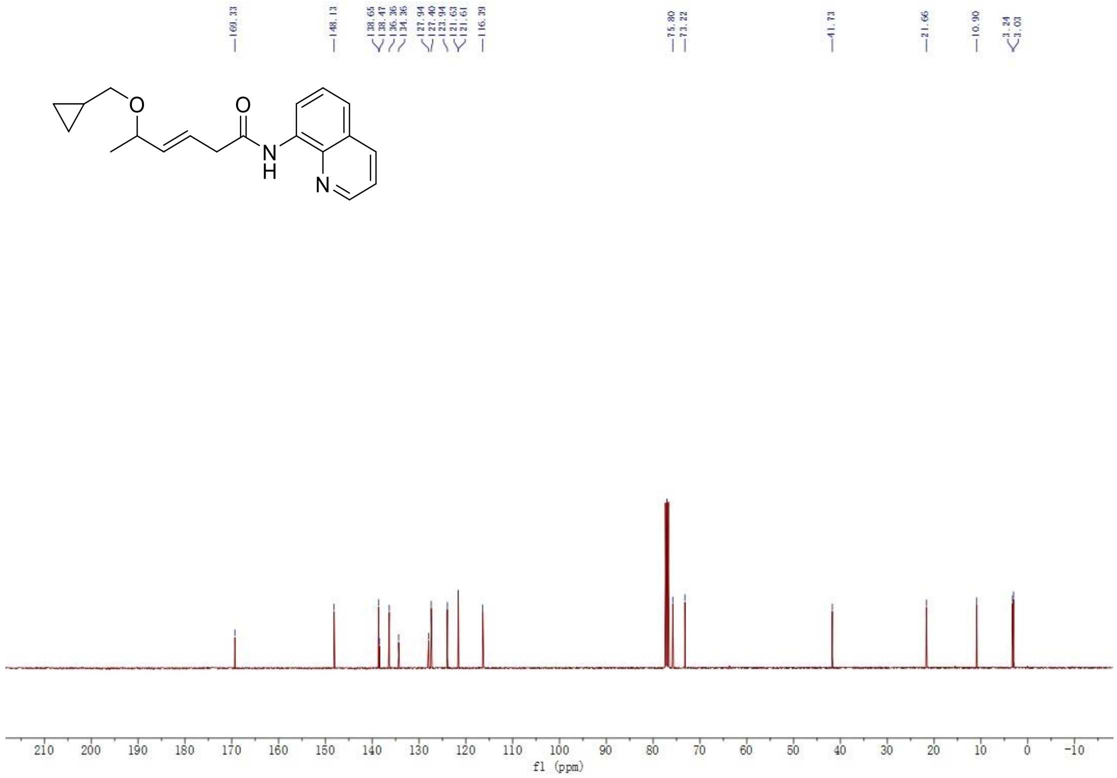

[1]**H NMR**-spectrum (400 MHz, $CDCl_3$) of **43**

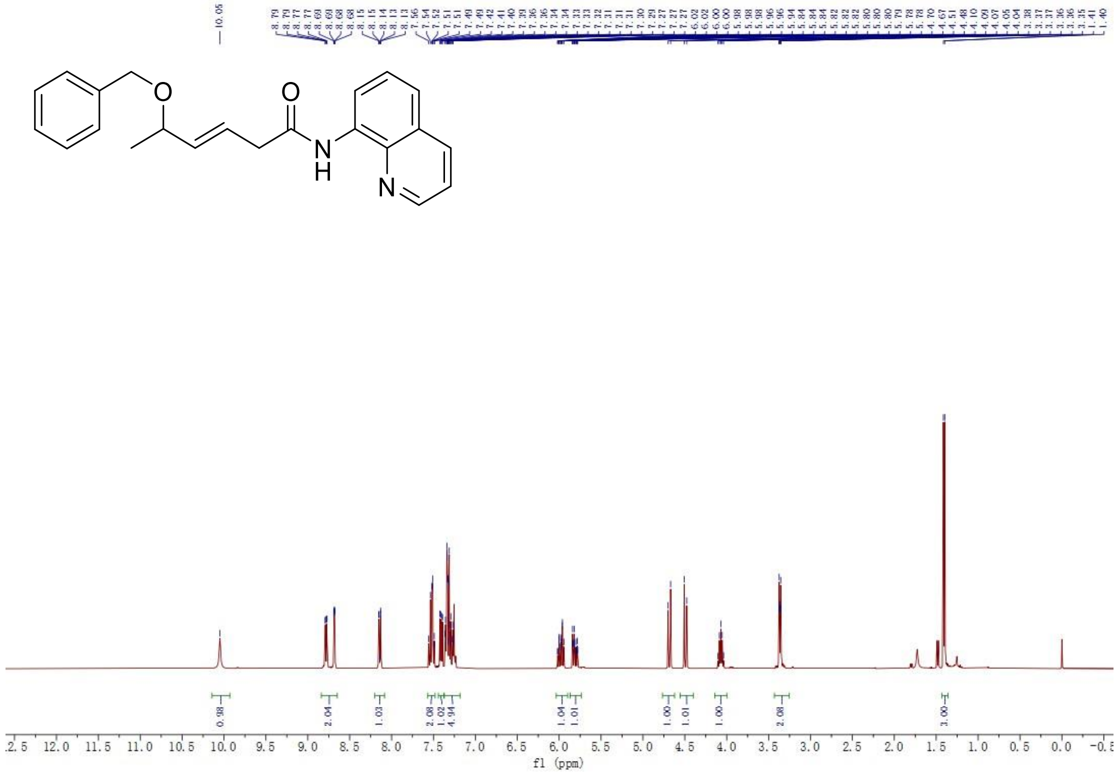

[13]**C NMR**-spectrum (101 MHz, $CDCl_3$) of **43**

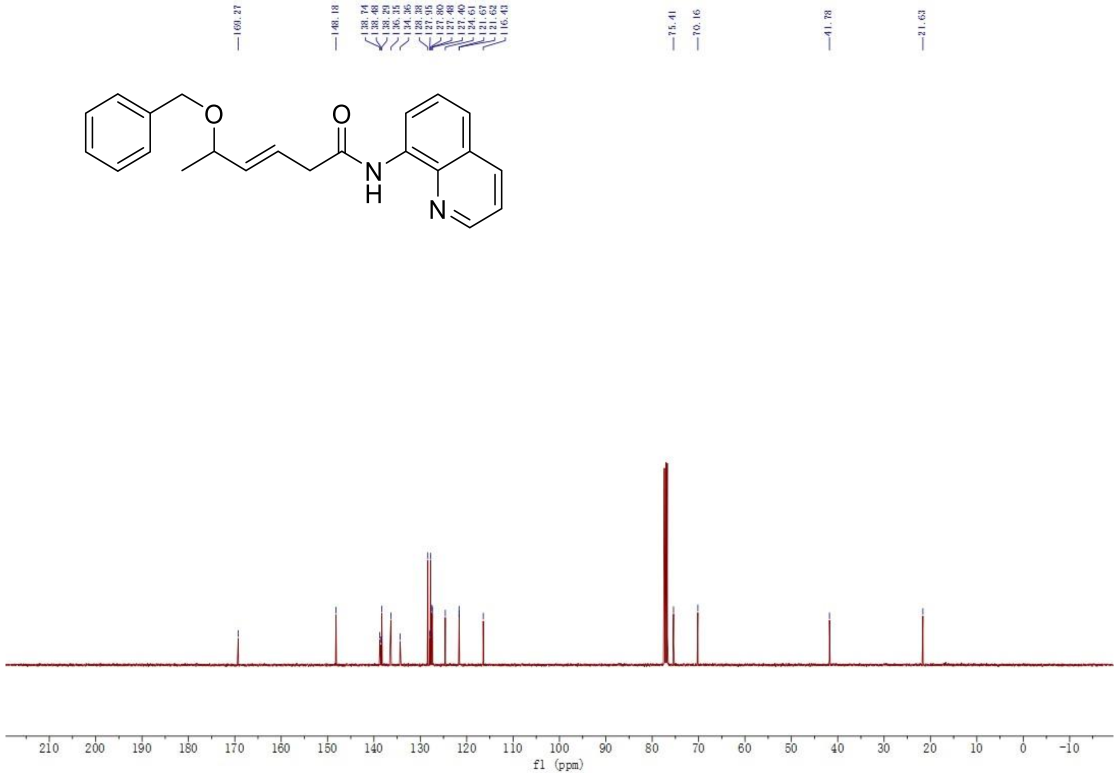

[1]**H NMR**-spectrum (400 MHz, $CDCl_3$) of **44**

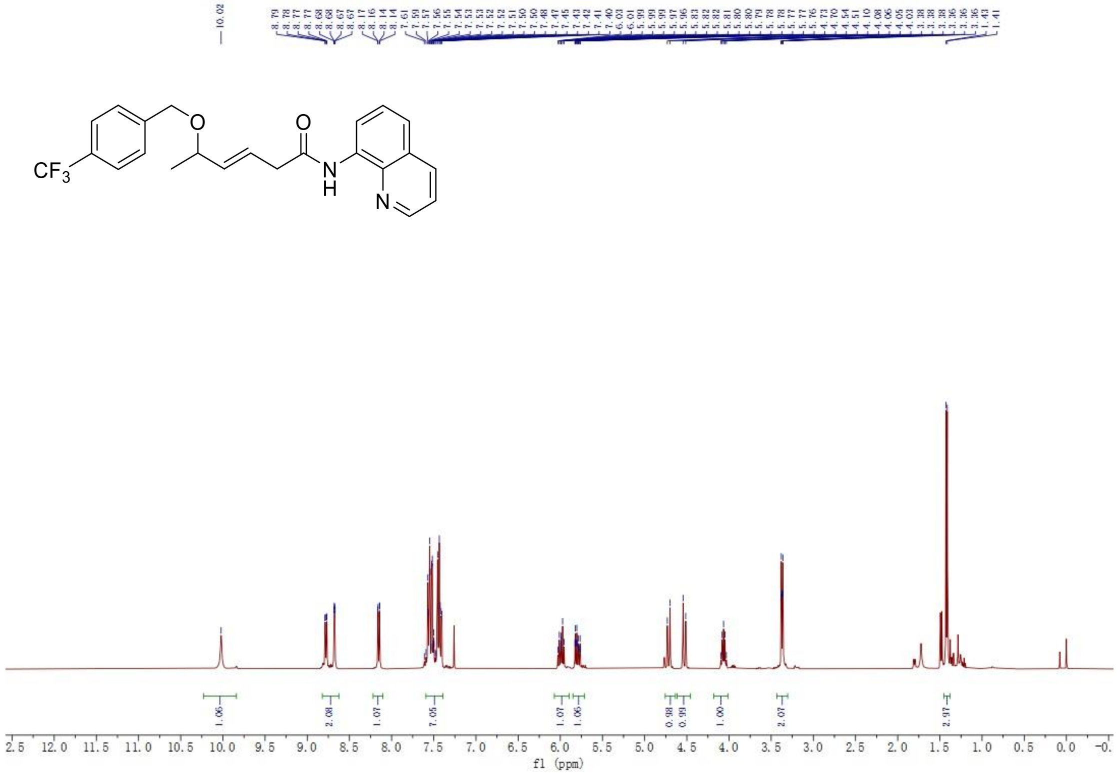

^{13}C **NMR**-spectrum (101 MHz, $CDCl_3$) of **44**

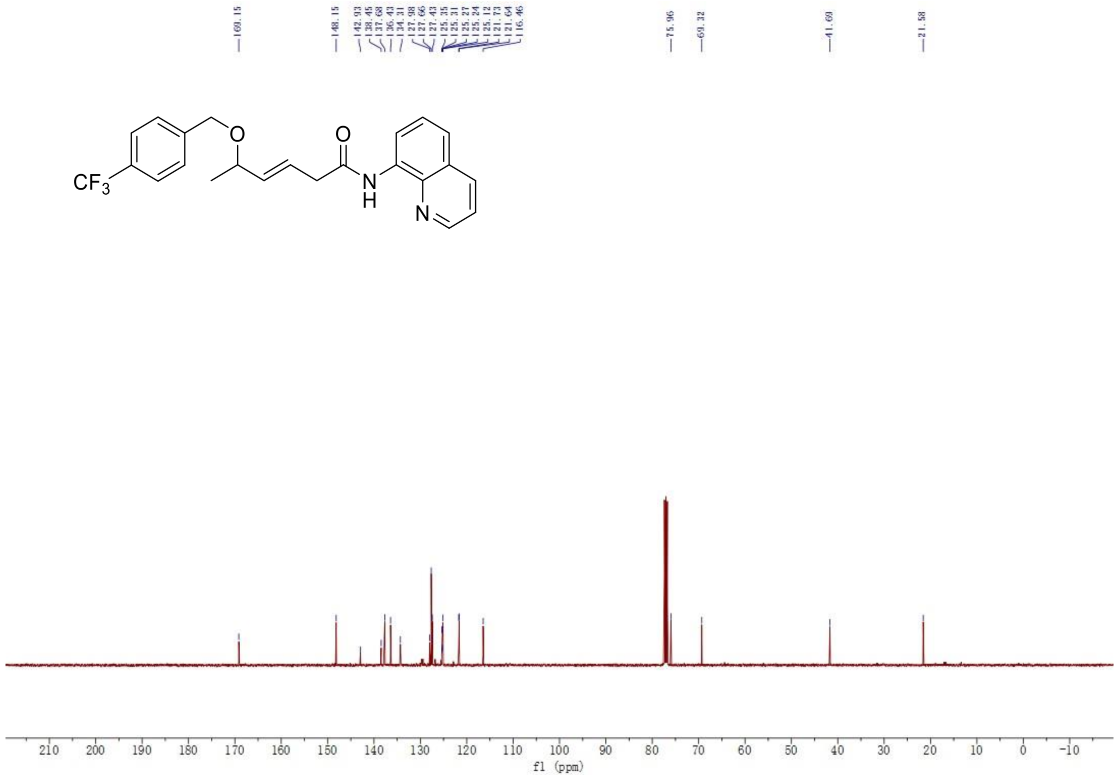

^{19}F **NMR**-spectrum (376 MHz, $CDCl_3$) of **44**

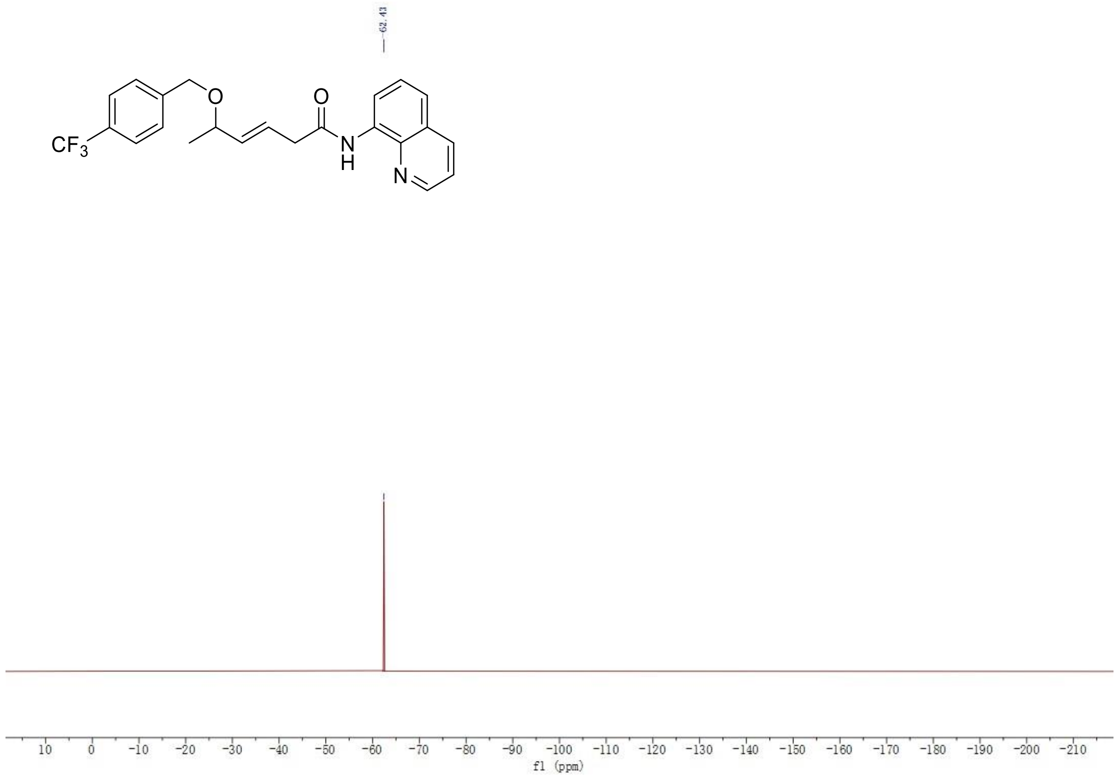

[1]**H NMR**-spectrum (400 MHz, CDCl3) of **45**

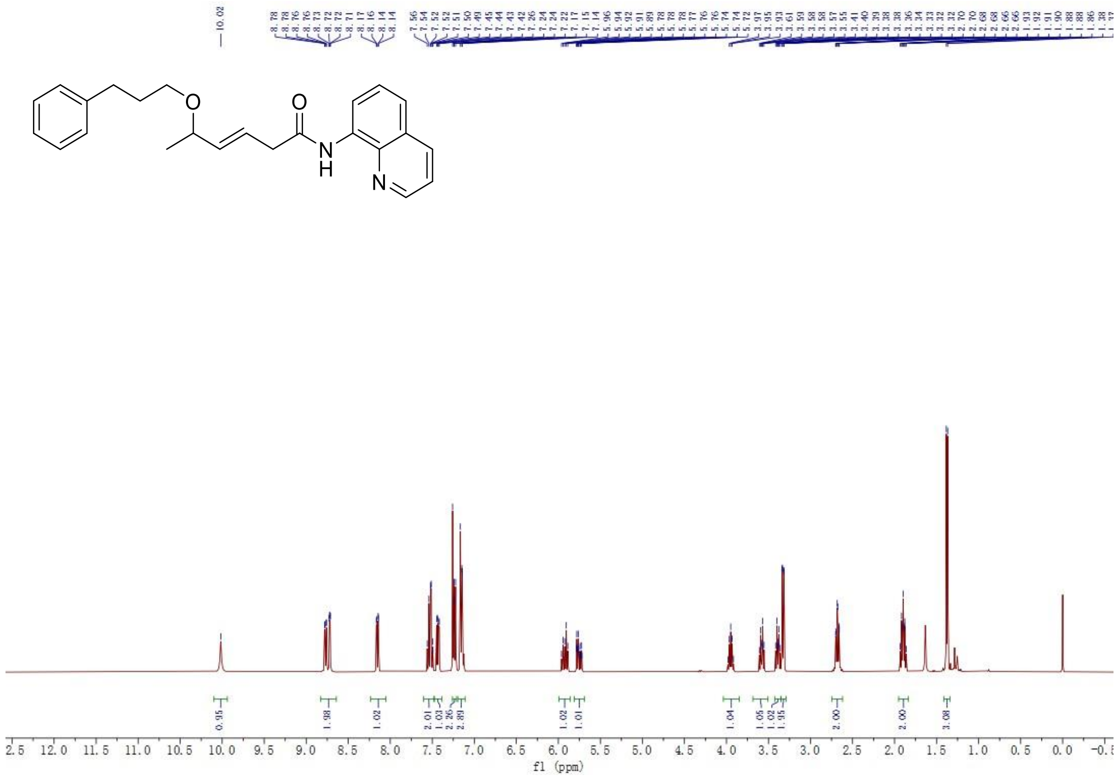

[13]**C NMR**-spectrum (101 MHz, CDCl3) of **45**

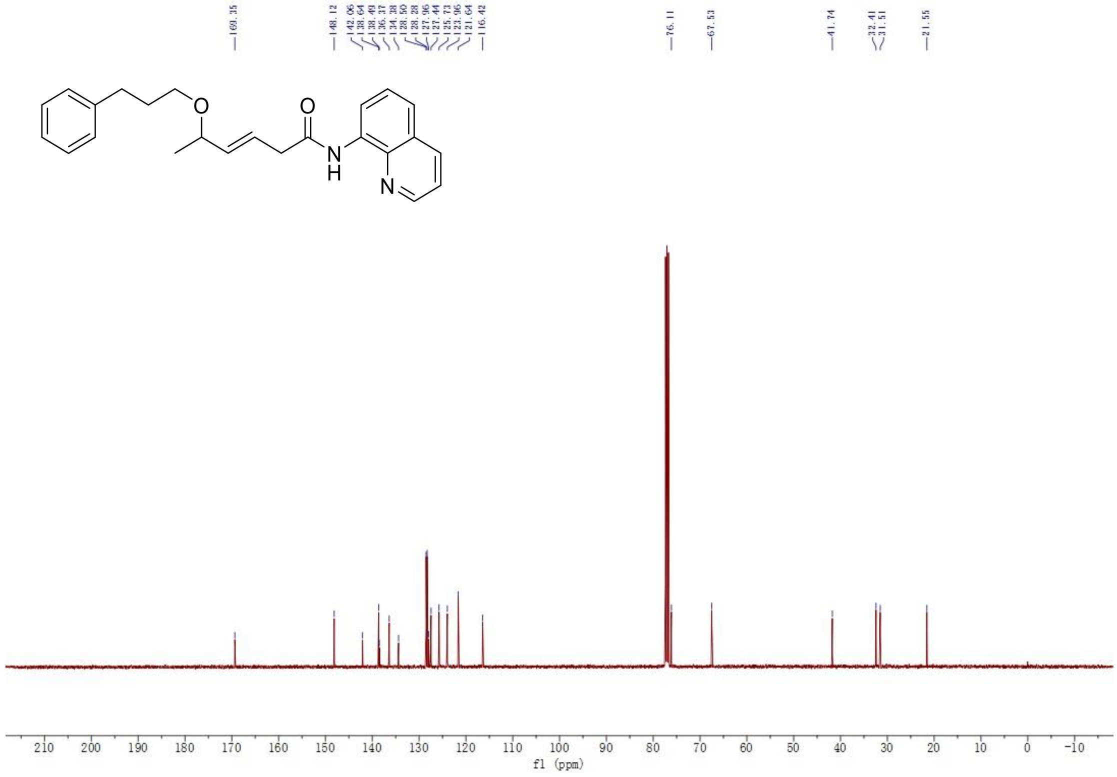

^{1}**H NMR**-spectrum (400 MHz, $CDCl_3$) of **46**

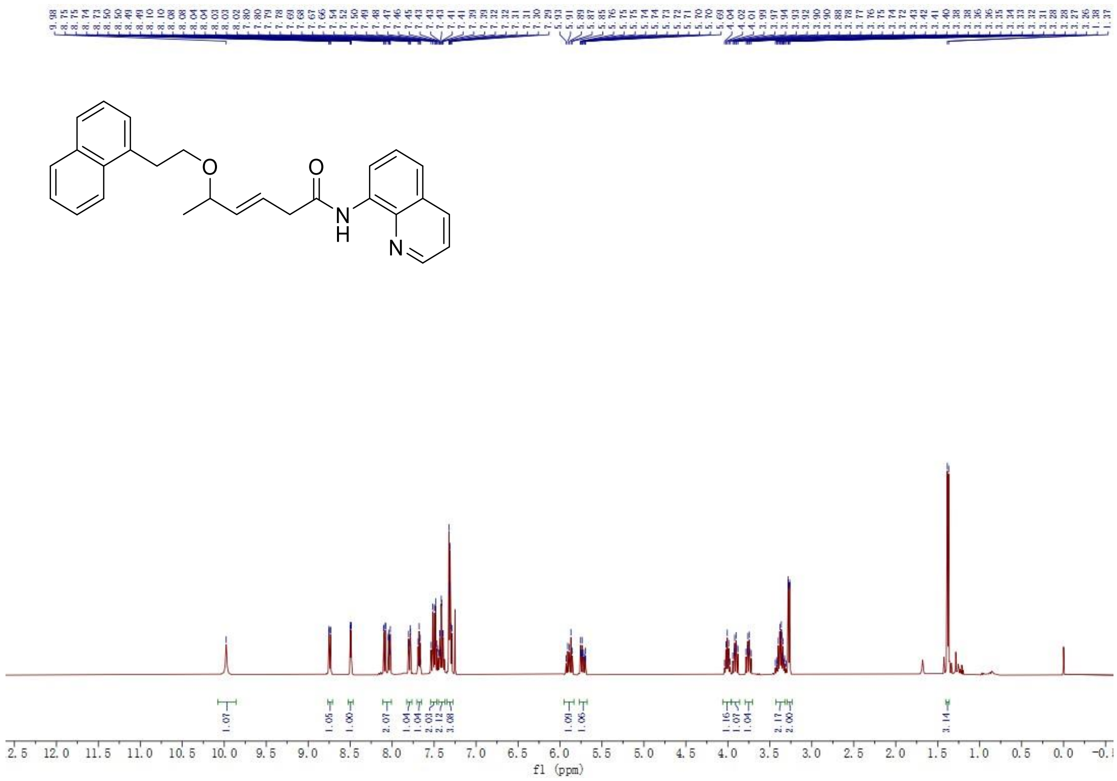

^{13}**C NMR**-spectrum (101 MHz, $CDCl_3$) of **46**

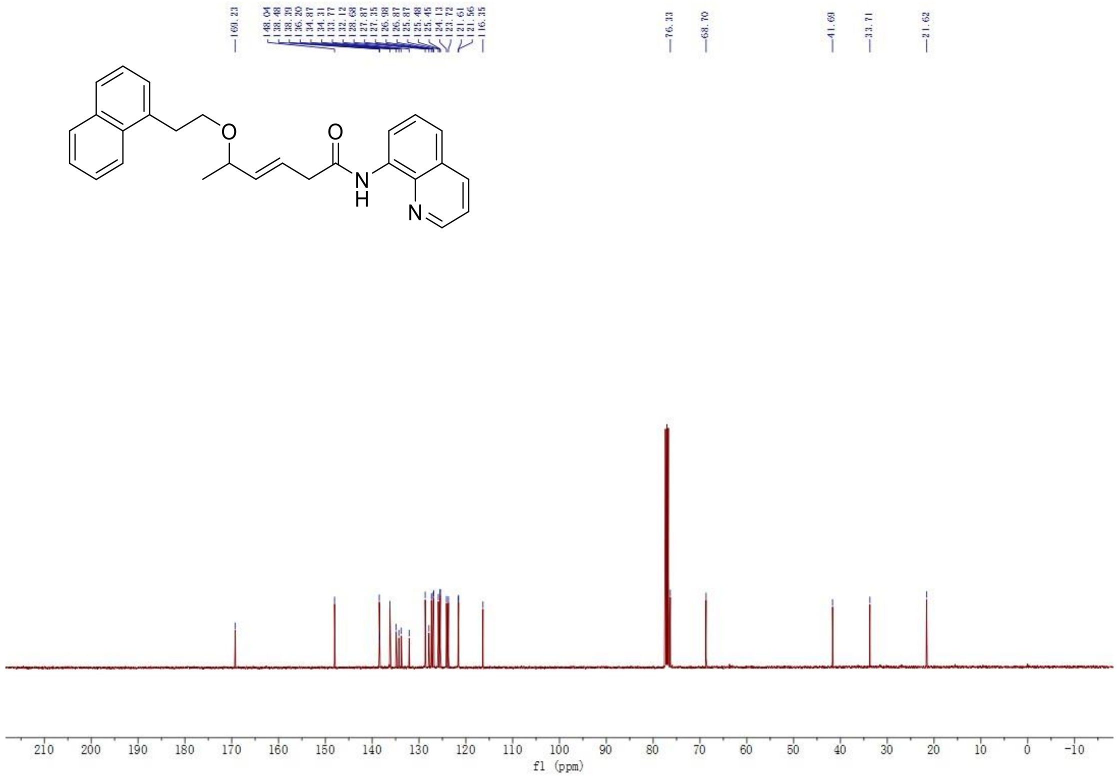

^{1}H NMR-spectrum (400 MHz, CDCl3) of **47**

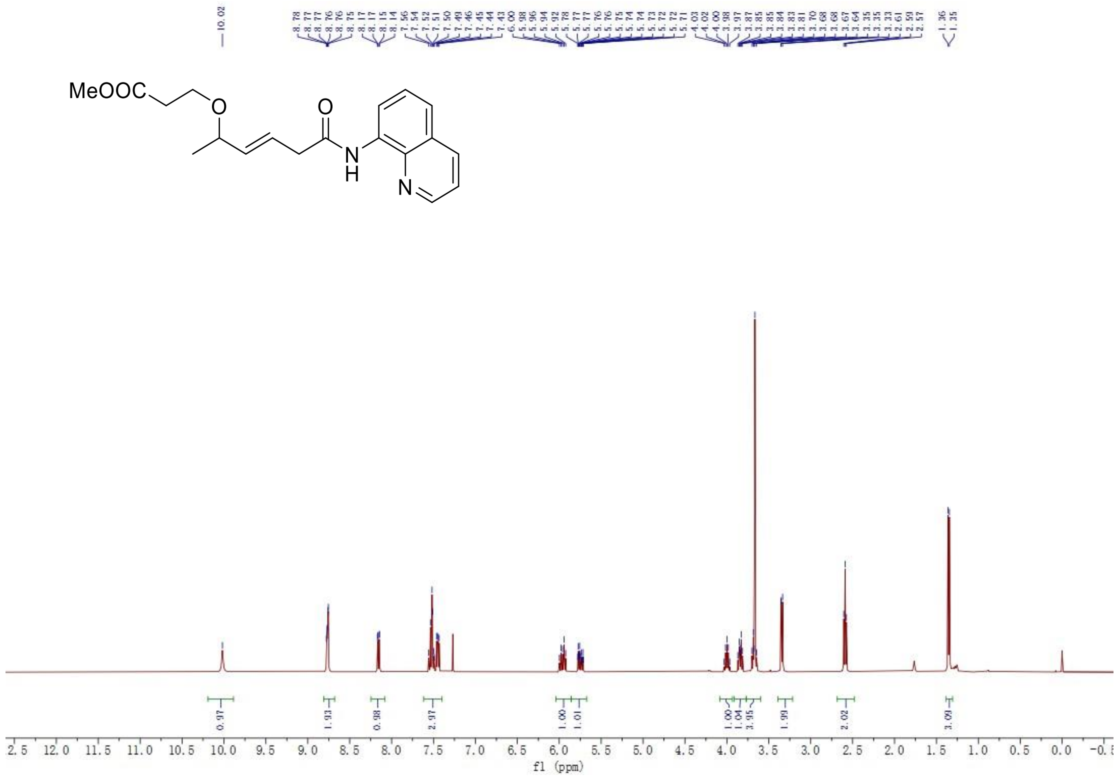

^{13}C NMR-spectrum (101 MHz, CDCl3) of **47**

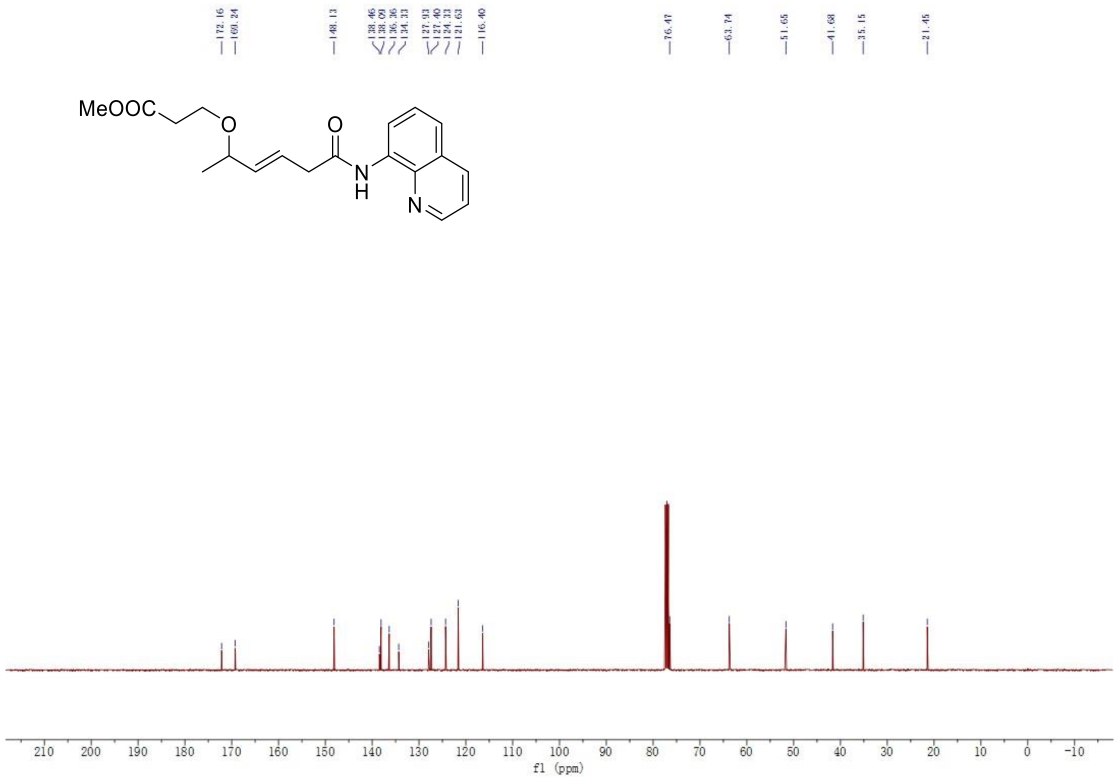

[1]**H NMR**-spectrum (400 MHz, $CDCl_3$) of **48**

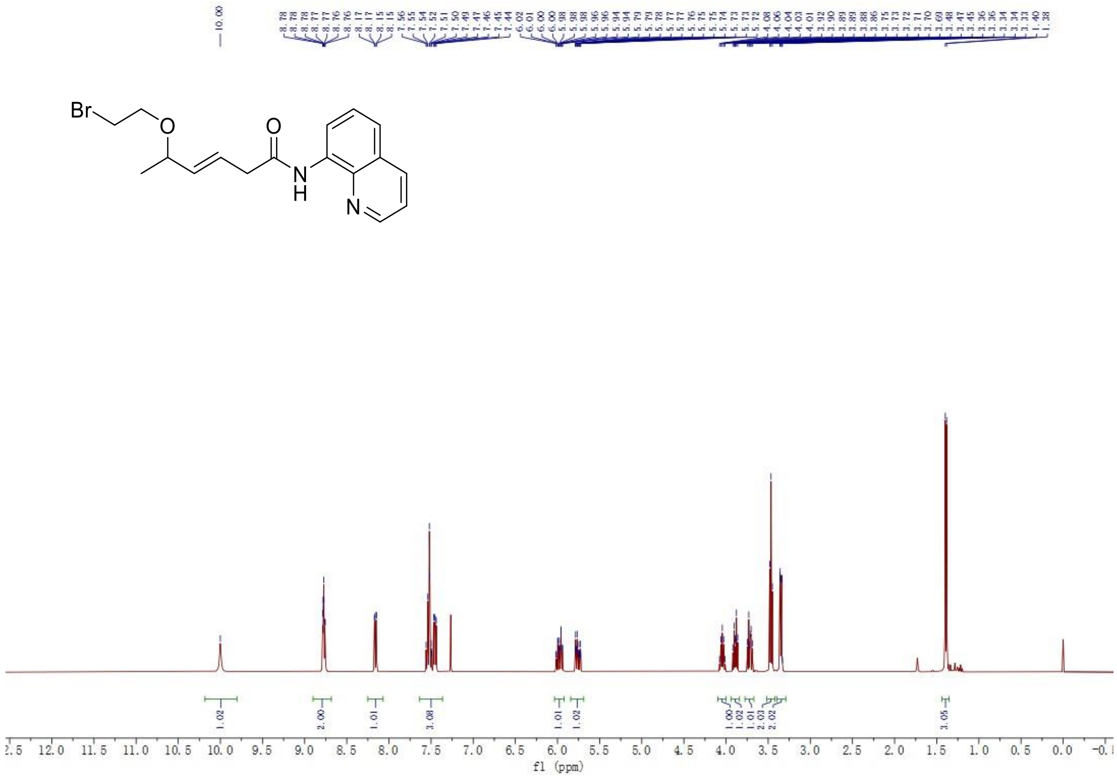

[13]**C NMR**-spectrum (101 MHz, $CDCl_3$) of **48**

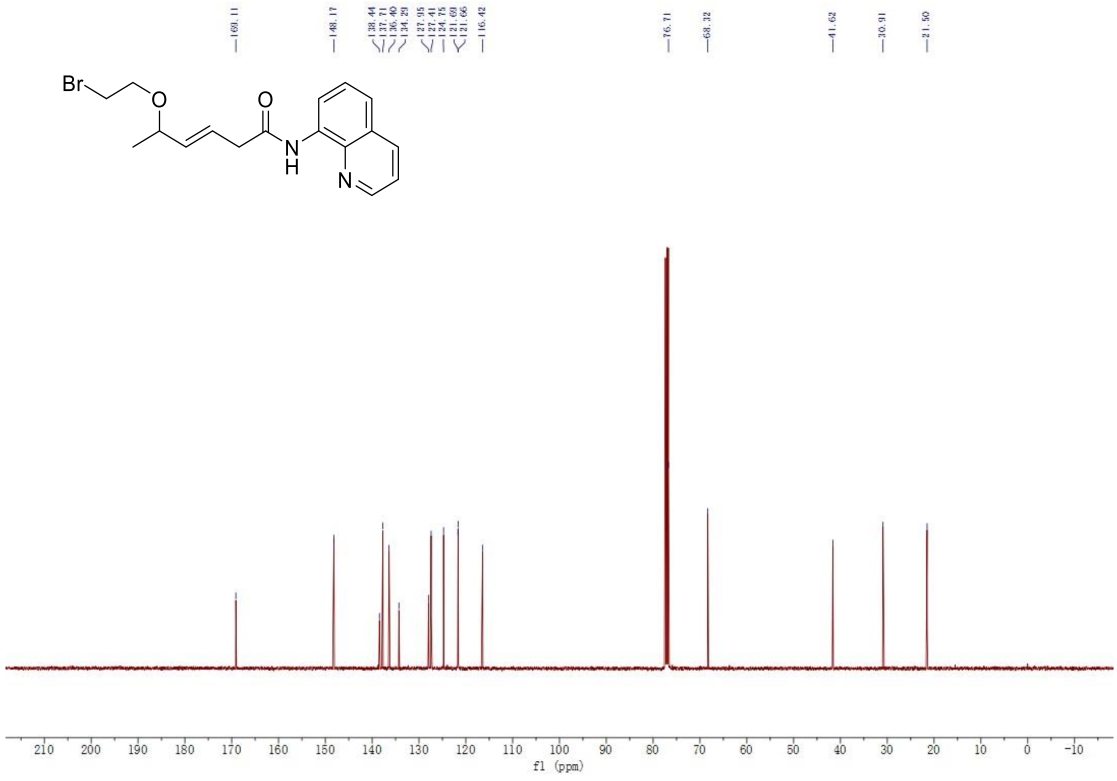

^{1}**H NMR**-spectrum (400 MHz, CDCl$_3$) of **49**

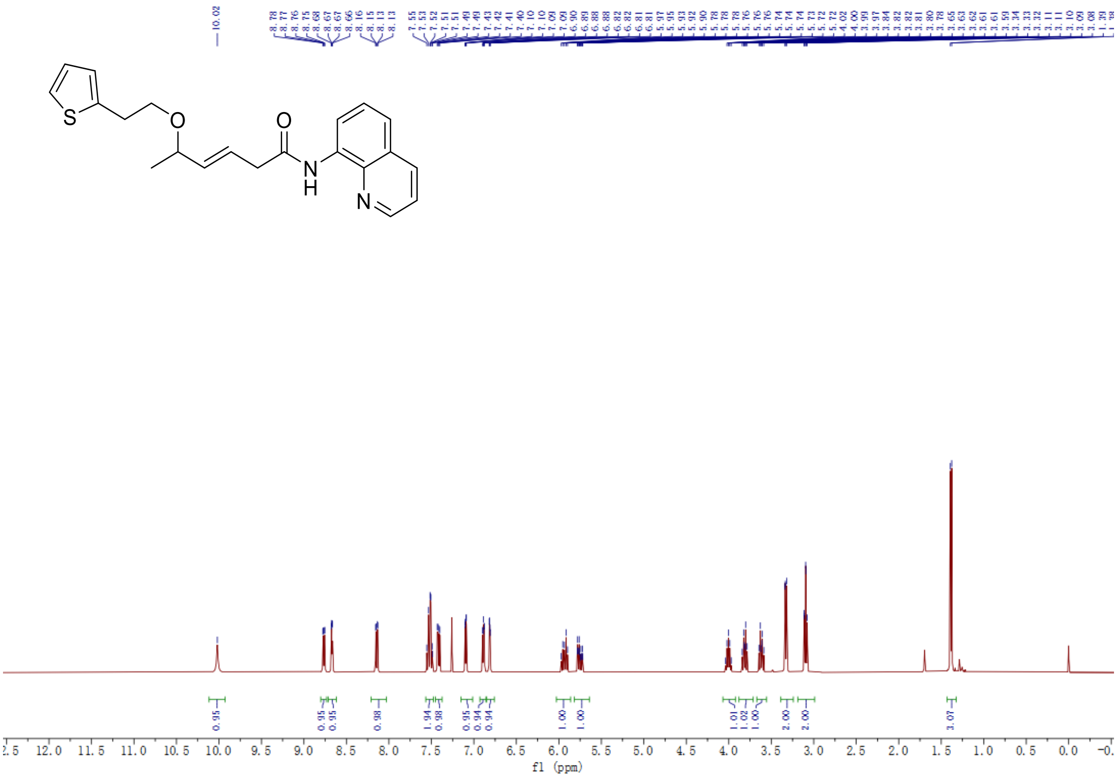

^{13}**C NMR**-spectrum (101 MHz, CDCl$_3$) of **49**

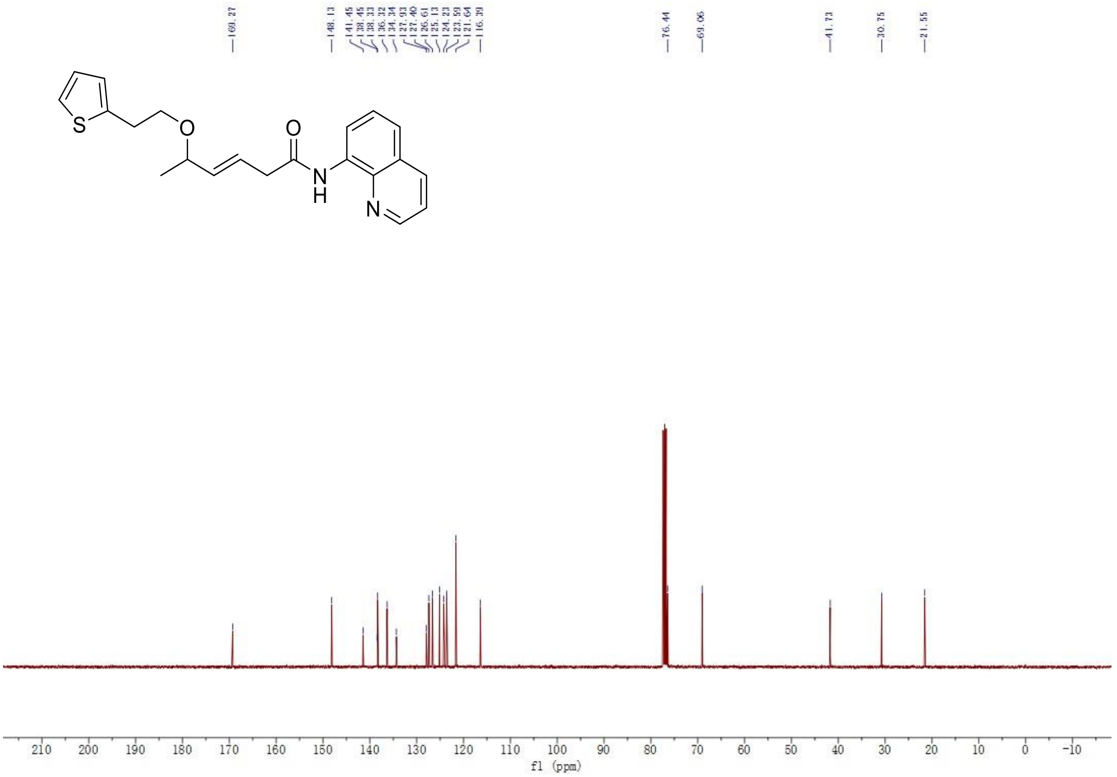

^{1}H **NMR**-spectrum (400 MHz, $CDCl_3$) of **50**

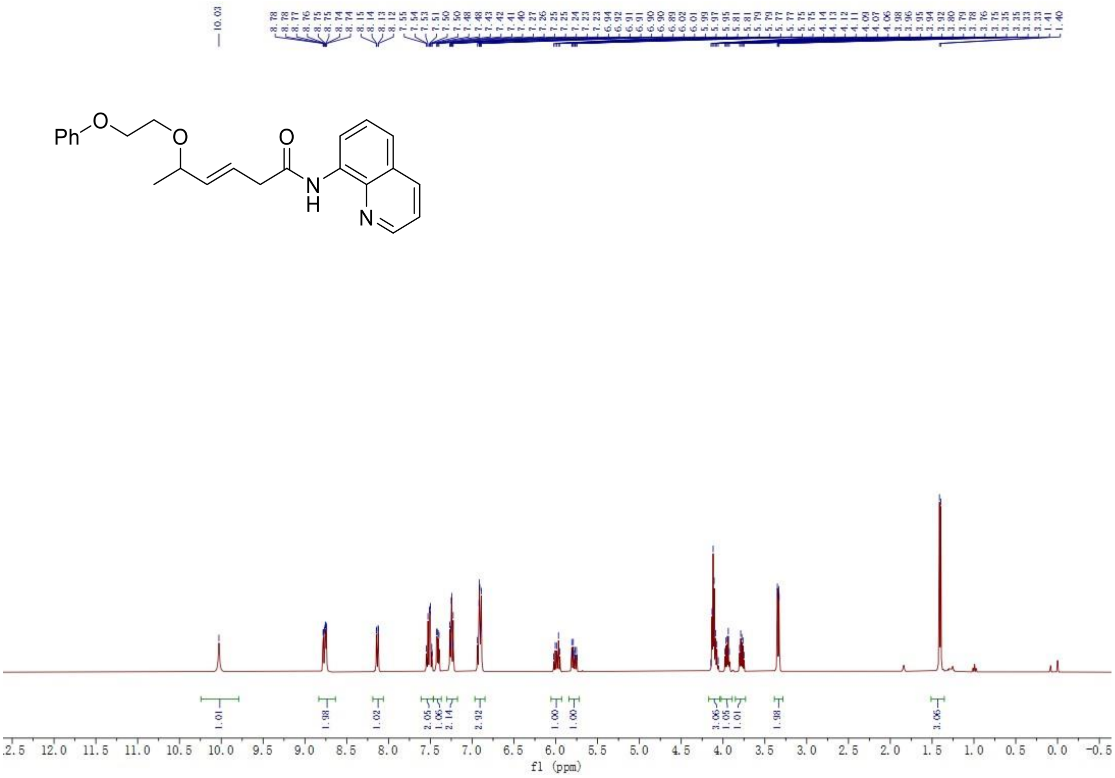

^{13}C **NMR**-spectrum (101 MHz, $CDCl_3$) of **50**

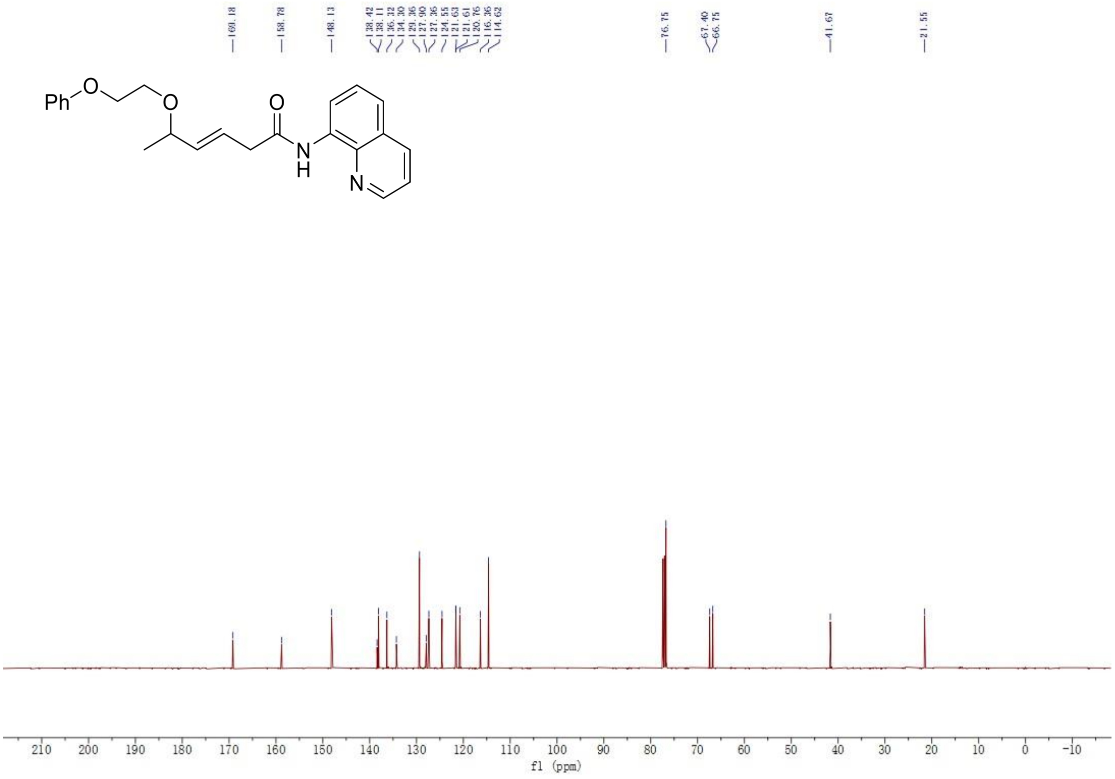

^{1}H NMR-spectrum (400 MHz, $CDCl_3$) of **51**

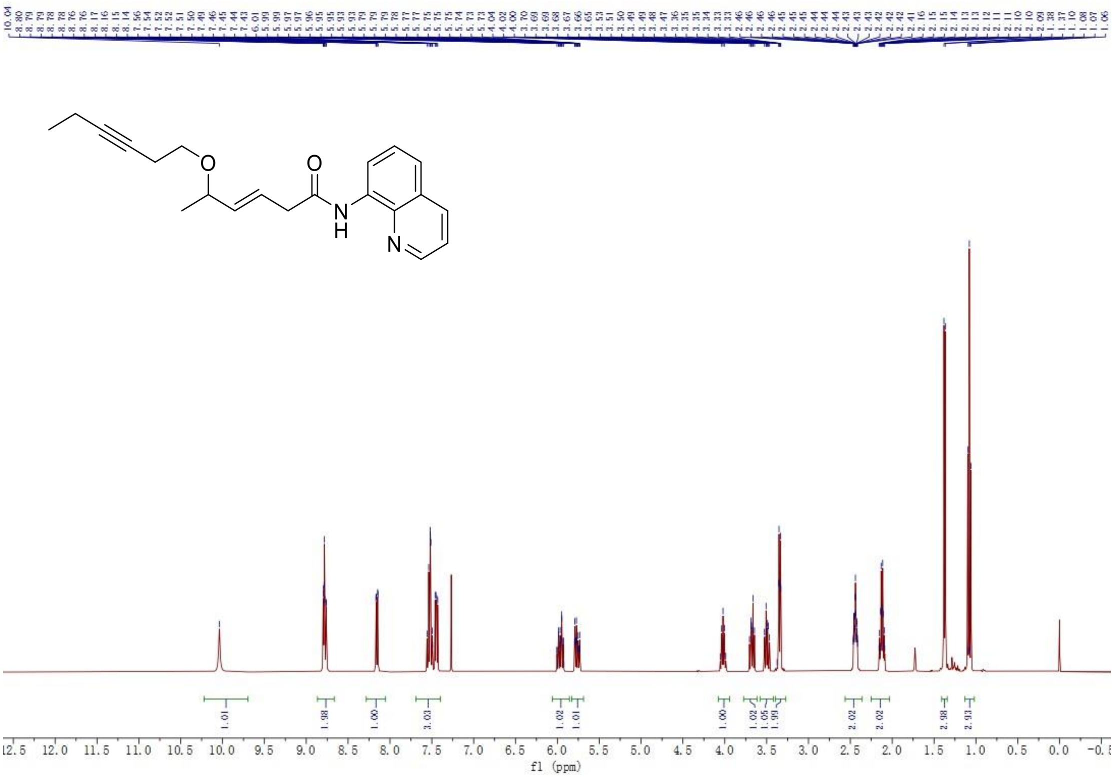

^{13}C NMR-spectrum (101 MHz, $CDCl_3$) of **51**

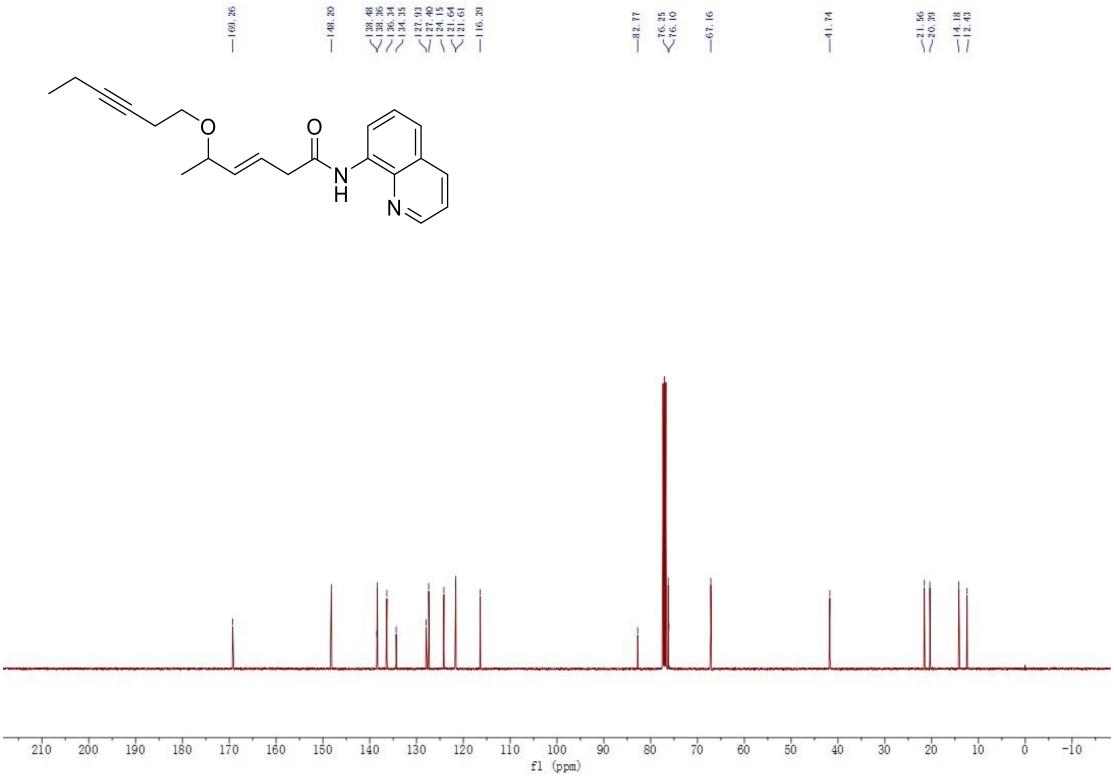

[1]**H NMR**-spectrum (400 MHz, CDCl3) of **52**

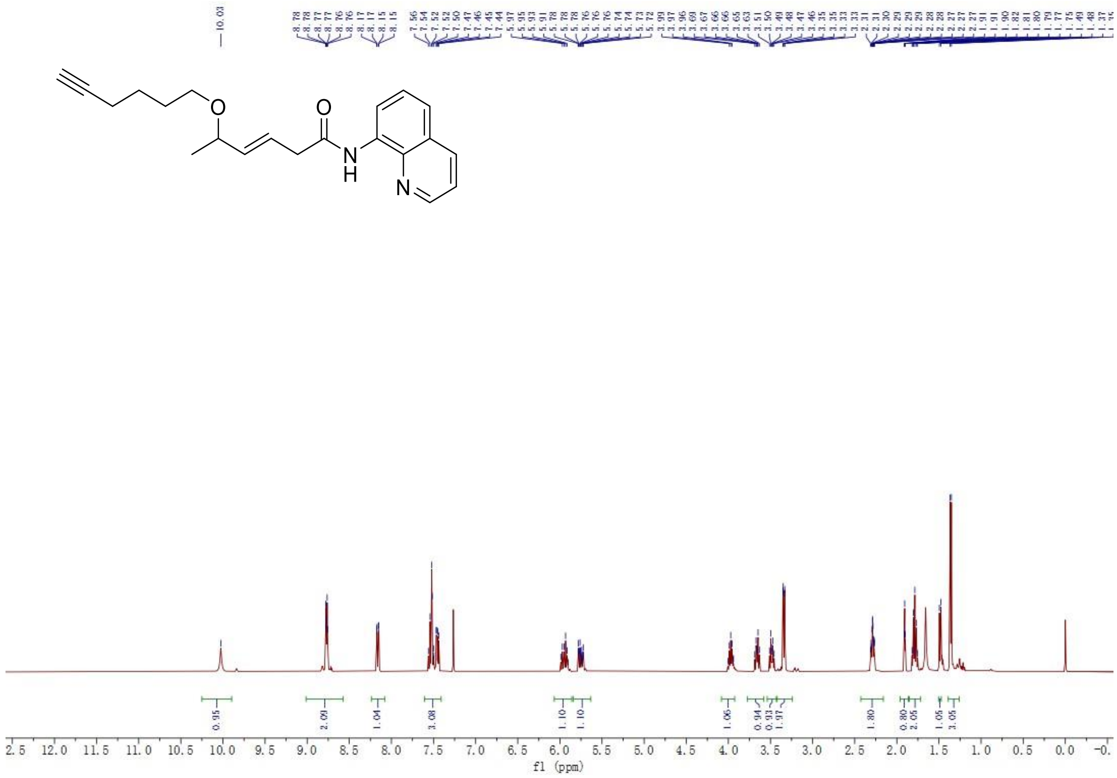

[13]**C NMR**-spectrum (101 MHz, CDCl3) of **52**

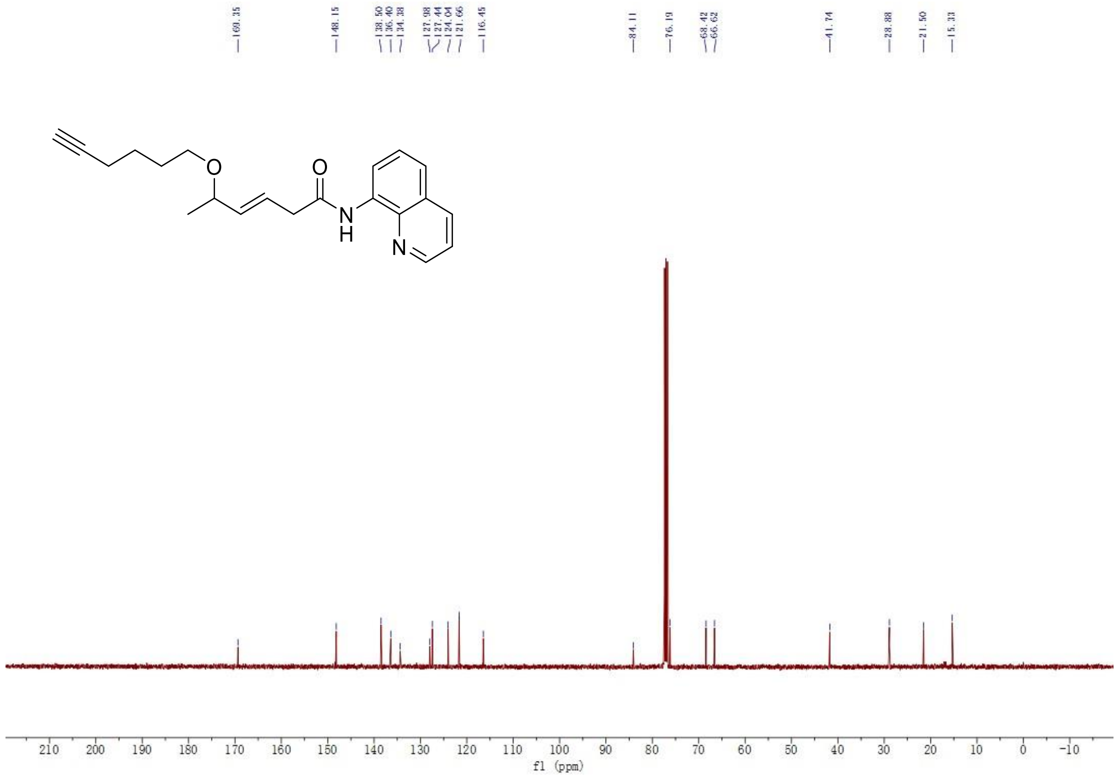

^{1}H NMR-spectrum (400 MHz, $CDCl_3$) of **53**

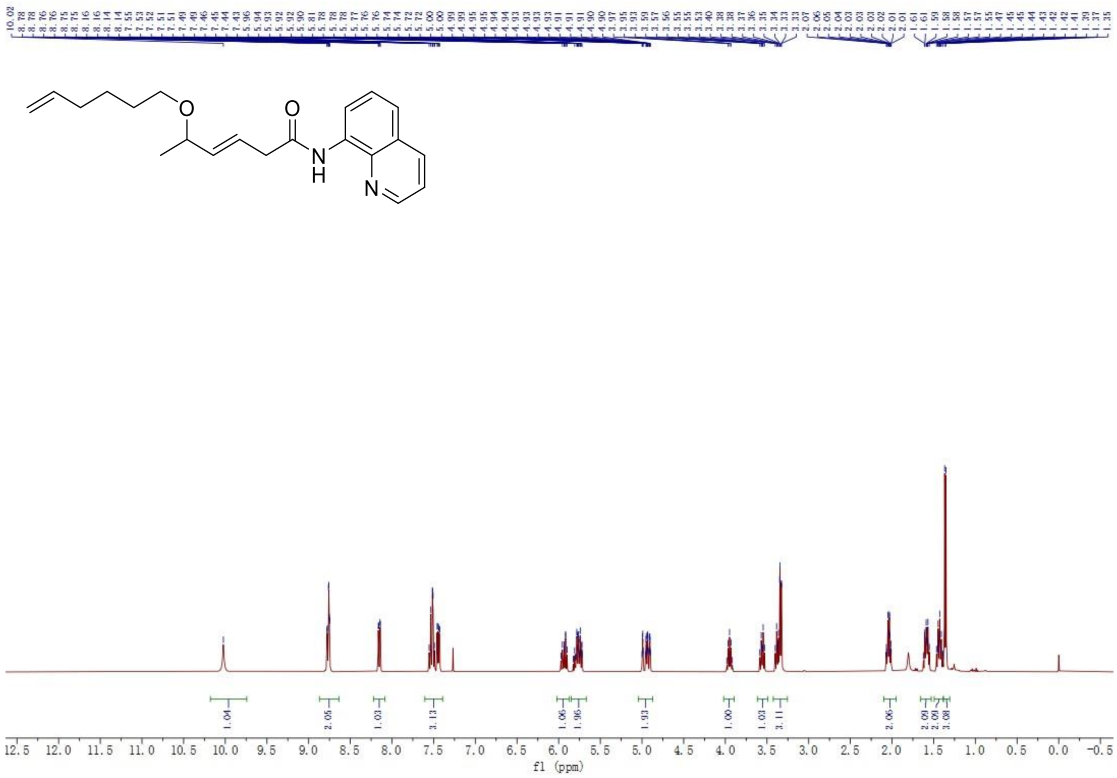

^{13}C NMR-spectrum (101 MHz, $CDCl_3$) of **53**

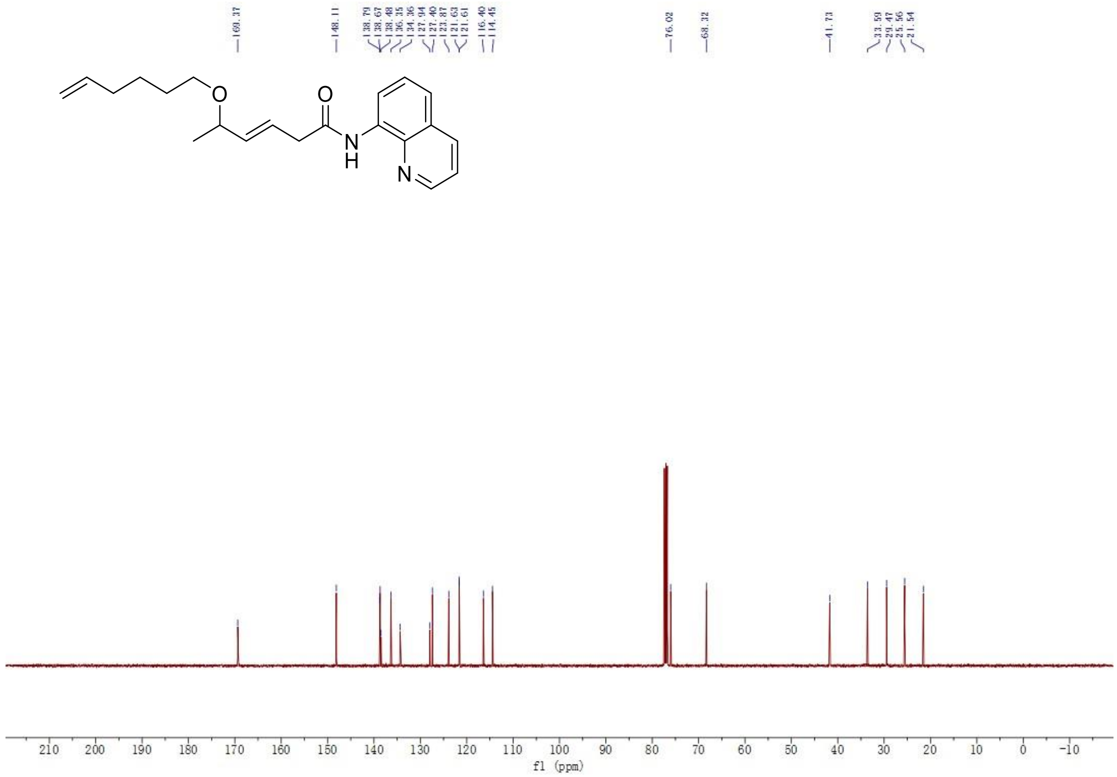

^{1}H NMR-spectrum (400 MHz, $CDCl_3$) of **54**

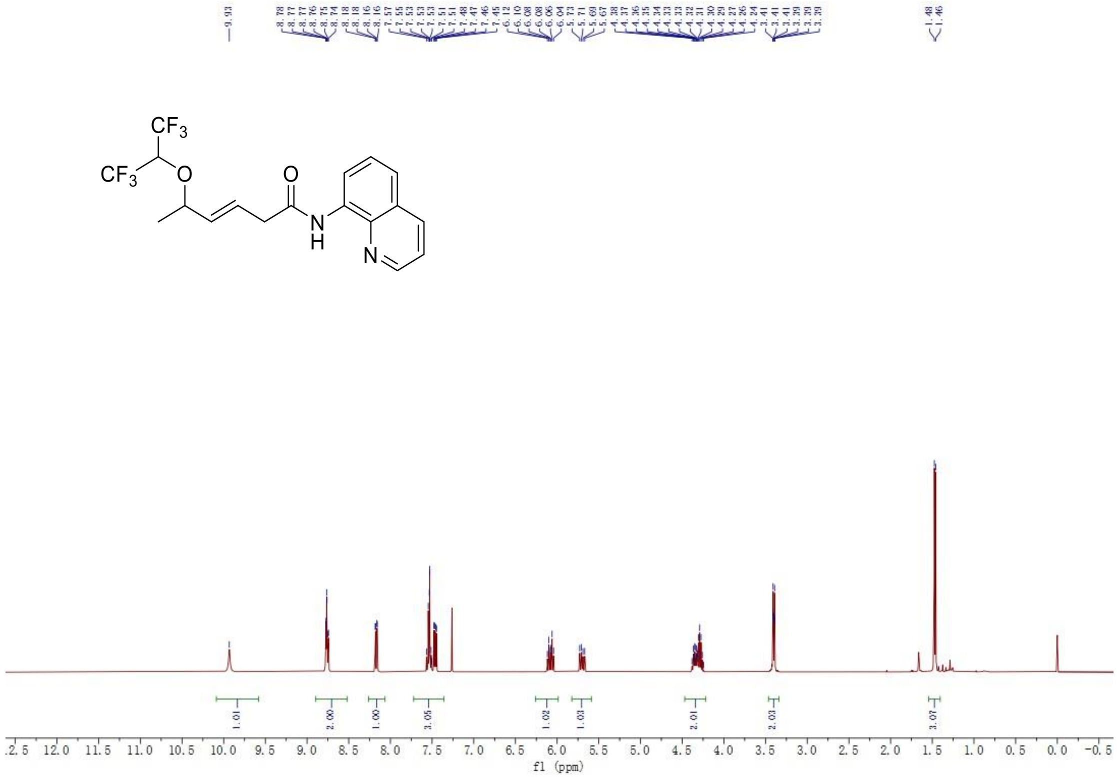

^{13}C NMR-spectrum (101 MHz, $CDCl_3$) of **54**

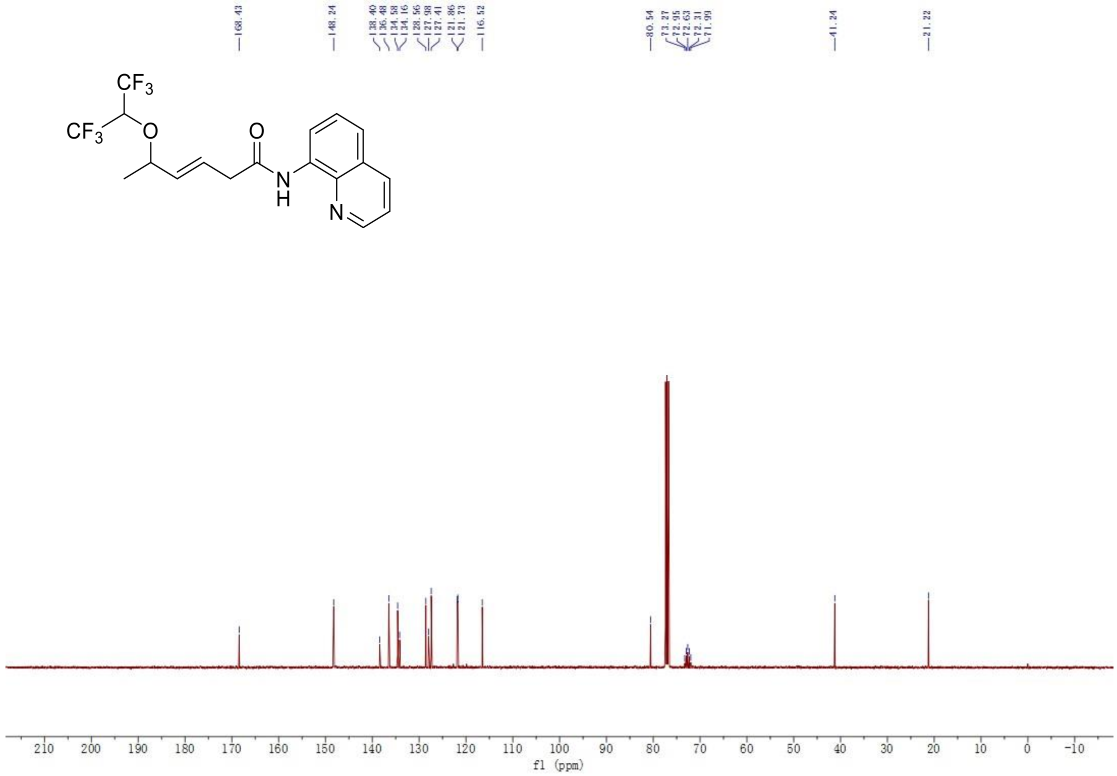

[19]**F NMR**-spectrum (376 MHz, $CDCl_3$) of **54**

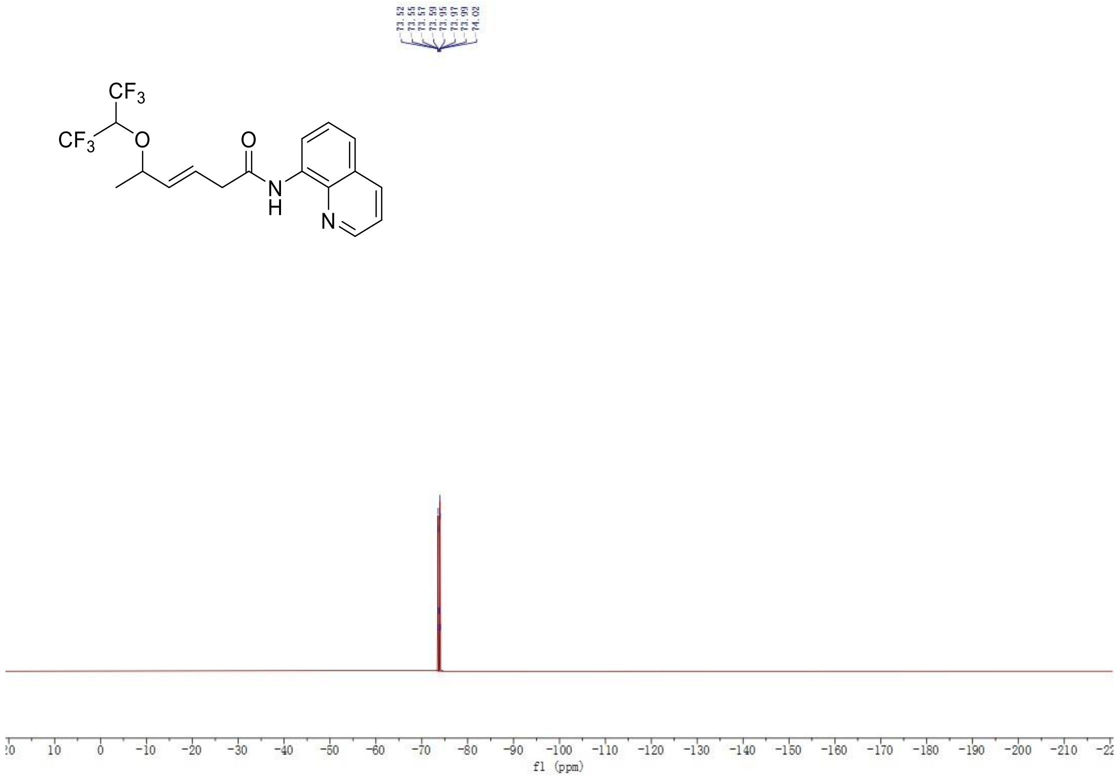

[1]**H NMR**-spectrum (400 MHz, $CDCl_3$) of **55**

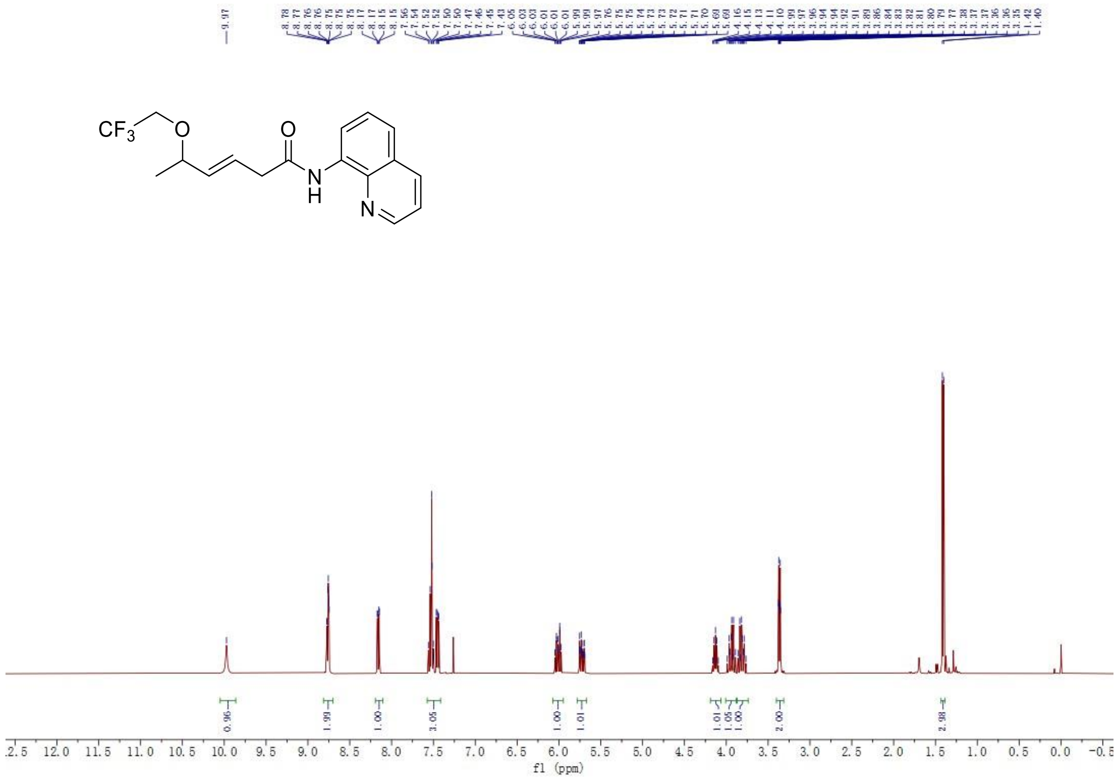

^{13}**C NMR**-spectrum (101 MHz, $CDCl_3$) of **55**

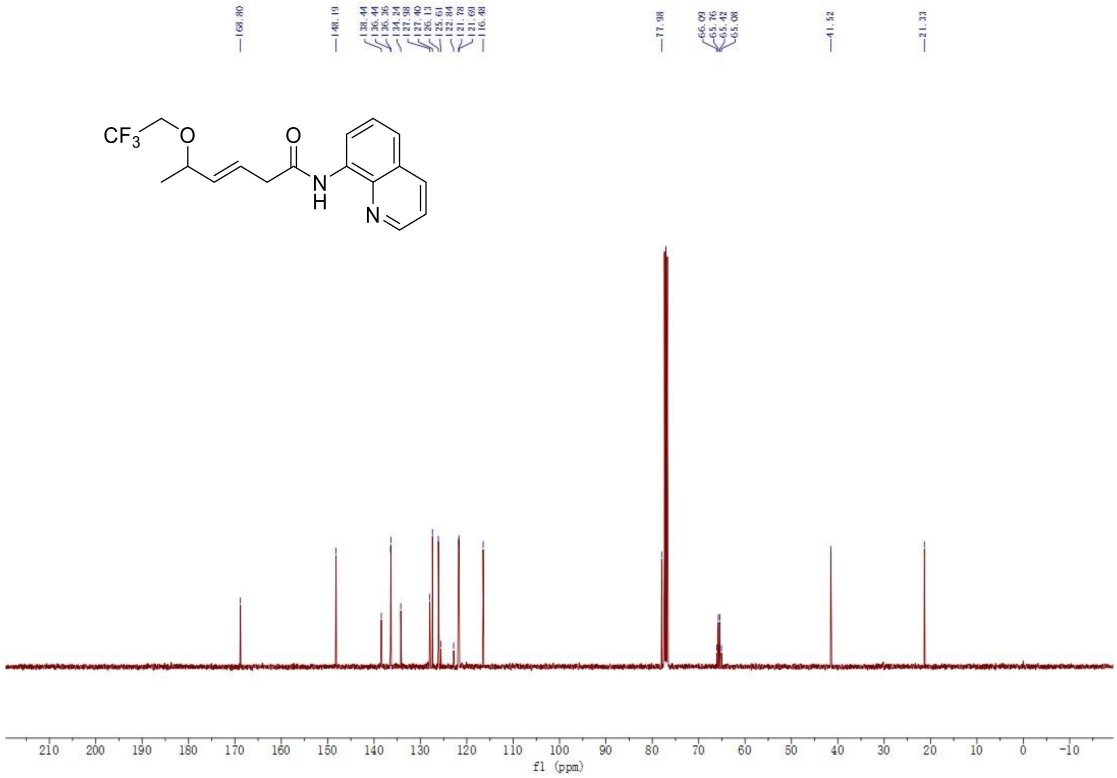

^{19}**F NMR**-spectrum (376 MHz, $CDCl_3$) of **55**

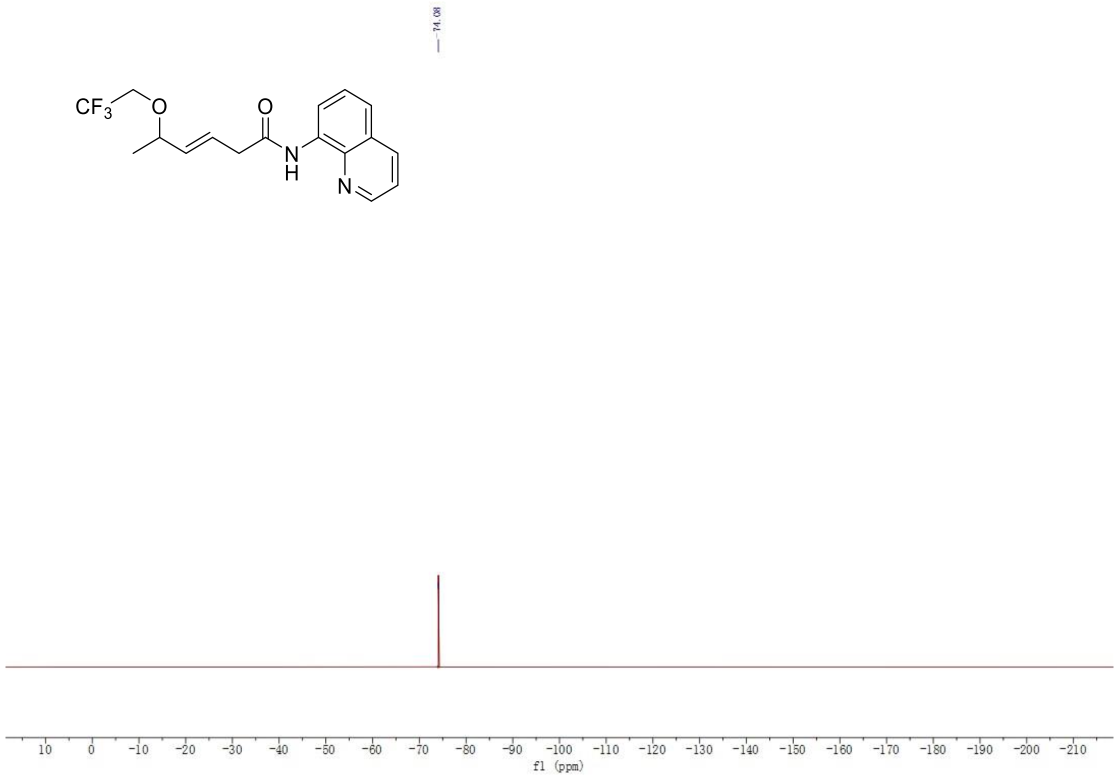

^{1}**H NMR**-spectrum (400 MHz, CDCl$_3$) of **56**

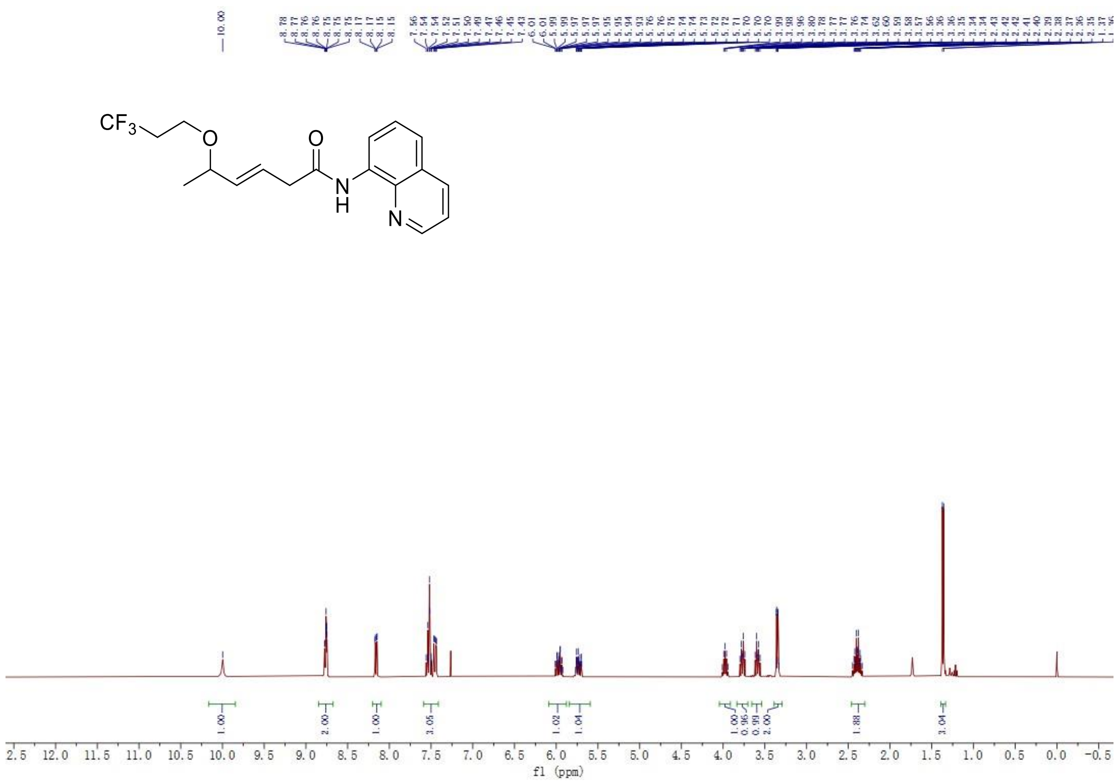

^{13}**C NMR**-spectrum (101 MHz, CDCl$_3$) of **56**

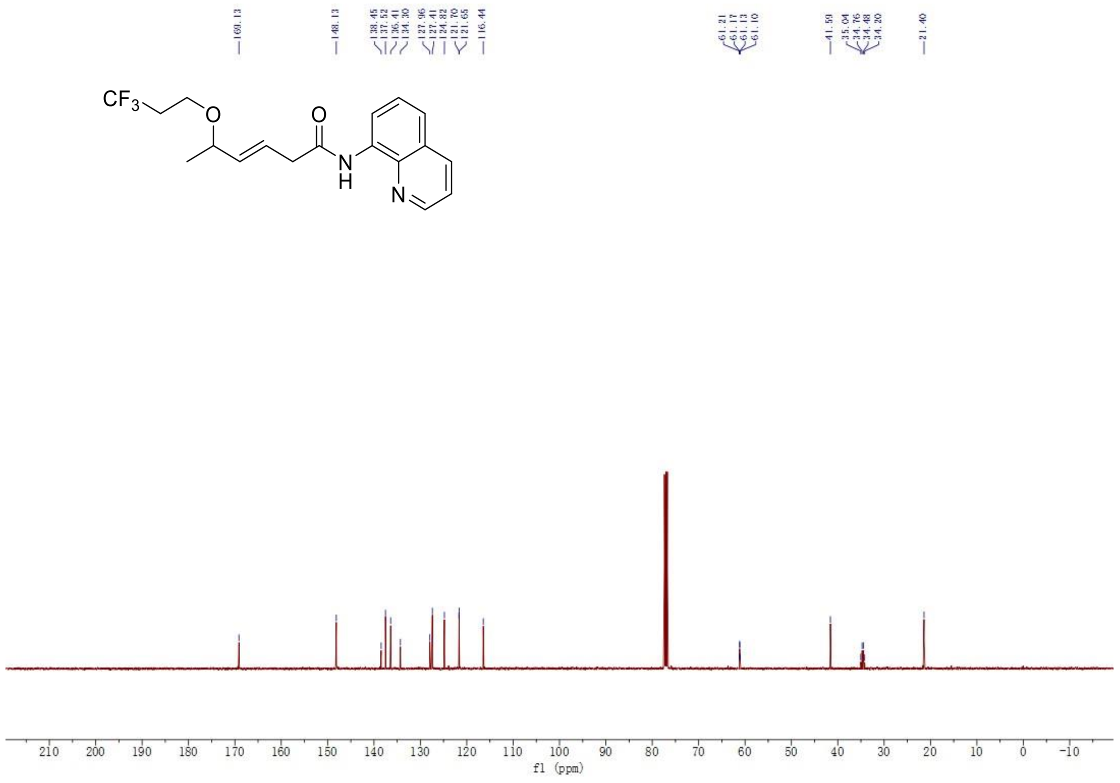

[19]**F NMR**-spectrum (376 MHz, CDCl3) of **56**

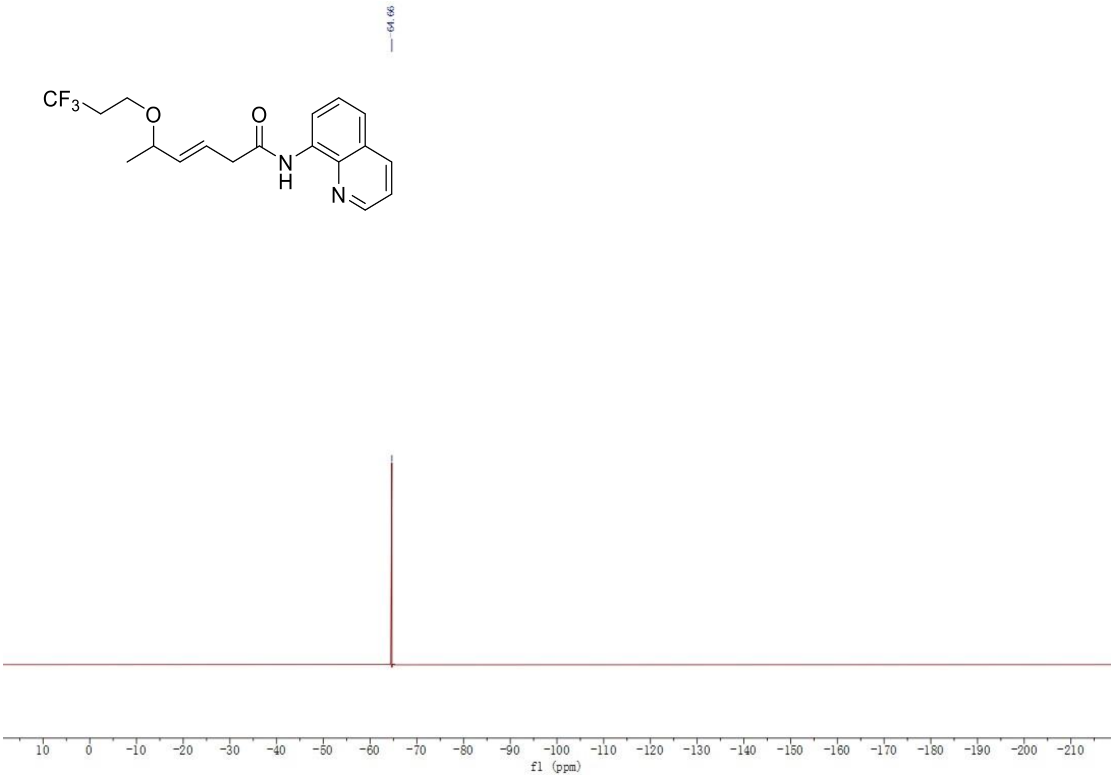

[1]**H NMR**-spectrum (400 MHz, CDCl3) of **57**

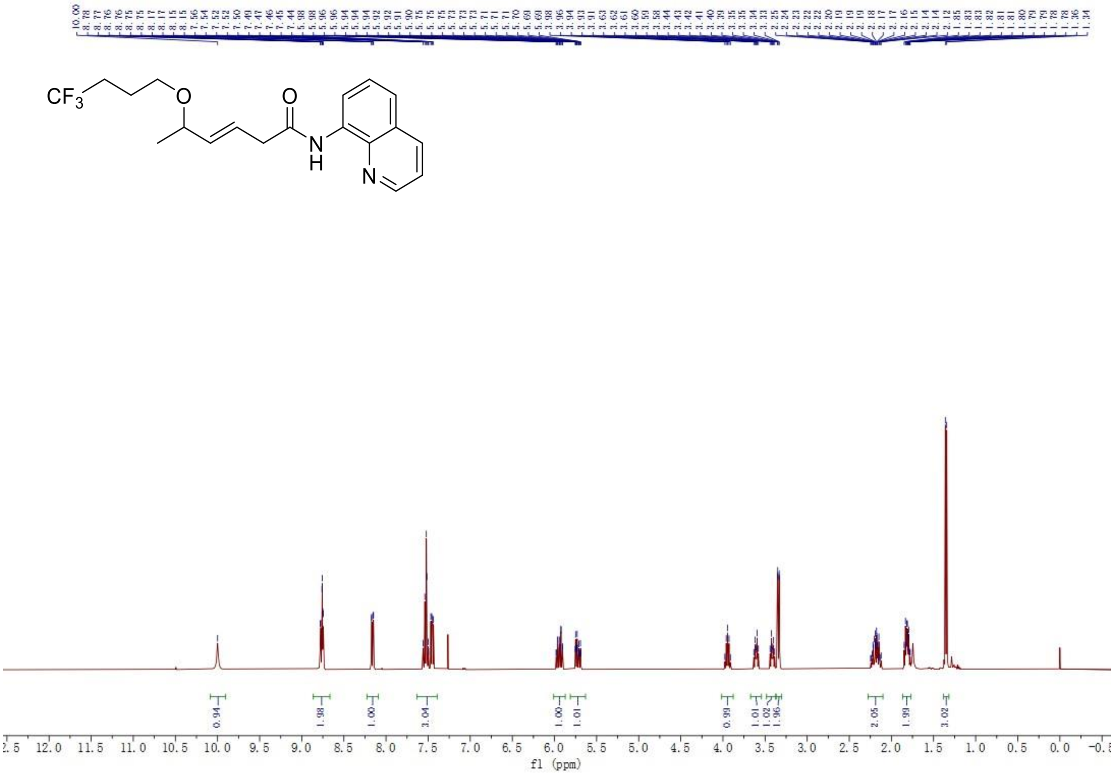

^{13}**C NMR**-spectrum (101 MHz, $CDCl_3$) of **57**

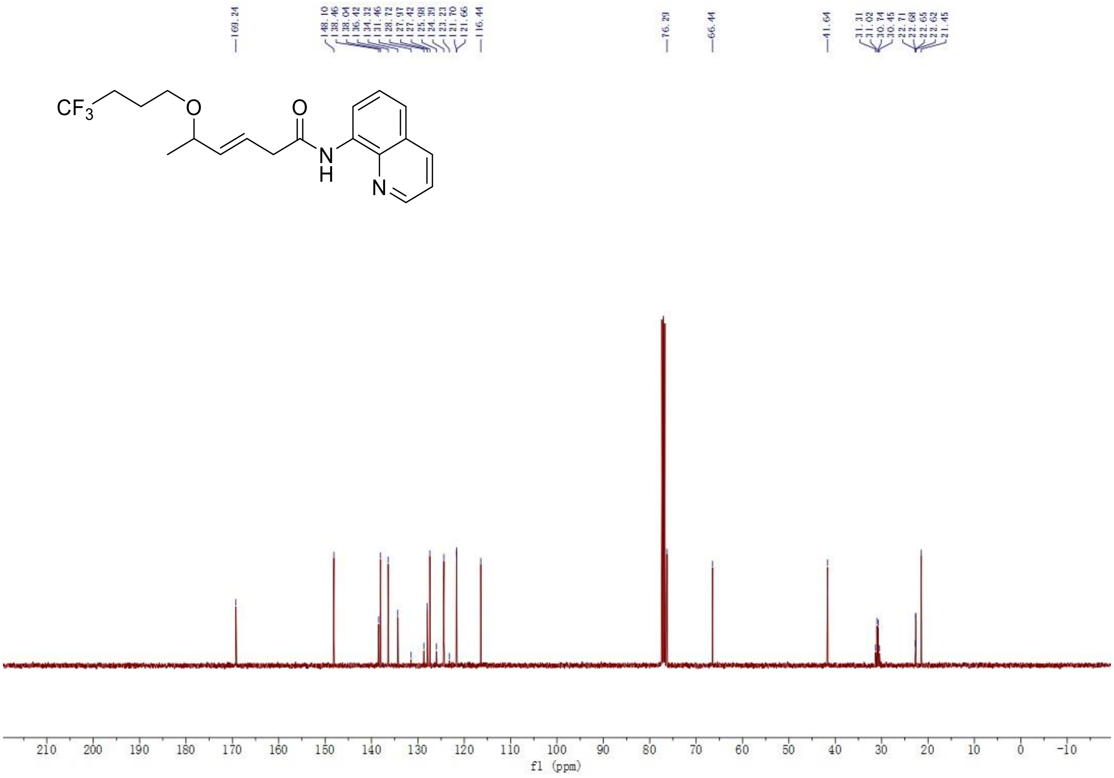

^{19}**F NMR**-spectrum (376 MHz, $CDCl_3$) of **57**

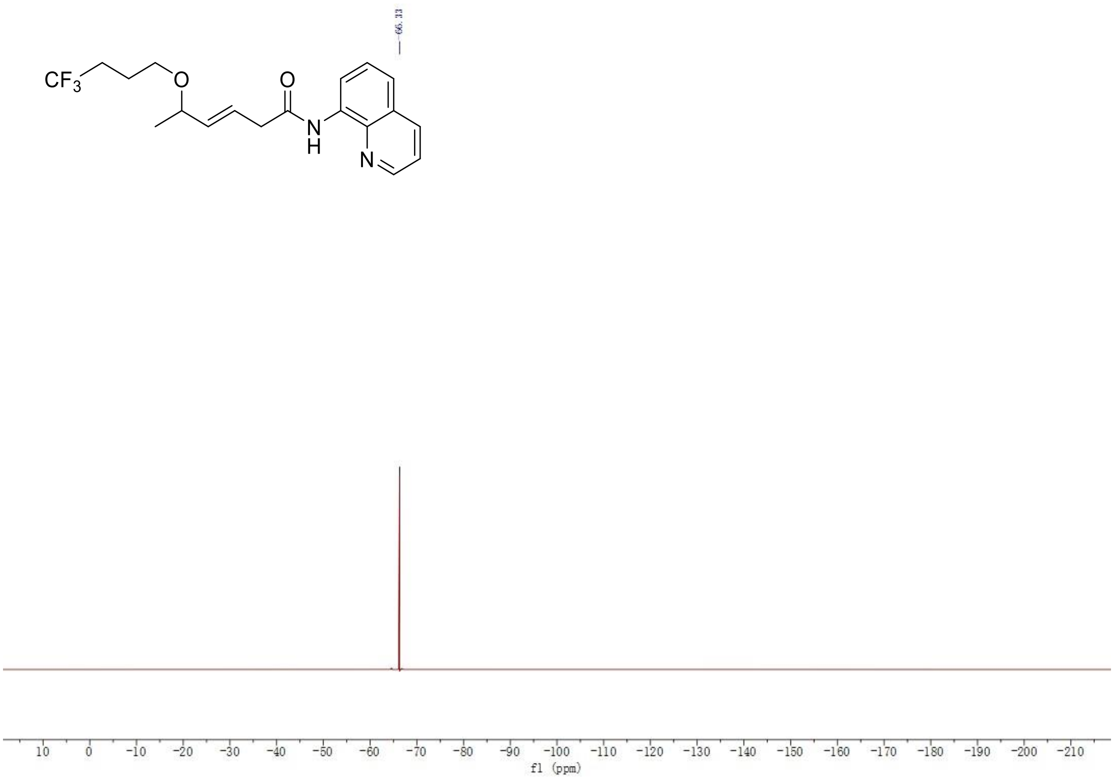

^{1}**H NMR**-spectrum (400 MHz, $CDCl_3$) of **58**

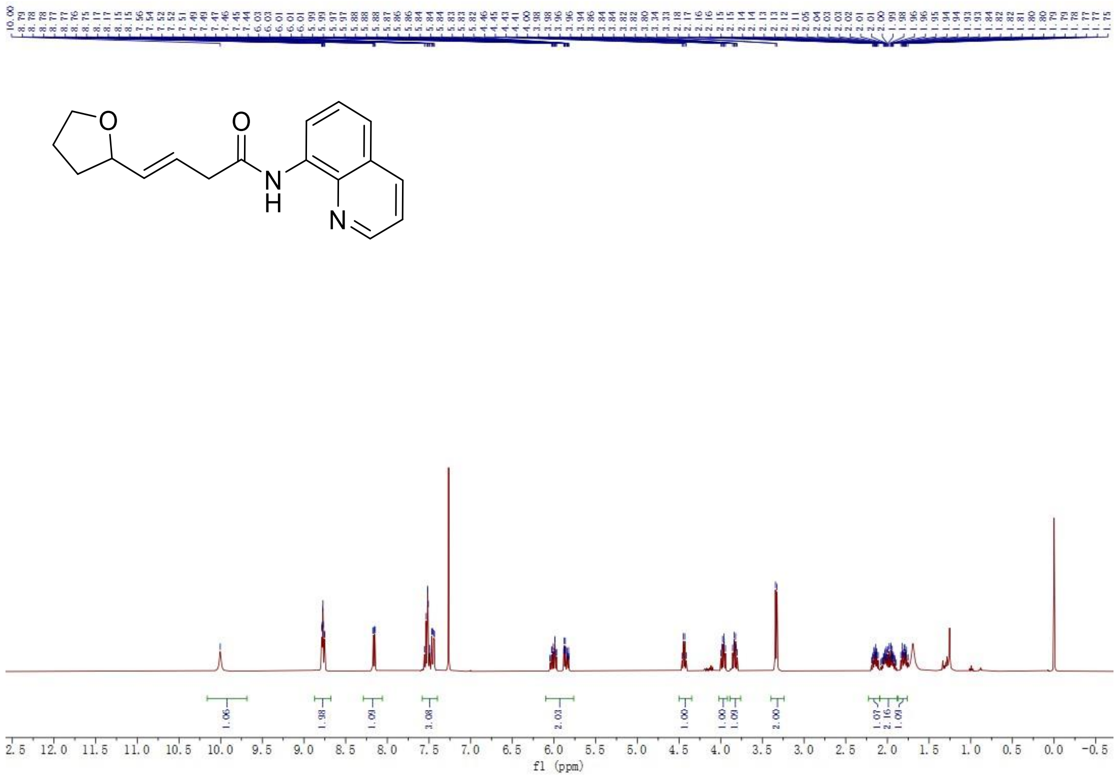

^{13}**C NMR**-spectrum (101 MHz, $CDCl_3$) of **58**

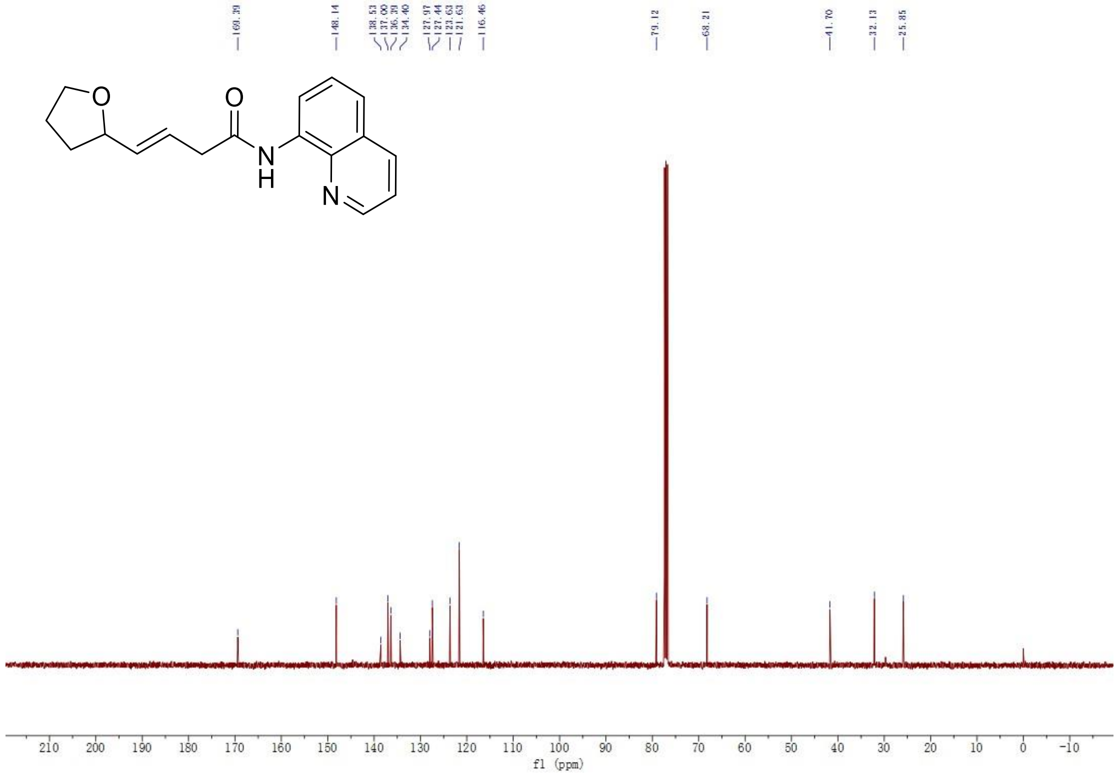

[1]**H NMR**-spectrum (400 MHz, CDCl3) of **59**

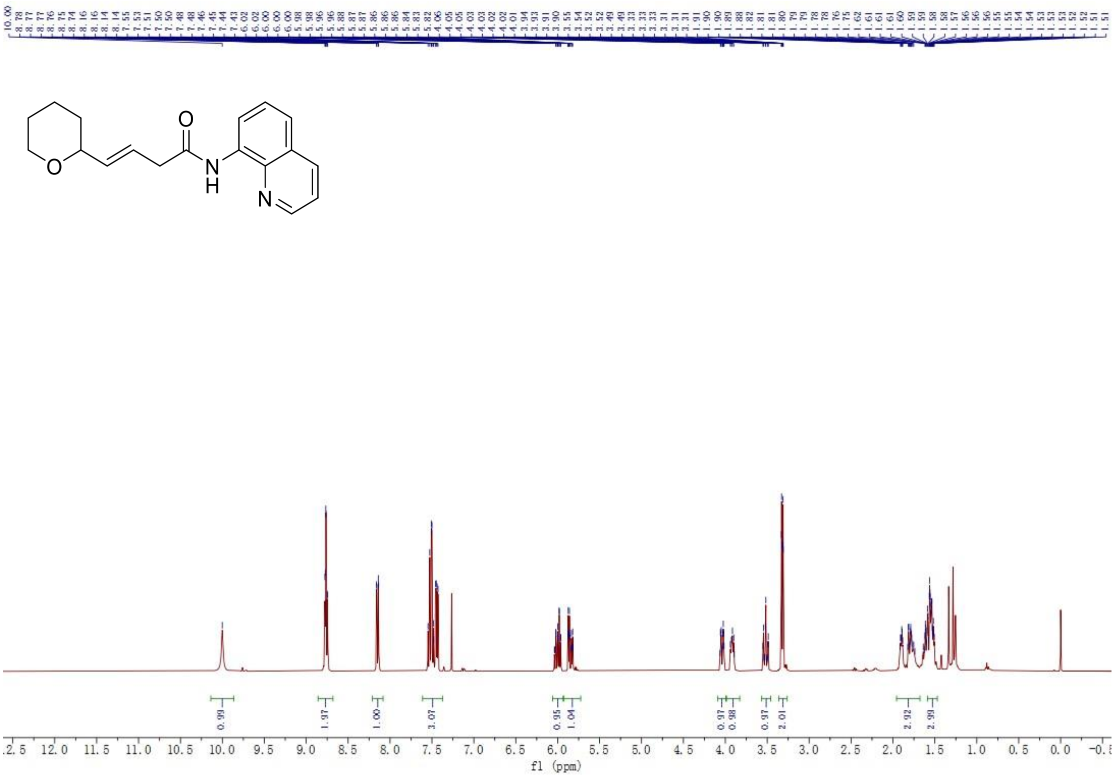

[13]**C NMR**-spectrum (101 MHz, CDCl3) of **59**

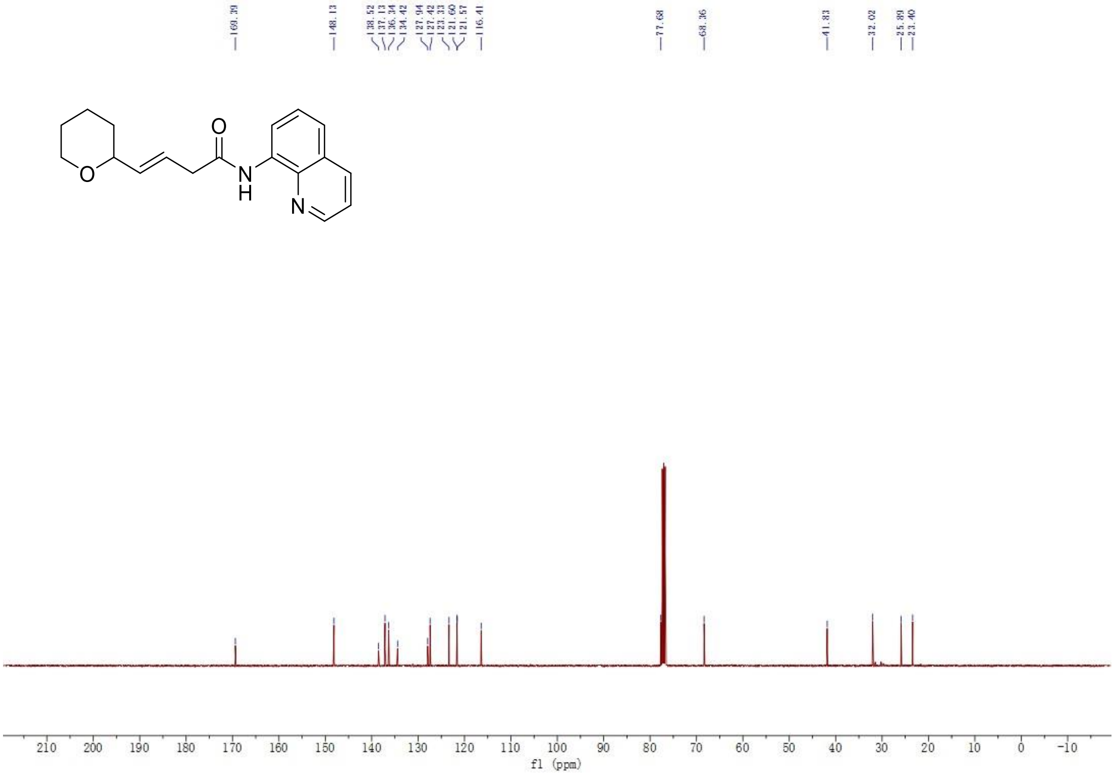

^{1}H NMR-spectrum (400 MHz, $CDCl_3$) of **60**

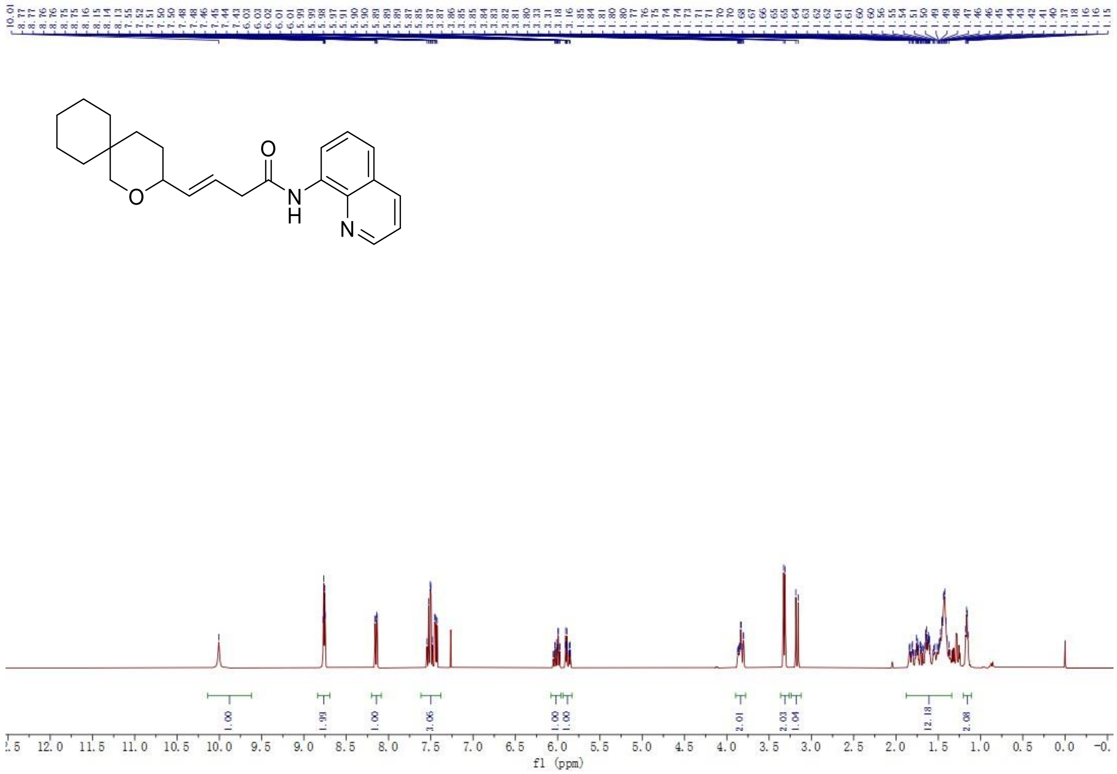

^{13}C NMR-spectrum (101 MHz, $CDCl_3$) of **60**

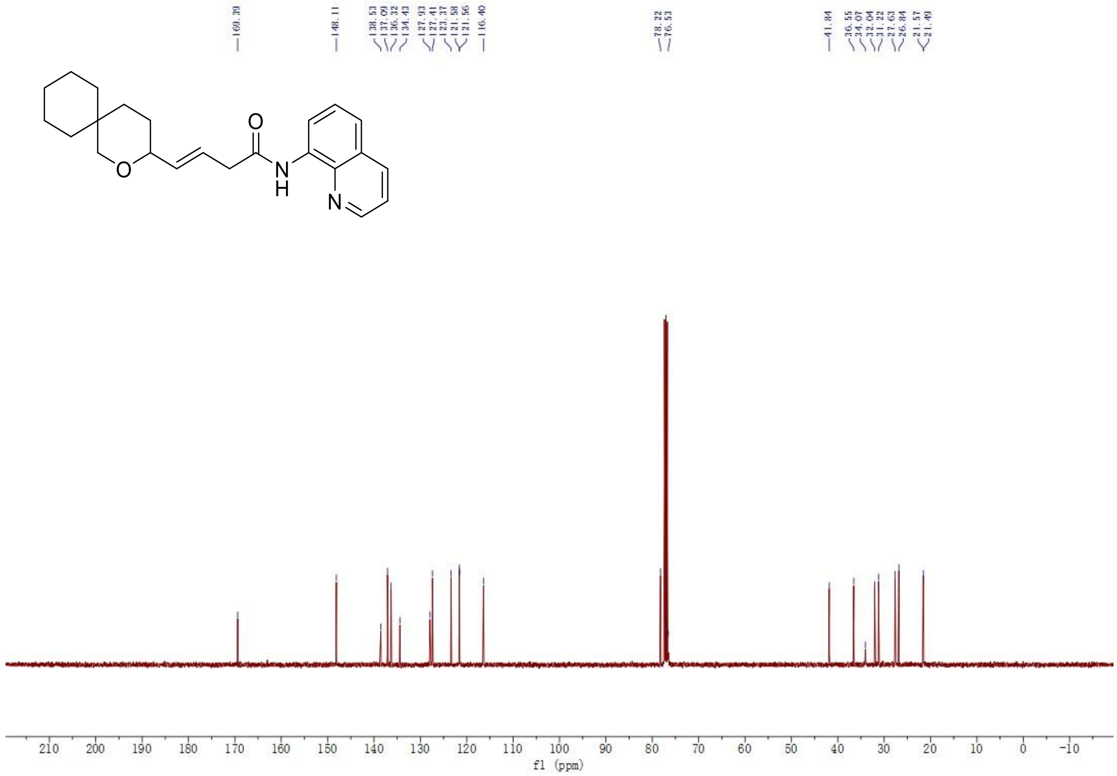

^{1}**H NMR**-spectrum (400 MHz, $CDCl_3$) of **61**

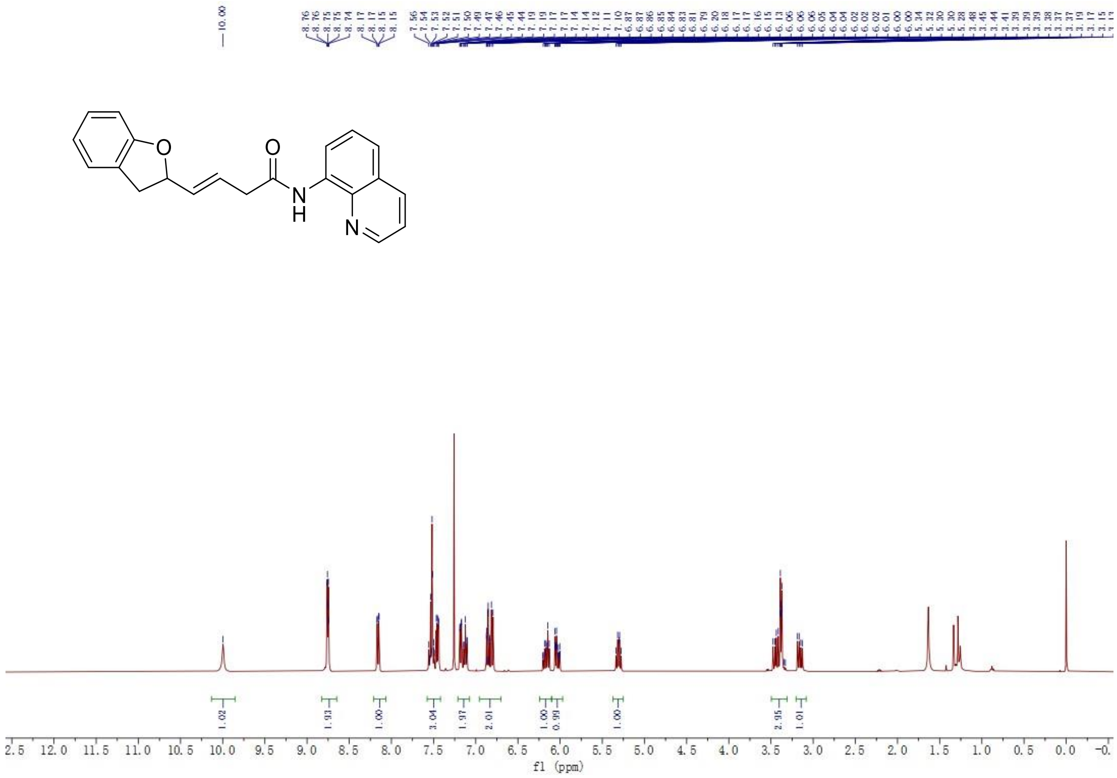

^{13}**C NMR**-spectrum (101 MHz, $CDCl_3$) of **61**

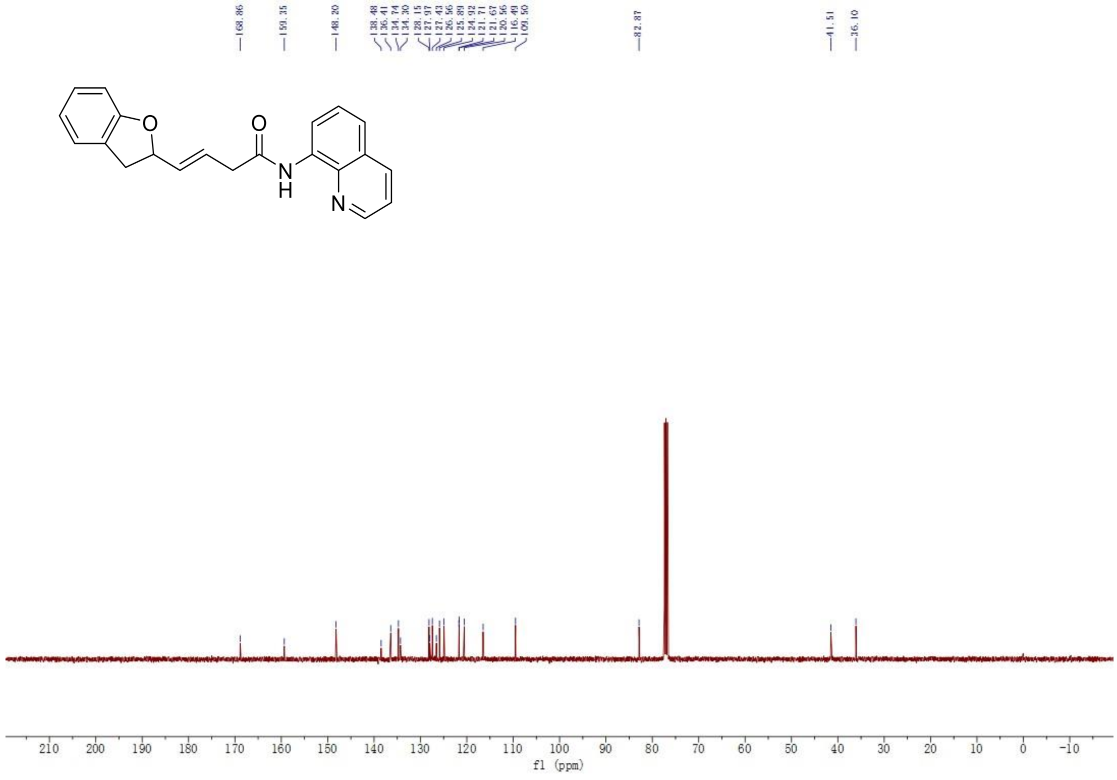

[1]**H NMR**-spectrum (400 MHz, CDCl3) of **62**

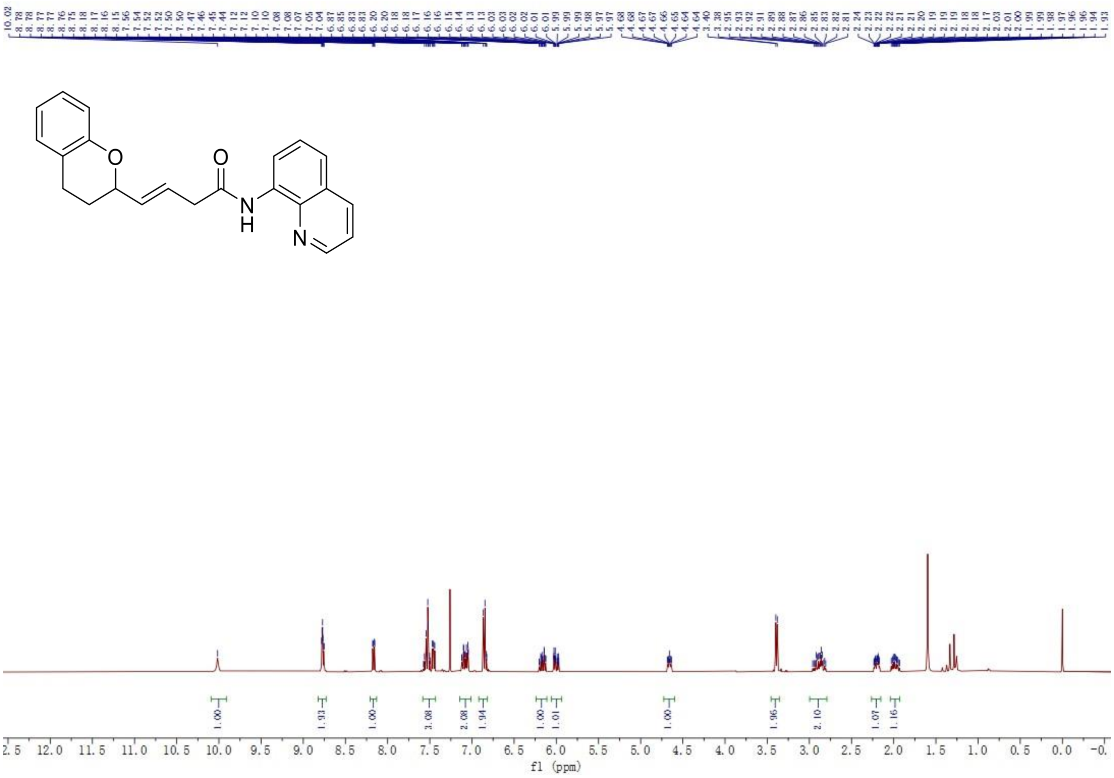

[13]**C NMR**-spectrum (101 MHz, CDCl3) of **62**

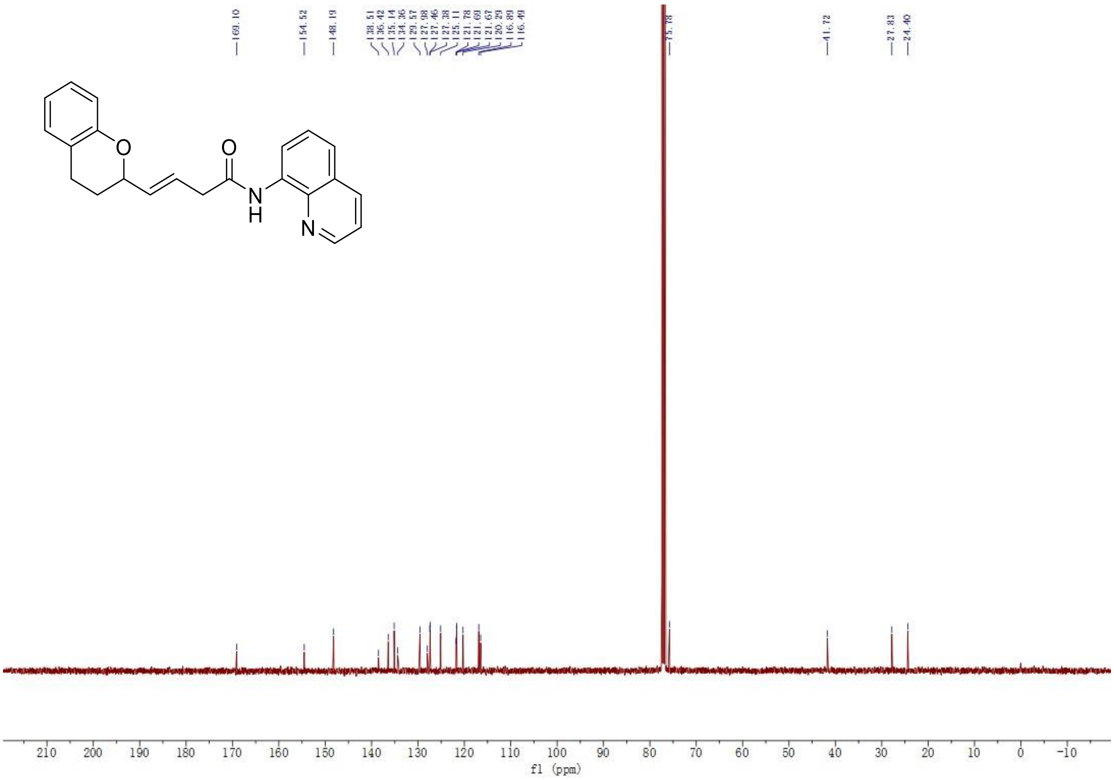

^{1}H NMR-spectrum (400 MHz, $CDCl_3$) of **63**

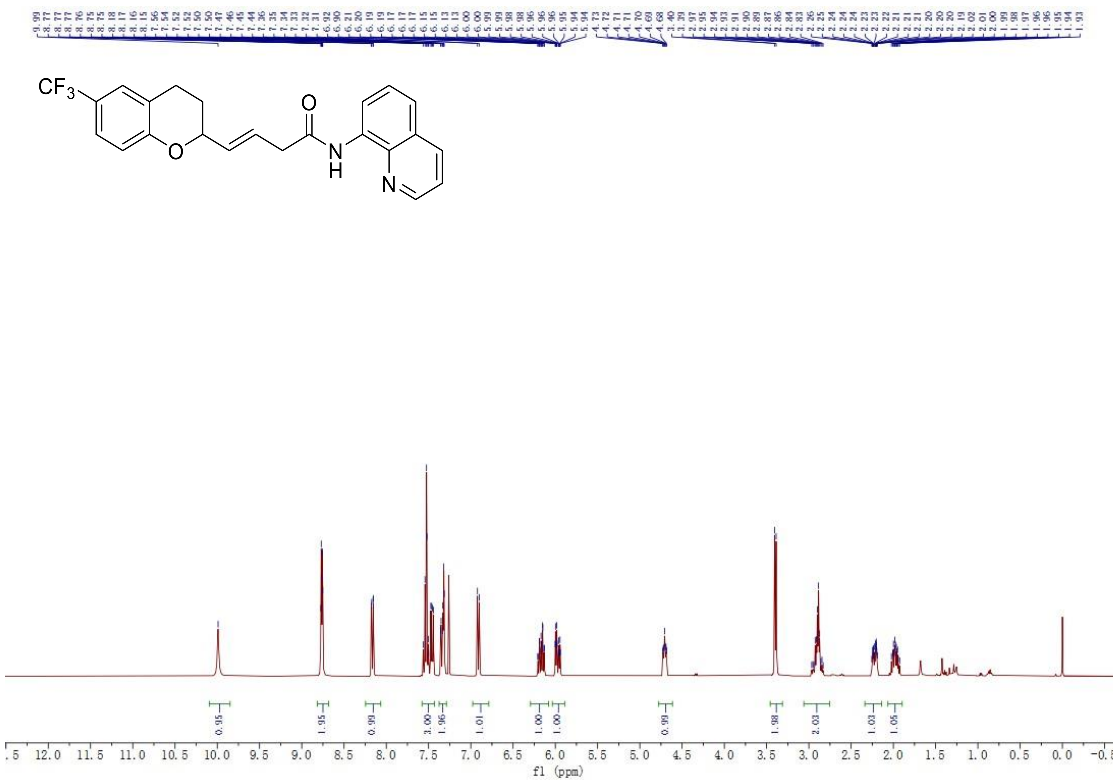

^{13}C NMR-spectrum (101 MHz, $CDCl_3$) of **63**

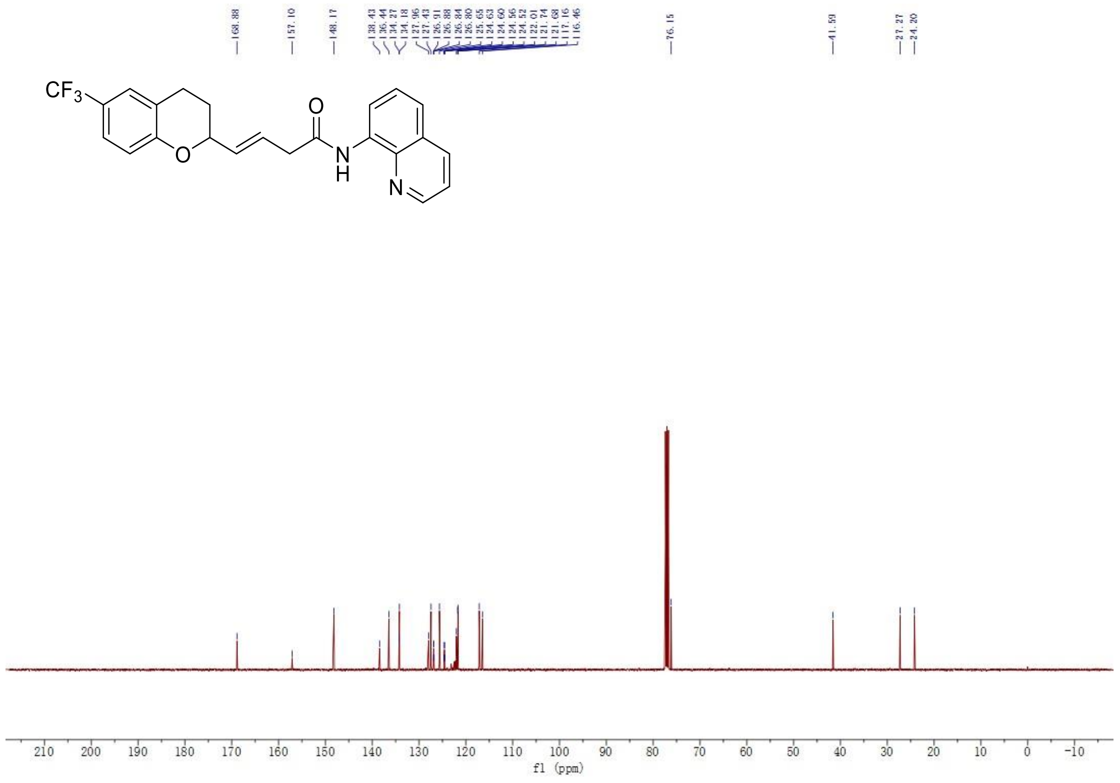

[19]**F NMR**-spectrum (376 MHz, $CDCl_3$) of **63**

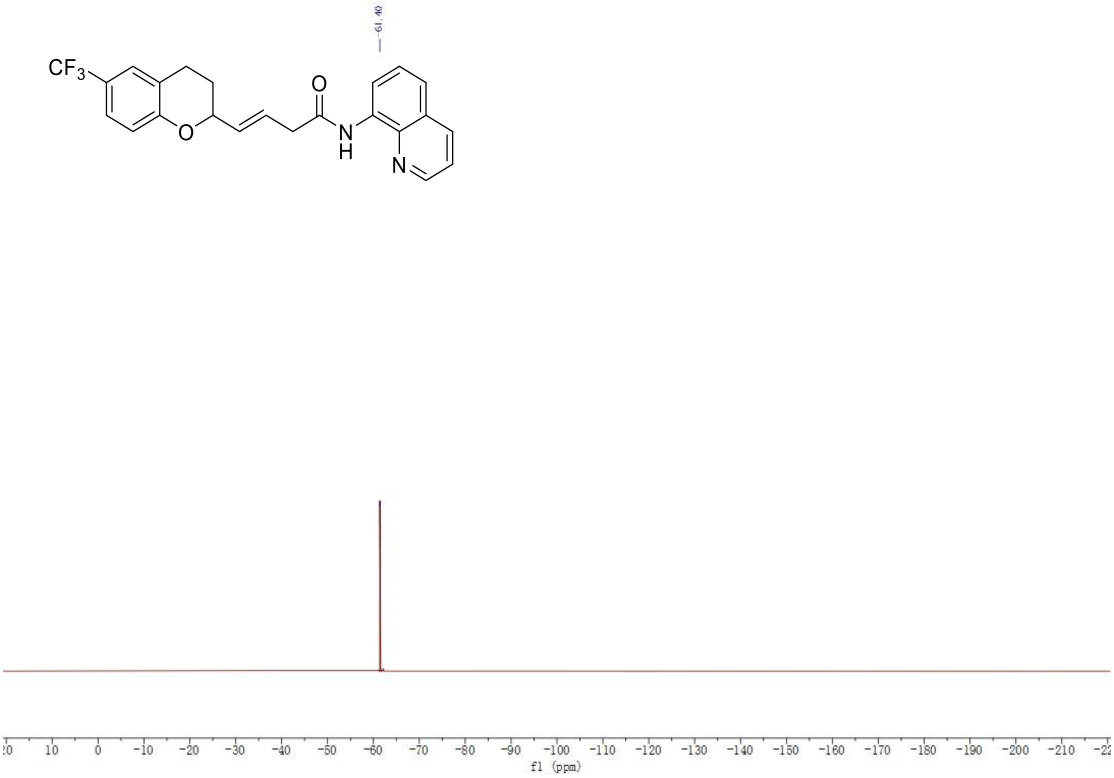

[1]**H NMR**-spectrum (400 MHz, $CDCl_3$) of **64**

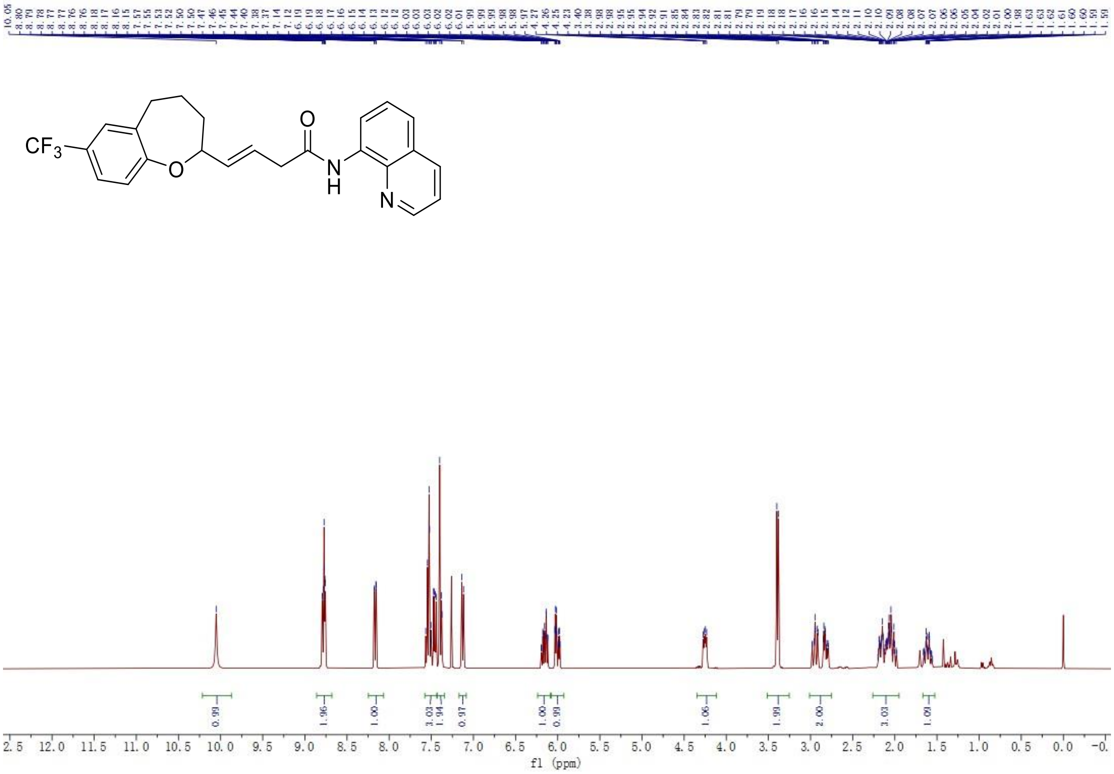

^{13}**C NMR**-spectrum (101 MHz, $CDCl_3$) of **64**

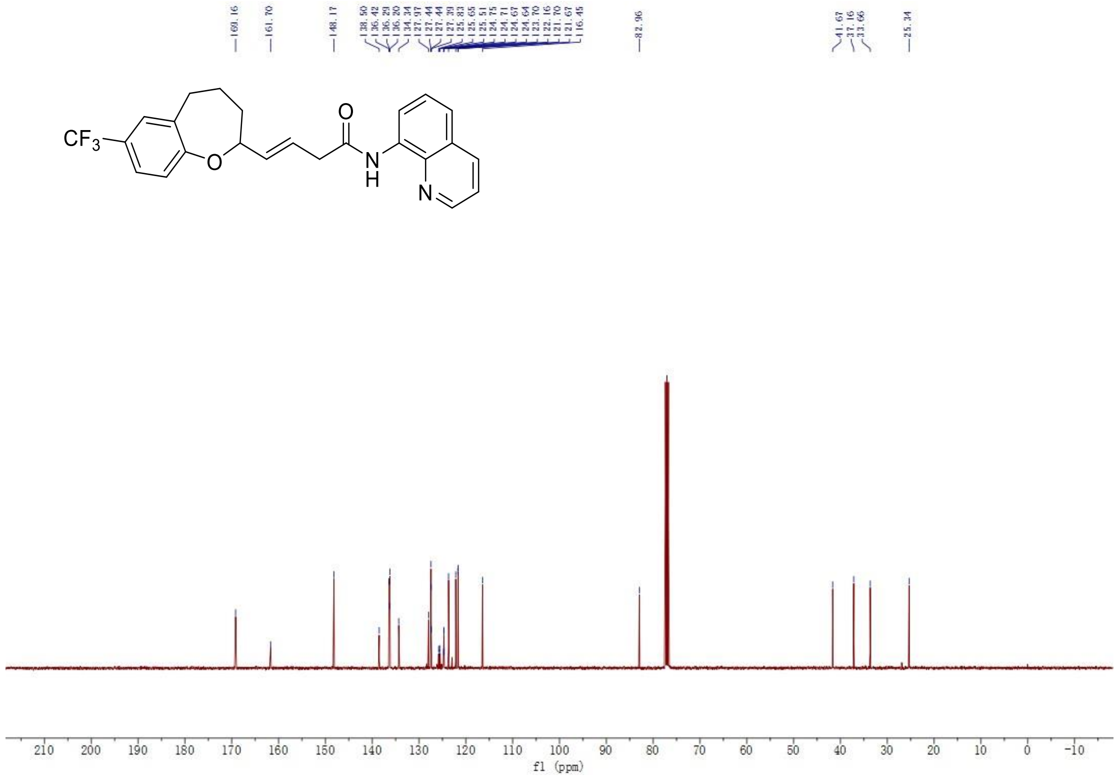

^{19}**F NMR**-spectrum (376 MHz, $CDCl_3$) of **64**

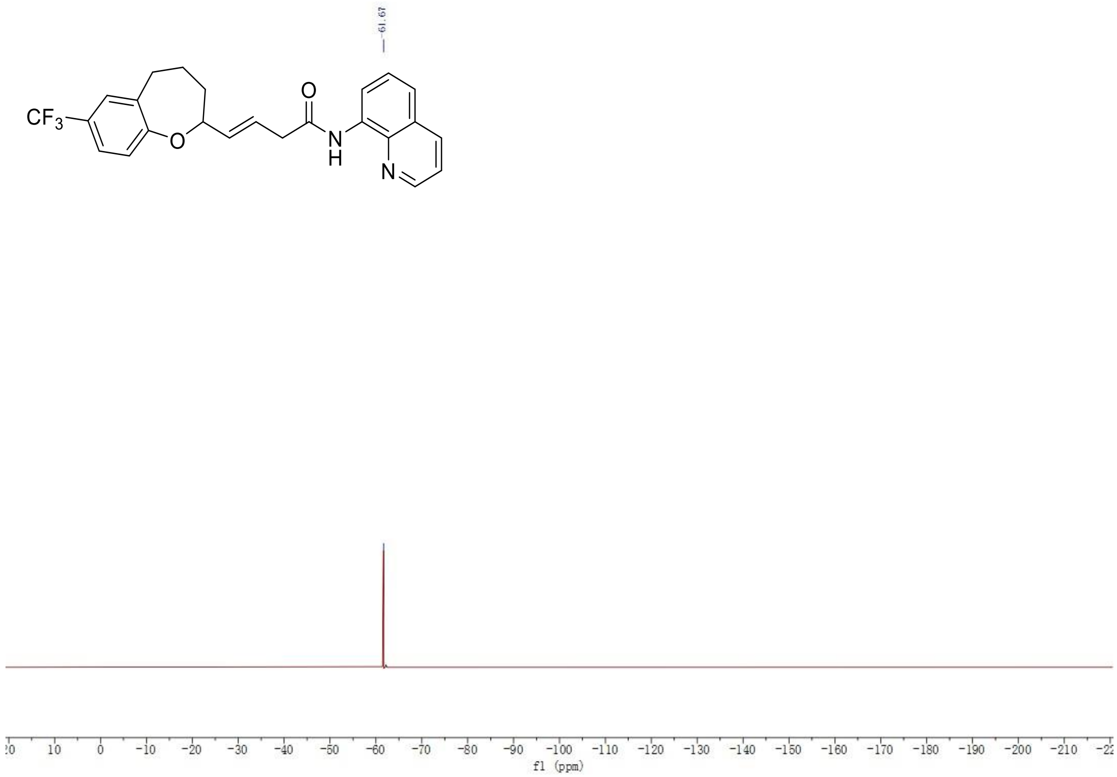

^{1}**H NMR**-spectrum (400 MHz, $CDCl_3$) of **65**

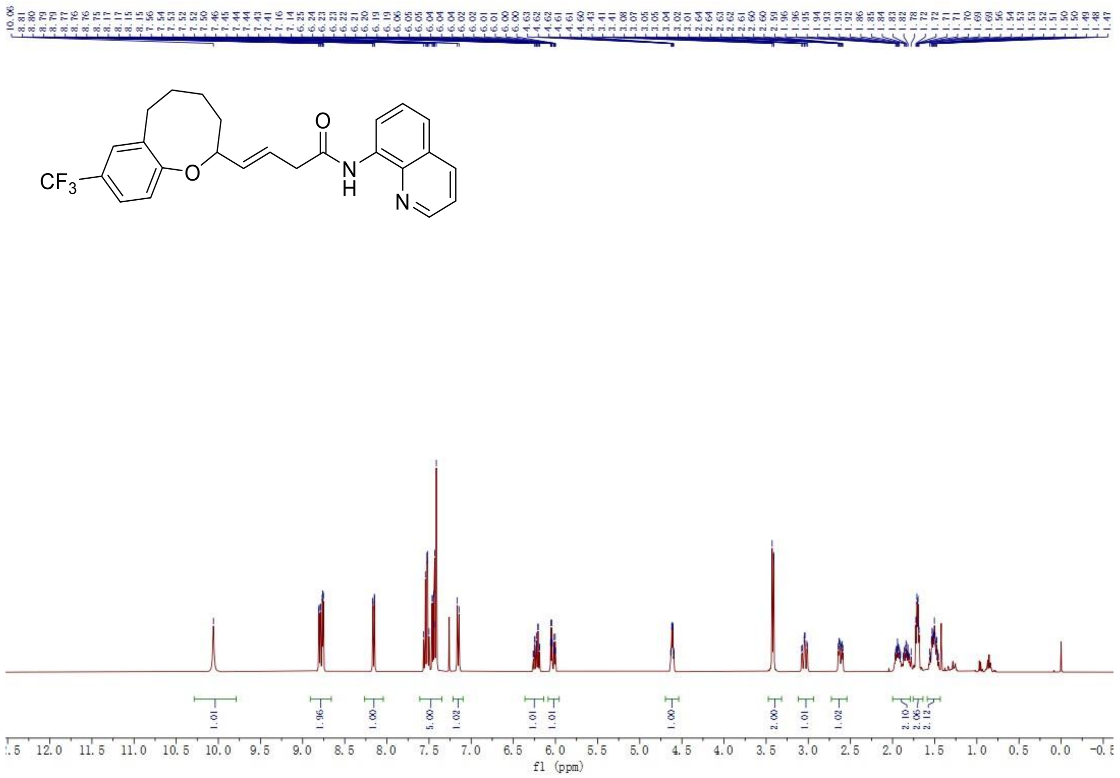

^{13}**C NMR**-spectrum (101 MHz, $CDCl_3$) of **65**

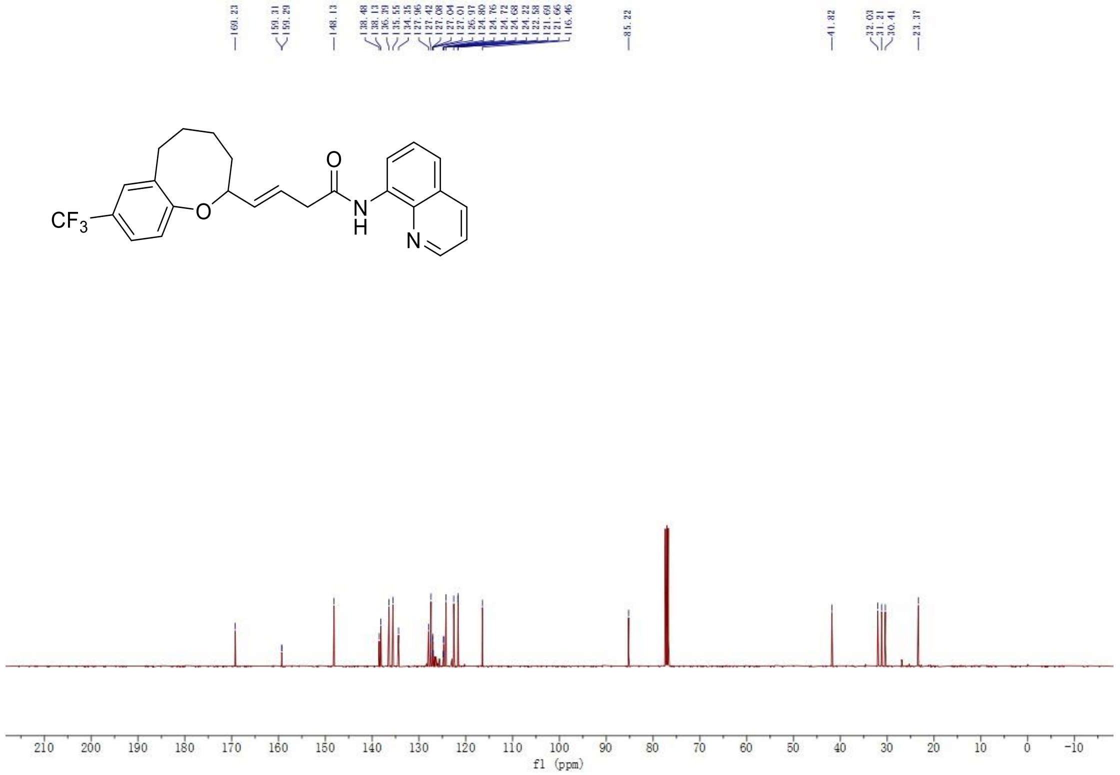

^{19}**F NMR**-spectrum (376 MHz, $CDCl_3$) of **65**

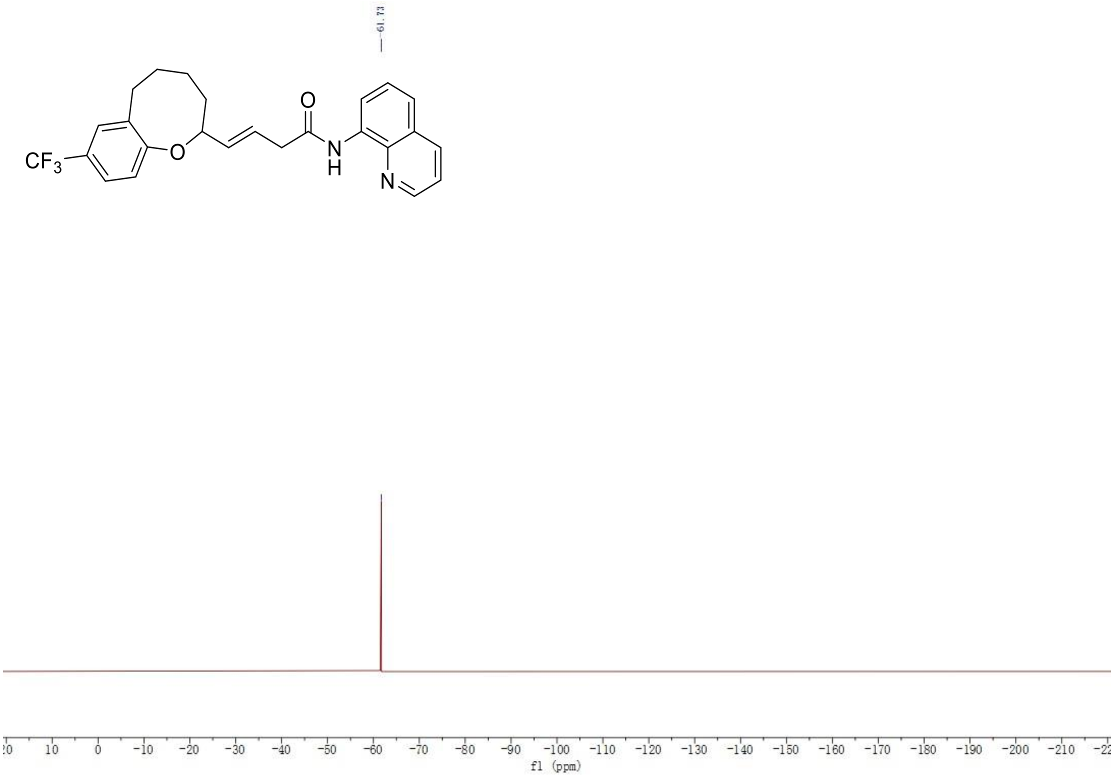

^{1}**H NMR**-spectrum (400 MHz, $CDCl_3$) of **66**

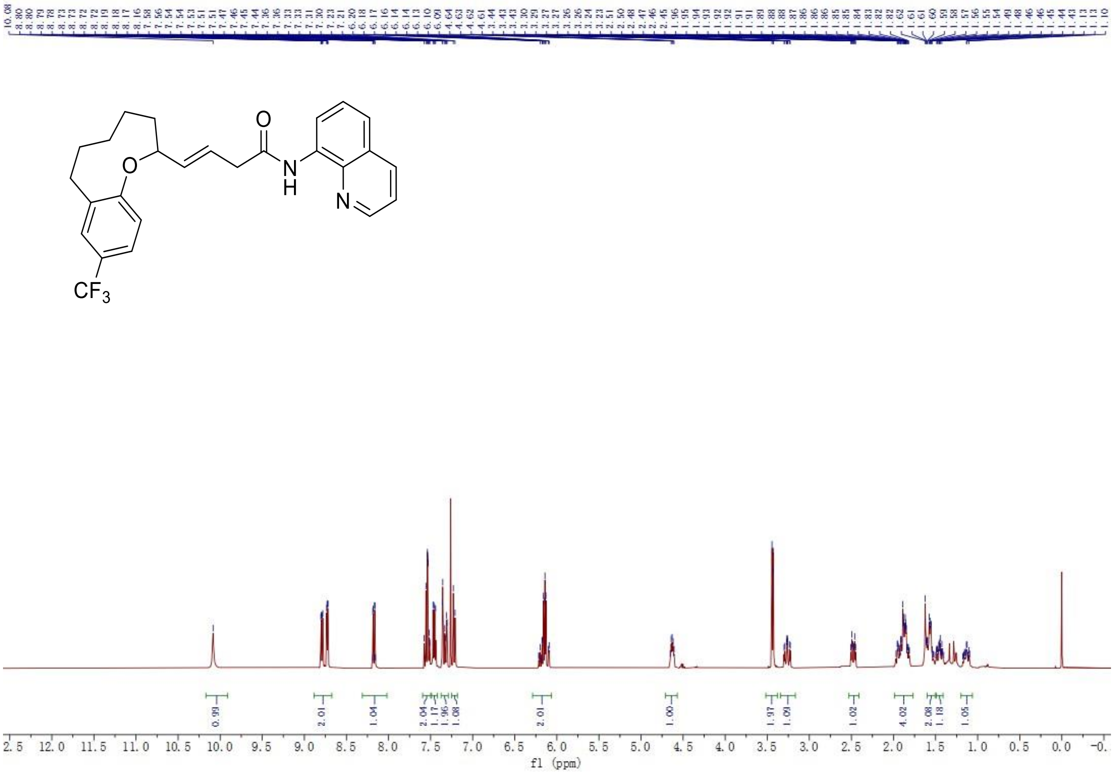

^{13}C NMR-spectrum (101 MHz, CDCl3) of **66**

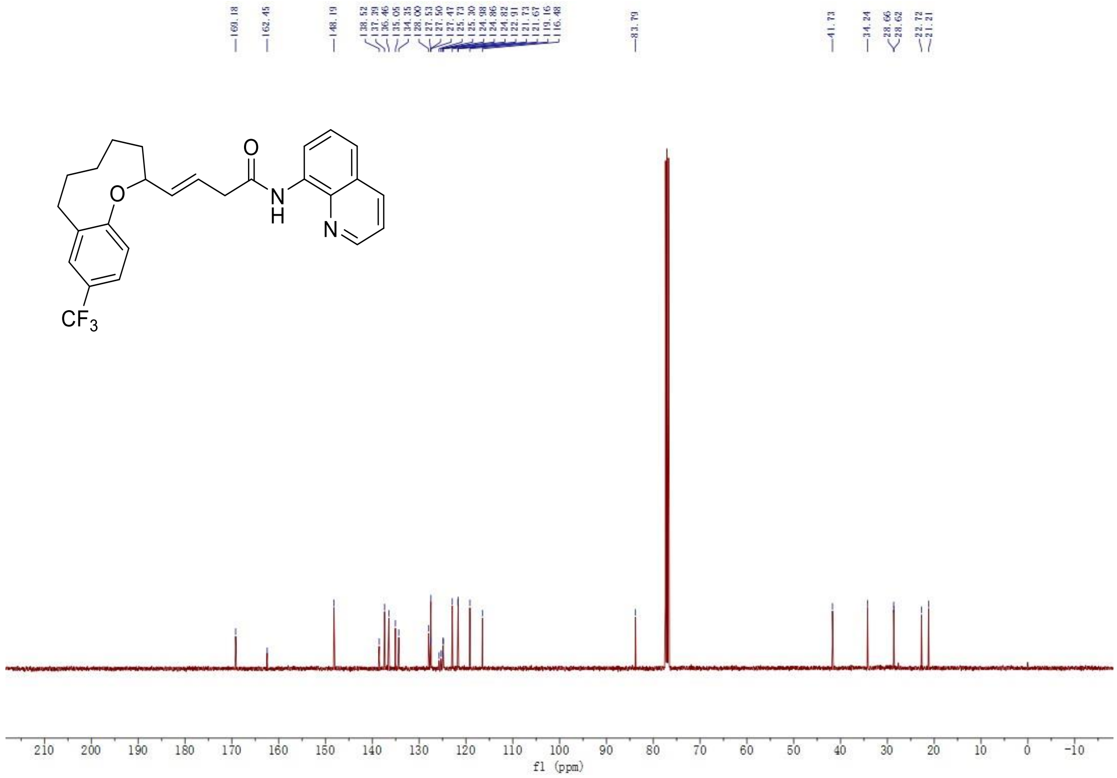

^{19}F NMR-spectrum (376 MHz, CDCl3) of **66**

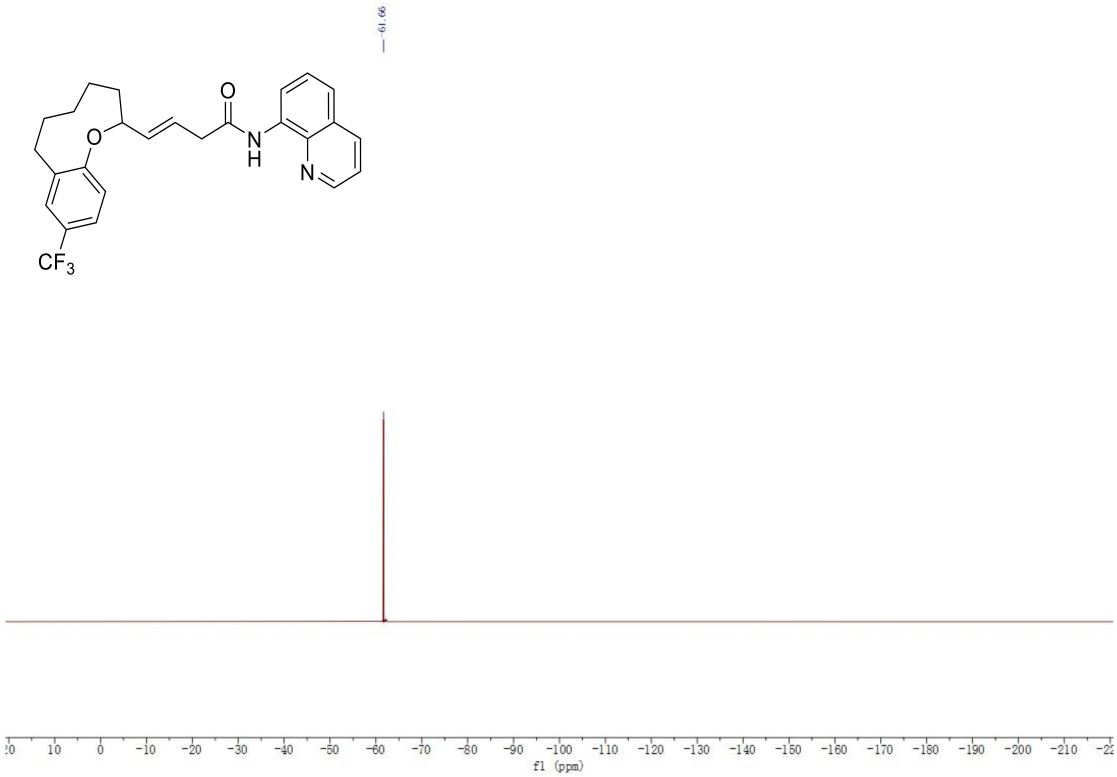

^{1}H NMR-spectrum (400 MHz, $CDCl_3$) of **67**

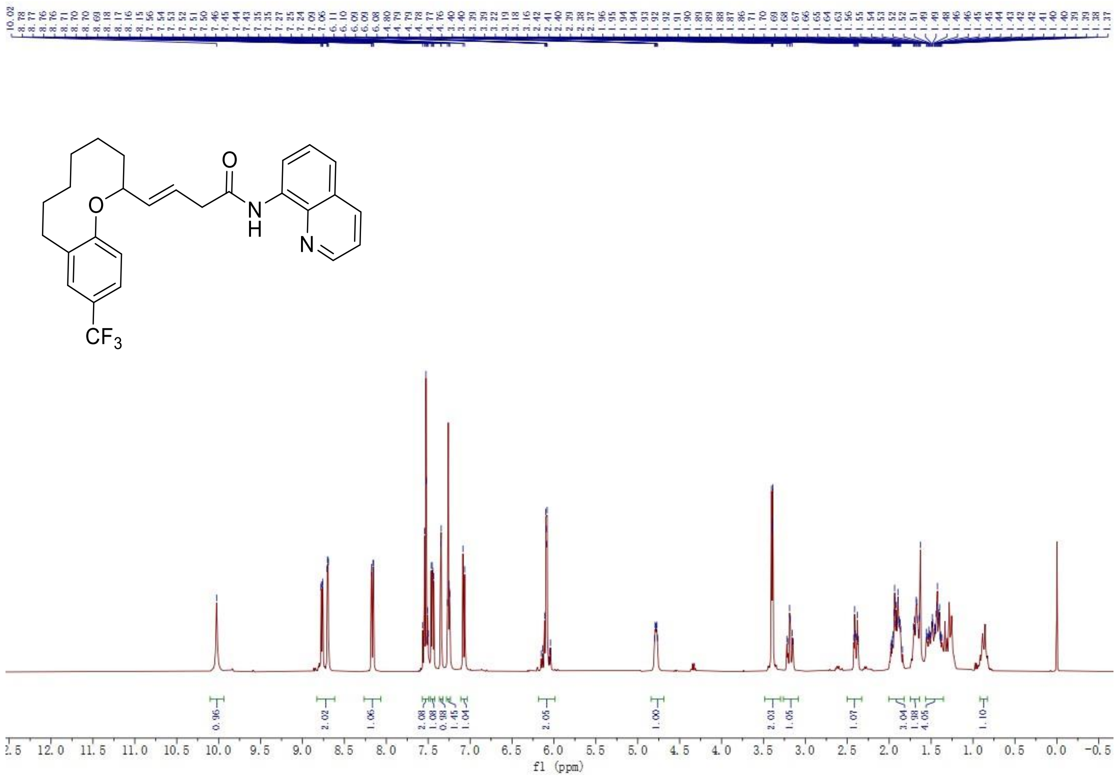

^{13}C NMR-spectrum (101 MHz, $CDCl_3$) of **67**

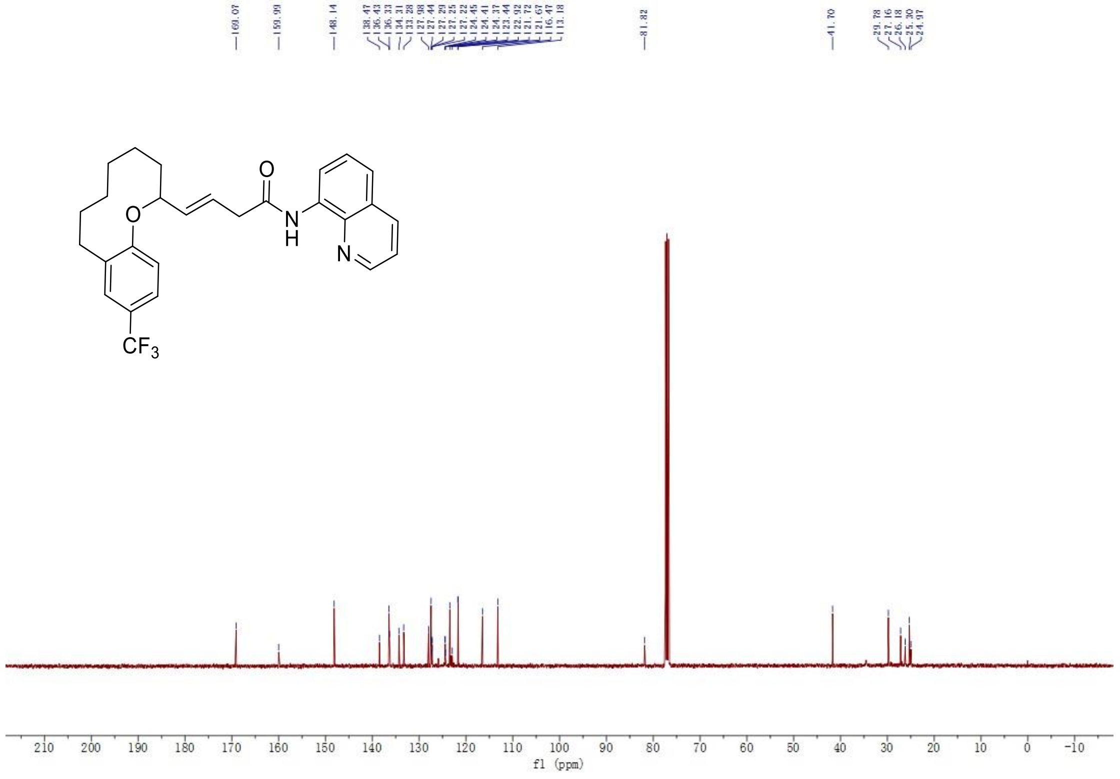

^{19}**F NMR**-spectrum (376 MHz, $CDCl_3$) of **67**

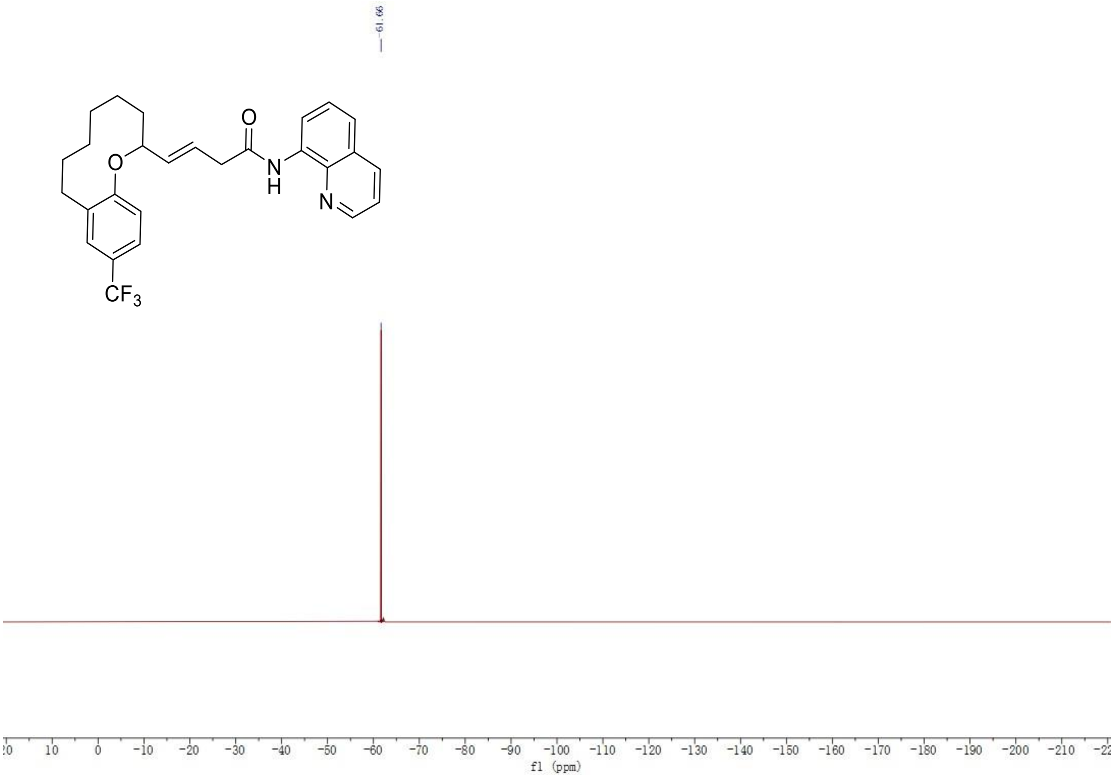

^{1}**H NMR**-spectrum (400 MHz, $CDCl_3$) of **68**

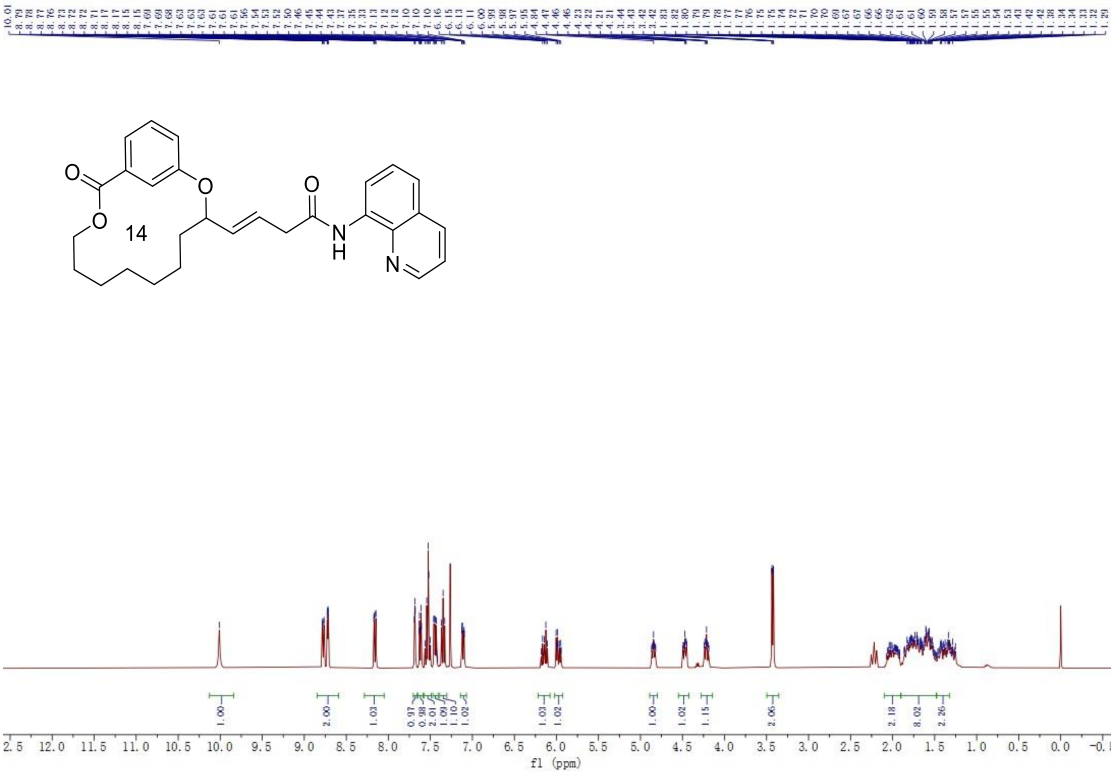

^{13}**C NMR**-spectrum (101 MHz, CDCl3) of **68**

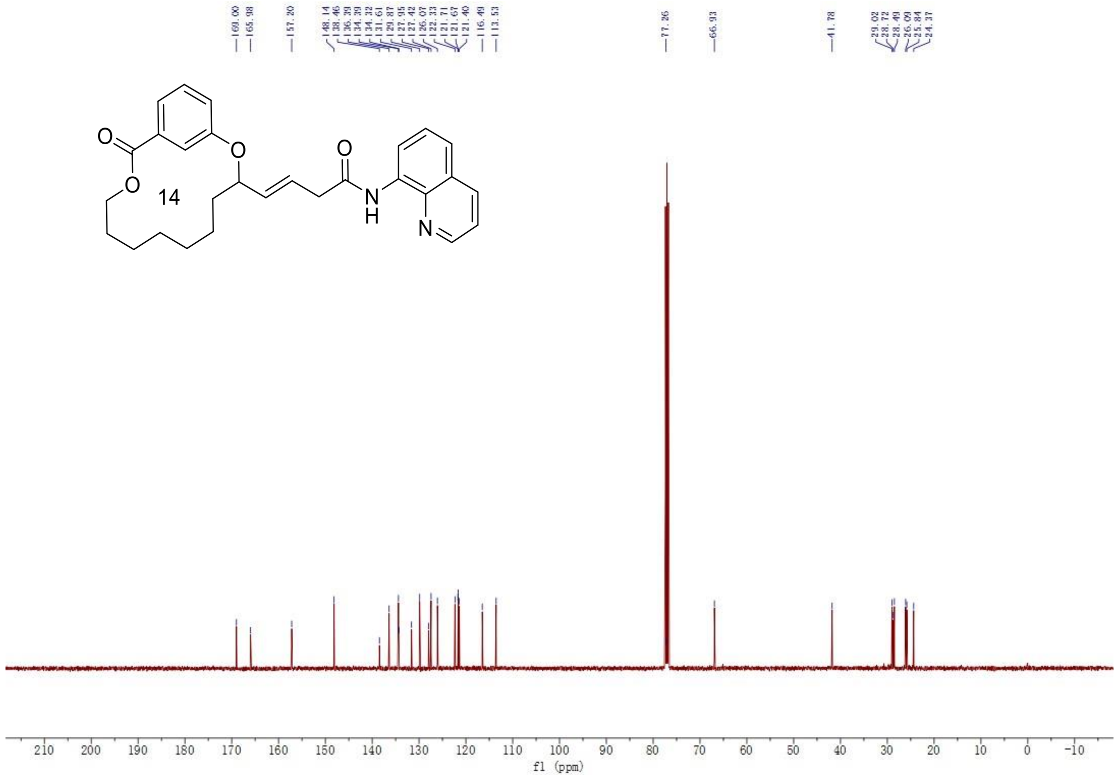

^{1}**H NMR**-spectrum (400 MHz, CDCl3) of **69**

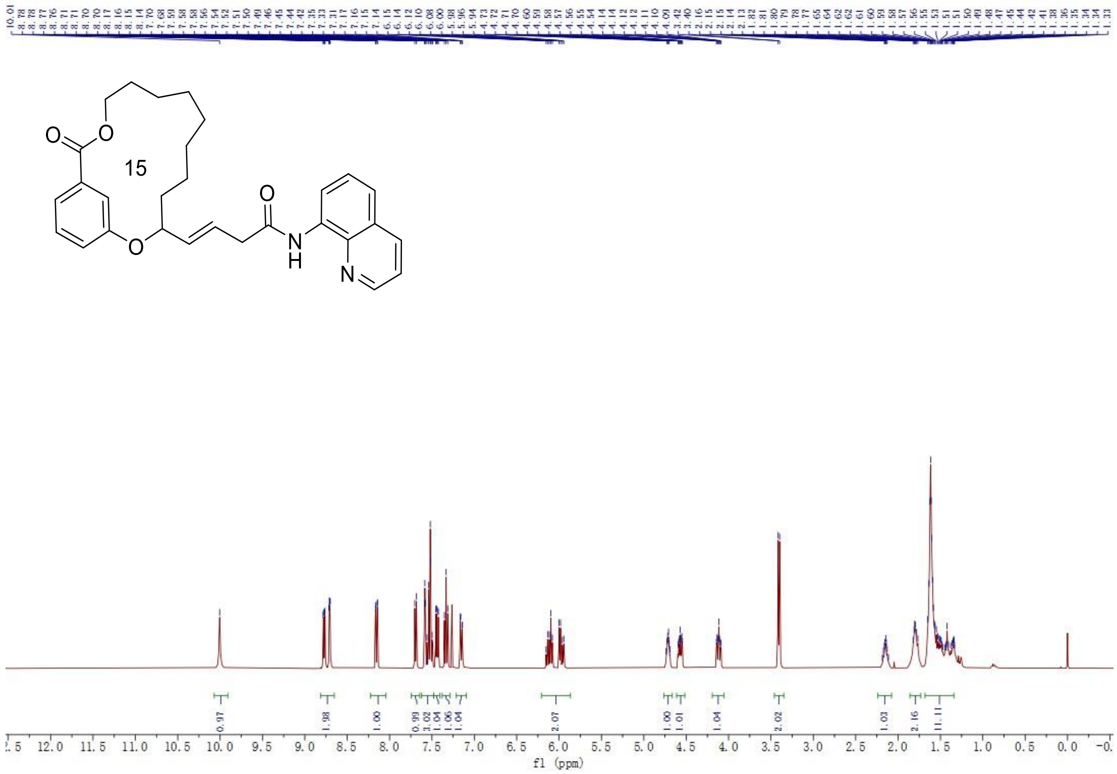

^{13}**C NMR**-spectrum (101 MHz, $CDCl_3$) of **69**

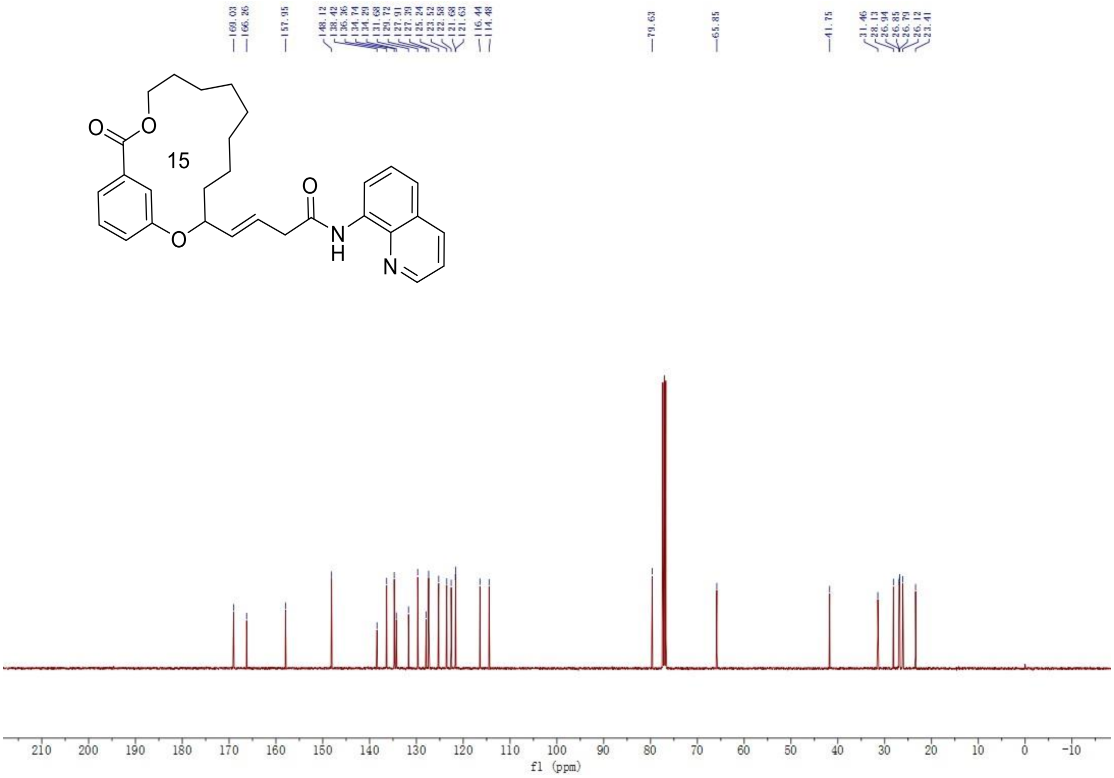

^{1}**H NMR**-spectrum (400 MHz, $CDCl_3$) of **70**

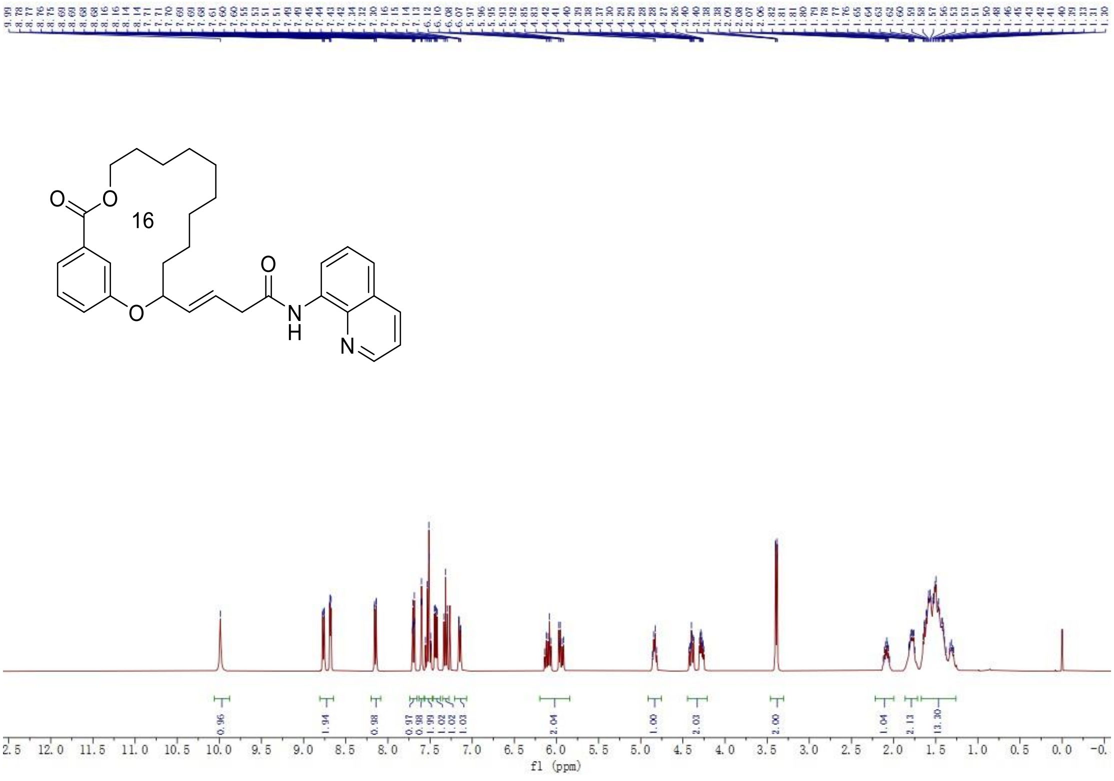

[13]**C NMR**-spectrum (101 MHz, CDCl3) of **70**

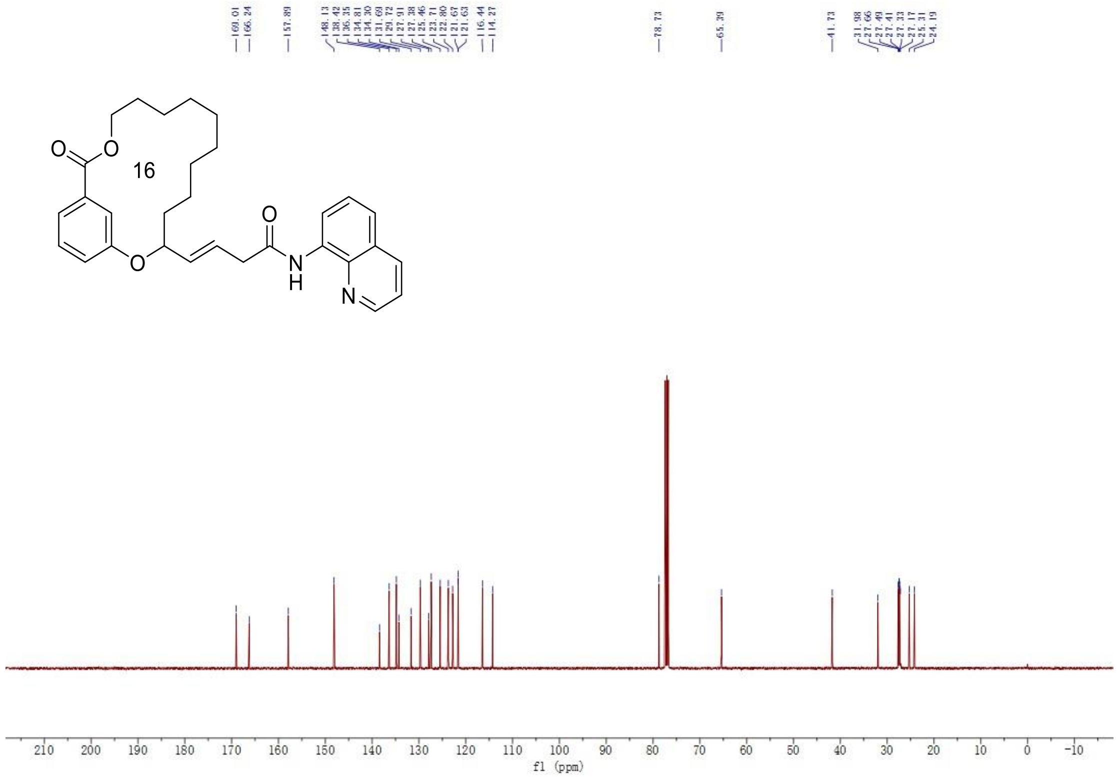

[1]**H NMR**-spectrum (400 MHz, CDCl3) of **71**

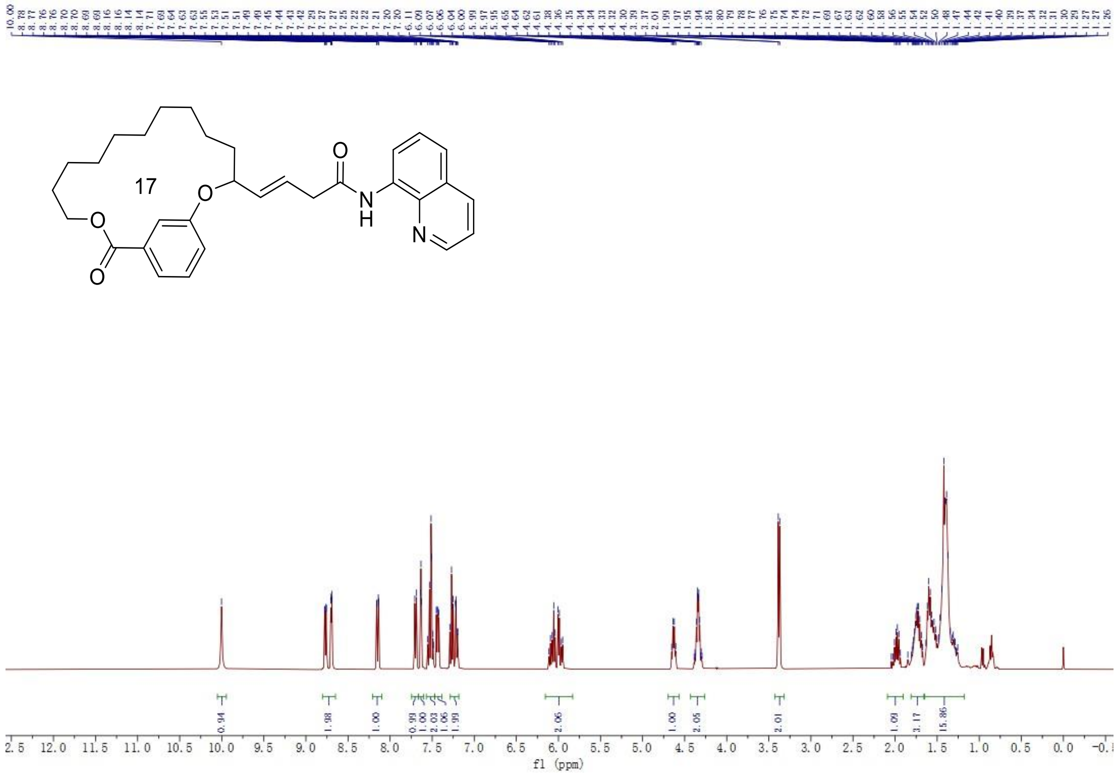

^{13}C NMR-spectrum (101 MHz, CDCl3) of **71**

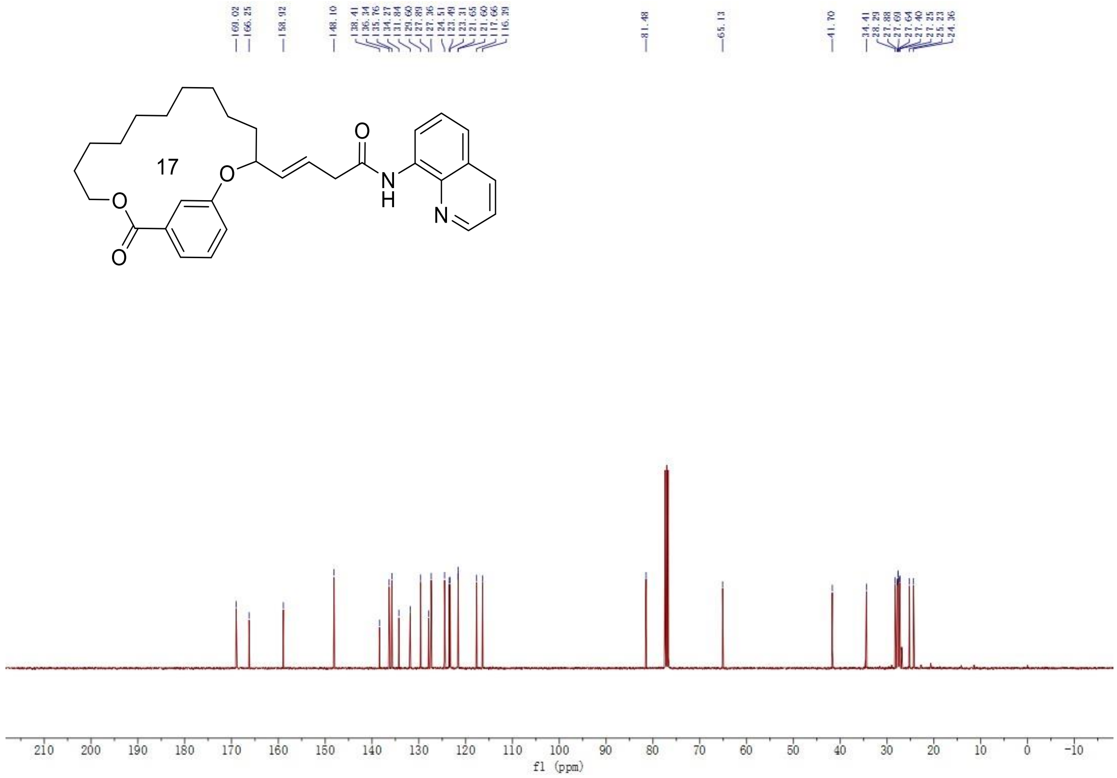

^{1}H NMR-spectrum (400 MHz, CDCl3) of **72**

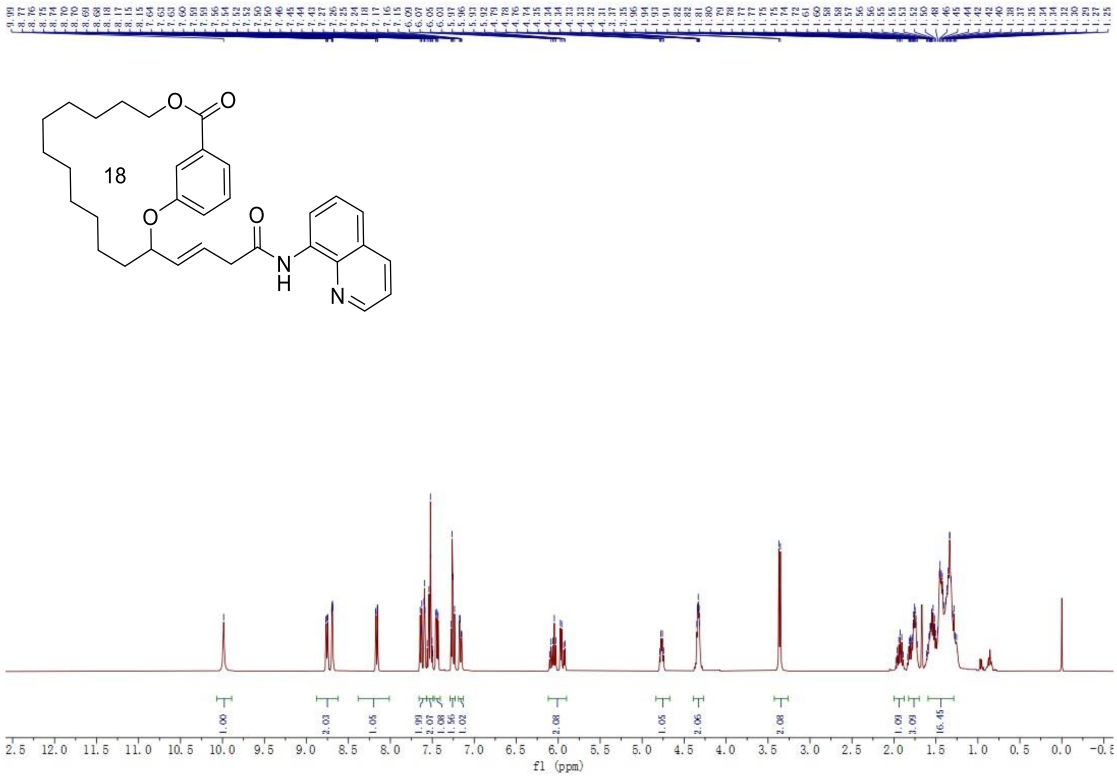

^{13}C NMR-spectrum (101 MHz, CDCl3) of **72**

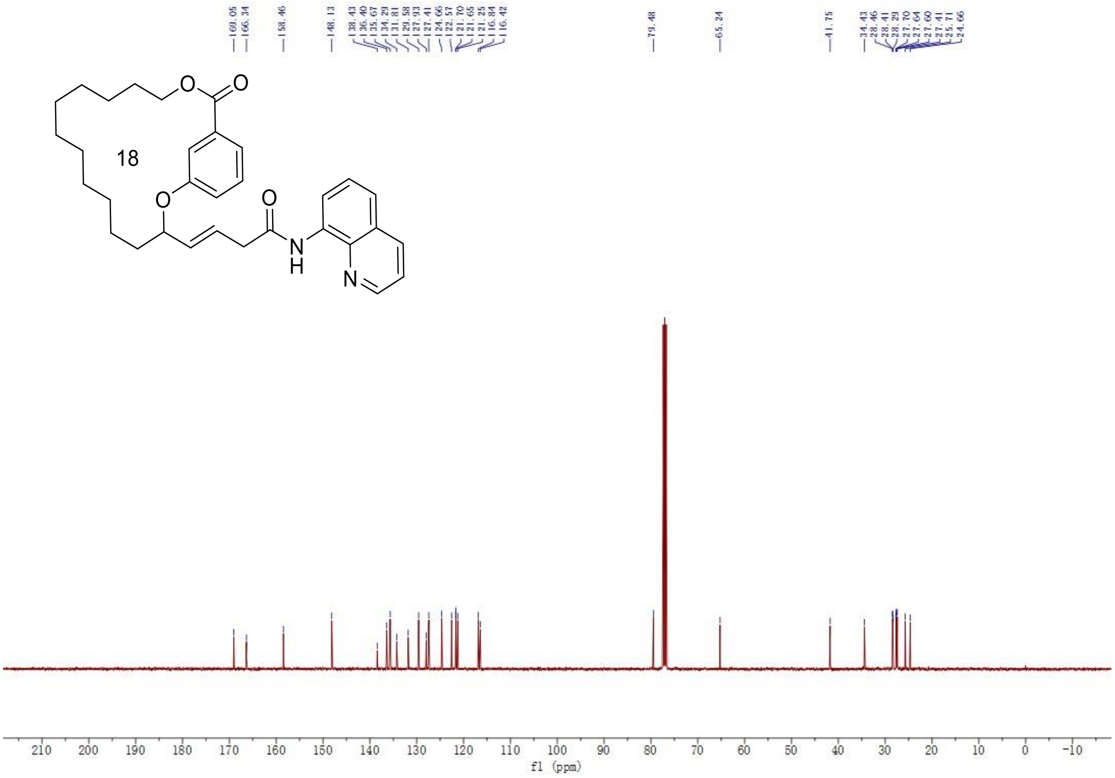

^{1}H NMR-spectrum (400 MHz, CDCl3) of **73**

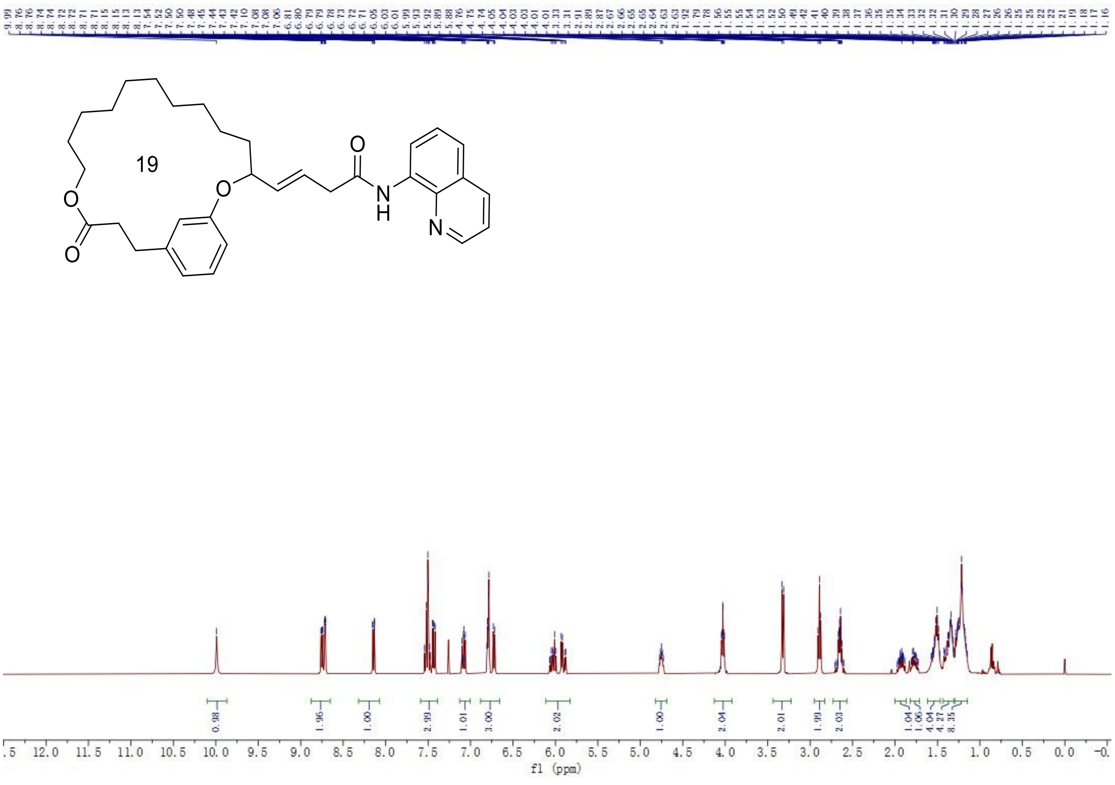

[13]**C NMR**-spectrum (101 MHz, CDCl3) of **73**

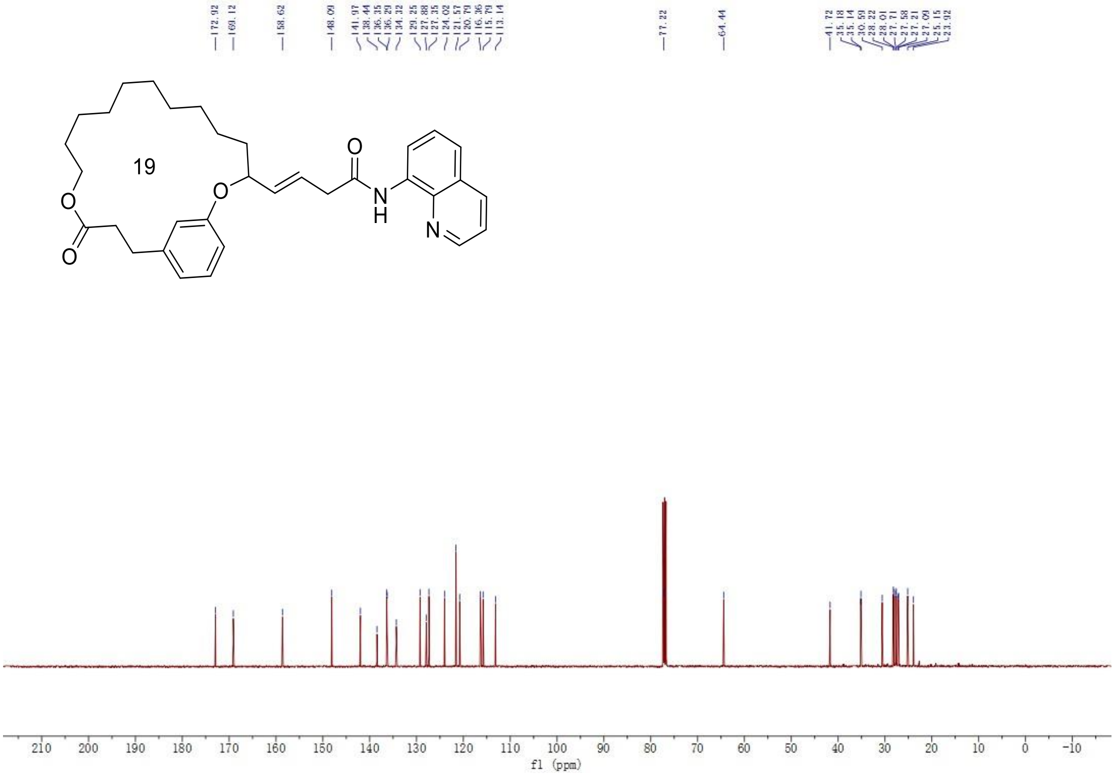

[1]**H NMR**-spectrum (400 MHz, CDCl3) of **74**

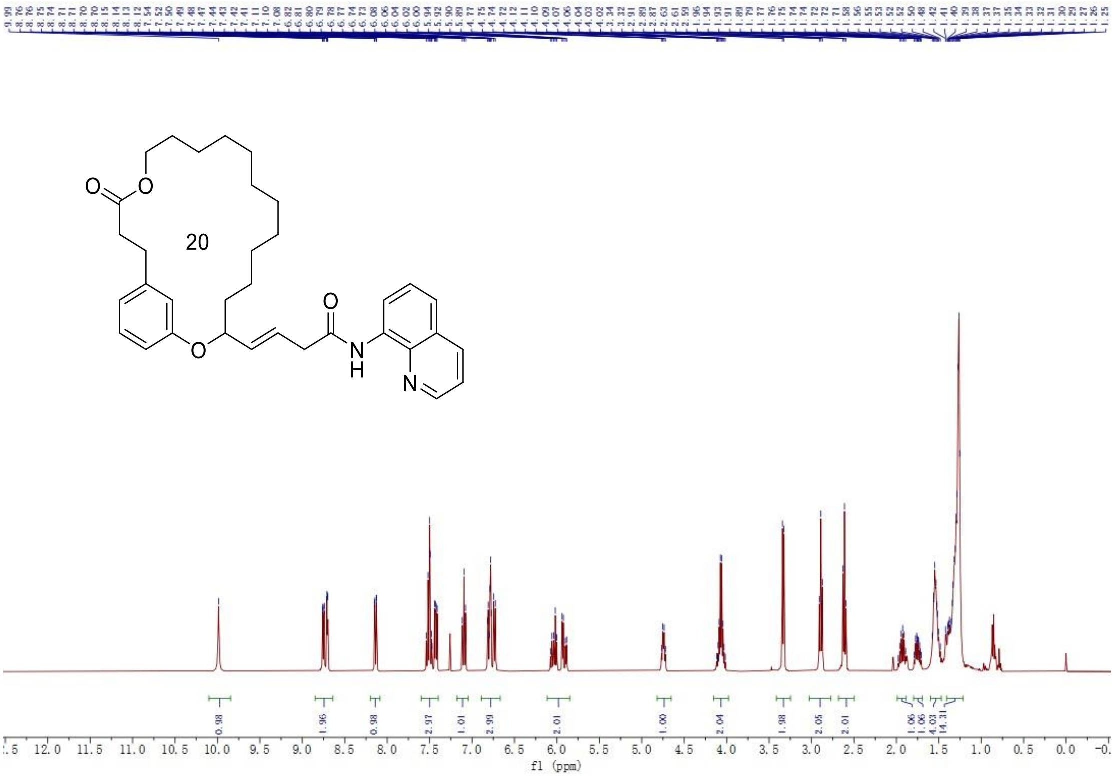

[13]**C NMR**-spectrum (101 MHz, CDCl3) of **74**

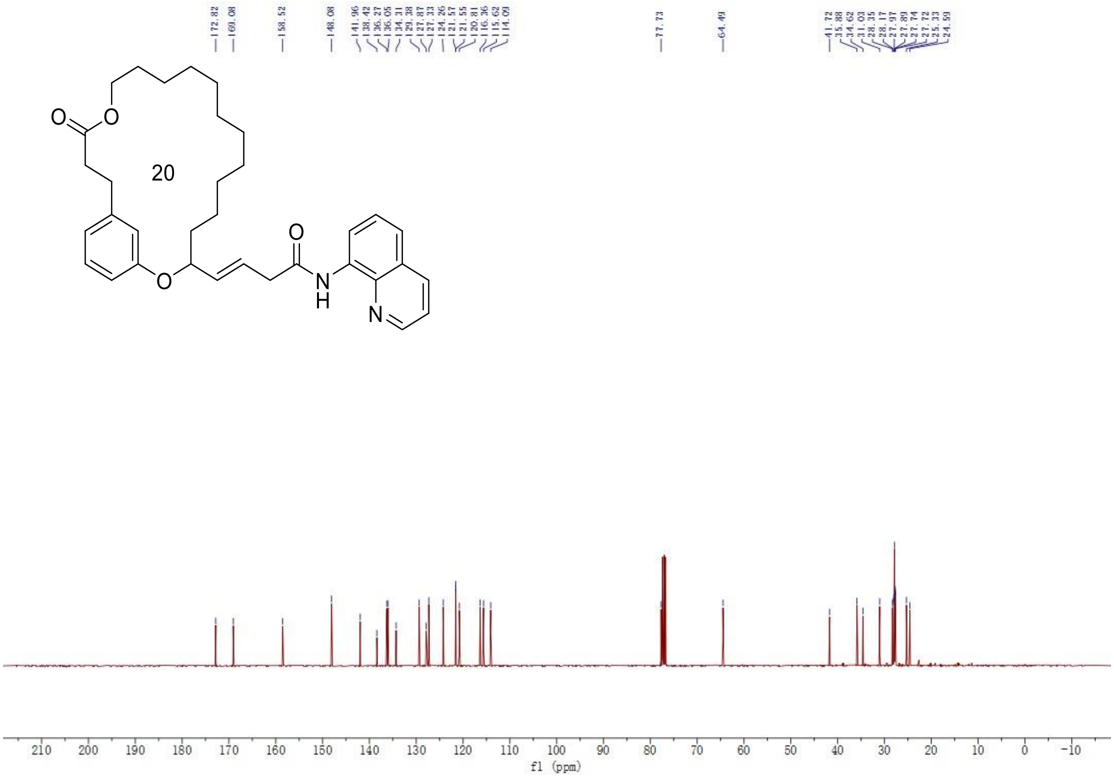

[1]**H NMR**-spectrum (400 MHz, CDCl3) of **75**

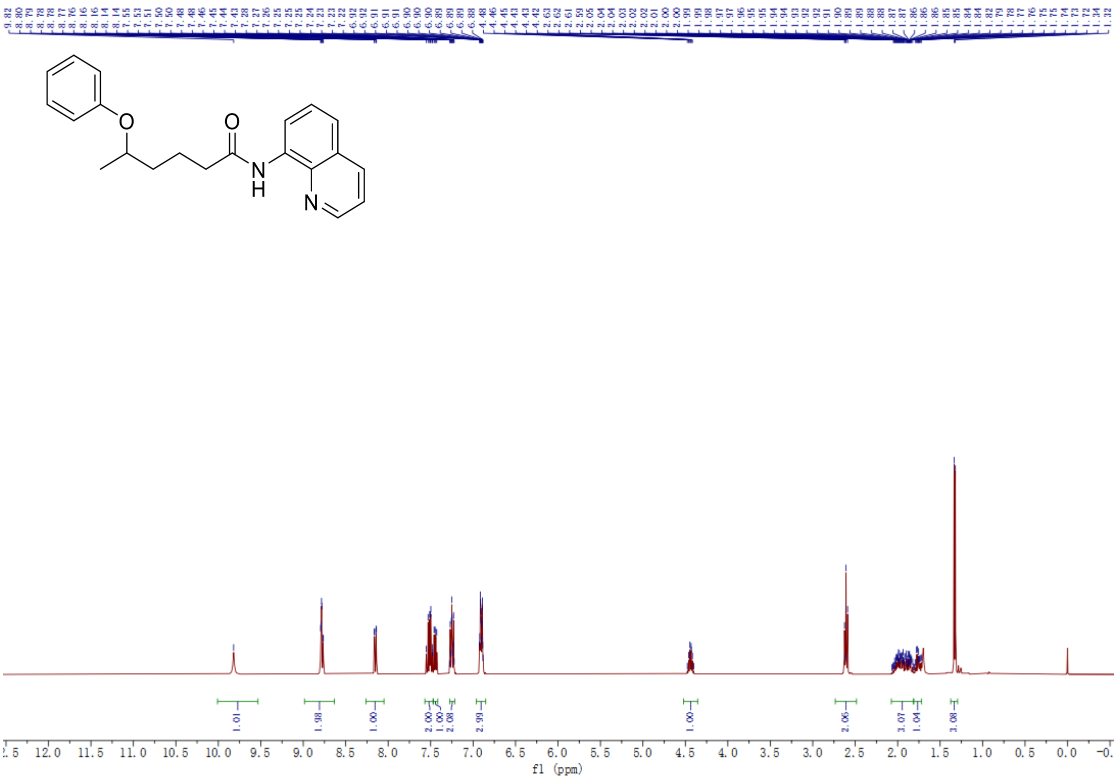

^{13}C NMR-spectrum (101 MHz, CDCl3) of **75**

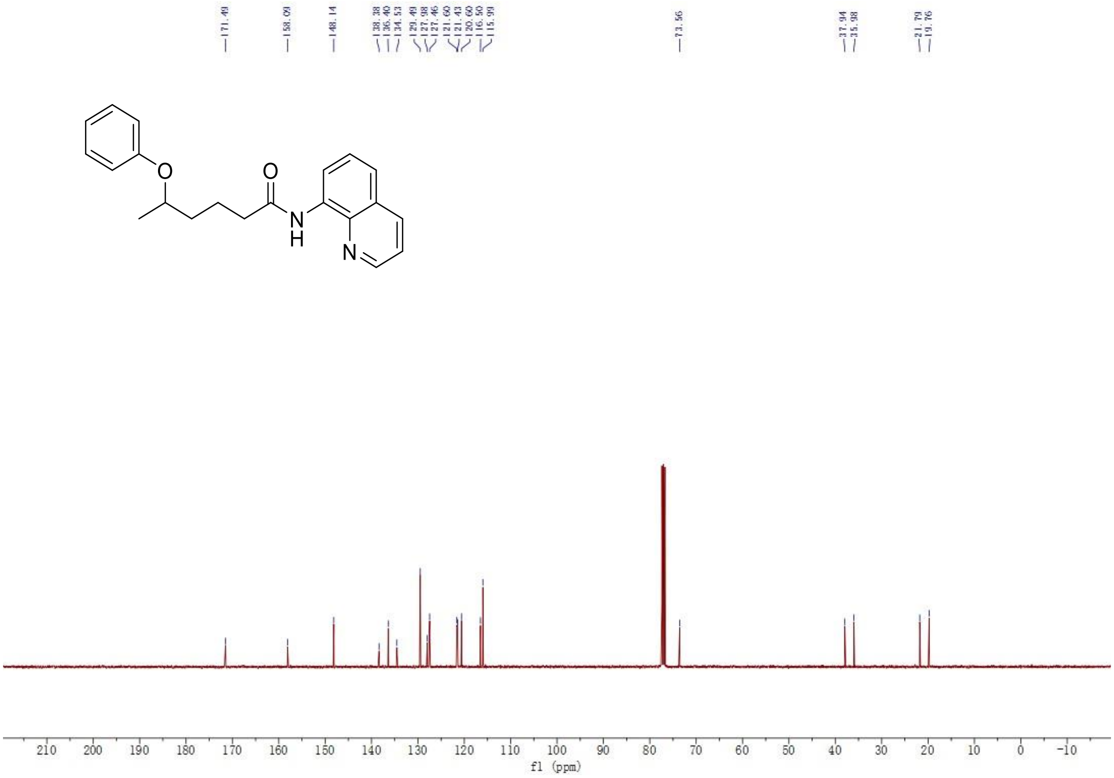

^{1}H NMR-spectrum (400 MHz, CDCl3) of **76**

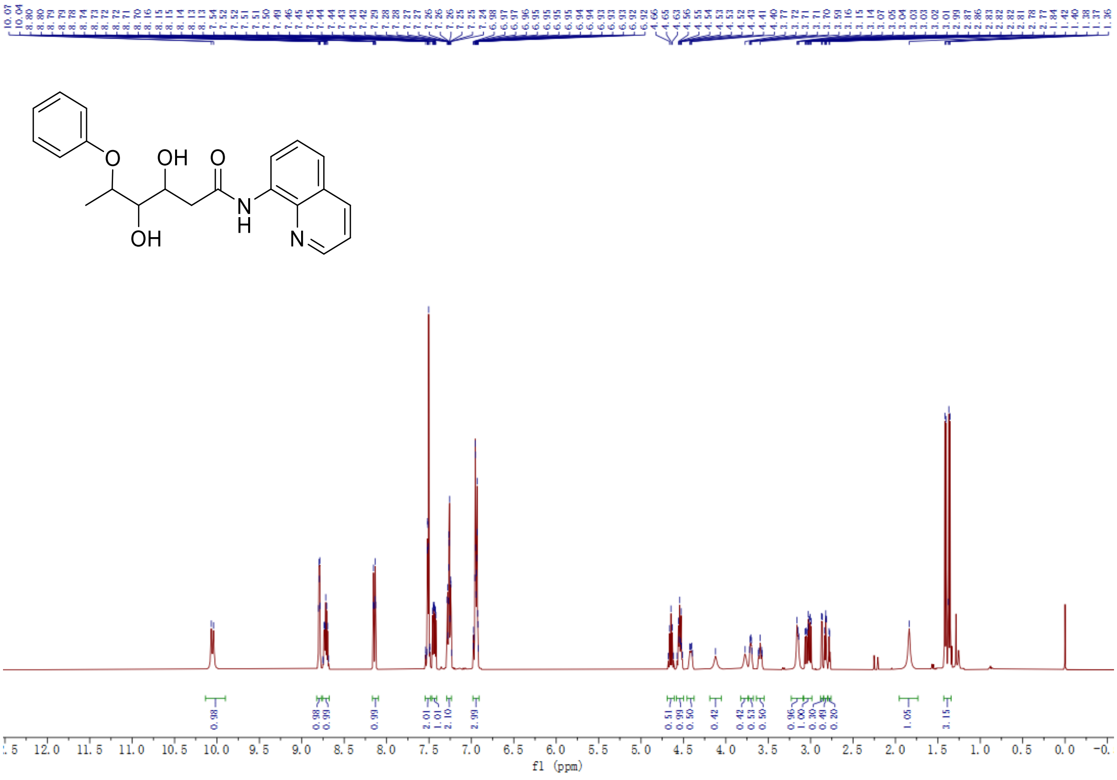

^{13}C NMR-spectrum (101 MHz, $CDCl_3$) of **76**

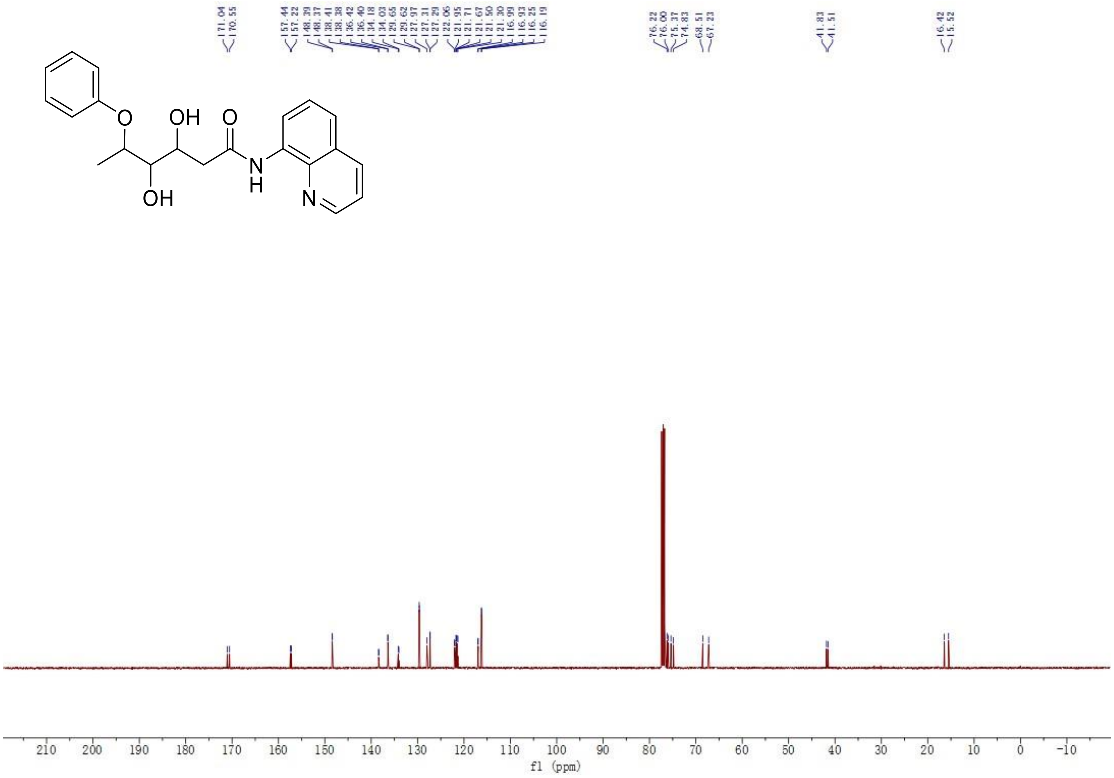

^{1}H NMR-spectrum (400 MHz, $CDCl_3$) of **77**

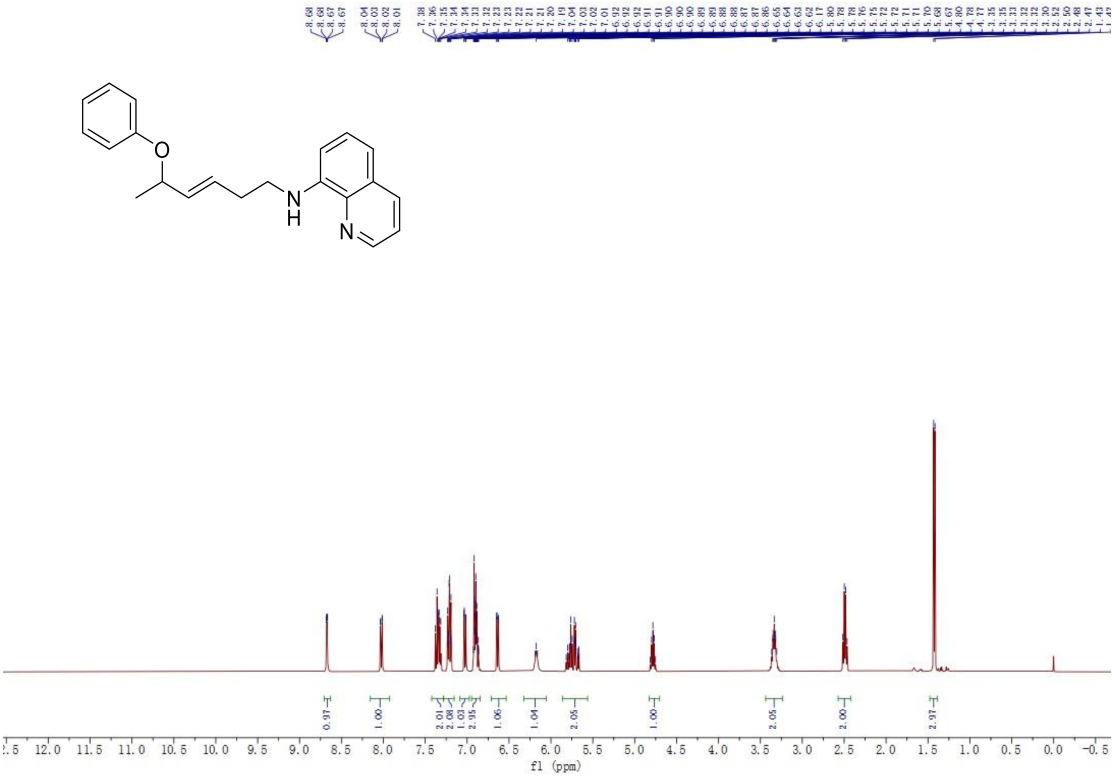

^{13}C NMR-spectrum (101 MHz, $CDCl_3$) of **77**

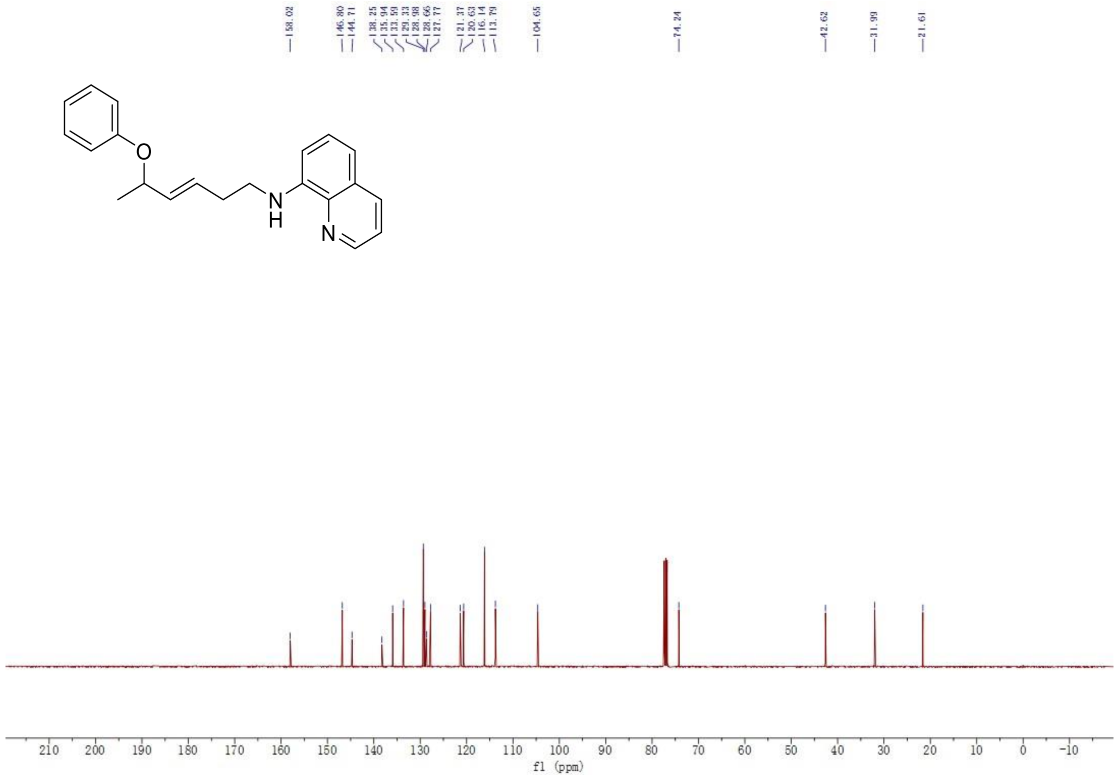

^{1}H NMR-spectrum (400 MHz, $CDCl_3$) of **78**

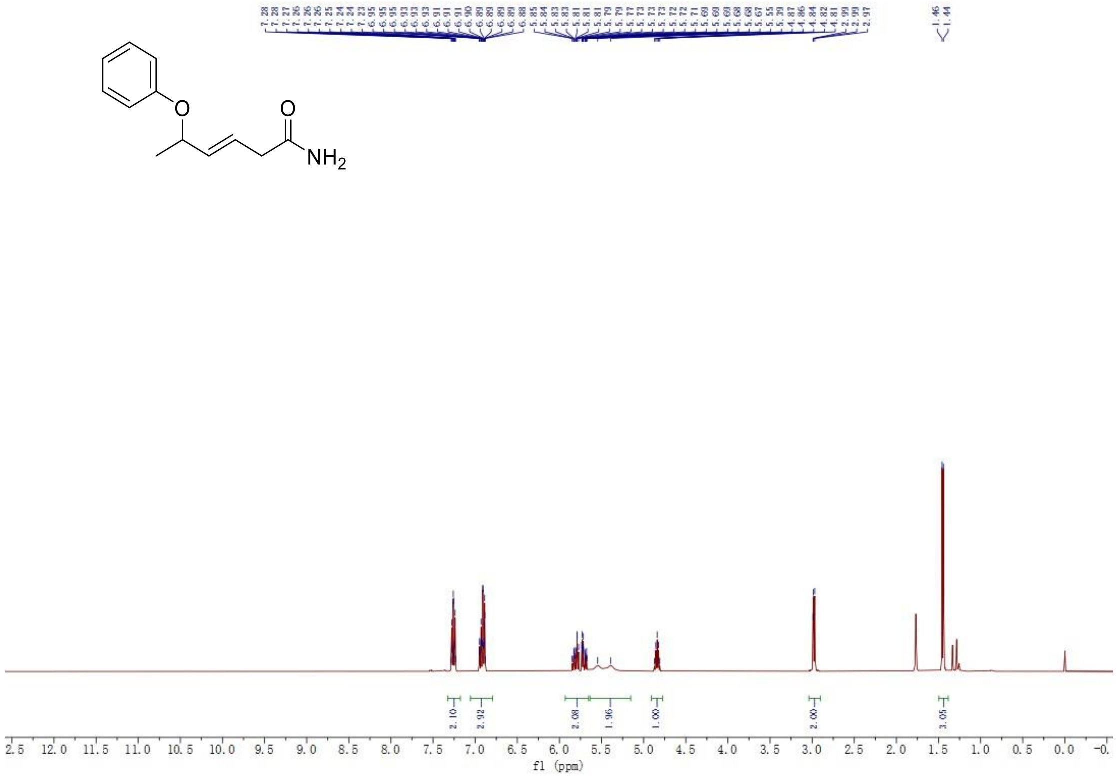

[13]**C NMR**-spectrum (101 MHz, $CDCl_3$) of **78**

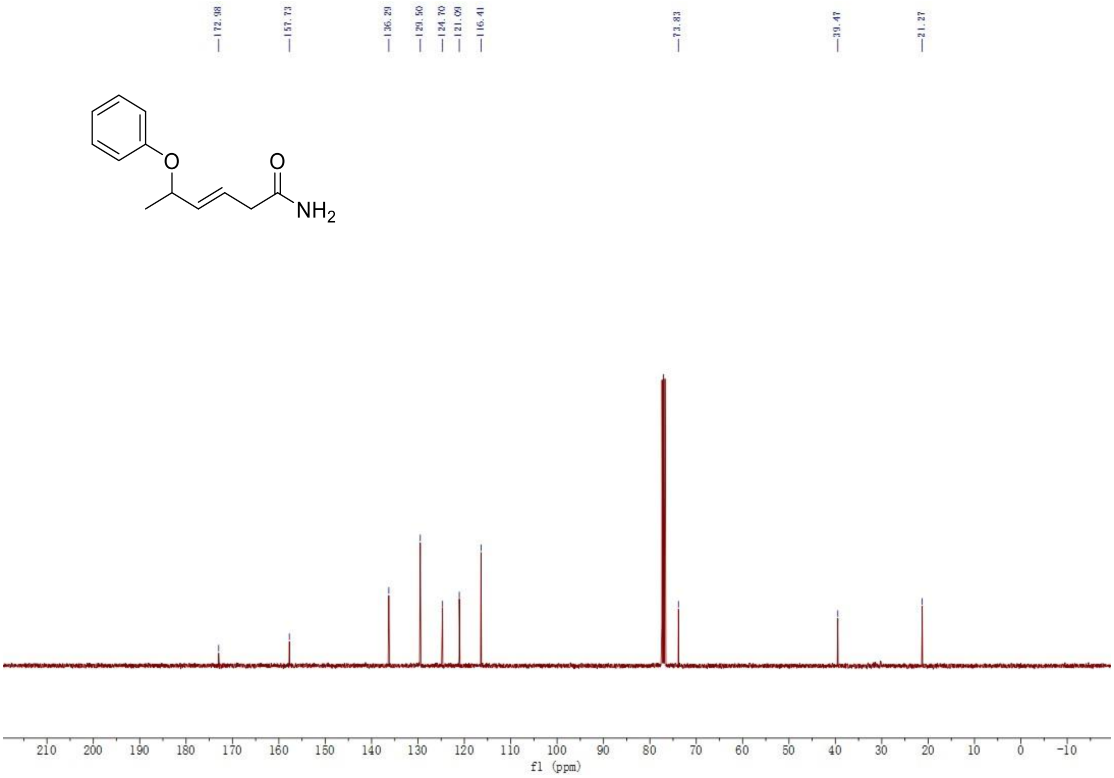

[1]**H NMR**-spectrum (400 MHz, $CDCl_3$) of **79**

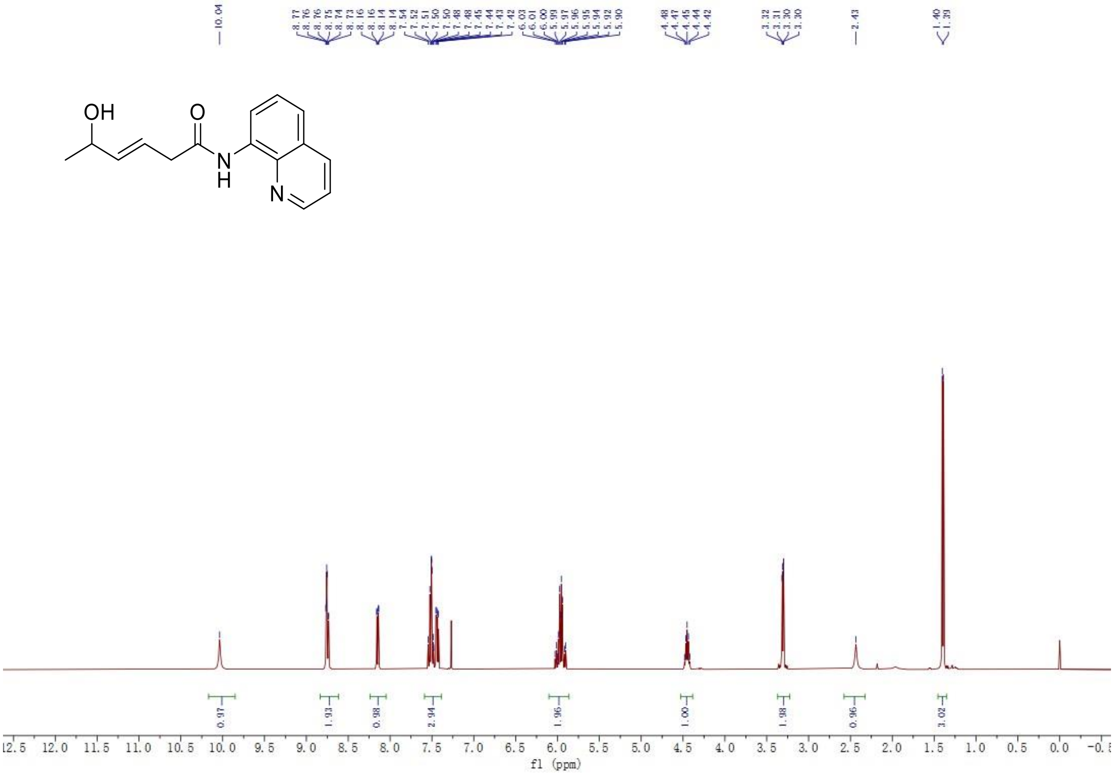

^{13}C NMR-spectrum (101 MHz, $CDCl_3$) of **79**

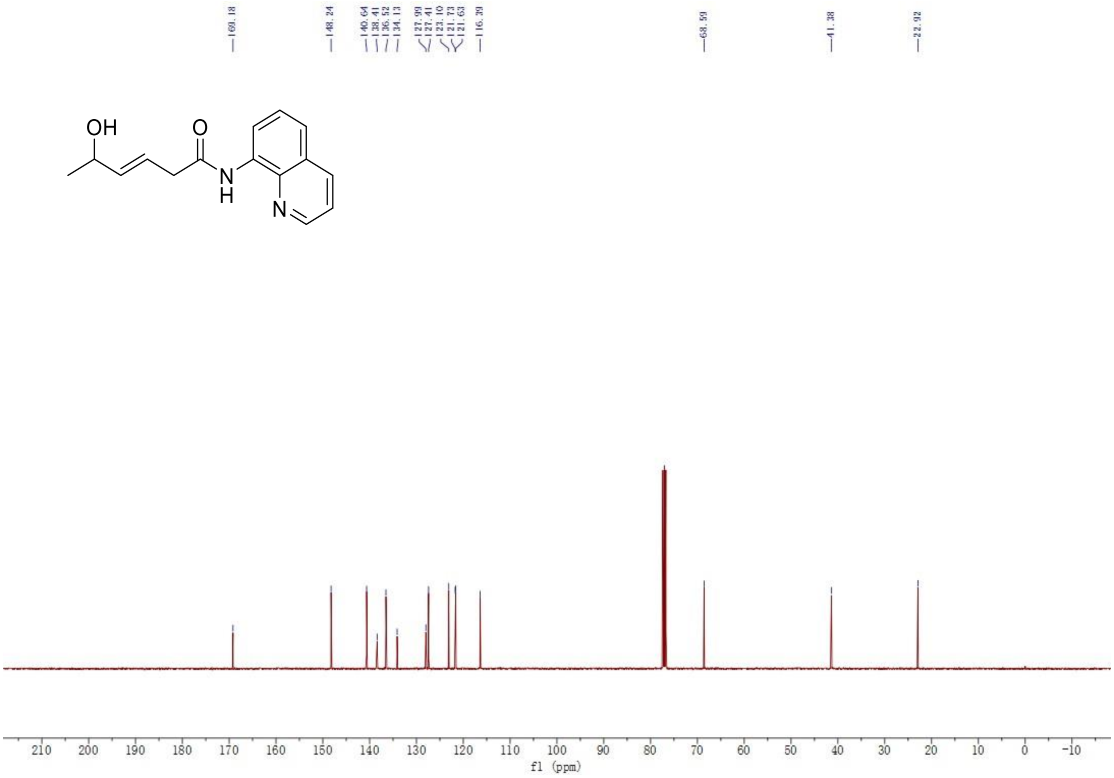

11. References

1. Wang, H., Bai, Z.-B., Jiao, T.-Q. & Chen, G. Site-Selective 1,1-Difunctionalization of Unactivated Alkenes Enabled by Cationic Palladium Catalysis. *J. Am. Chem. Soc.* **140**, 3542-3546 (2018).
2. Jeon, J., Ryu, H. & Hong, S. Site-Selective 1,1-Difunctionalization of Unactivated Alkenes Enabled by Cationic Palladium Catalysis. *J. Am. Chem. Soc.* **141**, 10048-10059 (2019).
3. Wang, L., Wang, C.-L., Li, Z.-H., Lian, P.-F., Kang, J.-C., Zhou, J., Hao, Y., Liu, R.-X., Bai, H.-Y. & Zhang, S.-Y. Cooperative Cu/azodiformate system-catalyzed allylic C–H amination of unactivated internal alkenes directed by aminoquinoline. *Nature Commun* **15**, 1483 (2024).
4. Zhao, Y. & Truhlar, D. G. The M06 suite of density functionals for main group thermochemistry, thermochemical kinetics, noncovalent interactions, excited states, and transition elements: two new functionals and systematic testing of four M06-class functionals and 12 other functionals. *Theor Chem Acc.* **120**, 215-241 (2008).
5. (a) Hay, P. J. & Wadt, W. R. Ab Initio Effective Core Potentials for Molecular

Calculations. Potentials for K to Au Including the outermost Core Orbitals. *J. Chem. Phys.* **82**, 299-310 (1985). (b) Roy, L. E., Hay, P. J. & Martin, R. L. Revised Basis Sets for the LANL Effective Core Potentials. *J. Chem. Theory Comput*. **4**, 1029-1031 (2008).

6. (a) Krishnan, R., Binkley, J. S., Seeger, R. & Pople, J. A. Self-consistent molecular orbital methods. XX. A basis set for correlated wave functions. *J. Chem. Phys.* **72**, 650-654 (1980). (b) McLean, A. D. & Chandler, G. S. Contracted Gaussian basis sets for molecular calculations. I. Second row atoms, Z=11–18. *J. Chem. Phys.* **72**, 5639-5648 (1980).
7. Marenich, A. V., Cramer, C. J. & Truhlar, D. G. Universal Solvation Model Based on Solute Electron Density and on a Continuum Model of the Solvent Defined by the Bulk Dielectric Constant and Atomic Surface Tensions. *J. Phys. Chem. B.* **113**, 6378-6396 (2009).
8. (a) Dolg, M., Wedig, U., Stoll, H. & Preuss, H. J. Energy-adjusted ab initio pseudopotentials for the first row transition elements. *Chem. Phys.* **86**, 866-872 (1987). (b) Andrae, D., Häußermann, U., Dolg, M., Stoll, H. & Preuß, H. Energy-adjusted ab initio pseudopotentials for the second and third row transition elements. *Theor. Chim. Acta*. **77**, 123-141 (1990).
9. Gaussian 16, Revision A.03, M. J. Frisch, G. W. Trucks, H. B. Schlegel, G. E. Scuseria, M. A. Robb, J. R. Cheeseman, G. Scalmani, V. Barone, G. A. Petersson, H. Nakatsuji, X. Li, M. Caricato, A. V. Marenich, J. Bloino, B. G. Janesko, R. Gomperts, B. Mennucci, H. P. Hratchian, J. V. Ortiz, A. F. Izmaylov, J. L. Sonnenberg, D. Williams-Young, F. Ding, F. Lipparini, F. Egidi, J. Goings, B. Peng, A. Petrone, T. Henderson, D. Ranasinghe, V. G. Zakrzewski, J. Gao, N. Rega, G. Zheng, W. Liang, M. Hada, M. Ehara, K. Toyota, R. Fukuda, J. Hasegawa, M. Ishida, T. Nakajima, Y. Honda, O. Kitao, H. Nakai, T. Vreven, K. Throssell, J. A. Montgomery, Jr., J. E. Peralta, F. Ogliaro, M. J. Bearpark, J. J. Heyd, E. N. Brothers, K. N. Kudin, V. N. Staroverov, T. A. Keith, R. Kobayashi, J. Normand, K. Raghavachari, A. P. Rendell, J. C. Burant, S. S. Iyengar, J. Tomasi, M. Cossi, J. M. Millam, M. Klene, C. Adamo, R. Cammi, J. W. Ochterski, R. L. Martin, K. Morokuma, O. Farkas, J. B. Foresman, and D. J. Fox, Gaussian, Inc., Wallingford CT, 2016.
